# Electron‐Rich Diruthenium Complexes with π‐Extended Alkenyl Ligands and Their F_4_TCNQ Charge‐Transfer Salts**

**DOI:** 10.1002/chem.202104403

**Published:** 2022-03-18

**Authors:** Rajorshi Das, Michael Linseis, Stefan M. Schupp, Lukas Schmidt‐Mende, Rainer F. Winter

**Affiliations:** ^1^ Fachbereich Chemie Universität Konstanz Universitätsstrasse 10 78457 Konstanz Germany; ^2^ Fachbereich Physik Universität Konstanz Universitätsstrasse 10 78457 Konstanz Germany

**Keywords:** charge-transfer salts, donor-acceptor complexes, electrochemistry, extended π-conjugated systems, ruthenium

## Abstract

The synthesis of dinuclear ruthenium alkenyl complexes with {Ru(CO)(P^*i*^Pr_3_)_2_(L)} entities (L=Cl^−^ in complexes **Ru_2_‐3** and **Ru_2_‐7**; L=acetylacetonate (acac^−^) in complexes **Ru_2_‐4** and **Ru_2_‐8**) and with π‐conjugated 2,7‐divinylphenanthrenediyl (**Ru_2_‐3**, **Ru_2_‐4**) or 5,8‐divinylquinoxalinediyl (**Ru_2_‐7**, **Ru_2_‐8**) as bridging ligands are reported. The bridging ligands are laterally π‐extended by anellating a pyrene (**Ru_2_‐7**, **Ru_2_‐8**) or a 6,7‐benzoquinoxaline (**Ru_2_‐3**, **Ru_2_‐4**) π‐perimeter. This was done with the hope that the open π‐faces of the electron‐rich complexes will foster association with planar electron acceptors via π‐stacking. The dinuclear complexes were subjected to cyclic and square‐wave voltammetry and were characterized in all accessible redox states by IR, UV/Vis/NIR and, where applicable, by EPR spectroscopy. These studies signified the one‐electron oxidized forms of divinylphenylene‐bridged complexes **Ru_2_‐7**, **Ru_2_‐8** as intrinsically delocalized mixed‐valent species, and those of complexes **Ru_2_‐3** and **Ru_2_‐4** with the longer divinylphenanthrenediyl linker as partially localized on the IR, yet delocalized on the EPR timescale. The more electron‐rich acac^−^ congeners formed non‐conductive 1 : 1 charge‐transfer (CT) salts on treatment with the F_4_TCNQ electron acceptor. All spectroscopic techniques confirmed the presence of pairs of complex radical cations and F_4_TCNQ^.−^ radical anions in these CT salts, but produced no firm evidence for the relevance of π‐stacking to their formation and properties.

## Graphical abstract

**The synthesis of** charge‐transfer salts from diruthenium complexes with two differently extended π‐conjugated ligands and the strong electron‐acceptor F_4_TCNQ is presented. These salts have been characterized by EPR, UV/Vis/NIR (in solution and solid states) and IR spectroscopy.

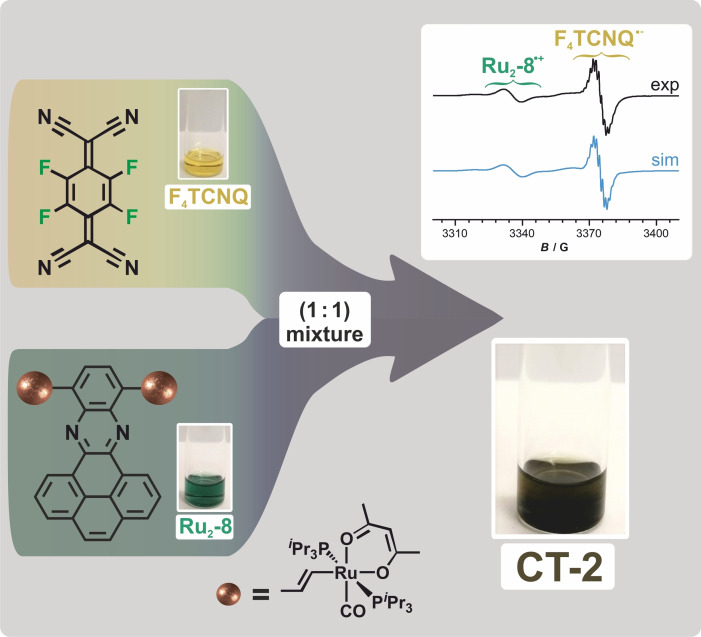

## Introduction

Charge‐transfer (CT) salts have emerged as an important area of research over the last few decades. Particular emphasis was put on the realization of redox‐responsive, ordered and nanostructured materials, which can serve as electrical (semi)conductors and find applications in molecular electronics and magnetochemistry.^[1]^ Such materials may exhibit complete or partial electron transfer between a planar electron donor (D) and a likewise planar electron acceptor (A). Depending on the degree of electron transfer (or ionicity) and the ensuing multitopic, noncovalent interactions in the solid state, CT salts may adopt several different types of structures, ranging from simple ion pairs to alternating D^δ+^⋅⋅⋅A^δ−^ or (D_n_)^δ+^⋅⋅⋅(A_m_)^δ−^ stacks, or pairs of segregated (D_n_)^δ+^ and (A_m_)^δ−^ stacks, often with formally fractional charges δ (0≤δ≤1) on the individual D^δ+^ or A^δ−^ constituents. The packing motifs in the solid state have often profound influence on the electronic structures and electrical conductivities of such CT salts.[^1^, ^2^]

Owing to their favourable electronic properties, tetrathiafulvalene (TTF) and its many derivatives as well as a wide variety of purely organic polycyclic aromatic hydrocarbons (PAHs) have been employed as electron‐rich donor constituents of CT salts.[^1^, ^3^] The delocalized molecular orbitals (MOs) of such extended π‐conjugated systems and their propensity to undergo redox processes at moderate potentials are also essential for their (poly)electrochromism and their tuneable optical absorption and emission properties.^[4]^ Despite the importance and diverse applications of planar electron‐rich PAHs as constituents of CT salts, the use of transition metal complexes with π‐extended PAH ligands for such purposes is considerably less well documented in the literature.^[5]^ Notable exceptions are CT salts with metallocenes,^[6]^ first and foremost ferrocenes,[^6c^, ^7^] or metal dithiolene or enedithiolate complexes[^6c^, ^7e^, ^8^] as electron donors. Other examples include CT salts of metal complexes with porphyrinic or pyridine‐appended TTF derivatives as ligands.^[9]^

**Figure 1:**
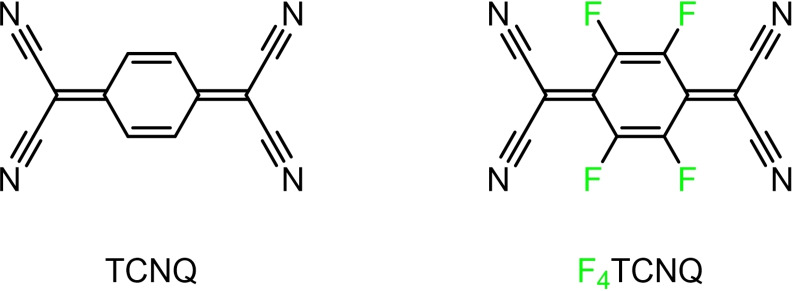
Molecular structures of 7,7,8,8‐tetracyanoquinodimethane (TCNQ) and 2,3,5,6‐tetrafluoro‐7,7,8,8‐tetracyanoquinodimethane (F_4_TCNQ).

Owing to its strong electron‐accepting abilities, TCNQ (7,7,8,8‐tetracyanoquinodimethane, Figure 1) is a widely used acceptor, which has found versatile applications in various fields, including energy and data storage,^[10]^ superconductivity,^[11]^ optical and electrical recording,^[12]^ catalysis,^[13]^ light‐emitting materials, or sensor devices.^[14]^ It readily forms CT salts with many common electron donors like π‐extended PAHs or TTF and its many derivatives.[^1a^, ^1b^] In the presence of transition metal ions, TCNQ can also bind coordinatively unsaturated transition metal complex entities with formation of direct M‐TCNQ bonds via nitrogen lone‐pairs of the nitrile functionalities.^[15]^

Its tetrafluoro variation F_4_TCNQ (F_4_TCNQ=2,3,5,6‐tetrafluoro‐7,7,8,8‐tetracyanoquinodimethane) has even superior acceptor properties as reflected by its considerably lower LUMO energy (LUMO=energetically lowest unoccupied molecular orbital).^[16]^ Moreover, electrochemical data indicate that the reductions of F_4_TCNQ to first the radical anion F_4_TCNQ^.−^ and subsequently to the dianion F_4_TCNQ^2−^ are fully reversible, which is an important prerequisite for the stability of their CT salts. In addition, the presence of four fluorine atoms enables F_4_TCNQ^n−^ (n=0, 1, or 2) to engage in strong hydrogen bonding or other stabilizing non‐covalent E⋅⋅⋅F contacts (e. g., E=S, Se), which can promote intermolecular association and electronic interactions.[^16c^, ^17^] These assets have made F_4_TCNQ a widely used acceptor in the context with PAH donors,[^1a^, ^2b^, ^15b^, ^18^] and the acceptor of choice for our present study.

Some of us have a long‐standing interest in electron‐rich bis(alkenyl)arylene‐bridged diruthenium complexes [{(PR_3_)_2_(L)(X)(CO)Ru}_2_(μ‐CH=CH−Aryl−CH=CH)] (L=PR_3_, a pyridine derivative or a vacant coordination site, X=Cl^−^, or (L)(X)=bidentate monoanionic carboxylate or β‐ketoenolate four‐electron donor ligand) and their electron‐transfer properties.^[19]^ These complexes are oxidized stepwise to first their radical cations and then their dications and exhibit strong polyelectrochromism in the visible (Vis) and the near infrared (NIR) as well as thermally accessible open‐shell diradical states or diradical ground states at their dication level. Their oxidation potentials are considerably lower than those of the corresponding divinyl‐ or diethynyl‐substituted parent arenes. This makes such complexes interesting donors for CT salts. However, the bulky, strongly electron‐donating P^*i*^Pr_3_ ligands that are required in order to chemically stabilize the otherwise reactive oxidized forms of these complexes cover a large part of the π‐conjugated linker and thus prevent a close approach of such complexes and planar, π‐extended acceptors. Initial experiments had shown that the electron‐rich divinylphenylene‐bridged diruthenium complex {Ru}_2_(μ‐CH=CH−C_6_H_4_−CH=CH‐1,4) (**Ru_2_‐9**) {Ru=Ru(CO)Cl(P^*i*^Pr_3_)_2_)[^19l^, ^20^] reacts readily with organic acceptors, but that the resulting compounds are unstable and decompose within few minutes. We mused that these potential drawbacks can be overcome by using π‐conjugated linkers with extended lateral expansion as can be obtained by annealing a PAH with the arylene linker. Herein, we present a comprehensive study of bis(alkenyl)arylene‐bridged diruthenium complexes with π‐extended divinylarylene ligands and the salts derived from the two most electron‐rich congeners, complexes **Ru_2_‐4** and **Ru_2_‐8**, with F_4_TCNQ. We disclose, that PAH anellation at the arylene linker is critical to render the CT salts chemically stable species with benchtop lifetimes of at least three days.

## Results and Discussion

### Synthesis and characterization

**Scheme 1:**
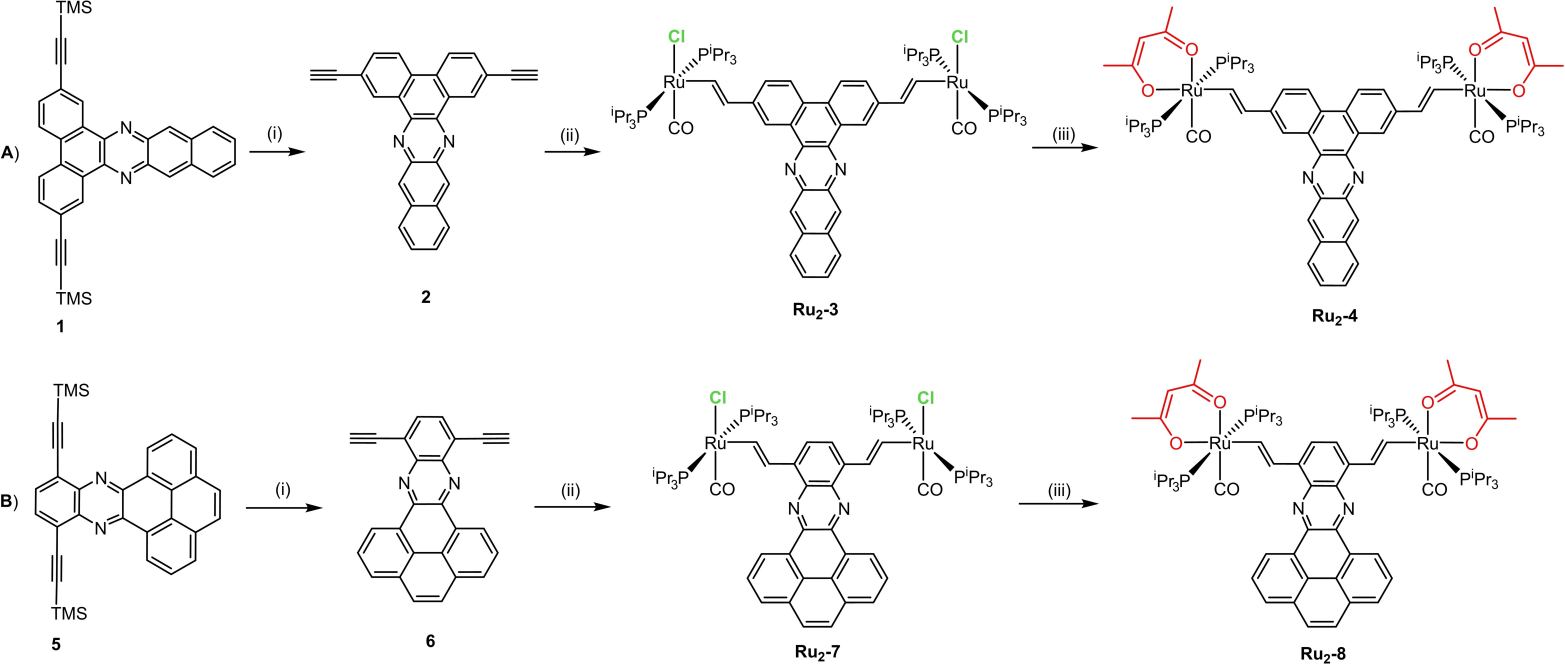
Synthesis of diruthenium complexes with extended π‐conjugated systems. Complexes **Ru_2_‐3** and **Ru_2_‐7** feature pentacoordinated ruthenium(II) centres whereas **Ru_2_‐4** and **Ru_2_‐8** feature hexacoordinated ruthenium(II) centres. Reaction conditions: (i) KF, THF/MeOH, 25 °C, 6 h; (ii) HRu(CO)Cl(P^*i*^Pr_3_)_2_, CH_2_Cl_2_, 1 h, 25 °C; (iii) CH_3_COCH_2_COCH_3_, K_2_CO_3_, CH_2_Cl_2_/MeOH, 25 °C, 4 h.

The synthesis of the reported diruthenium complexes is shown in Scheme 1. The trimethylsilyl‐ (TMS‐) protected 2,7‐diethynylphenanthrene **1** with a laterally fused dibenzophenazine constituent was obtained in an overall yield of 76 % by the acid‐catalyzed condensation of 2,7‐dibromophenanthrene‐9,10‐dione with 1,2‐diaminonapthalene, followed by Sonogashira coupling with Me_3_Si−C≡CH (TMSA, see the Supporting Information for experimental details). TMS‐protected 5,8‐diethynylquinoxaline **5** with a condensed pyrene pendent was synthesized by a slightly modified procedure adapted from Bunz et al.^[4]^ The subsequent removal of the Me_3_Si protecting groups from **1** and **5** with KF in MeOH/THF yielded the corresponding diterminal dialkynes **2** and **6** in yields of 62 % or 86 %, respectively. Arylene‐bridged diruthenium alkenyl complexes **Ru_2_‐3** and **Ru_2_‐7** with pentacoordinated 16 valence electron ruthenium complex entities were easily accessible by reacting one equiv. of dialkyne **2** or **6** with 2 equiv. of the hydride complex [HRu(CO)(Cl)(P^*i*^Pr_3_)_2_] in CH_2_Cl_2_. They were isolated in yields of 84 % or 72 % as dark grey‐green or dark green solids. Complexes **Ru_2_‐3** or **Ru_2_‐7** offer much higher solubilities in aromatic (e. g. benzene, toluene) and chlorinated solvents (dichloromethane, chloroform) compared to their dialkyne precursors **2** or **6**. Both complexes are moderately stable towards air and moisture. Prior studies have revealed that the electron‐donating capabilities of such complexes and the stabilities of their associated oxidized forms are further enhanced on coordinative saturation of the metal centres, which is easily achieved by replacing the chlorido ligands with bidentate, monoanionic four‐electron donors like carboxylates or β‐ketoenolates.[^19d^, ^21^] With this in mind, we converted complexes **Ru_2_‐3** and **Ru_2_‐7** to their acetylacetonato (acac^−^) derivatives **Ru_2_‐4** or **Ru_2_‐8**. The latter were isolated as leaf‐green solids in 66 % and 62 % yields, respectively.

For comparison purposes, we also prepared and investigated the new acac^−^ derivative of the “simple” 1,4‐divinylphenylene‐bridged diruthenium complex **Ru_2_‐9**,[^19l^, ^20^] i. e. the grey‐green complex {Ru(acac)(CO)(P^*i*^Pr_3_)_2_}_2_(μ‐CH=CH−C_6_H_4_−CH=CH‐1,4) (**Ru_2_‐10**) (consult the Supporting Information for experimental details and characterization data).

The precursor alkynes and the new complexes were characterized by multinuclear NMR, IR and UV/Vis/NIR spectroscopy and by mass spectrometry. NMR spectra can be found as Figures S1 to S23 in the Supporting Information. The characteristic resonances of the alkenyl protons (−C*H*=*H*−) in their ^1^H NMR spectra are located at *δ*=9.01 (Ru‐C*H*) and 6.33 ppm (Ru−CH=C*H*) for **Ru_2_‐3**, or at *δ*=9.13 and 7.63 ppm for **Ru_2_‐7** in CD_2_Cl_2_ (see the Supporting Information for spectroscopic data). The resonance of the vinylic α proton Ru−C*H* is notably shifted to lower field in the 18 valence electron acac^−^ complexes (**Ru_2_‐4**, *δ*=9.44 ppm; **Ru_2_‐8**, *δ*=9.32 ppm). The same also applies to the pair of complexes **Ru_2_‐9** and **Ru_2_‐10**, where the corresponding proton resonance shifts from *δ*=8.27 to 8.68 ppm. The acac^−^ ligands give rise to resonances at *δ*=5.41, 5.37 or 5.30 ppm for the methine and at *δ*=2.05 and 1.86, 2.01 and 1.83, or 1.91 and 1.77 ppm for the protons of the methyl groups. Corresponding ^13^C resonances of the carbonyl/enolate donors are found at *δ*=186.7 and 188.6 ppm for **Ru_2_‐4**, at 186.6 and 188.5 ppm for **Ru_2_‐8**, or at 188.3 and 186.4 ppm for **Ru_2_‐10**. The resonances of the C atoms of the carbonyl ligands are observed near *δ*=203 ppm for the 16 VE complexes **Ru_2_‐3**, **Ru_2_‐7** and **Ru_2_‐9** and are shifted to ca. 209 ppm in their 18 VE congeners with resolved ^2^ *J*_PC_ couplings of ca. 13 Hz. Similar trends prevail for the resonances of the alkenyl carbon atoms Ru‐*C*H, which are observed at *δ*=156.7, 155.2, or 148.5 ppm in **Ru_2_‐3**, **Ru_2_‐7**, or **Ru_2_‐9**, and at 169.6, 167.8 or 161.8 ppm in **Ru_2_‐4**, **Ru_2_‐8** or **Ru_2_‐10**. The ^31^P{^1^H} NMR spectra showed a sharp singlet for the *trans*‐disposed P^*i*^Pr_3_ ligands at *δ*=38.46 ppm for **Ru_2_‐3** or 38.84 ppm for **Ru_2_‐7** with ensuing upfield shifts to 36.45 or 36.15 ppm for their coordinatively saturated congeners **Ru_2_‐4** and **Ru_2_‐8**. The ^31^P resonance of **Ru_2_‐10** exhibits a similar high‐field shift to 36.20 ppm as compared to its 16 valence electron precursor **Ru_2_‐9** (δ=38.20 ppm). High‐resolution electrospray‐ionization mass spectra (HR‐ESI MS, see Figures S24 to S31 of the Supporting Information) further confirmed the identity of the complexes by virtue of the base peaks for the molecular ions, which matched perfectly with the calculated masses.

**Figure 2:**
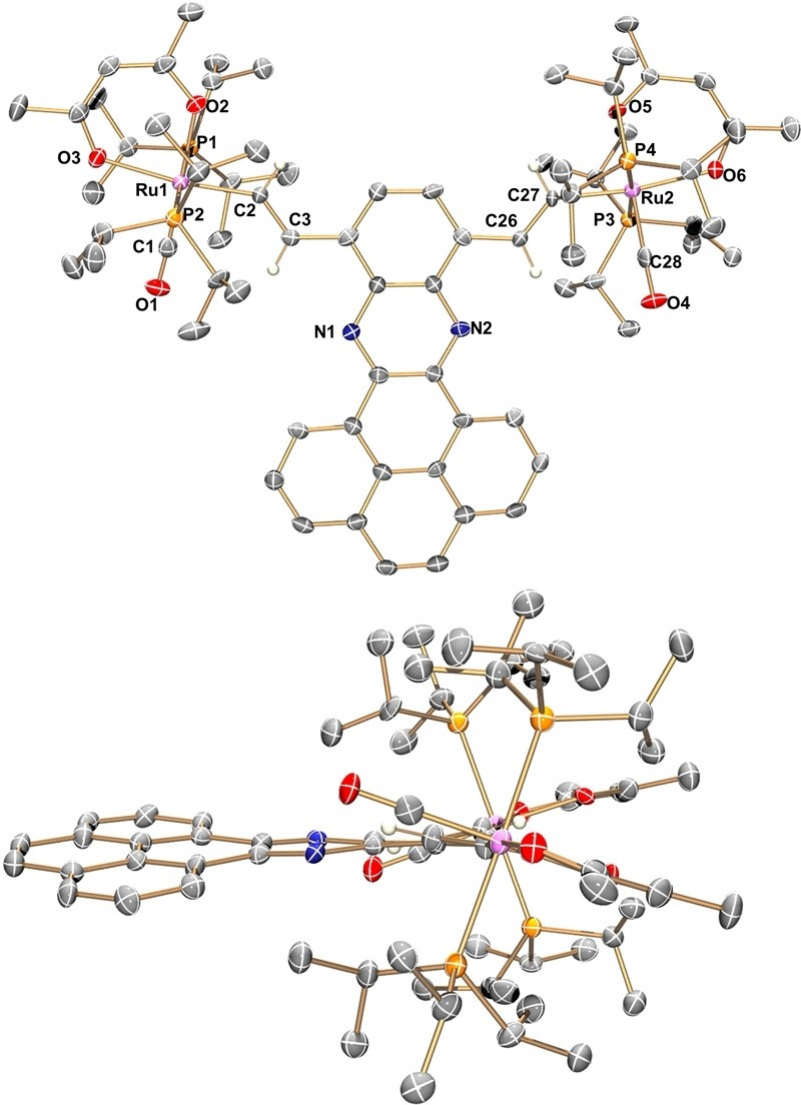
Top and side views of the molecular structure of complex **Ru_2_‐8** in **Ru_2_‐8**⋅4 CH_2_Cl_2_. Solvent molecules and hydrogen atoms except for the alkenyl protons are removed for clarity reasons. Thermal ellipsoids are displayed at the 50 % probability level.

Unequivocal confirmation for the identity of complex **Ru_2_‐8** was obtained by X‐ray diffraction analysis. Single crystals were grown by slow evaporation of solvent from a saturated solution of the complex in dichloromethane. Figure 2 provides two different views of the molecular structure of **Ru_2_‐8**.

Complex **Ru_2_‐8** crystallizes in the triclinic space group *P* $\bar{1}$ . Despite the high symmetry in solution, the two ruthenium alkenyl moieties are crystallographically different and have slightly different bond parameters (see Table S1 of the Supporting Information). Equivalent bond lengths or angles are nevertheless identical within the error margins of the experiment. For example, the Ru−C_alkenyl_ and Ru−C_CO_ bonds measure 2.032(6) or 2.045(6) Å and 1.811(7) or 1.806(7) Å, respectively, while Ru−P bond lengths group in a narrow range of 2.421(2) to 2.429(2) Å. The bond angles P3‐Ru1‐P4 and P1‐Ru2‐P2 of 177.85(6) and 175.27(6)° attest to a near linear arrangement. Due to the strong σ‐*trans*‐influence of the alkenyl ligand, the Ru−O bonds to the acac^−^ oxygen donor atoms *trans* to the alkenyl ligand of 2.192(4) and 2.192(5) Å are appreciably longer than those of 2.122(4) and 2.140(4) Å opposite to the CO ligand. All these bond parameters fall in the ranges previously observed for other crystallographically characterized Ru alkenyl complexes with acac^−^ coligands.[^19d^, ^21a^, ^21b^]

The appended π‐conjugated phenanthroquinoxaline in **Ru_2_‐8** is not fully planar, but is slightly twisted along the N⋅⋅⋅N vector with a maximum torsion N−C−C−N of 8.6° at the adjacent carbon atoms of the divinylphenylene linker (Figure 2). The vinyl ruthenium units are likewise tilted by 11.3° or 13.3° and are oriented towards the more open side of the condensed phenanthroquinoxaline heterocycle. Even larger rotations are observed for the individual {Ru(acac)(CO)(P^*i*^Pr_3_)_2_} subunits so that the P−Ru⋅⋅⋅Ru−P dihedrals assume unusually large values of 38.7° or 39.8°, respectively (Figure 2).

The {Ru} fragments adopt a *cisoid* arrangement with the sterically more demanding acac^−^ ligands oriented to opposite of the condensed PAH heterocycle. In the crystal, individual molecules of **Ru_2_‐8** arrange in sheets that roughly bisect the *ac* plane. Phenanthroquinoxaline ligands of molecules that belong to different sheets are coplanar to each other, but point to different sides. They are separated from each other by the P^*i*^Pr_3_ ligands, which form weak pairwise CH⋅⋅⋅π contacts via methyl protons with carbon atoms of the extended PAH ligand (see Figure S32 of the Supporting Information for a packing diagram and short contacts). Hence, the bulky {Ru(acac)(CO)(P^*i*^Pr_3_)_2_} entities suppress intermolecular π‐stacking of the anellated pyrene rings, which accounts for the increased solubility of the complexes when compared to their alkynylated precursors. At the same time, they leave the entire pyrene constituent exposed to the exterior (for a top view on a space‐filling model see Figure S33 of the Supporting Information).

### Electrochemistry

**Figure 3:**
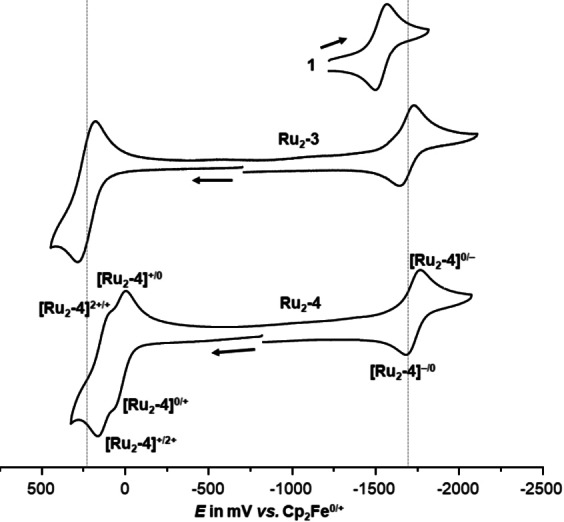
Cyclic voltammograms of **1**, **Ru_2_‐3** and **Ru_2_‐4** (THF/0.1 M NBu_4_^+^PF_6_^−^, 295(±3) K, scan rate *v*=100 mV/s). The dotted grey lines represent the (overall) half‐wave potentials of the oxidations and of the reduction of complex **Ru_2_‐3**; they are intended as a guide to the eye.

The electrochemical properties of the new complexes were investigated by cyclic and square‐wave voltammetry in tetrahydrofuran (THF) and in CH_2_Cl_2_ in the presence of 0.1 M NBu_4_^+^ PF_6_^−^ as the supporting electrolyte. Pertinent data are provided in Table 1, while Figure 3 compares representative voltammograms of complexes **Ru_2_‐3** and **Ru_2_‐4** and their precursor dialkyne **1**. Exemplary CVs of the other complexes can be found as Figures S34 to S45 in the Supporting Information. All complexes exhibit two consecutive one‐electron oxidations as well as one bridge‐centred reduction. While the second oxidation of complex **Ru_2_‐7** in THF and the reduction of complexes **Ru_2_‐3** and **Ru_2_‐7** are chemically only partially reversible, all other redox waves constitute chemically reversible one‐electron redox couples that, apart from slightly larger peak‐potential differences for the reductions, obey the criteria of uncomplicated Nernstian processes. For complex **Ru_2_‐3**, the first and the second oxidation occur at very similar potentials and are therefore merged into a single voltammetric wave or square‐wave peak in THF (Figure 3), while they are clearly resolved into two individual waves with a half‐wave potential splitting Δ*E*_1/2_ of 113 mV in CH_2_Cl_2_ (Figure S37 of the Supporting Information). The two oxidations of complex **Ru_2_‐4** are already resolved into two separate waves in THF with an enhanced splitting in CH_2_Cl_2_ (Table 1, Figure S39). This also holds true for complexes **Ru_2_‐7** and **Ru_2_‐8**, which exhibit substantially larger redox splittings Δ*E*_1/2_ as a consequence of the smaller extension of the π‐conjugated linker and stronger electronic coupling in the one‐electron‐oxidized, mixed‐valent (MV) state (see below). This agrees with the general behaviour of previously reported divinylarylene‐bridged diruthenium complexes.[^19a^, ^19b^, ^19c^, ^19d^, ^19e^, ^19f^, ^19h^, ^22^] The half‐wave potentials for the stepwise oxidations of the acac^−^ complexes **Ru_2_‐4**, **Ru_2_‐8** and **Ru_2_‐10** are by roughly 200 or even 300 mV (**Ru_2_‐10**) (*E*_1/2_^0/+^) or 150 to 225 mV (*E*_1/2_^+/2+^) lower than those of their 16 VE chloro precursors **Ru_2_‐3**, **Ru_2_‐7** and **Ru_2_‐9**, which is due to their increased valence electron count.^[21b]^

**Table 1:** Cyclic voltammetry data of dialkynes **1**, **5** and of complexes **Ru_2_‐3** to **Ru_2_‐10**.^[a]^

	*E*_1/2_^0/+^ (Δ*E*_p_)	*E*_1/2_^+/2+^ (Δ*E*_p_)	Δ*E*_1/2_	*E*_1/2_^0/−^(Δ*E*_p_)
**1**	–	–	–	−1531 (65)
**Ru_2_‐3**	202 (–) [160 (69)]	267 (–) [273 (83)]	65 [113]	−1685 (87) [−1632 (139)]
**Ru_2_‐4**	35 (63) [−67 (66)]	124 (84) [65 (80)]	89 [132]	−1726 (84) [−1652 (99)]
**5**	–	–	–	−1645 (88)
**Ru_2_‐7**	‐68 (111) [‐154 (64)]	77 (104) [78 (67)]	145 [232]	−2085 (175) [−1510 (–)]
**Ru_2_‐8**	−279 (76) [−343 (77)]	−93 (69) [−97 (81)]	186 [246]	−2162 (90) [−2044 (102)]
**Ru_2_‐9^[b]^**	[−75 (–)]	175 (–)]	[250]	n. a.
**Ru_2_‐10**	[−363(66)]	[−50(70)]	[313]	n. a.

[a] All data in millivolts versus Cp_2_Fe^0/+^ in THF/NBu_4_^+^PF_6_^−^ [CH_2_Cl_2_/NBu_4_^+^PF_6_^−^] (0.1 M) at 295(±3) K and at *ν*=100 mV/s; potentials are subject to error margins of ±4 mV. The *E*_1/2_^0/+^ and *E*_1/2_^+/2+^ values of **Ru_2_‐3** in THF were obtained by digital simulation.^[23]^ [b] Data from Ref. [19l].

The ligand‐based reduction of the corresponding azaacene unit is shifted by 154 to 517 mV cathodic (i. e. to more negative potential) when compared to the corresponding TMS‐protected dialkyne. The magnitude of this shift increases in parallel with the electron‐donating capabilities of the appended ruthenium complex moieties and with a smaller extension of the linker (i. e. a closer proximity of the electron‐rich {Ru} complex entities), so that the largest difference is seen for complex **Ru_2_‐8**.

### Electronic spectroscopy and spectroelectrochemistry

**Figure 4:**
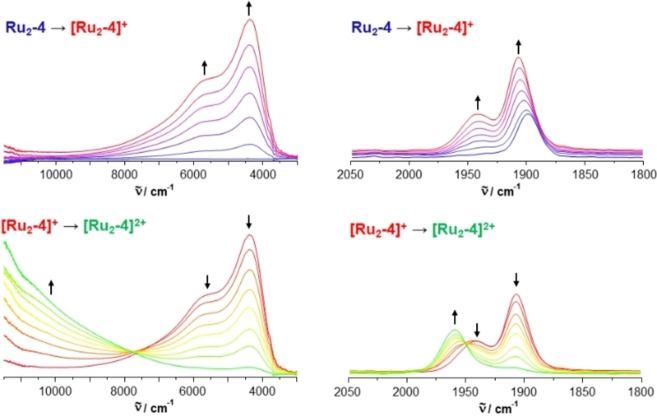
Spectroscopic changes of the Ru(CO) stretching vibrations and in the near infrared in 0.1 M 1,2‐C_2_H_4_Cl_2_/NBu_4_PF_6_ at r.t. during stepwise oxidations of complex **Ru_2_‐4**.

The presence and strength of electronic coupling in the mixed‐valent, one‐electron oxidized forms of these complexes (i. e. the extent of charge and spin delocalization) can be deduced from their spectroscopic signatures in the IR and NIR regions. Owing to the presence of a carbonyl ligand at every ruthenium ion, these complexes possess an indicative IR probe with an inherently high oscillator strength that senses the charge density (and redox‐induced changes thereof) locally at the corresponding metal atom to which it is attached. This is due to the synergistic nature of the M−CO bond and the weakening of metal‐carbonyl back‐bonding concomitant with the depletion of electron density from the metal atom upon oxidation. Oxidation‐induced changes of the IR spectra were monitored in situ using an optically transparent thin‐layer electrolysis (OTTLE) cell according to the design of Hartl et al.^[24]^ This was done by increasing the potential at the working electrode, so to stepwisely oxidize the complexes to first their mixed‐valent (MV) radical cations and then their dications. All spectroelectrochemical experiments were conducted on solutions of the complexes in 1,2‐dichloroethane (1,2‐C_2_H_4_Cl_2_, DCE) with in 0.1 M NBu_4_^+^ PF_6_^−^ as the supporting electrolyte. DCE is a more oxidation‐inert, higher boiling solvent than THF and provides enhanced potential separations of the individual oxidation processes (note that electrochemical potentials in CH_2_Cl_2_ and in DCE are generally very similar). Figure 4 displays the results of such studies on the complex **Ru_2_‐4** as a representative example, while those of all other complexes are depicted as Figures S48–S55 in the Supporting Information. Relevant data are summarized in Table 2.

**Table 2:** Spectroscopic IR/NIR and UV/Vis/NIR data of the complexes **Ru_2_‐3** to **Ru_2_‐10** in their neutral, monocationic and dicationic states.^[a]^

	ν_CO_ (cm^−1^)	λ (nm) (*ϵ_max_* [M^−1^ cm^−1^])
**1**	–	425 (22300), 402 (16300), 340 (32600), 318 (82600), 305 (86000)
**Ru_2_‐3**	1910	582 (7340), 424 (20700), 378 (37100), 320 (44500)
**[Ru_2_‐3]^.+^**	1924, 1957	2065 (9800), 1610 (6600), 506 (10600), 379 (33800), 319 (54000)
**[Ru_2_‐3]^2+^**	1970	927 (10200), 804 (16000), 470 (14800), 401 (25000), 316 (59000)
**Ru_2_‐4**	1899	621 (2100), 405 (56000), 391 (57000), 319 (57000)
**[Ru_2_‐4]^.+^**	1906, 1941	2283 (13600), 1760 (7700), 535 (8600), 404 (39000), 311 (60000)
**[Ru_2_‐4]^2+^**	1958	783 (31500), 462 (14400), 424 (26500), 404 (23600), 313 (84000)
**5**	–	458 (11000), 426 (13000), 349 (14200), 331 (25400), 315 (23200), 305 (29700), 293 (36600)
**Ru_2_‐7**	1912	597 (11200), 400 (26296), 351 (48000)
**[Ru_2_‐7]^.+^**	1934, 1945 (sh)	1220 (7900), 947 (5500), 814 (8800), 602 (20500), 512 (17700), 452 (17000), 350 (33700)
**[Ru_2_‐7]^2+^**	1955	1009 (9800), 564 (13000), 451 (14400), 349 (35000)
**Ru_2_‐8**	1898	620 (7700), 407 (25600), 384 (28000)
**[Ru_2_‐8⋅]^+^**	1934, 1947 (sh)	1304 (3600), 992 (2740), 860 (4600), 521 (8600), 404 (17600)
**[Ru_2_‐8]^2+^**	1959	1003 (2700), 594 (9100), 450 (12000), 406 (13900)
**Ru_2_‐9** ^[b]^	1910	503 (1330), 405 (2630), 353 (10300)
**[Ru_2_‐9]^.+^** ^[b]^	1932, 1942 (sh)	1255 (4110), 585 (4270), 346 (5820)
**[Ru_2_‐9]^2+^** ^[b]^	1991	624 (5360), 430 (3230), 266 (9060)
**Ru_2_‐10**	1896	358 (28862)
**[Ru_2_‐10]^.+^**	1918, 1932	341 (13400), 541 (12700), 591 (17600), 783 (2382), 990 (3000), 1152 (8100), 1364 (15500)
**[Ru_2_‐10]^2+^**	1958	296 (33400), 462 (11300), 630 (12800), 780 (3100)

[a] Measurements were carried out in 0.1 M 1,2‐C_2_H_4_Cl_2_/NBu_4_^+^PF_6_^−^ at r. t. [b] From Ref. [19 l].

One should note here that the modest half‐wave potential splittings of the phenanthrenediyl‐bridged complexes **Ru_2_‐3** and **Ru_2_‐4** imply that the comproportionation equilibria that connect the dications and the neutral complexes with their one‐electron oxidized forms, e. g. **[Ru_2_‐3]^2+^** +**Ru_2_‐3**⇄**2 [Ru_2_‐3]^.+^**, are not completely shifted to the side of the radical cations. In the present case, the associated equilibrium constants *K*_comp_ (Eq. (1); R=universal gas constant and F=Fararaday's constant) dictate that, after stoichiometric release of one electron, i. e. at the point, where the radical cations reach their maximum concentrations, 9 % (**Ru_2_‐3**) or 7 % (**Ru_2_‐4**) of the neutral and the dioxidized forms each are present besides 82 % or 86 % of the one‐electron oxidized forms (see Figure S56 of the Supporting Information for a graphical account). This does however, not affect the following discussion, as the bands of the neutral and the dicationic complexes are sufficiently removed from those of the radical cations and so weak that they do not distort the peak positions of the monooxidized forms to a noticeable extent.

(1) $$K{}_{\mathrm{comp}}=\exp\;\left(R\cdot T\cdot \Delta E{}_{1/2}\right)/F$$

**Figure 5:**
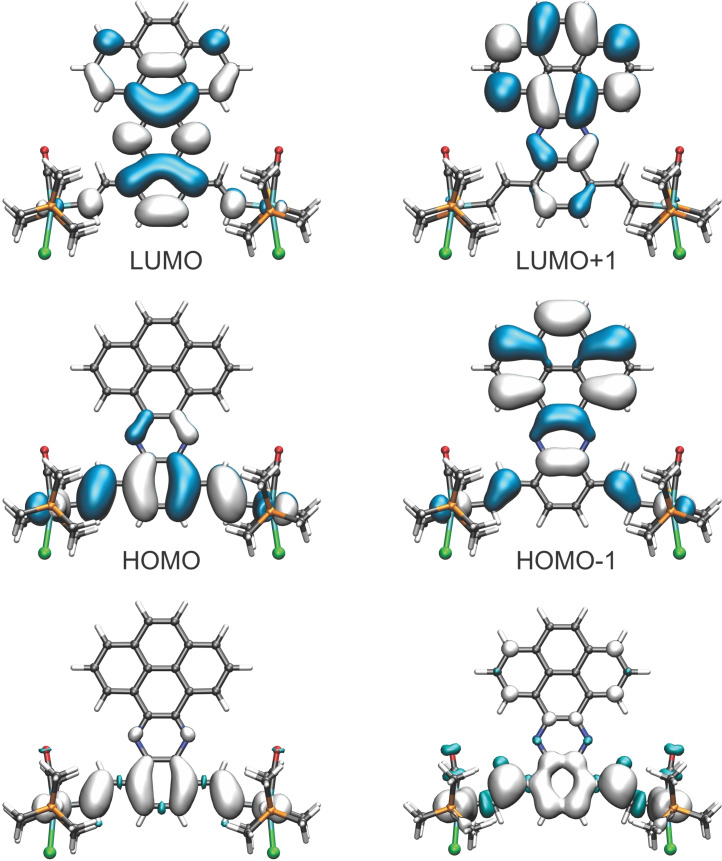
Top and middle row: Contour plots of the calculated HOMO, HOMO‐1, LUMO and LUMO+1 of a simplified PMe_3_ model of complex **Ru_2_‐7** (PBE1PBE/6‐31G(d)PCM (CH_2_Cl−CH_2_Cl). Bottom: Computed spin densities for the radical cation (left) and the dication (right) of the model for complex **Ru_2_‐7**.

**Figure 6:**
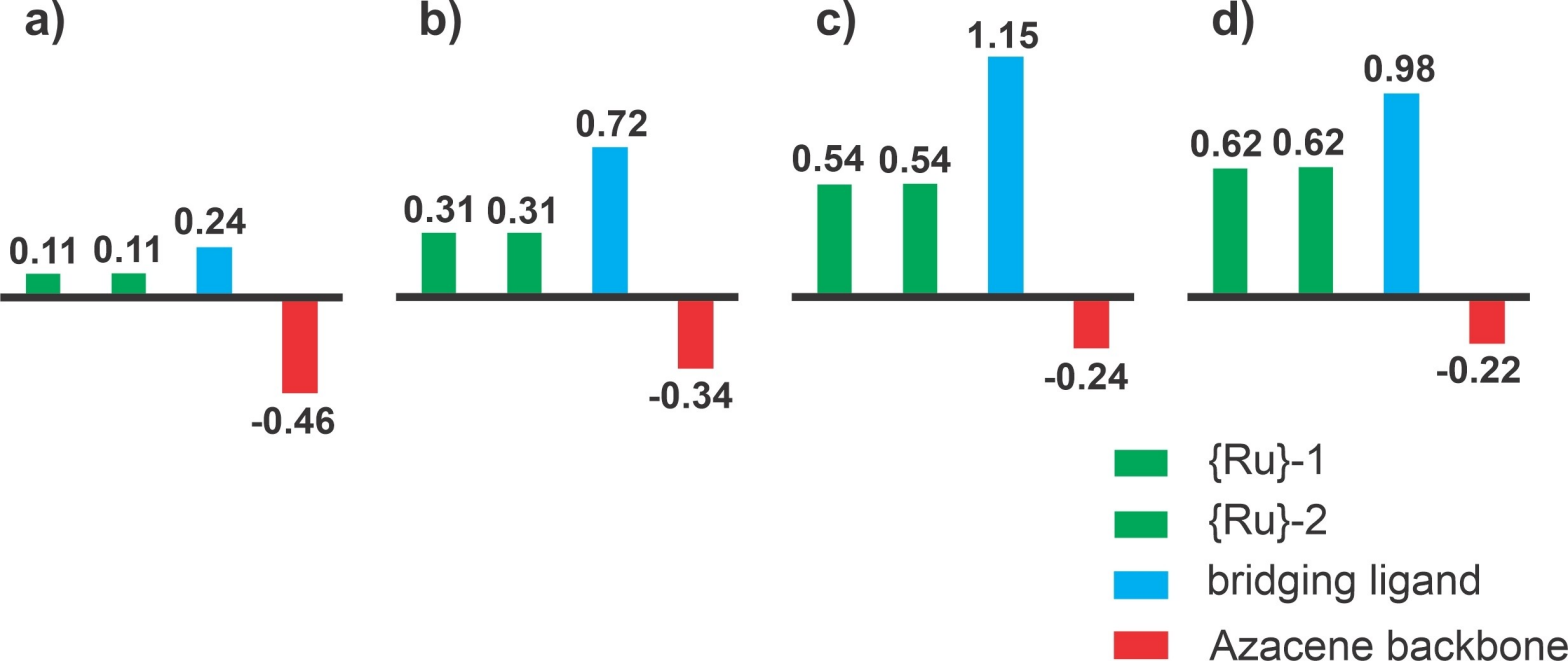
PB1PBE‐computed charge densities on the constituents of a simplified PMe_3_ model of complex **[Ru_2_‐7]^n+^** in it's a) neutral (n=0), b) monocationic (n=1), **c**) dicationic (n=2) (singlet) and **d**) dicationic (triplet) states according to NBO‐analysis. {Ru}=Ru(CO)(Cl)(P^*i*^Pr_3_)_2_.

As a consequence of large ligand contributions to the HOMO of the neutral complexes and the SOMO of their associated radical cations, the overall CO band shift on twofold oxidation falls significantly short to that of ca. 120 cm^−1^ observed for related Ru(CO)_2_(PR_3_)_3_ complexes, where the oxidation is entirely metal‐based.^[25]^ Large bridge contributions to the individual redox processes are also indicated by the computed compositions of the occupied frontier molecular orbitals of the neutral complexes and the SOMO of their associated radical cations. The results of NBO analysis and the computed charges for the individual {Ru} entities, the divinylarylene bridge and the laterally appended azaacene π‐perimeter point into the same direction. Figure 5 and Figure 6 illustrate the results of these calculations on complex **Ru_2_‐7**; graphical displays and tabulated data for **Ru_2_‐3** are provided in Table S2 and S3 and in Figures S60 to S62 of the Supporting Information. We also note distinct changes of bridge‐associated bands in the mid‐IR region (see also Figures S50 and S54 of the Supporting Information for the results on **Ru_2_‐3**).^[19g]^

All neutral diruthenium complexes **Ru_2_‐3**, **Ru_2_‐7** and **Ru_2_‐10** show a single Ru(CO) band at 1910 to 1912 cm^−1^ for the five‐coordinated complexes and at 1896 to 1899 cm^−1^ for their six‐coordinated counterparts. The red‐shift on coordinative saturation is again due to the increase of the electron count from 16 to 18. In their MV states, complexes **Ru_2_‐7** and **Ru_2_‐8** as well as **Ru_2_‐10** with a shorter divinylphenylene‐type linker between the Ru ions show two closely spaced Ru(CO) bands that consist of an intense band at lower energy and a weaker shoulder at ca. 10 to 15 cm^−1^ higher energy (see the left panel in Figure 4). Such a band pattern has also been noted for **[Ru_2_‐9]^.+^**, which has been identified as an intrinsically delocalized MV system of Class III[^19l^, ^20^] according to the Robin‐and‐Day classification scheme.^[26]^ This peculiar band pattern originates from an enhanced splitting between the allowed asymmetric and the nominally forbidden symmetric combinations of the CO stretches of the two Ru(CO) entities and some intensity gain of the latter in the one‐electron oxidized state, when compared to the neutral and dicationic forms.

Detailed studies on related mixed‐valent diethynylphenylene‐bridged diruthenium complexes had shown that complex, multi‐peak patterns of charge‐sensitive IR and of IVCT bands in the NIR may also result from the simultaneous presence of different rotamers, if the latter differ with respect to their electronic coupling strengths.^[27]^ We therefore screened computationally three individual rotamers of a truncated PMe_3_ model of complex **[Ru_2_‐7]^.+^** that differ with respect to the mutual orientations of the alkenyl ruthenium entities (*transoid (trans)*, *cisoid* (*cis*) or *cis/trans* (unlabelled in the corresponding Figures and Tables) with respect to the PAH bridging ligand). These studies revealed hardly any differences in the computed CO band patterns and energies of the CO stretches for the two most favourable *cisoid* or *cis/trans* conformers or of the electronic absorption bands between them (see Table S4 of the Supporting Information). Atomic displacements during the two combinations of Ru(CO) stretches and the computed Ru(CO) band patterns for the three rotamers are shown in a Power‐Point animation, which is deposited as an additional Supporting Information (view in the presentation mode to see the animation). This confirms that the peculiar shape of the Ru(CO) band in the one‐electron oxidized forms of the quinoxalinyl‐bridged complexes **[Ru_2_‐7]^.+^** and **[Ru_2_‐8]^.+^** or in phenylene‐bridged **[Ru_2_‐9]^.+^** and **[Ru_2_‐10]^.+^** are very likely not due to the presence of different rotamers or electronic imbalances between the bridged {Ru} sites.

The neutral penta‐ and hexacoordinated complexes **Ru_2_‐3** and **Ru_2_‐4** with the more extended 2,7‐phenanthrenediyl linker show a different behaviour. Upon oxidation to their corresponding radical cations, the initial CO band gradually evolves into a pattern of two distinct, similarly intense Ru(CO) bands at 1924 cm^−1^ and 1957 cm^−1^, or 1906 cm^−1^ and 1941 cm^−1^, which are both displaced to higher energies when compared to the neutral state. They again merge into a single, further blue‐shifted band at 1970 cm^−1^ or at 1955 cm^−1^ as the second oxidation to the corresponding dications proceeds. The appearance of two similarly intense, widely separated CO bands (Δν_CO_=33 or 35 cm^−1^, respectively) in the one‐electron‐oxidized complexes **[Ru_2_‐3]^.+^** and [**Ru_2_‐4]^.+^** reveals that, in these cases, the unipositive charge is unequally distributed across the two molecular halves, so that they represent mixed‐valent compounds of Class II with an only partial charge delocalization.

For such type of carbonyl ligand‐bearing MV complexes, Geiger and coworkers have established the charge‐distribution parameter Δ*ρ* as a quantitative measure of electron delocalization. This parameter is based on the relative shifts of the CO bands of a mixed‐valent carbonyl complex with respect to the ones for the isovalent bordering dioxidized and neutral states. It is defined according to Equation (2).^[28]^ In Equation (2), *ν’*_ox_ and *ν’*_red_ denote the energies of the CO stretching vibrations of the dioxidized and the neutral forms, while Δ*ν*_ox_ and Δ*ν*_red_ are the energy differences between the band positions for the dioxidized species and the higher‐energy band of the monooxidized form (Δ*ν*_ox_), or between the lower‐energy band of the monooxidized form and the neutral complex (Δ*ν*_red_), respectively. By this definition, Δ*ρ* scales between 0.00 for a completely localized MV system of Class I and 0.50 in the case of complete charge delocalization. By applying Equation (2) to MV complexes **[Ru_2_‐3]^.+^** and [**Ru_2_‐4]^.+^**, we obtain Δ*ρ* values of 0.23 or 0.20, which identifies them as Class II systems with ca. 25 %/75 % to 20 %/80 % of the unipositive charge on each of the two molecule halves. This resembles the situation encountered for MV diruthenium divinylarylene complexes with linkers of similar π‐extension like tetraphenylethene, *E*‐stilbene, 2,2’‐bipyridine, or carbazole.[^19a^, ^19b^, ^19c^, ^19g^, ^19k^]

(2) $$\Delta \rho =\left(\Delta \nu {}_{\mathrm{ox}}+\Delta \nu {}_{\mathrm{red}}\right)/2\;\left[\nu {}^{'}{}_{\mathrm{ox}}-\nu {}^{'}{}_{\mathrm{red}}\right]$$

**Figure 7:**
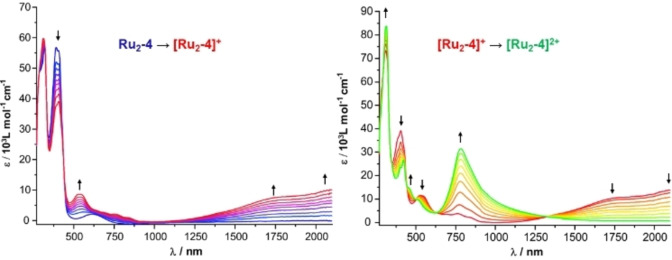
Spectroscopic changes in UV/Vis/NIR absorption spectra during oxidation of neutral **Ru_2_‐4** to **[Ru_2_‐4]^.+^** (left) and dicationic **[Ru_2_‐4]^2+^** (right).

Profound spectroscopic changes upon oxidation are also seen in the ultraviolet, visible and near‐infrared (UV/Vis/NIR) regions of the electronic spectra. This is exemplarily shown in Figure 7 for complex **Ru_2_‐4**; for graphical representations of the spectroscopic changes during oxidation of the other complexes see Figures S49 to S55 of the Supporting Information). All complexes exhibit highly intense π→π* absorptions of the organic bridging ligand that resemble those of the parent dialkynes (Figures S46 and S47 of the Supporting Information) and are only mildly influenced by the attached complex entities and their valence electron counts. Most neutral complexes feature an additional band near 600 nm for the 16 VE or near 620 nm for the 18 VE complexes, which has no counterpart in related diruthenium complexes lacking the electron‐accepting azaacene part of the divinylarylene bridging ligand, including **Ru_2_‐10**. Such a band is also present in the free alkynes, albeit at higher energies. We therefore assign it as a charge‐transfer band with an ensuing shift of electron density from the electron‐rich diruthenium‐divinylphenylene or ‐phenanthrenylene part of the molecule to the azaacene segment, i. e. as mixed intraligand/metal‐to‐ligand CT (ILCT/MLCT).

This is also supported by the results of our TD‐DFT calculations, which identify this transition as the HOMO→LUMO excitation. Figure 4 and Figure S57 to S59 of the Supporting Information provide contour diagrams of the frontier MOs of truncated PMe_3_ models of the real complexes **Ru_2_‐3** and **Ru_2_‐7** and demonstrate the stronger bias of the LUMO towards the fused diimine part of the bridging ligand.

NBO‐derived fragment contributions, charges and spin densities are compared in Figure 6 and Figure S57 to S62 of the Supporting Information with pertinent values listed in Tables S2 of the Supporting Information. Figures S63 to S73 of the Supporting Information provide TD‐DFT‐computed spectra and associated electron density difference plots for the most important electronic transitions of complexes **[Ru_2_‐3]^n+^** and **[Ru_2_‐7]^n+^** in all relevant oxidation states (n=−1, 0, +1, +2 (singlet and triplet states) as computed by the pbe1pbe functional. Those obtained with the BLYP35 functional^[29]^ are collected in Figures S75 to S81 and in Table S3 for comparison (see below).

The ILCT/MLCT band is still retained in the one‐electron‐oxidized MV radical cations (Figure 7; see also Figures S64–S66, S70–S72, S75 to S77 and S79 to S81 of the Supporting Information), mostly even with enhanced intensity, but is shifted to somewhat higher energy as compared to the neutral complexes. The most characteristic asset of all mixed‐valent forms is, however, a rather intense intervalence charge absorption in the near infrared (NIR). This band is associated with charge transfer between the differently charged redox sites in mixed‐valent systems of Class II, while this is not the case for an intrinisically delocalized MV system of Class III.^[30]^ Its specific appearance in the one‐electron oxidized states makes it a convenient marker for assessing the point at which formation of the radical cation has gone to completion or where the maximum concentrations of this species are present and where the second oxidation commences.

In the phenanthrenediyl‐bridged complexes **[Ru_2_‐3]^.+^** and [**Ru_2_‐4]^.+^**, the IVCT band is vibrationally structured with three discernible peaks and the maximum intensity for the most red‐shifted one. The latter peak lies outside the accessible range of our Vis/NIR spectrometer and is hence best observed at the high energy side of IR/NIR spectra, where it is located at 4840 cm^−1^ (2065 nm) or 4380 cm^−1^ (2280 nm), respectively. Peak splittings are in the range of ca. 1275 cm^−1^ and likely correspond to vibrational modes of the PAH backbone. An assignment to several coexisting conformers that differ with respect to the orientations of the alkenyl ruthenium pendents is less likely, as our TD‐DFT calculations predict much smaller energy differences for the corresponding bands of different conformers than are experimentally observed. In contrast, the IVCT band of **[Ru_2_‐7]^.+^** and [**Ru_2_‐8]^.+^** provides only a single peak.

Calculations on the truncated PMe_3_ model of **[Ru_2_‐3]^.+^** with the pbe1pbe functional failed to produce unequal charge distributions over the two molecular halves. Such oversymmetrization is a known shortcoming of pure DFT routines,[^27a^, ^31^] which may be cured by an appropriate admixture of Hartree‐Fock exact exchange. The BLYP35 functional introduced by Kaupp et al. is particularly successful in this respect while not compromising intrinsically delocalized MV system of Class III.[^29^, ^32^] It has been applied to adequately model valence distributions in metal–organic and organic MV compounds.^[33]^ This functional provided indeed a more localized electronic structure for **[Ru_2_‐3]^.+^** and reproduced the experimentally observed splitting and intensity distribution of the Ru(CO) bands very well (Δ$\tilde{\nu }$ =28 or 29 cm^−1^ as compared to the experimental value of 33 cm^−1^, see Table S4 of the Supporting Information). Consistent with these results, the TD‐DFT computed electron density difference map for the NIR band shows a distinct flow of electron density from the less to the more oxidized half of the molecule (see Figures S75 to S77 of the Supporting Information). Gratifyingly, **[Ru_2_‐7]^.+^** remained delocalized with an only small computed splitting of the Ru(CO) bands of 5 to 7 cm^−1^ and identical intensity distributions and band origins for the individual rotamers as obtained by pbe1pbe (see above). Figures S82 and S83 of the Supporting Information provide the computed IR spectra in the Ru(CO) region and demonstrate the excellent match of the BLYP35 computed spectra with the experiment.

The second oxidation is accompanied by a bleach of this structured, low‐energy NIR band and the parallel increase of a new band of similar intensity at higher energy, yet still peaking in the NIR or the NIR/Vis border regime. Our DFT calculations indicate that this band has mixed intraligand/metal‐ligand charge‐transfer character with a flow of electron density from the condensed benzoquinoxaline or pyrene part or the Ru ions and the chloride ligands to the central divinylarylene part of the bridging ligand (see Figures S67, S68 and S73 of the Supporting Information). As per the higher degree of anellation of the PAH segment, the corresponding absorption is shifted to lower energies in **[Ru_2_‐7]^2+^** and **[Ru_2_‐8]^2+^** as compared to complexes **[Ru_2_‐3]^2+^** and **[Ru_2_‐4]^2+^** (Table 2).

### EPR spectroscopy

**Figure 8:**
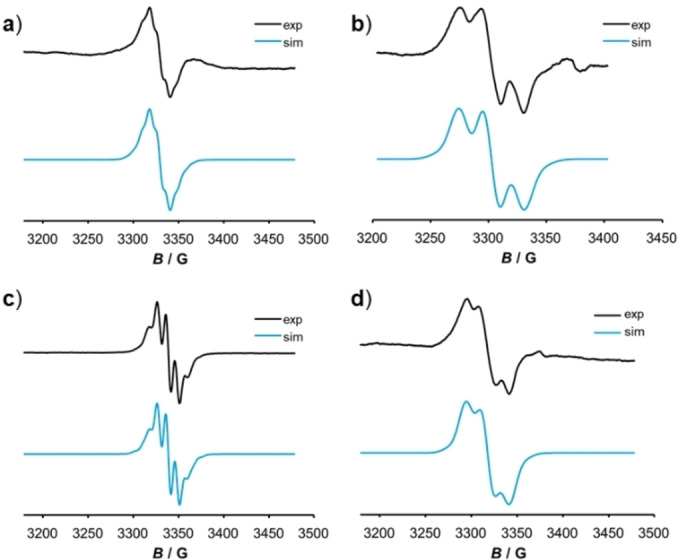
Experimentally observed (black line, top curve) and simulated (blue line, bottom curve) EPR spectra of **Ru_2_‐3** (**a**, **b**) and **Ru_2_‐7** (**c**, **d**) after the first (left, a) and c)) and after the second oxidation (right, b) and d)), respectively.

EPR spectroscopy is a good analytical tool to probe the delocalization of the unpaired spin density in the MV radical cations on a time scale of 10^−8^ to 10^−9^ s, which is roughly three orders of magnitude slower than the one of 10^−11^ to 10^−12^ s of IR spectroscopy. Samples of the one‐electron oxidized radical cations were generated by the addition of 1 equiv. of ferrocenium hexafluorophosphate or acetylferrocenium hexafluoroantimonate to the corresponding neutral complexes, while the dications of the complexes were prepared by their reaction with 2.2 equiv. of acetylferrocenium hexafluoroantimonate. EPR spectra of complexes **[Ru_2_‐3]^.+^** and **[Ru_2_‐7]^.+^** are depicted as Figure 8 (see Figures S84‐S93 of the Supporting Information for EPR spectra of the other complexes). As per usual, only the radical cations of the five‐coordinated complexes **Ru_2_‐3** and **Ru_2_‐7** provided spectra with resolved hyperfine splittings (hfs's) to the phosphorus and the ^99/101^Ru nuclei. In both cases, the spectra of the radical cations were adequately reproduced by assuming hfs to four identical ^31^P and two identical ^99/101^Ru nuclei (Table 3). This signals valence averaging of **[Ru_2_‐7]^.+^** on the slower EPR timescale, i. e. a symmetric spin density distribution over both molecular halves, as opposed to the partial valence trapping (i. e. an asymmetric charge distribution) on the faster IR timescale. The rate of intramolecular electron transfer falls thus in between these two time domains. An identical situation was encountered for the one‐electron oxidized forms of other divinylarylene‐bridged diruthenium complexes with π‐extended bridging ligands and similar values of the charge‐distribution parameter Δ*ρ*.[^19a^, ^19b^, ^19c^, ^19g^, ^19k^] From the spectra of the hexacoordinated complexes, only the hfs constant to the Ru nuclei can be extracted, as those to the P atoms remain unresolved. The corresponding data are provided in Table 3.

**Table 3:** EPR data of all four new complexes **Ru_2_‐3** to **Ru_2_‐10** in their monocationic and dicationic states.^[a]^

	*g_iso_*	hfc (*A*)		
		^1^H	^31^P	^99/101^Ru
**[Ru_2_‐3]^+^**	2.018	–	10.6 (4P)	9.6 (2 Ru)
**[Ru_2_‐3]^2+^**	2.034	2.9 (1 H)	27.9 (2 P)	12 (1 Ru)
**[Ru_2_‐4]^+^**	2.020		–	5.7 (2 Ru)
**[Ru_2_‐4]^2+^**	2.030		–	17.1 (1 Ru)
**[Ru_2_‐7]^+^**	2.010		12.5 (4 P)	8.7 (2 Ru)
**[Ru_2_‐7]^2+^**	2.020		22.6 (2 P)	12.0 (1 Ru)
**[Ru_2_‐8]^+^**	2.013		–	7.7 (2 Ru)
**[Ru_2_‐8]^2+^**	2.032		–	12.5 (1 Ru)
**[Ru_2_‐10]^+^**	2.012		–	6.2 (2 Ru)
**[Ru_2_‐10]^2+^**	2.025		–	8.9 (1 Ru)

[a] All hyperfine coupling (hfc) constants are reported in Gauss. The monocations were generated with ferrocenium(III) hexafluorophosphate and the dications were generated with acetylferrocenium(III) hexafluoroantimonate.

**Scheme 2:**
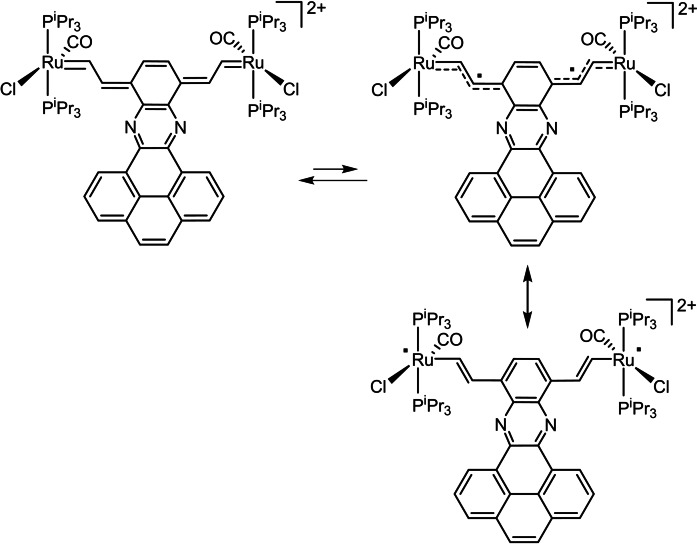
Electronic and resonance structures of dication **[Ru_2_‐7]^2+^**.

Samples of the dications are also EPR active, yet with distinctly smaller signal intensities than those for equally concentrated samples of the radical cations. The EPR resonances of complexes **[Ru_2_‐3]^2+^** and **[Ru_2_‐7]^2+^** with five‐coordinated Ru ions again provide resolved hfs's, but this time to only two P atoms and with roughly doubled hfs constants, in concert with one unpaired spin per molecule half (Figure 8). Paramagnetism of dication samples of such complexes arising from either triplet ground states or from thermally accessible diradical states have been observed on earlier occasions and are tokens of the high ability of the vinylruthenium entities to stabilize unpaired spin density by delocalizing it onto the periphery.[^19a^, ^34^] This may allow the arylene linker to retain its aromaticity as an alternative to closed‐shell quinoidal structures as shown exemplarily for **[Ru_2_‐7]^2+^** in Scheme 2. The *T*‐dependence of the EPR intensities of dications **[Ru_2_‐4]^2+^** on the one hand and **[Ru_2_‐7]^2+^** and **[Ru_2_‐8]^2+^** on the other however differs. While the EPR signal intensity of phenanthrenediyl‐bridged **[Ru_2_‐4]^2^** ^+^ increases as *T* is lowered, those of **[Ru_2_‐7]^2+^** and **[Ru_2_‐8]^2+^** reversibly decrease on cooling. This suggests that in **[Ru_2_‐7]^2+^** and **[Ru_2_‐8]^2+^** the paramagnetic state is populated by thermal excitation, whereas it constitutes the ground state in **[Ru_2_‐4]^2^** ^+^.

Experimental observations are matched by the results of our quantum chemical calculations, which place the triplet state of the phenanthrenediyl‐bridged complex by a rather small margin of ca. 8 kJ/mol below the singlet ground state, while that of **[Ru_2_‐7]^2+^** is ca. 60 kJ/mol above the singlet state. In both cases, the singlet state is by an insignificantly small margin of ca. 2 kJ/mol below the open‐shell singlet.

### Formation of charge‐transfer salts with F_4_TCNQ

Since the hexacoordinated acac^−^ derivatives **Ru_2_‐4** and **Ru_2_‐8** possess lower oxidation potentials and, because of their coordinative saturation, are expected to form chemically more robust radical cations and dications, they were selected for the synthesis of charge‐transfer salts with F_4_TCNQ as the electron acceptor. Comparison with pristine F_4_TCNQ (E_1/2_^0/−^=153 mV, E_1/2_^−/2−^=−484 mV under our conditions) suggest that a redox process with complete electron transfer, that is formation of a radical cation/radical anion pair, is exergonic for all acac^−^ complexes. When equimolar amounts of pale yellow F_4_TCNQ and green diruthenium complexes **Ru_2_‐4** and **Ru_2_‐8** were dissolved in CH_2_Cl_2_ and these solutions were combined, a rapid colour change to a more intense olive green ensued. In the case of **Ru_2_‐4**, this reaction proceeded through a short‐lived, deep purple intermediate, which is the typical colour of the isolated **[Ru_2_‐4]^.+^** radical cation.

**Figure 9:**
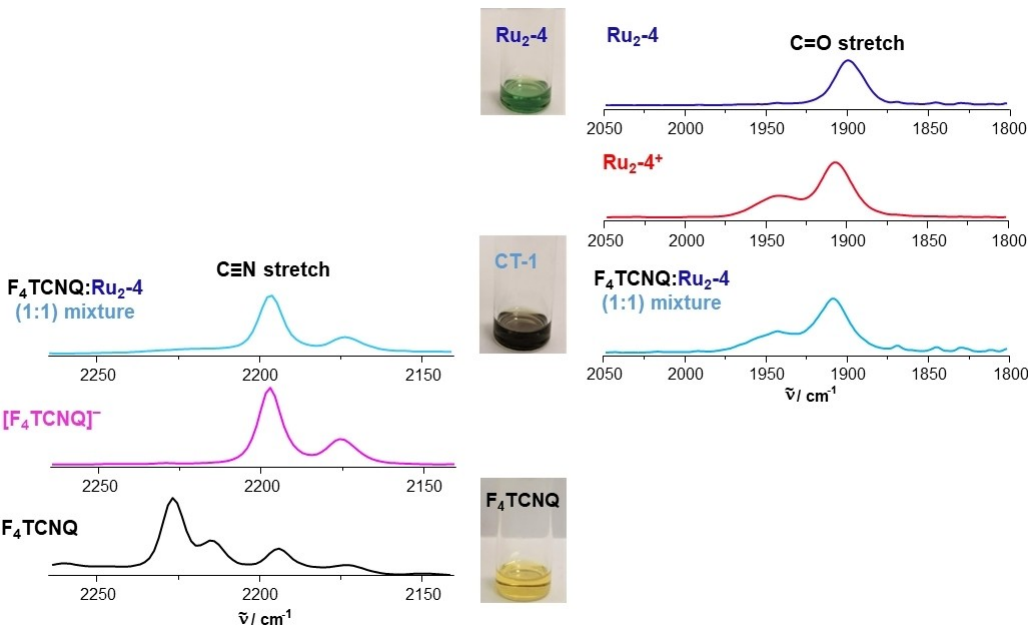
Monitoring the formation of charge‐transfer salt **CT‐1** with IR spectroscopy. The blue‐shift of the CO stretch of the ruthenium complex due to its oxidation as well as the red‐sift of CN stretch of the F_4_TCNQ due to its reduction are clearly observed. Colour images of the corresponding neutral precursors and charge‐transfer salt **CT‐1** are shown in the middle panel.

**Figure 10:**
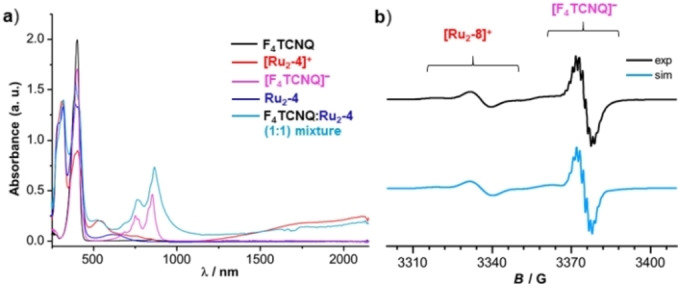
a) Comparison of the UV/Vis/NIR spectrum of salt **CT‐1** with that of the neutral complex **Ru_2_‐4**, neutral F_4_TCNQ, mono‐oxidized complex **[Ru_2_‐4]^+^** and reduced F_4_TCNQ^.−^ for comparison purposes. b) EPR spectrum of salt **CT‐2** synthesized from **Ru_2_‐8** and F_4_TCNQ. Two separate signals for the radical cation and radical anion are clearly observed. The experimental spectrum is shown at the top and simulated one is at the bottom.

Spectroscopic characterization of 1 : 1 mixtures of **Ru_2_‐4** or **Ru_2_‐8** and F_4_TCNQ, henceforth denoted as charge‐transfer salts **CT‐1** and **CT‐2**, provided the genuine spectroscopic fingerprints of the radical cations **[Ru_2_‐4]^.+^** or **[Ru_2_‐8]^.+^**, respectively, as evidenced by their characteristic Vis/NIR and Ru(CO) bands. Moreover, the Vis/NIR and IR spectra also featured the structured NIR absorptions at 769 and 864 nm as well as the C≡N stretching vibration at 2194 cm^−1^ of the F_4_TCNQ^•−^ anion, the latter being red‐shifted by 32 cm^−1^ from the C≡N stretch of neutral F_4_TCNQ.[^16c^, ^17^, ^18d^, ^35^] These results are shown in Figures 9 and 10 and in Figures S95 to S99 of the Supporting Information. Figures 9 and S97 of the Supporting Information also show the visual colour impressions of the sample solutions.

**Figure 11:**
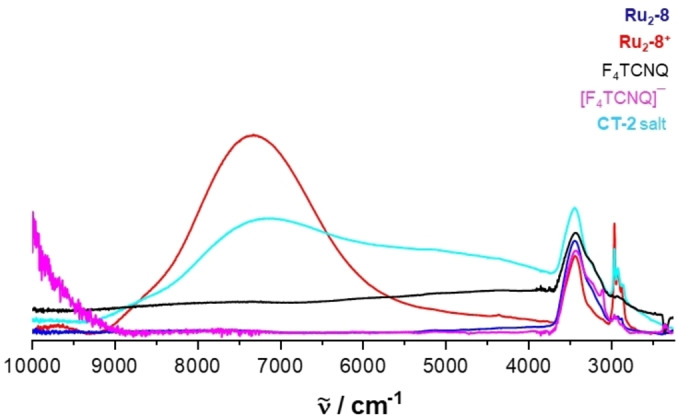
Comparison of the solid‐state NIR spectrum (KBr pellet) of **CT‐2** with those of its neutral components **Ru_2_‐8**, the organic electron‐acceptor F_4_TCNQ, the spectra of the PF_6_^−^ salt of **[Ru_2_‐8]^.+^** as well as that of the cobaltocenium salt of the radical anion F_4_TCNQ^.−^.+

Similarly to the solution spectra, the solid state Vis/NIR absorption spectra are also indicative to the formation of charge‐transfer salts. The comparison of the spectra of solid samples of compound **CT‐2** with those of their corresponding neutral complex precursor and F_4_TCNQ in Figure 11 identifies a new band at 863 nm, which is typical of the F_4_TCNQ^.−^ anion.^[32]^ The IVCT band of [**Ru_2_‐8**]**⋅** ^+^ was observed as a separate peak at 1008 nm. Moreover, solution EPR spectra of the salt **CT‐2** showed the simultaneous presence of the EPR resonances of both its paramagnetic constituents, radical cation [**Ru_2_‐8**]**⋅** ^+^ (*g*=2.013) and F_4_TCNQ^.−^ (*g*=1.9888), the latter with characteristic hyperfine splittings to four nitrogen and four fluorine atoms of 1.4 G and 1.8 G, respectively (Figure 10b). Curiously, solution and powder EPR spectra of **CT‐1** provide an only very weak, broadened signal of the **[Ru_2_‐4]^.+^** cation along with the much more intense one of the TCNQ^.−^ anion (see Figures S100 and S101 in the Supporting Information).

Complex **Ru_2_‐10**, the simple divinylphenylene‐bridged analog of **Ru_2_‐8**, also yielded an intense purple‐brownish solution of the 1 : 1 salt **CT‐3**, comprising of the associated radical cation **[Ru_2_‐10]^.+^** and the F_4_TCNQ^•−^ anion as shown by its IR/NIR, UV/Vis/NIR and EPR spectra (see Figures S102 and S103 of the Supporting Information). At odds with the stable salts **CT‐1** and **CT‐2**, **CT‐3** decomposed within only 15 min. Sample degradation is accompanied with an almost complete loss of the Ru(CO) label (see Figure S104 of the Supporting Information). This signals that the lateral expansion of the bridging ligand and the enhanced delocalization of the unpaired spin density aid considerably in chemically stabilizing CT salts formed from this kind of metal–organic electron donors.

Until now, all our efforts as to obtain single crystals of any of these salts and so to probe for interactions between the radical cations and the radical anions in the solid state failed. The comparison of the UV/Vis/NIR and IR spectra of the salts with those of their individual constituents provided nevertheless some hints as to the existence of some interactions between these ions. One comes from the tailing of the NIR band of F_4_TCNQ^•−^ towards the low‐energy side, which is observed in fluid solution (see Figure 10) and in the solid state. In salt **CT‐2**, this even leads to a separate band at 5070 cm^−1^ (1970 nm) in addition to that of the PF_6_^−^ salt of **Ru_2_‐8^.+^** at ca. 7200 cm^−1^ (1400 nm, Figure 11).

Since the first example of an electrically conductive CT salt was reported in 1973,^[11a]^ their electrical properties have been extensively studied.[^1a^, ^1b^, ^2b^, ^36^] It has been established that the conductivities of a CT salt depend on many factors such as the packing motifs of the donor and acceptor molecules in the solid (crystalline) state, the extent of π‐overlap between the individual constituents, and the extent of charge‐transfer between the donor and the acceptor components.[^1a^, ^1b^, ^2b^, ^37^] We therefore also explored the conductive properties of the present CT‐salts. To these ends, solid powdered samples of **CT‐1** and **CT‐2** were isolated by evaporation of the CH_2_Cl_2_ solvent from their solutions and compressed into pellets. These pellets were placed on a gold plate and contacted with two nanoprobes that served as cathode and anode. After applying a maximum gate voltage of 20 V, no significant current flow was observed, even at the closest distance of 10 μm (Figures S105 and S106 of the Supporting Information). Possible reasons behind the non‐conductivity of **CT‐1** and **CT‐2** are the full separation of charge, i. e. the coexistence of radical cations and anions rather than an only partial charge transfer and the formation of mixed, alternating anion/cation stacks with large separations between neighbouring anion/cation pairs.[^36^, ^38^] Scanning electron microscopy (SEM) on the pelleted samples also revealed that they are composed of small, porous grains (see Figure S107 of the Supporting Information). This may provide large barriers for charge‐transport due to contact resistance at grain boundaries. However, such pellets have often provided measurable responses with conductance values in the order of 10^−4^ Ω^−1^ cm^−1^ for intrinsically conductive CT‐salts, despite their polycrystallinity and disorder.[^8l^, ^36^, ^38b^]

Exploratory variation of the stoichiometric ratios of the complex donor and the F_4_TCNQ acceptor components showed that, in the presence of a larger number of equivalents of F_4_TCNQ, CT‐salts featuring complex dications can also be obtained. As is shown in Figures S108 and S109 of the Supporting Information, the Vis/NIR/IR spectra of solutions that were obtained by combining **Ru_2_‐8** and F_4_TCNQ in a molar ratio of 1 : 2 are indicative of the presence of the **[Ru_2_‐8]^2+^** ion. Unfortunately, this compound decomposes within only few minutes, which precludes its further characterization.

## Conclusion

We have synthesized four diruthenium complexes with two different divinylarylene bridging ligands with laterally extended polycyclic aromatic hydrocarbyl (PAH) bridging ligands. The electrochemical and spectroscopic properties of the complexes in all their accessible oxidation states were studied by voltammetric methods as well as by IR/NIR, UV/Vis/NIR and, where applicable, by EPR spectroscopy. These studies identified the cations **[Ru_2_‐7]^.+^** and **[Ru_2_‐8]^.+^** with the shorter divinylphenylene‐type linker as intrinsically delocalized mixed‐valent systems of Class III according to Robin and Day,^[26]^ while phenanthrenylene‐bridged **[Ru_2_‐3]^.+^** and **[Ru_2_‐4]^.+^** are Class II systems with a ca. 80 : 20 charge distribution over the two molecular halves. Valence averaging however ensues on the slower EPR timescale. Taking advantage of their lower oxidation potentials, complexes **Ru_2_‐4** and **Ru_2_‐8** with hexacoordinated ruthenium entities were utilized for the synthesis of charge‐transfer salts. Defined radical‐cation/radical‐anion salts **CT‐1** and **CT‐2** were obtained from combining equimolar amounts of the ruthenium complexes and the strong organic acceptor F_4_TCNQ. Their spectroscopic characterization confirms full charge separation between the donor and the acceptor constituents of these salts. This study therefore demonstrates that diruthenium bis(vinylarylene) complexes with enhanced lateral π‐extension of the bridging ligand can be employed as the electron donors for the formation of CT salts, just like electron‐rich organic polycyclic aromatic hydrocarbons. Enlarging the π‐surface of the bridging ligands enhances the stability of the resulting CT salts considerably when compared with the CT salt **CT‐3** derived from the simpler divinylphenylene‐bridged analog **Ru_2_‐10**. We can presently only speculate about the roles played by π‐stacking or other attractive interactions between individual donor or between donor/acceptor pairs of molecules for their chemical stabilization, but note some differences in the NIR spectra between salts **CT‐1** and **CT‐2** and their isolated constituents. This and the properties of compounds derived from these and similar divinylarylene‐bridged diruthenium complexes with less powerful organic electron acceptors are the subject of ongoing work in our laboratories.

## Experimental Section

Detailed information as to the employed instrumentation, the analytical and computational methods, as well as the experimental details and characterization data for the ligands and the complexes (NMR, HR ESI‐MS) are provided in the Supporting Information. Also included are the spectroelectrochemical data of IR, UV/Vis/NIR and EPR studies for **Ru_2_‐3**, **Ru_2_‐4**, **Ru_2_‐7**, **Ru_2_‐8** and **Ru_2_‐10** as well as the spectra pertinent to the characterization of CT‐salts **CT‐1**, **CT‐2** and **CT‐3** and the results of quantum chemical calculations with NBO charges, TD‐DFT computed absorption spectra and electron density difference maps for the most important absorption bands, and tabulated energies of the Ru(CO) stretches.

Deposition number contains the supplementary crystallographic data for this paper. These data are provided free of charge by the joint Cambridge Crystallographic Data Centre and Fachinformationszentrum Karlsruhe Access Structures service.

## Conflict of interest

The authors declare no conflict of interest.

1

## Supporting information

As a service to our authors and readers, this journal provides supporting information supplied by the authors. Such materials are peer reviewed and may be re‐organized for online delivery, but are not copy‐edited or typeset. Technical support issues arising from supporting information (other than missing files) should be addressed to the authors.

Supporting Information

Supporting Information

## Data Availability

The data that support the findings of this study are available in the supplementary material of this article.
