# Supplementary material for: Electron‐Rich Diruthenium Complexes with π‐Extended Alkenyl Ligands and Their F4TCNQ Charge‐Transfer Salts
Source: Chemistry. 2022 Mar 18;28(23):e202104403. doi: 10.1002/chem.202104403 (PMC9310581; doi:10.1002/chem.202104403)
Supplement: Supplementary file 1 — Supporting Information [file CHEM-28-0-s001.pdf]

# Chemistry–A European Journal

Supporting Information


## Electron-Rich Diruthenium Complexes with π-Extended Alkenyl Ligands and Their $F_4$TCNQ Charge-Transfer Salts


Rajorshi Das,* Michael Linseis, Stefan M. Schupp, Lukas Schmidt-Mende, and Rainer F. Winter*

## Supporting Information

**Experimental Methods and Materials.** All manipulations were performed under an atmosphere of purified nitrogen with dry, distilled, and nitrogen-saturated solvents. All reagents were purchased from commercial sources and were used without further purification. $^{1}H$ NMR (400 MHz), $^{19}F$ NMR (376 MHz), and $^{31}P$ NMR (162 MHz) spectra were recorded on a Bruker Avance III 400 spectrometer; $^{1}H$ (600 MHz), $^{13}C$ (151 MHz), and $^{31}P$ (243 MHz) NMR spectra were recorded on a Bruker Avance III 600 spectrometer. $^{1}H$ NMR (800 MHz) and $^{13}C$ (202 MHz) NMR spectra were recorded on a Bruker Avance Neo 800 spectrometer. 2,7-Dibromophenanthrene-9,10-dione was purchased from commercially available source. 2,1,3-Benzothiadiazole **10**,[1] 4,7-dibromo-2,1,3-benzothiadiazole **11**,[1] 3,6-bis((trimethylsilyl)ethynyl)benzothiazole **12**,[2] 3,6-bis((trimethylsilyl)ethynyl)benzene-1,2-diamine **13**,[2] pyrene-4,5-dione **14**[3] and complex **$Ru_2$-9**[4] were prepared according to literature reported procedures.

**Electrochemical and Spectroelectrochemical Measurements.** All electrochemical experiments were performed in a self-constructed cylindrical vacuum-tight one-compartment cell. A spiral-shaped Pt wire and an Ag wire as the counter and reference electrodes are sealed into glass capillaries that are introduced via Quickfit screws at opposite sides of the cell. A platinum electrode is introduced as the working electrode through the top port via a Teflon screw cap with a suitable fitting. It is polished first with 1 μm and then 0.25 μm diamond paste from Buehler-Wirtz before the measurements. The cell may be attached to a conventional Schlenk line via a side arm equipped with a Teflon screw valve that allows experiments to be performed under an atmosphere of argon with approximately 5 mL of analyte solution. $NBu_4^+PF_6^-$ was used as a supporting electrolyte. Referencing was done by the addition of decamethylferrocene ($Cp^*_2Fe$) as an internal standard to the analyte solution after all data of interest had been acquired. Representative sets of scans were repeated with the added standard. Final referencing was done against the ferrocene/ferrocenium ($Cp_2Fe^{0/+}$) redox couple with $E_{1/2}(Cp^*_2Fe^{0/+})$ = −550 mV vs $E_{1/2}(Cp_2Fe^{0/+})$ in the 0.1 M $CH_2Cl_2/NBu_4^+PF_6^-$ supporting electrolyte or -495 mV in 0.1 M $THF/NBu_4^+PF_6^-$. Electrochemical data were acquired with a computer-controlled BASi potentiostat. The OTTLE cell was also lab-built and comprises a Pt-mesh working and counter electrode and a thin silver wire as a pseudoreference electrode sandwiched between the $CaF_2$ windows of a conventional liquid IR cell. Its design follows that of Hartl et al.[5] The working electrode is positioned in the centre of the spectrometer beam.

Simulation of the cyclic voltammograms was performed using the program DigiSim.[6] Mandatory initial parameters where set as follows: Initial concentration = 0.1 mM; electrode surface area = 0.0256 $cm^2$; T = 298 K; electron transfer coefficient $\alpha$ = 0.5; electron transfer rate $k_s$ = 0.008 cm/s. Half-wave potentials were estimated by deconvolution of the corresponding square-wave voltammogram. By loading cyclic voltammograms within the scan rate range of 100 mV/s the program was free to change the chosen parameters in an iterative manner to replicate the shape of the CV at any given scan rate. A fixed value of ca. $3 \times 10^{-7}$ F was eventually added to accommodate for non-Faradaic currents.

FT-IR spectra were recorded on a Bruker Tensor II instrument instrument with a photovoltaic MCT detector and a tungsten lamp source. UV/vis/NIR spectra were obtained on a TIDAS fiberoptic diode array spectrometer (combined MCS UV/NIR and PGS NIR instrumentation) from J&M in HELLMA quartz cuvettes with 0.1 cm optical path lengths. Electron paramagnetic resonance (EPR) studies were performed on a table-top X-band spectrometer MiniScope MS 400 from magnettec. Simulation of the experimental EPR spectra was performed with the MATLAB EasySpin program.[7] All measurements were performed at room temperature and the diruthenium complexes were chemically oxidized using ferrocenium hexafluorophosphate ($Cp_2Fe^+$ $PF_6^-$) or acetylferrocenium hexafluoroantimonate ($AcCp_2Fe^+$ $SbF_6^-$) as oxidizing agents.

**Computational Details.** The ground state electronic structures were calculated by density functional theory (DFT) methods using the Gaussian 09 program packages.[8] Open shell systems were calculated by the unrestricted Kohn- Sham approach (UKS). Geometry optimization followed by vibrational analysis was performed in solvent media. Solvent effects were described by the polarizable continuum model (PCM) with standard parameters for 1,2-dichloroethane.[9] The quasirelativistic Wood-Boring small-core pseudopotentials (MWB)[10] and the corresponding optimized set of basis functions for Ru[11] and 6-31G(d) polarized double-ξ basis sets[12] for the remaining atoms were employed together with the Perdew, Burke, Ernzerhof exchange and correlation functional (PBE0).[13] Additional calculations were performed with the non standard global hybrid functional BLYP35 introduced by Kaup and coworkers.[14] This global hybrid functional was constructed as suggested from BLYP according to the equation: $E_{XC} = ( 1 – \boldsymbol{a} ) (E_X^{LSDA} + \Delta E_X^{B88}) + \boldsymbol{a}\, E_X^{HF} + E_C^{LYP}$ with $\boldsymbol{a} = 0.35$ using the command „BLYP iop(3/76=0650003500)“ in the Gaussian route section and performing frequency analysis in a seperate step with the same commands in the route section. The GaussSum program package was used to analyze the results[15], while the visualization of the results was performed with the Avogadro program package.[16] Graphical representations of molecular orbitals were generated with the help of GNU Parallel[17] and plotted using the vmd program package[18] in combination with POV-Ray.

**X-Ray Crystallography.** X-Ray diffraction analysis was performed on a STOE IPDS-II diffractometer (STOE & CIE GmbH, Darmstadt, Germany) equipped with a graphite monochromated $MoK_\alpha$ radiation source ($\lambda$ = 0.71073 Å) and an image plate detection system at $T$ = 100.15 K. Using *Olex2*,[19] the structures were solved with the *ShelXT*[20] structure solution program using Intrinsic Phasing and refined with the *ShelXL*[20] refinement package using Least Squares minimization. Hydrogen atoms were introduced at their calculated positions. Structure plots were generated with the *ORTEP* program.[21]

**Conductivity Measurements.** The transfer length method (TLM) was first used to check the conductivity of these charge-transfer salts **CT-1** and **CT-2**. This method was adopted from the

literature.[22] Pellets of charge-transfer salt **CT-1** and **CT-2** were made by an IR pellet maker. These pellets were used for conductivity measurement.[23]

**Synthesis and Characterization.**

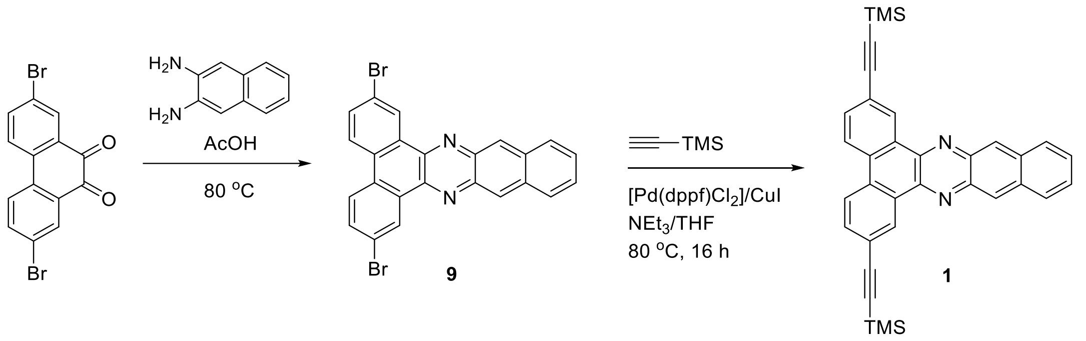


**Scheme S1**. Synthesis of trimethylsilyl-protected diethynyl compound **1.**

**Synthesis of 9.** Compound **9** was synthesized by following a literature reported procedure.[24] In a 100 mL round bottom flask fitted with a magnetic stir bar, 2,7-dibromophenanthrene (312 mg, 0.85 mmol) and 1,2-diaminonapthalene (210 mg, 1.32 mmol) were added. To this mixture, 20 mL of glacial acetic acid were added and then the reaction mixture was stirred for 4 h at 80 °C. After cooling to room temperature, the mixture was poured into water (200 mL). An orange-red precipitate was formed which was washed with water for several times. After drying in vacuum the product **9** was obtained as a deep orange solid in 91% yield (378 mg, 0.774 mmol). The product was directly used for synthesis of the compound **1** without further purification.

**Synthesis of 1**.

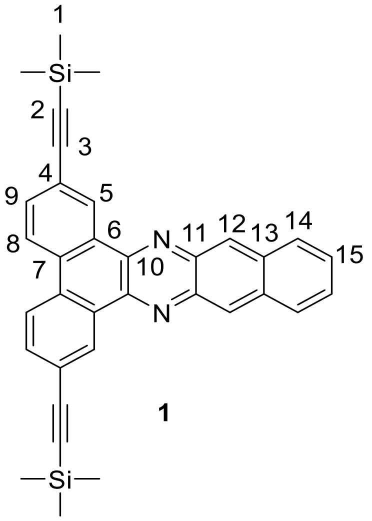


**1**

In a 200 mL Schlenk flask containing a magnetic stir bar, compound **9** (378 mg, 0.774 mmol), [Pd(dppf)$Cl_2$] (57 mg, 0.078 mmol) and copper(I) iodide (15 mg, 0.078 mmol) were put under nitrogen atmosphere and dissolved in a mixture of dry dimethylformamide (5 mL), tetrahydrofuran (10 mL) and

triethylamine (7 mL). Trimethylsilylacetylene (0.44 mL, 3.1 mmol) was then added dropwise into the mixture. After stirring the reaction mixture at 80 °C for 48 h, the solvent was evaporated. Compound **1** was purified from the solid residue by column chromatography over silica (petroleum ether : dichloromethane, 2:1). The product was obtained as an orange solid.

**Yield**: 328 mg (0.64 mmol, 81%).

**$^1$H NMR** (400 MHz, $CDCl_3$): $\delta$ in ppm = 9.43 (s, 2H, $H_5$), 8.90 (s, 2H, $H_{12}$), 8.35 (dd, $^3J_{H,H}$ = 7.7 Hz, $^4J_{H,H}$ = 1.2 Hz, 2H, $H_8$), 8.15-8.12 (m, 2H, $H_{14}$), 7.81 (dd, $^3J_{H,H}$ = 7.7 Hz, $^4J_{H,H}$ = 1.2 Hz, 2H, $H_9$), 7.59-7.53 (m, 2H, $H_{15}$), 0.31 (18 H, Si(C$\underline{H}_3$)$_3$).

**$^{13}$C{$^1$H} NMR** (100 MHz, $CDCl_3$): $\delta$ in ppm = 142.9 ($C_6$), 138.9 ($C_{11}$), 134.3 ($C_{13}$), 133.7 ($C_9$), 131.6 ($C_7$), 130.6 ($C_4$), 130.3 ($C_5$), 128.6 ($C_{14}$), 127.6 ($C_{12}$), 126.8 ($C_{15}$), 123.4 ($C_8$), 104.7 ($C_3$), 96.4 ($C_2$), 0.04 (Si($\underline{C}H_3$)$_3$).

**ESI-MS** (positive ion mode), *m/z*: $[M]^+$ 523.2017 (calcd for $C_{34}H_{31}N_2Si_2$ = 523.2020).

**Elemental (CHN) Analysis**: Found for **1**: C 77.91, H 5.82, N 5.24 (calcd for $C_{34}H_{30}N_2Si_2{\cdot}H_2O$: C 78.11, H 5.78, N 5.36).

**Synthesis of 2.**

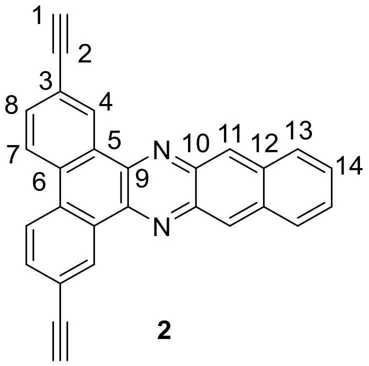


**2**

Compound **1** (90 mg, 0.172 mmol) was dissolved in dichloromethane/tetrahydrofuran/methanol solution (10:30:15 mL). An excess of potassium fluoride was added, and the reaction mixture was stirred overnight at room temperature. After complete deprotection (checked by TLC), the volume of the solution was reduced to 15 mL under reduced pressure. A precipitate formed, was isolated by filtration and washed with water (2 × 20 mL) and methanol (2 × 15 mL). The product was obtained as a yellow solid.

**Yield**: 60 mg (0.158 mmol, 92%).

**$^1$H NMR** (600 MHz, $CDCl_3$): $\delta$ in ppm = 9.56 (d, $^3J_{H,H}$ = 1.2 Hz, 2H, $H_4$), 8.95 (s, 2H, $H_{11}$), 8.44 (d, $^3J_{H,H}$ = 8.3 Hz, 2H, $H_8$), 8.23-8.17 (m, 2H, $H_{13}$), 7.88 (dd, $^3J_{H,H}$ = 8.3 Hz, $^4J_{H,H}$ = 1.2 Hz, 2H, $H_8$), 7.63-7.59 (m, 2H, $H_{14}$), 3.31 (s, 2H, $H_1$).

**$^{13}$C{$^1$H} NMR** (150 MHz, $CDCl_3$): $\delta$ in ppm = 142.7 ($C_9$), 138.9 ($C_{10}$), 134.4 ($C_{12}$), 133.8 ($C_8$), 131.7 ($C_3$), 130.7 ($C_6$), 130.5 ($C_4$), 129.8 ($C_6$), 128.6 ($C_{13}$), 127.7 ($C_{11}$), 126.9 ($C_{14}$), 123.5 ($C_7$), 122.4 ($C_3$), 83.3 ($C_2$).

**ESI-MS** (+ve ion mode), *m/z*: $[M]^+$ 379.1228 (calcd for $C_{28}H_{15}N_2$ = 379.1230).

**Synthesis of $Ru_2$-3.**

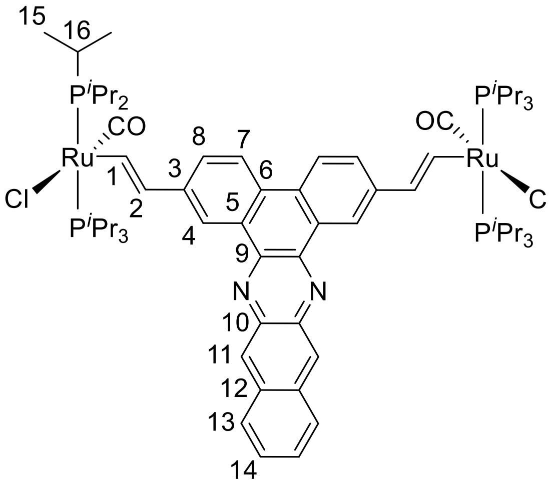


To a 100 mL Schlenk flask, deprotected dialkyne **2** (26.2 mg, 0.069 mmol) and $[HRu(CO)(Cl)(P^iPr_3)_2]$ (74 mg, 0.152 mmol) were added. After adding dichloromethane (10 mL) into the flask, the mixture was stirred at room temperature for 2 h. The colour of reaction mixture turned from dark red to dark grey-green. The solution was evaporated to dryness. The dark grey-green solid was then washed with *n*-hexane (3 × 5 mL) and MeOH (3 × 5 mL).

**Yield**: 82.4 mg (0.061 mmol, 88%).

**$^1$H NMR** (400 MHz, $CD_2Cl_2$): $\delta$ in ppm = 9.01 (d, $^3J_{H,H}$ = 13.5 Hz, 2H, $H_1$), 8.88 (s, 2H, $H_{11}$), 8.84 (s, 2H, $H_4$), 8.21 (d, $^3J_{H,H}$ = 8.4 Hz, 2H, $H_7$), 8.20-8.15 (m, 2H, $H_{13}$), 7.63-7.55 (m, 2H, $H_{14}$), 7.54 (dd, $^3J_{H,H}$ = 8.4 Hz, $^4J_{H,H}$ = 2.0 Hz, 2H, $H_8$), 6.33 (d, $^3J_{H,H}$ = 13.5 Hz, 2H, $H_2$), 2.89-2.65 (m, 12H, $H_{16}$), 1.40-1.19 (m, 72H, $H_{15}$).

**$^{13}$C{$^1$H} NMR** (200 MHz, $CD_2Cl_2$): $\delta$ in ppm = 203.5 (t, $^2J_{C,P}$ = 13.0 Hz, Ru-$\underline{C}$O), 156.7 (t, $^2J_{C,P}$ = 12.0 Hz, $C_1$), 144.9 ($C_9$), 139.2 ($C_3$), 138.1 ($C_{10}$), 134.4 ($C_2$), 134.2 ($C_4$), 129.8 ($C_6$), 130.5 ($C_{12}$), 129.2 ($C_{11}$), 128.8 ($C_{13/14}$), 127.6 ($C_{11}$), 127.0 ($C_{13/14}$), 126.8 ($C_8$), 123.2 ($C_7$), 121.5 ($C_5$), 25.1 (t, $^2J_{C,P}$ = 8.6 Hz, $C_{16}$), 20.12, 20.0 ($H_{15}$).

**$^{31}$P{$^1$H} NMR** (160 MHz, $CD_2Cl_2$): $\delta$ in ppm = 38.43 (s, $\underline{P}^iPr_3$).

**IR** ($CH_2Cl_2$): 1910 $cm^{-1}$ ($\nu_{C≡O}$).

**ESI-MS** (+ve ion mode, $CH_2Cl_2$), *m/z*: [**M**]$^+$ 1350.426 (calcd for $C_{66}H_{100}Cl_2N_2O_2P_4Ru_2$ = 1350.421).

**Synthesis of $Ru_2$-4.**

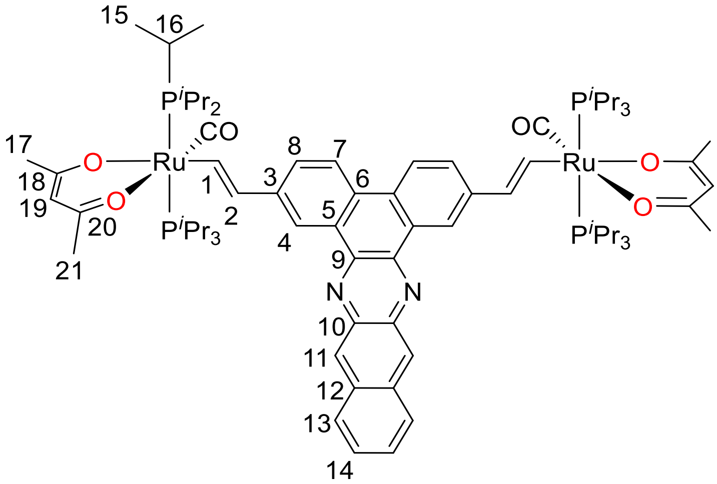


To a 25 mL flask, pentacoordinated diruthenium complex **$Ru_2$-3** (31 mg, 0.023 mmol) was put under nitrogen atmosphere. Subsequently, dichloromethane (5 mL), acetylacetone (4.6 mg, 0.046 mmol) and potassium carbonate (6 mg, 0.046 mmol) were added into the flask. The reaction mixture was stirred for 4 h at the room temperature, while the colour of the solution changed from grey-green to dark green. After evaporating solution under reduced pressure, the residue was filtered which was washed with methanol (3 × 2 mL) and hexane (3 × 2 mL).

**Yield:** 29 mg (0.019 mmol, 83%).

**$^1$H NMR** (800 MHz, $CD_2Cl_2$): $\delta$ in ppm = 9.44 (d, $^3J_{H,H}$ = 16.5 Hz, 2H, $H_1$), 8.95 (s, 2H, $H_{11}$), 8.94 (d, $^4J_{H,H}$ = 2.0 Hz, 2H, $H_4$), 8.28 (d, $^3J_{H,H}$ = 8.5 Hz, 2H, $H_7$), 8.27-8.23 (m, 2H, $H_{13}$), 7.77 (dd, $^3J_{H,H}$ = 8.5 Hz, $^4J_{H,H}$ = 2.0 Hz, 2H, $H_8$), 7.65-7.60 (m, 2H, $H_{14}$), 6.82 (d, $^3J_{H,H}$ = 16.5 Hz, 2H, $H_2$), 5.41 (s, 2H, $H_{19}$), 2.50-2.37 (m, 12H, $H_{16}$), 2.05 (s, 6H, $H_{17/21}$), 1.86 (s, 6H, $H_{17/21}$), 1.42-1.28 (m, 72H, $H_{15}$).

**$^{13}$C{$^1$H} NMR** (200 MHz, $CD_2Cl_2$): $\delta$ in ppm = 209.8 (t, $^2J_{C,P}$ = 15.1 Hz, Ru-$\underline{C}$O), 188.23 ($C_{20}$), 186.5 ($C_{18}$), 169.6 (t, $^2J_{C,P}$ = 13.2 Hz, $C_1$), 144.9 ($C_9$), 140.2 ($C_3$), 138.9 ($C_{10}$), 133.76 ($C_{12}$), 133.69 ($C_2$), 129.8 ($C_6$), 128.4 ($C_{13}$), 127.1 ($C_{11}$), 126.3 ($C_8$), 126.1 ($C_{14}$), 122.4 ($C_7$), 120.9 ($C_4$), 100.0 ($C_{19}$), 28.35 (C17), 28.39 (C21), 24.4 (t, $^2J_{C,P}$ = 8.6 Hz, $C_{16}$), 19.51, 19.33 ($H_{15}$).

**$^{31}$P{$^1$H} NMR** (200 MHz, $CD_2Cl_2$): $\delta$ in ppm = 36.45 (s, $\underline{P}^i$Pr$_3$).

**IR** ($CH_2Cl_2$): 1899 cm$^{-1}$ ($\nu_{C\equiv O}$).

**ESI-MS** (+ve ion mode, $CH_2Cl_2$), *m/z*: $[M]^+$ 1478.5643 (calcd. for $C_{76}H_{114}N_2O_6P_4Ru_2$ = 1478.5744).

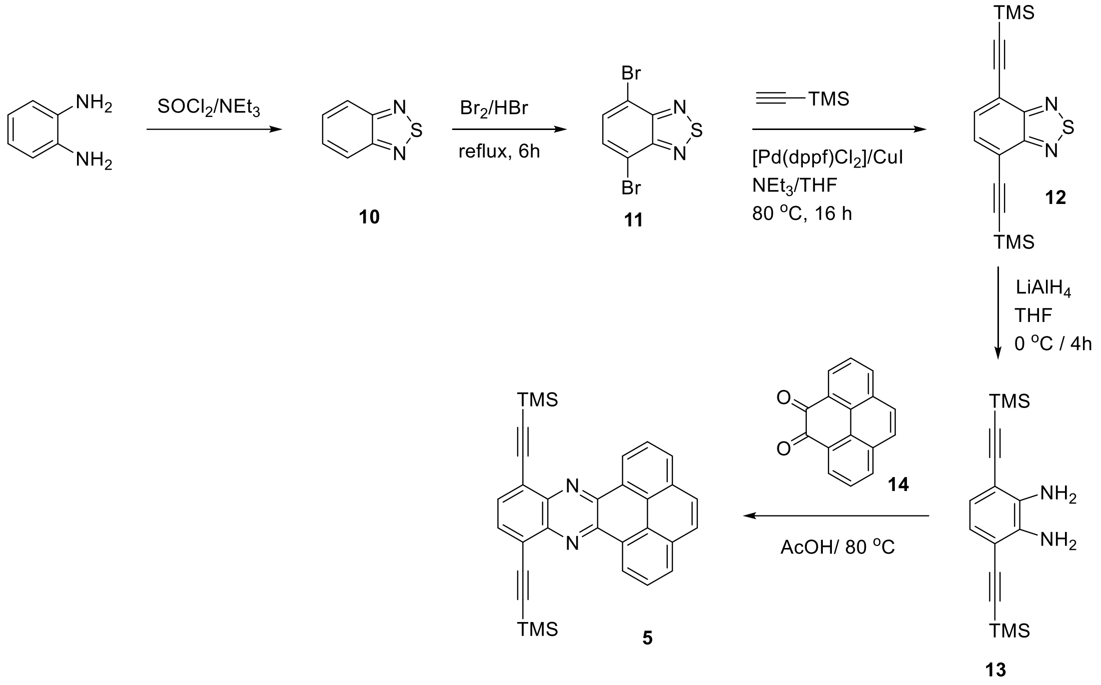

**Scheme S2**. Synthesis of trimethylsilyl-protected dialkyne **5.**

**Synthesis of 5**. To a 100 mL flask, diamino trimethylsilyl-protected dialkyne **13**[25] (0.93 g, 3.1 mmol) and pyrene-4,5-dione **14**[3] (0.72 g, 3.1 mmol) were added. Glacial acetic acid (50 mL) was added and the reaction mixture was heated to 80 °C for 24 h. The reaction was monitored by thin layer chromatography (TLC). After completion of the reaction, the solution was evaporated to dryness. The crude product was dissolved in dichloromethane, washed with $NaHCO_3$ solution (2 × 100 mL) and filtered over $MgSO_4$. The product was purified by column chromatography ($SiO_2$, PE/DCM).

**Yield:** 1.031 g (67%).

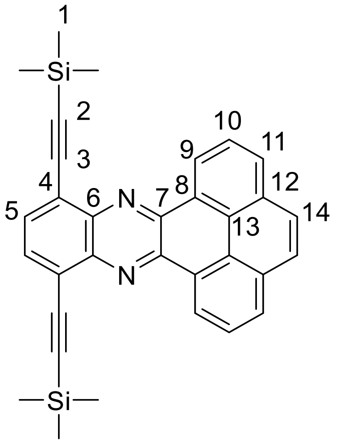

**H NMR** (600 MHz, $CDCl_3$): $\delta$ in ppm = 9.62 (d, $^3J_{H,H}$ = 7.7 Hz, 2H, $H_9$), 8.31 (d, $^3J_{H,H}$ = 7.7 Hz, 2H, $H_{11}$), 8.10 (t, $^3J_{H,H}$ = 7.7 Hz, 2H, $H_{10}$), 8.03 (s, 2H, $H_5$), 7.98 (s, 2H, $H_{13}$), 0.50 (18 H, Si(C$\underline{H}_3$)$_3$).

**$^{13}$C{$^1$H} NMR** (150 MHz, $CDCl_3$): $\delta$ in ppm = 143.6 ($C_8$), 142.4 ($C_6$) 133.4 ($C_5$), 131.4 ($C_{12}$), 129.7 ($C_{11}$), 129.3 ($C_{14}$), 127.3 ($C_{13}$), 126.9 ($C_{10}$), 126.4 ($C_7$), 124.5 ($C_9$), 123.8 ($C_4$), 103.7 ($C_3$), 101.6 ($C_2$), 0.11 (Si($\underline{C}H_3$)$_3$).

**ESI-MS** (+ve ion mode), *m/z*: $[M]^+$ 497.1857 (calcd for $C_{32}H_{29}N_2Si_2$ = 497.1864).
**Elemental (CHN) Analysis**: Found for **5**: C 76.43, H 6.19, N 5.13 (calcd for $C_{32}H_{28}N_2Si_2$: C 77.37, H 5.68, N 5.64).

**Synthesis of 6.**

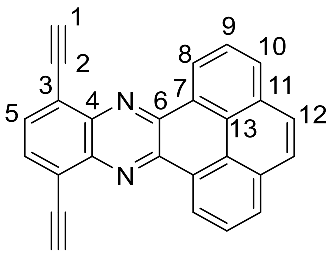


In a 250 mL flask fitted with a magnetic stir bar, compound **5** (0.511 g, 1 mmol) was added. A mixture of methanol (50 mL) and tetrahydrofuran (50 mL) was added into the flask to completely dissolve the reactant. Subsequently, potassium fluoride (0.174 g, 3 mmol) was added and the reaction mixture was stirred overnight at the room temperature. After completion of reaction (checked by TLC), water (100 mL) and dichloromethane (100 mL) were added. The organic phase was separated, washed with water (2 × 50 mL) and dried over $MgSO_4$. Dialkyne **6** was isolated as a microcrystalline orange solid and dried under vacuum.
**Yield**: 0.302 g (86%).
**$^1$H NMR** (800 MHz, $CDCl_3$): $\delta$ in ppm = 9.64 (dd, $^3J_{H,H}$ = 7.6 Hz, $^4J_{H,H}$ = 1.2 Hz, 2H, $H_8$), 8.30 (dd, $^3J_{H,H}$ = 7.6 Hz, $^4J_{H,H}$ = 1.2 Hz, 2H, $H_{10}$), 8.09 (t, $^3J_{H,H}$ = 7.6 Hz, 2H, $H_9$), 8.04 (s, 4H, $H_{12}$ and $H_5$), 3.82 (s, 2H, $H_1$).
**$^{13}$C{$^1$H} NMR** (200 MHz, $CDCl_3$): $\delta$ in ppm = 143.9 ($C_6$), 142.3 ($C_4$) 133.9 ($C_5$), 131.4 ($C_{11}$), 129.8 ($C_{10}$), 129.1 ($C_7$), 127.2 ($C_{12}$), 127.03 ($C_9$), 126.4 ($C_{13}$), 124.7 ($C_8$), 123.4 ($C_3$), 85.3 ($C_2$), 80.4 ($C_1$).
**ESI-MS** (+ve ion mode), *m/z*: $[M]^+$ 353.1068 (calcd for $C_{26}H_{13}N_2$ = 353.1073).
**Elemental (CHN) Analysis**: Found for **6**: C 86.03, H 3.94, N 7.62 (calcd for $C_{26}H_{12}N_2$: C 88.62, H 3.43, N 7.95).

**Synthesis of $Ru_2$-7.**

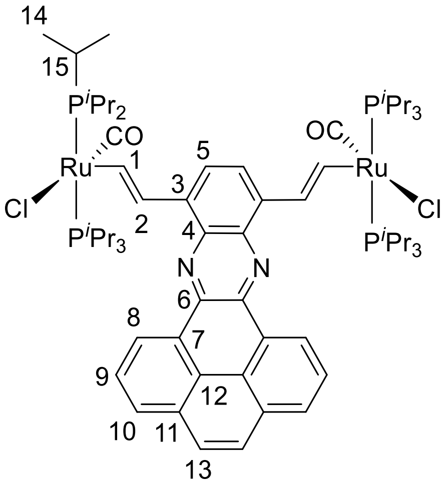


To a 100 mL Schlenk flask, dialkyne **6** (0.105 g, 0.3 mmol) and [HRuCO)(Cl)($P^iPr_3)_2$] (0.301 g, 0.62 mmol) were added. Dichloromethane (10 mL) was added into the flask and the mixture was stirred at room temperature for 2 h. The colour of reaction mixture turned from red to dark green. The solution was evaporated to dryness. The dark green solid was then washed with *n*-hexane (3 × 7 mL) and MeOH (3 × 7 mL).

**Yield**: 0.285 g (72%).

**$^1$H NMR** (600 MHz, $CD_2Cl_2$): $\delta$ in ppm = 9.61 (d, $^3J_{H,H}$ = 7.7 Hz, 2H, $H_8$), 9.10 (d, $^3J_{H,H}$ = 13 Hz, 2H, $H_1$), 8.28 (dd, $^3J_{H,H}$ = 7.7 Hz, $^4J_{H,H}$ = 1.2 Hz, 2H, $H_{10}$), 8.11 (t, $^3J_{H,H}$ = 7.7 Hz, 2H, $H_9$), 8.07 (s, 2H, $H_{13}$), 7.64 (d, $^3J_{H,H}$ = 13 Hz, 2H, $H_2$), 7.52 (s, 2H, $H_5$), 2.90-2.75 (m, 12H, $H_{15}$), 1.42-127 (m, 72H, $H_{14}$).

**$^{13}$C{$^1$H} NMR** (150 MHz, $CD_2Cl_2$): $\delta$ in ppm = 203.4 (t, $^2J_{C,P}$ = 13 Hz, Ru-<u>C</u>O), 155.2 (t, $^2J_{C,P}$ = 16 Hz, $C_1$), 140.7 ($C_6$), 138.4 ($C_4$), 131.8 ($C_{11}$), 131.4 ($C_5$), 130.4 ($C_{12}$), 129.8 ($C_2$), 128.2 ($C_{10}$), 127.1 ($C_9$), 126.8 ($C_{13}$), 125.9 ($C_7$), 124.1 ($C_3$), 123.7 ($C_8$), 24.5 (C15), 19.8, 19.6 (C14), 24.4 (t, $^2J_{C,P}$ = 8.6 Hz, $C_{16}$), 19.51, 19.33 ($H_{15}$).

**$^{31}$P{$^1$H} NMR** (160 MHz, $CD_2Cl_2$): $\delta$ in ppm = 38.84 (s, <u>P</u>$^i$Pr$_3$).

**IR** ($CH_2Cl_2$): 1912 cm$^{-1}$ ($\nu_{C\equiv O}$).

**ESI-MS** (+ve ion mode, $CH_2Cl_2$), *m/z*: [**M**]$^+$ 1324.4063 (calcd for $C_{64}H_{98}Cl_2N_2O_2P_4Ru_2$ 1324.4058).

**Synthesis of $Ru_2$-8.**

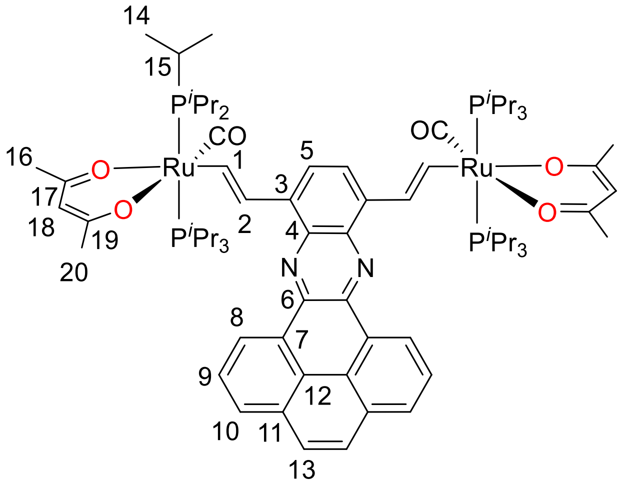


To a 50 mL flask, pentacoordinated diruthenium complex **$Ru_2$-7** (26.5 mg, 0.02 mmol) was added. Subsequently, dichloromethane (5 mL), acetylacetone (4 mg, 0.04 mmol) and potassium carbonate (5.5 mg, 0.04 mmol) were added into the flask. The reaction mixture was stirred for 4 h at room temperature, while the colour of the solution changed from dark green to leafy-green. The solution was evaporated to dryness and washed with methanol (3 × 2 mL) and hexane (3 × 2 mL).

**Yield**: 17.6 mg (62%).

**$^1$H NMR** (800 MHz, $CD_2Cl_2$): $\delta$ in ppm = 9.72 (dd, $^3J_{H,H}$ = 7.7 Hz, $^4J_{H,H}$ = 1.2 Hz, 2H, $H_8$), 9.32 (d, $^3J_{H,H}$ = 16 Hz, 2H, $H_1$), 8.30 (dd, $^3J_{H,H}$ = 7.7 Hz, $^4J_{H,H}$ = 1.2 Hz, 2H, $H_{10}$), 8.20 (d, $^3J_{H,H}$ = 16 Hz, 2H, $H_2$), 8.12 (t, $^3J_{H,H}$ = 7.7 Hz, 2H, $H_9$), 8.10 (s, 2H, $H_{13}$), 7.79 (s, 2H, $H_5$), 5.37 (s, 2H, $H_{18}$), 2.53-2.38 (m, 12H, $H_{15}$), 2.01 (s, 6H, $H_{16/20}$), 1.83 (s, 6H, $H_{16/20}$), 1.43-1.21 (m, 72H, $H_{14}$).

**$^{13}$C{$^1$H} NMR** (150 MHz, $CD_2Cl_2$): $\delta$ in ppm = 188.5 ($C_{17/19}$), 186.6 ($C_{17/19}$), 167.8 (t, $^2J_{C,P}$ = 16 Hz, $C_1$), 140.2 ($C_6$), 138.7 ($C_4$), 134.2 ($C_{11}$), 131.4 ($C_5$), 131.0 ($C_{12}$), 129.1 ($C_2$), 127.9 ($C_{10}$), 127.1 ($C_9$), 126.6 ($C_{13}$), 125.9 ($C_7$), 123.5 ($C_8$), 123.1 ($C_3$), 100.0 ($C_{18}$), 28.41 ($C_{16/20}$), 28.36 ($C_{16/20}$), 24.4 (t, $^2J_{C,P}$ = 8.8 Hz, $C_{15}$), 19.58 ($C_{14}$), 19.42 ($C_{14}$).

**$^{31}$P{$^1$H} NMR** (160 MHz, $CD_2Cl_2$): $\delta$ in ppm = 36.15 (s, $\underline{P}^i$Pr$_3$).

**IR** ($CH_2Cl_2$): 1898 cm$^{-1}$ ($\nu_{C\equiv O}$).

**ESI-MS** (+ve ion mode), *m/z*: Found [**M**]$^+$ at 1452.5614 (calcd for $C_{74}H_{112}N_2O_6P_4Ru_2$ = 1452.5587).

**Synthesis of $Ru_2$-10.**

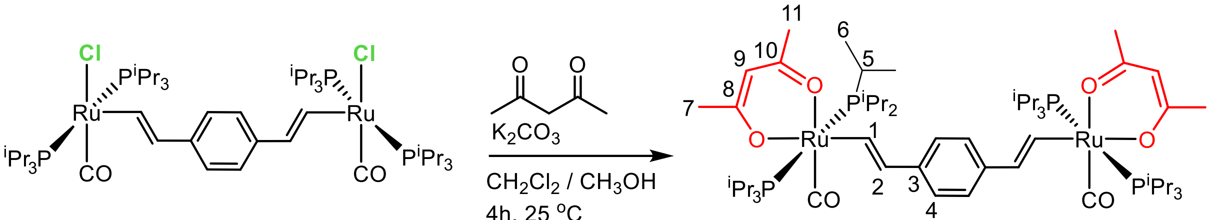


**Ru$_2$-9** **Ru$_2$-10**

To a 50 mL flask, pentacoordinated diruthenium complex **$Ru_2$-9**{Maurer, 2006 #225} (56 mg, 0.05 mmol) was added. Subsequently, dichloromethane (5 mL), acetylacetone (11 mg, 0.11 mmol) and potassium carbonate (15 mg, 0.11 mmol) were added into the flask. The reaction mixture was stirred for 4 h at room temperature, while the colour of the solution changed from dark purple to grey-green. The solution was evaporated to dryness and washed with methanol (3 × 2 mL) and hexane (3 × 2 mL).

**Yield**: 47.1 mg (76%).

**$^1$H NMR** (500 MHz, $CD_2Cl_2$): $\delta$ in ppm = 8.68 (d, $^3J_{H,H}$ = 16 Hz, 2H, $H_1$), 6.97 (s, 4H, $H_4$), 6.31 (d, $^3J_{H,H}$ = 16 Hz, 2H, $H_2$), 5.30 (s, 2H, $H_9$), 2.44-2.24 (m, 12H, $H_5$), 1.91 (s, 6H, $H_{7/11}$), 1.77 (s, 6H, $H_{7/11}$), 1.34-1.19 (m, 72H, $H_6$).

**$^{13}$C{$^1$H} NMR** (126 MHz, $CD_2Cl_2$): $\delta$ in ppm = 210.2 (t, $^2J_{C,P}$ = 14 Hz, Ru-$\underline{C}$O), 188.3 ($C_{8/10}$), 186.4 ($C_{8/10}$), 161.8 (t, $^2J_{C,P}$ = 12 Hz, $C_1$), 137.4 ($C_3$), 134.0 ($C_2$), 123.6 ($C_4$), 99.9 ($C_9$), 28.35 ($C_{7/11}$), 28.33 ($C_{7/11}$), 24.2 (t, $^2J_{C,P}$ = 8.7 Hz, $C_5$), 19.5, 19.3 ($C_{14}$).

**$^{31}$P{$^1$H} NMR** (162 MHz, $CD_2Cl_2$): $\delta$ in ppm = 36.2 (P$^i$Pr$_3$).

**IR** ($CH_2Cl_2$): 1896 cm$^{-1}$ ($\nu_{C\equiv O}$).

**Synthesis of [CoCp$_2$][F$_4$TCNQ]**

This compound is already known in the literature[26] and was made by salt metathesis from $[Cp_2Co]^+$ $PF_6^-$ and the potassium salt of F$_4$TCNQ$^{\bullet-}$. We made it here by the simpler irect oxidation of cobaltocene with F$_4$TCNQ. In a 50 mL flask, F$_4$TCNQ (14 mg, 0.05 mmol) was dissolved in dichloromethane (10 mL). $Cp_2Co$ (9.5 mg, 0.05 mmol) was added and the solution was stirred for 1 hour. The solvent was evaporated and the green residue was washed with diethylether (2x 5 mL). The product [CoCp$_2$][F$_4$TCNQ] was obtained in quantitative yield.

**IR** (KBr, cm$^{-1}$): 2195 (s), 2176 (m), 1632 (m), 1599 (m), 1536 (m), 1499 (m), 1416 (m), 1388 (s), 1345 (m), 1264 (w), 1198 (w), 1143 (w).

**Figure S1.** $^{1}H$ NMR spectrum of **1** measured in $CDCl_3$ (298 K).

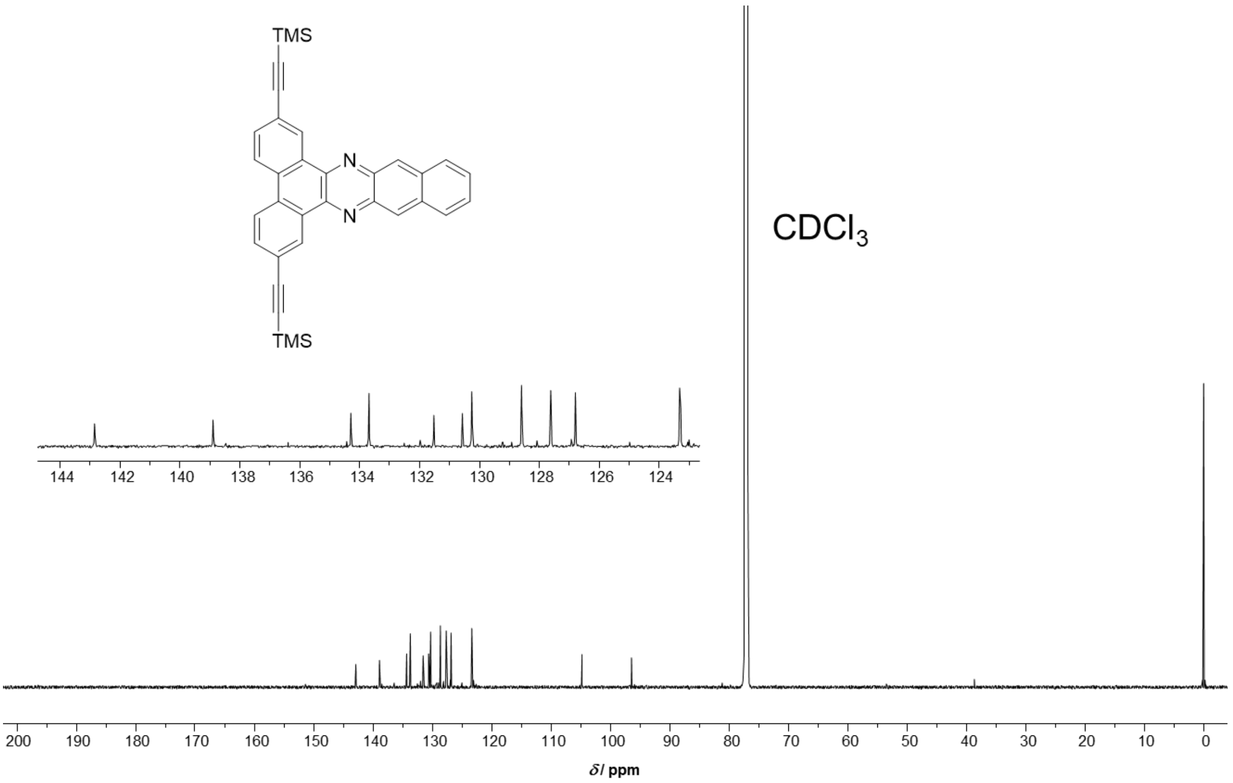


**Figure S2.** $^{13}C\{^{1}H\}$ NMR spectrum of **1** measured in $CDCl_3$ (298 K).

**Figure S3.** $^{1}$H NMR spectrum of **2** measured in $CDCl_3$ (298 K).

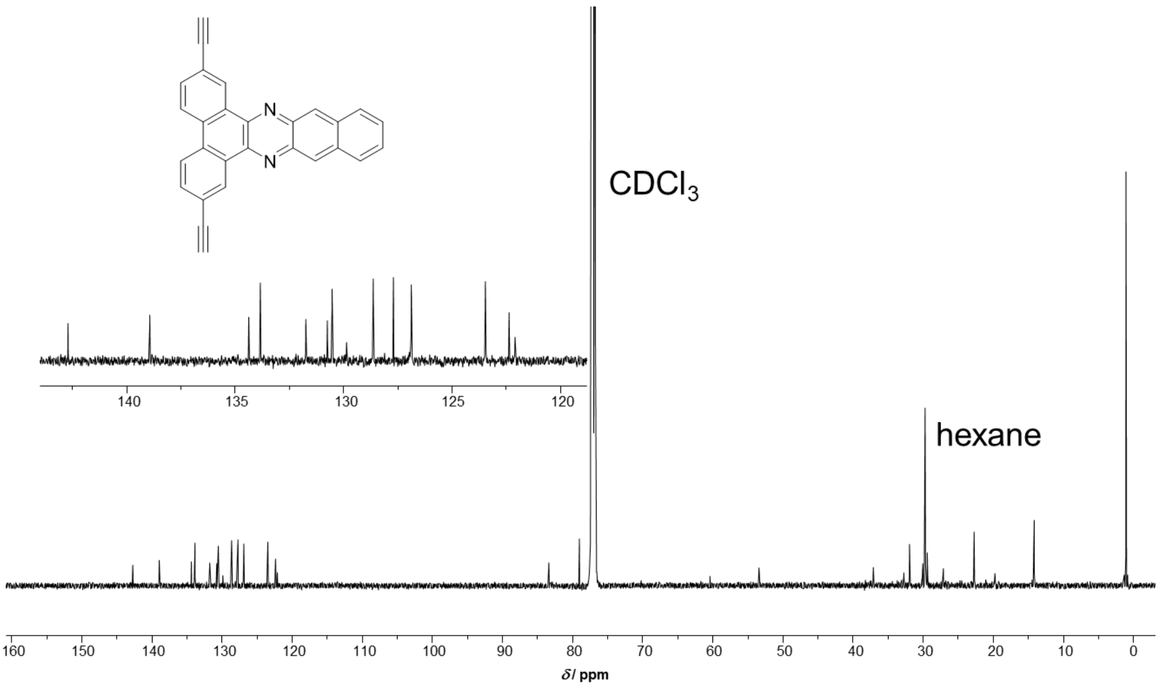


**Figure S4.** $^{13}C\{^{1}H\}$ NMR spectrum of **2** measured in $CDCl_3$ (298 K).

**Figure S5.** $^{1}H$ NMR spectrum of **$Ru_2$-3** measured in $CD_2Cl_2$ (298 K).

**Figure S6.** $^{31}P\{^{1}H\}$ NMR spectrum of **$Ru_2$-3** measured in $CD_2Cl_2$ (298 K).

**Figure S7.** $^{13}C\{^{1}H\}$ NMR spectrum of **$Ru_2$-3** measured in $CD_2Cl_2$ (298 K).

**Figure S8.** $^{1}H$ NMR spectrum of **$Ru_2$-4** measured in $CD_2Cl_2$ (298 K).

**Figure S9.** $^{31}P\{^1H\}$ NMR spectrum of **$Ru_2$-4** measured in $CD_2Cl_2$ (298 K).

**Figure S10.** $^{13}C\{^1H\}$ NMR spectrum of **$Ru_2$-4** measured in $CD_2Cl_2$ (298 K).

**Figure S11.** $^{1}$H NMR spectrum of **5** measured in $CDCl_3$ (298 K).

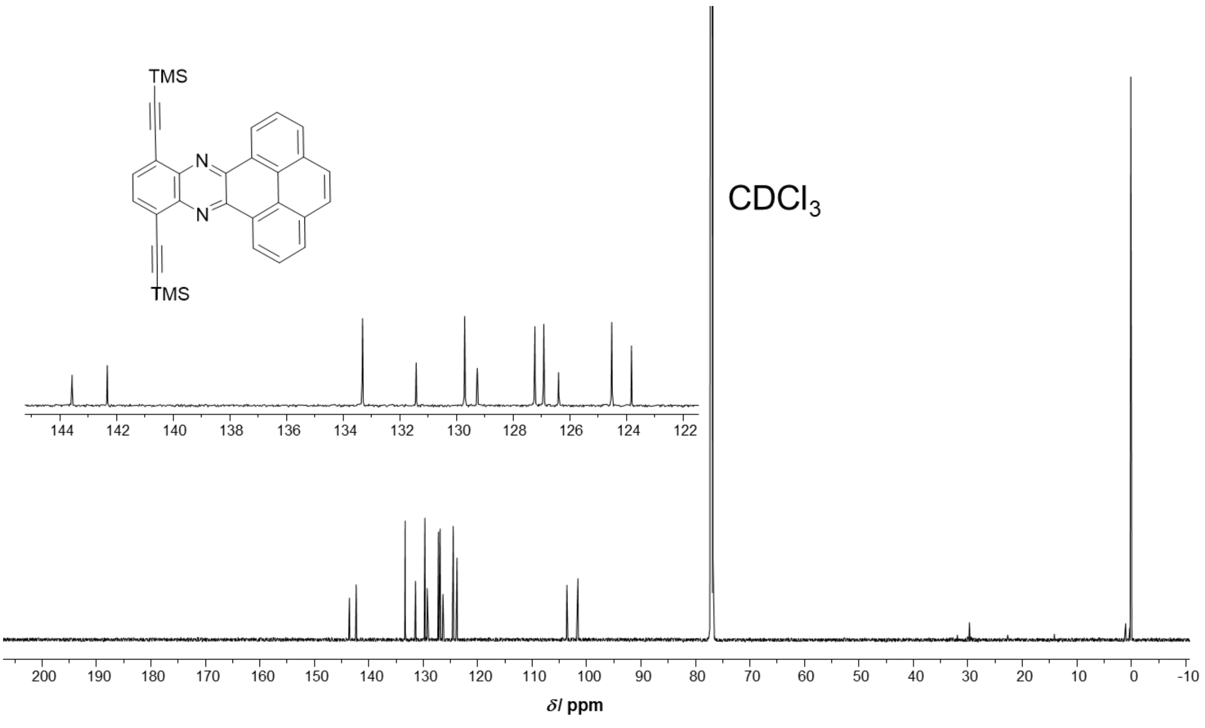


**Figure S12.** $^{13}$C{$^{1}$H} NMR spectrum of **5** measured in $CDCl_3$ (298 K).

Figure S13. $^{1}$H NMR spectrum of **6** measured in $CDCl_3$ (298 K).

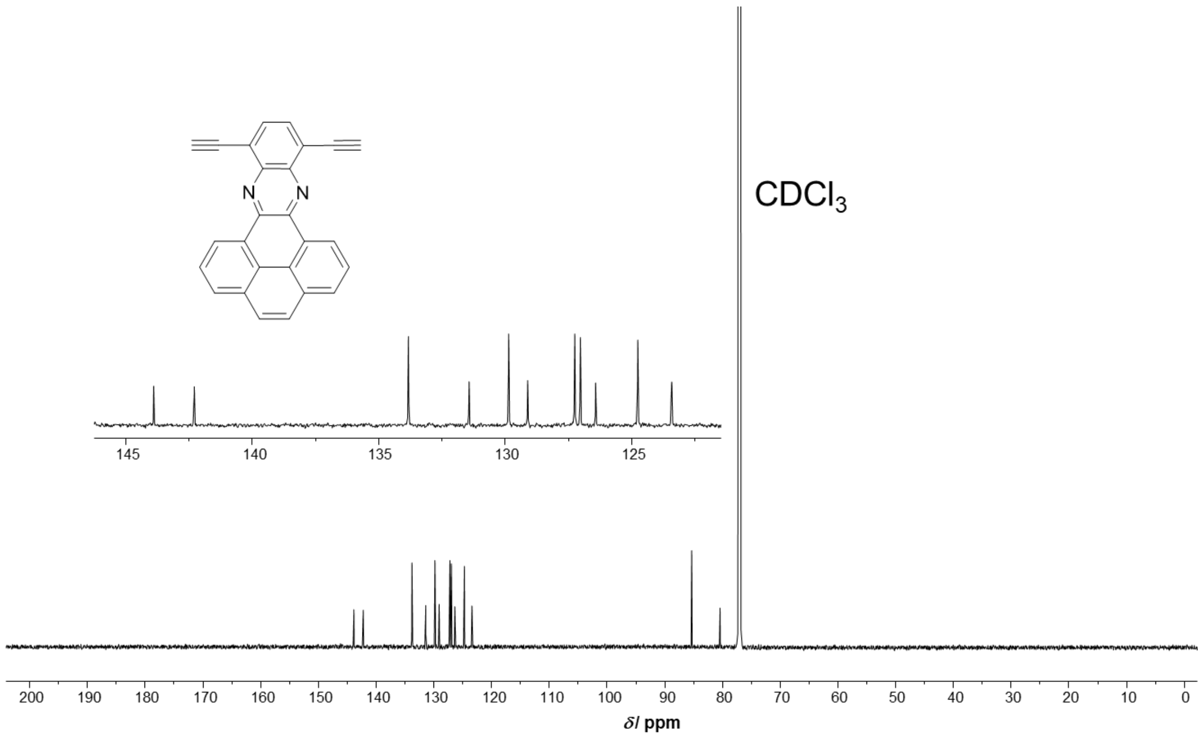


**Figure S14.** $^{13}C\{^{1}H\}$ NMR spectrum of **6** measured in $CDCl_3$ (298 K).

**Figure S15.** $^{1}H$ NMR spectrum of **$Ru_2$-7** measured in $CD_2Cl_2$ (298 K).

**Figure S16.** $^{31}P\{^{1}H\}$ NMR spectrum of **$Ru_2$-7** measured in $CD_2Cl_2$ (298 K).

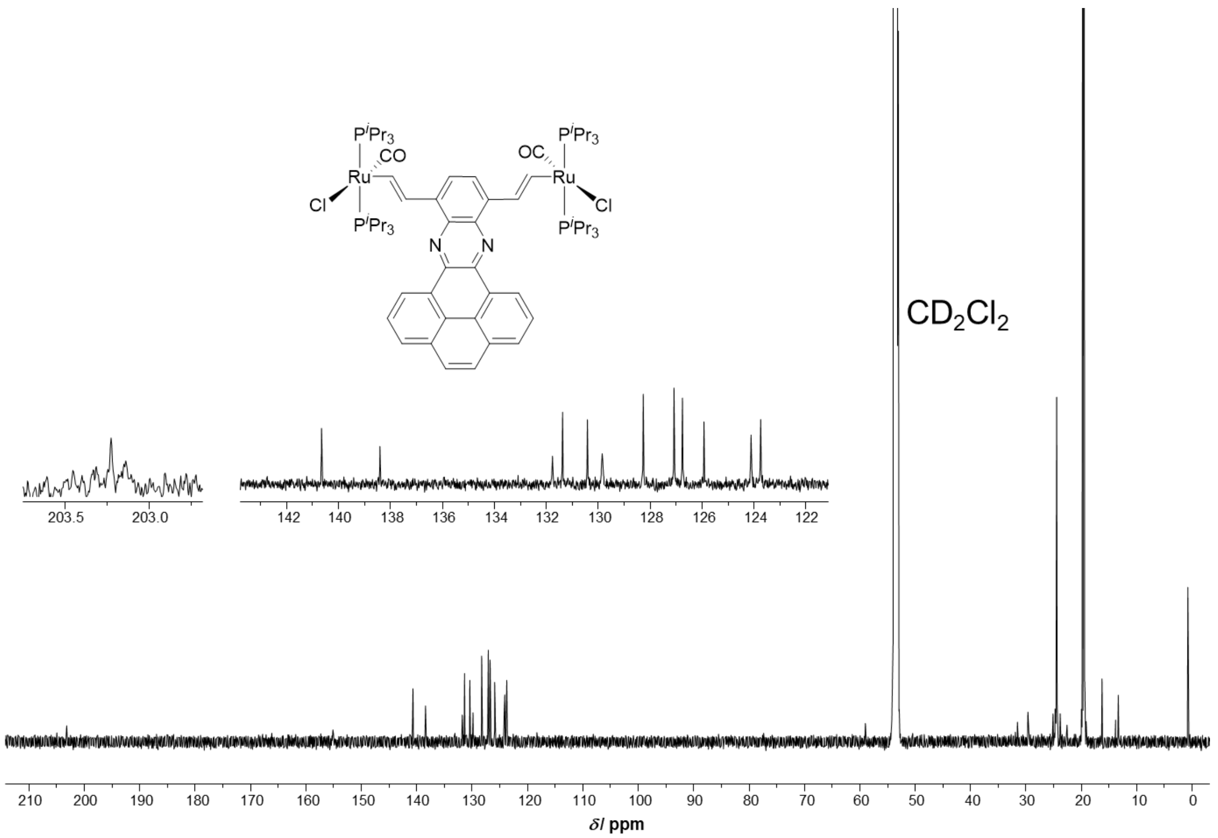


**Figure S17.** $^{13}C\{^1H\}$ NMR spectrum of **$Ru_2$-7** measured in $CD_2Cl_2$ (298 K).

**Figure S18.** $^1H$ NMR spectrum of **$Ru_2$-8** measured in $CD_2Cl_2$ (298 K).

**Figure S19.** $^{31}P\{^1H\}$ NMR spectrum of **$Ru_2$-8** measured in $CD_2Cl_2$ (298 K).

**Figure S20.** $^{13}C\{^1H\}$ NMR spectrum of **$Ru_2$-8** measured in $CD_2Cl_2$ (298 K).

**Figure S21.** $^{1}$H NMR spectrum of **Ru$_2$-10** measured in $CD_2Cl_2$ (298 K).

**Figure S22.** $^{31}$P{$^{1}$H} NMR spectrum of **Ru$_2$-10** measured in $CD_2Cl_2$ (298 K).

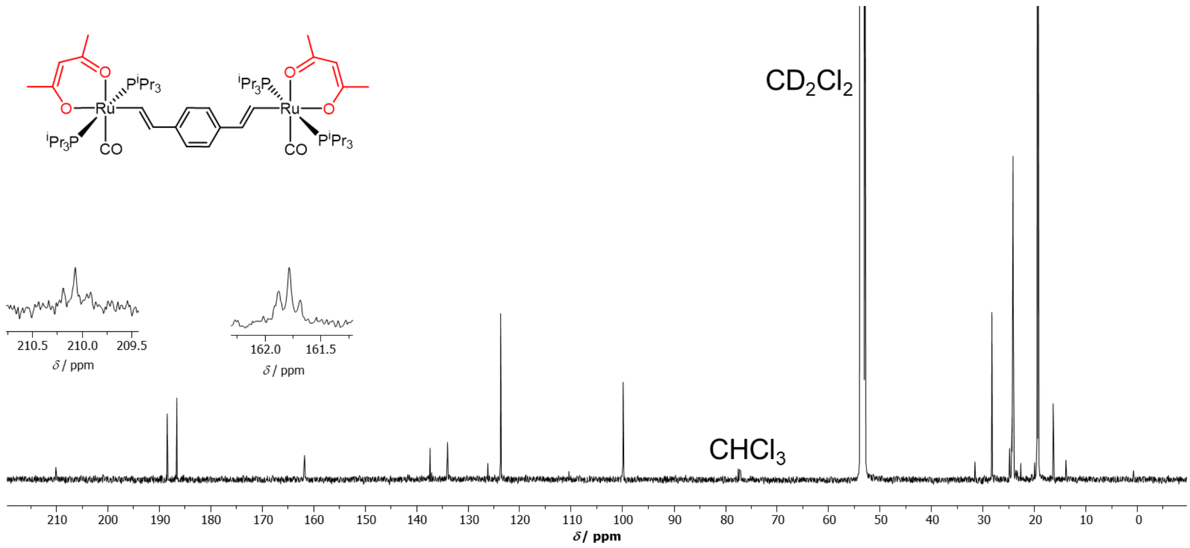


**Figure S23.** $^{13}$C{$^{1}$H} NMR spectrum of **Ru$_2$-10** measured in $CD_2Cl_2$ (298 K).

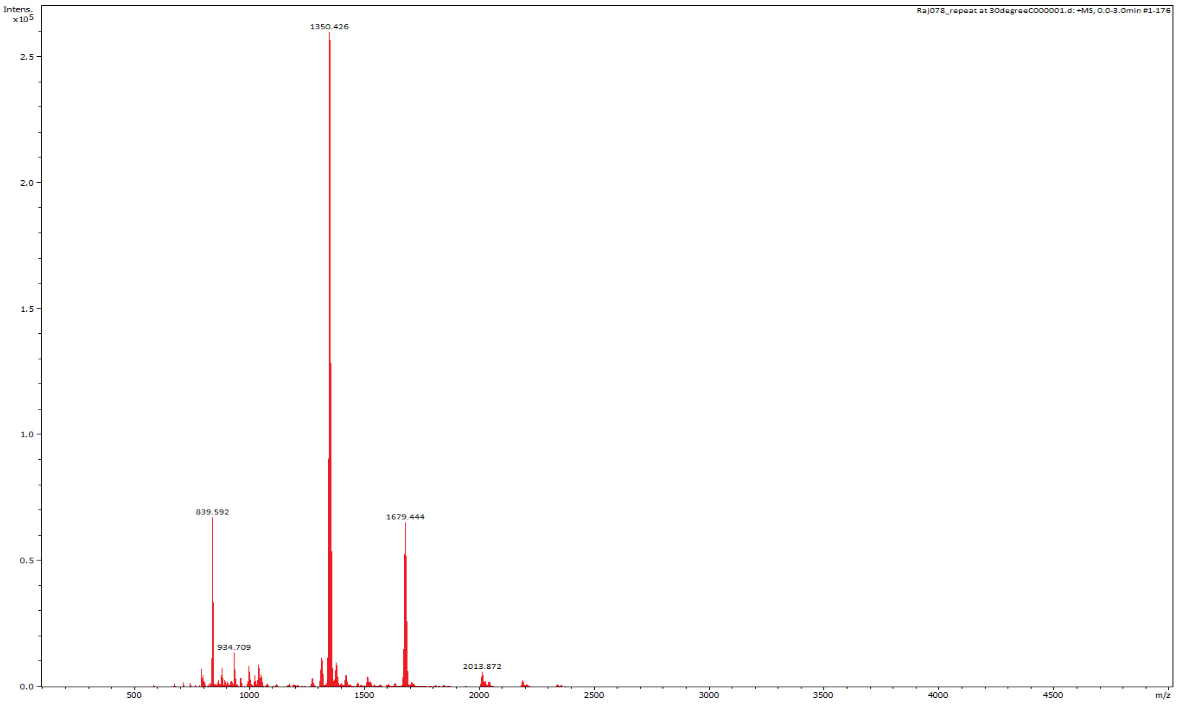


**Figure S24.** High-resolution electrospray ionisation mass spectrum (HR ESI-MS) of **$Ru_2$-3** (+ve ion mode).

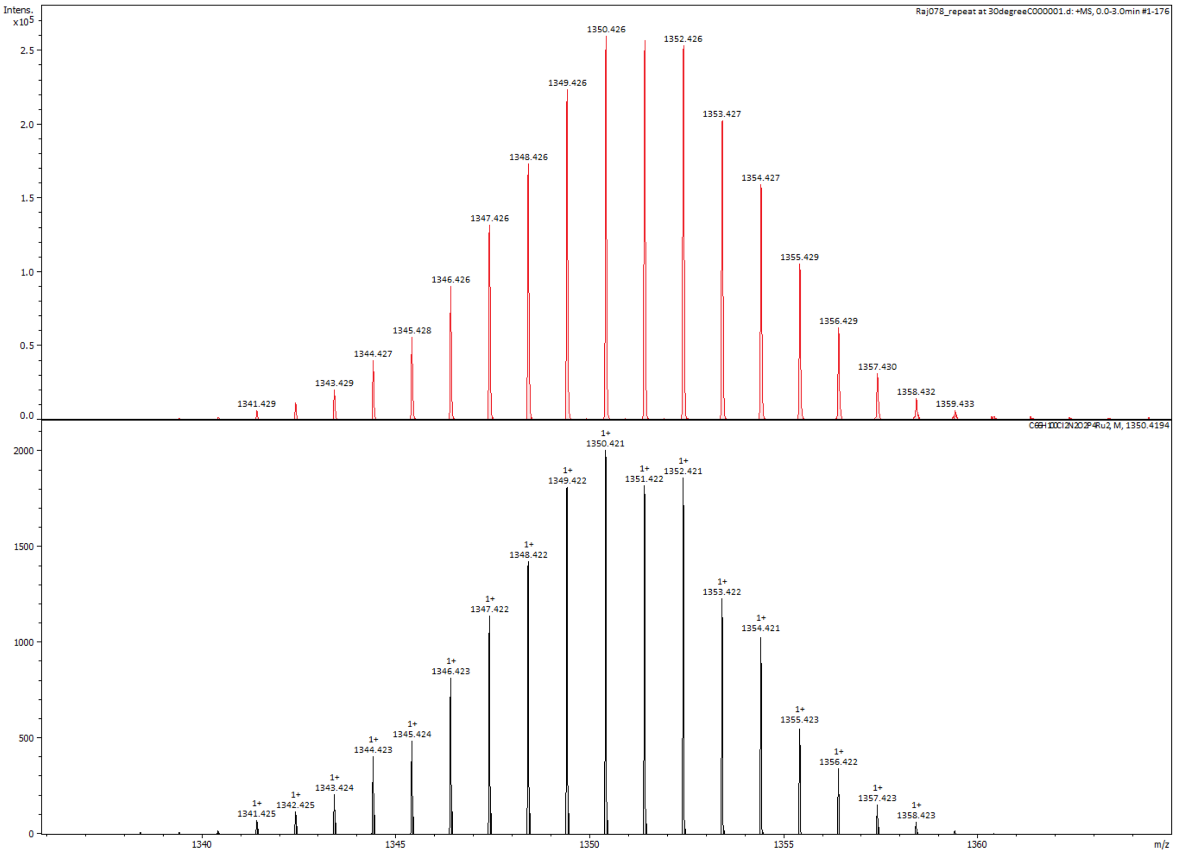


**Figure S25.** Isotopic pattern of the molecular ion $[\mathbf{M}]^+$ peak of **$Ru_2$-3**. Experimental spectrum (red) is displayed on top and simulated spectrum (black) is at the bottom.

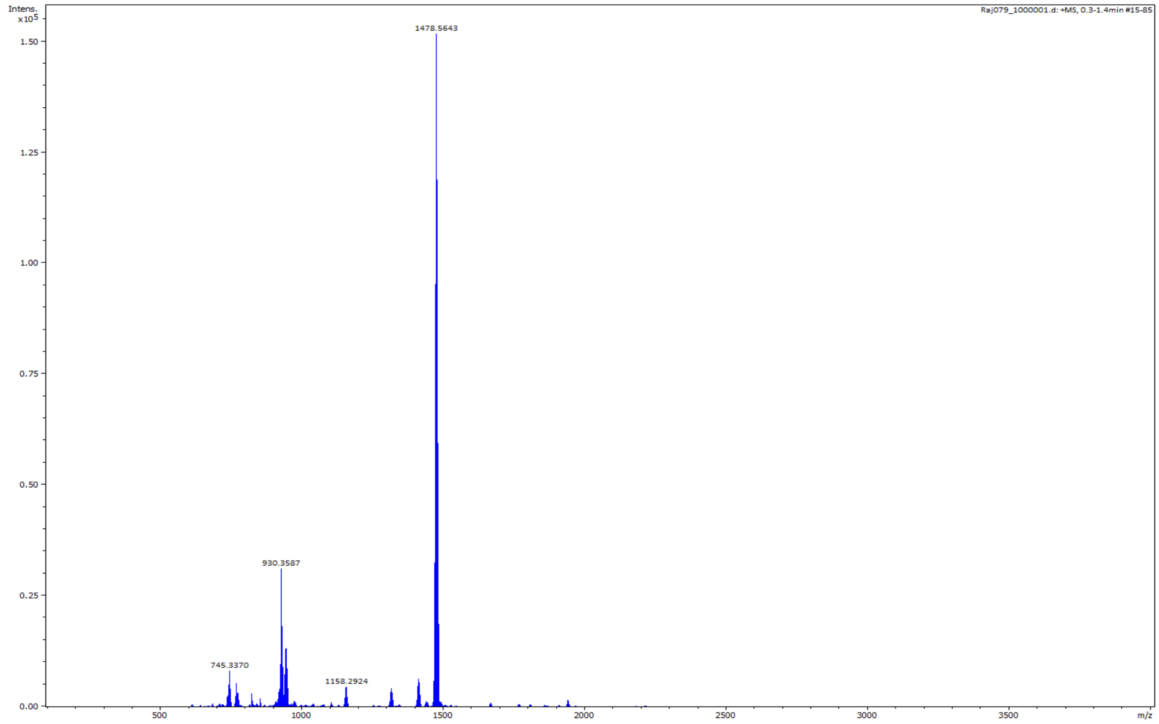


**Figure S26.** High-resolution electrospray ionisation mass spectrum (HR ESI-MS) of **$Ru_2$-4** (+ve ion mode).

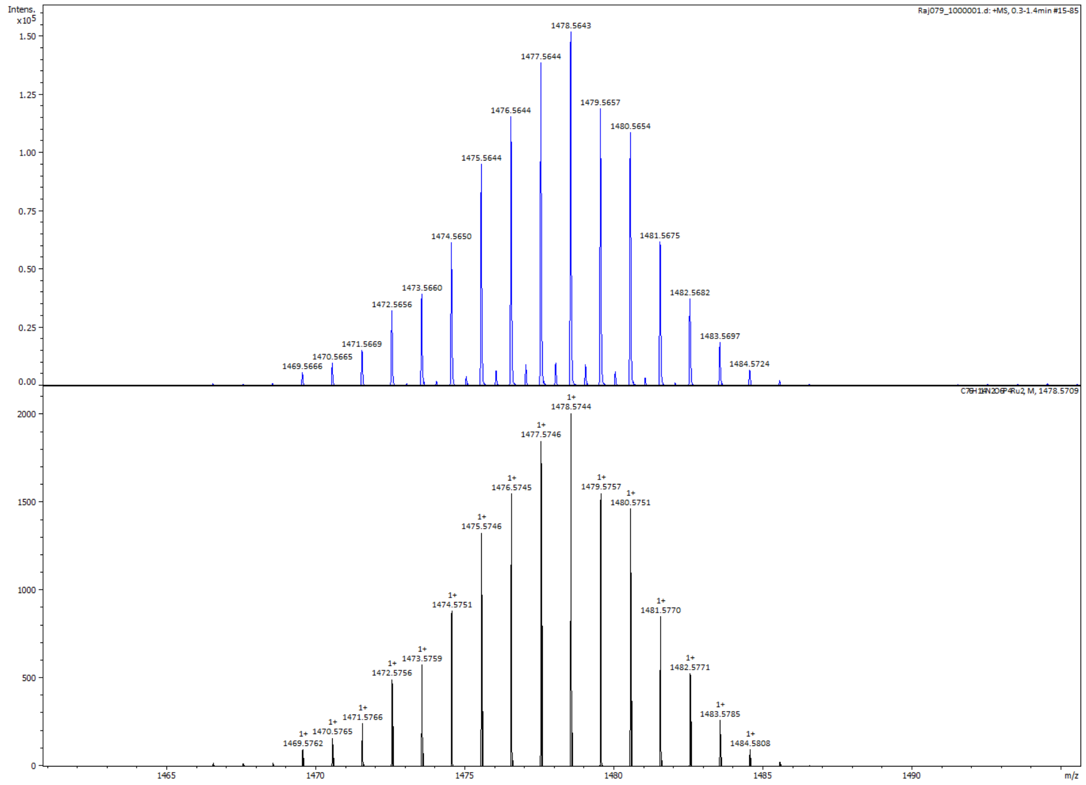


**Figure S27.** Isotopic pattern of the molecular ion [**M**]$^+$ peak of **$Ru_2$-4**. Experimental spectrum (blue) is displayed on top and simulated spectrum (black) is at the bottom.

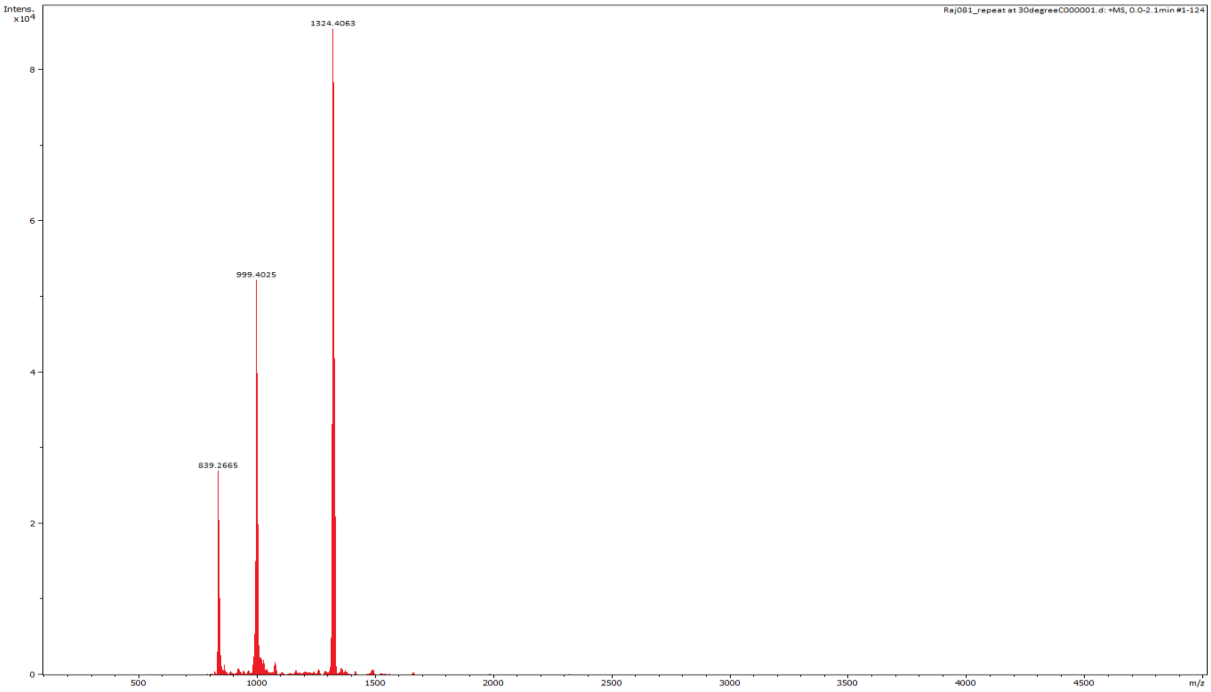


**Figure S28.** High-resolution electrospray ionisation mass spectrum (HR ESI-MS) of **$Ru_2$-7** (+ve ion mode).

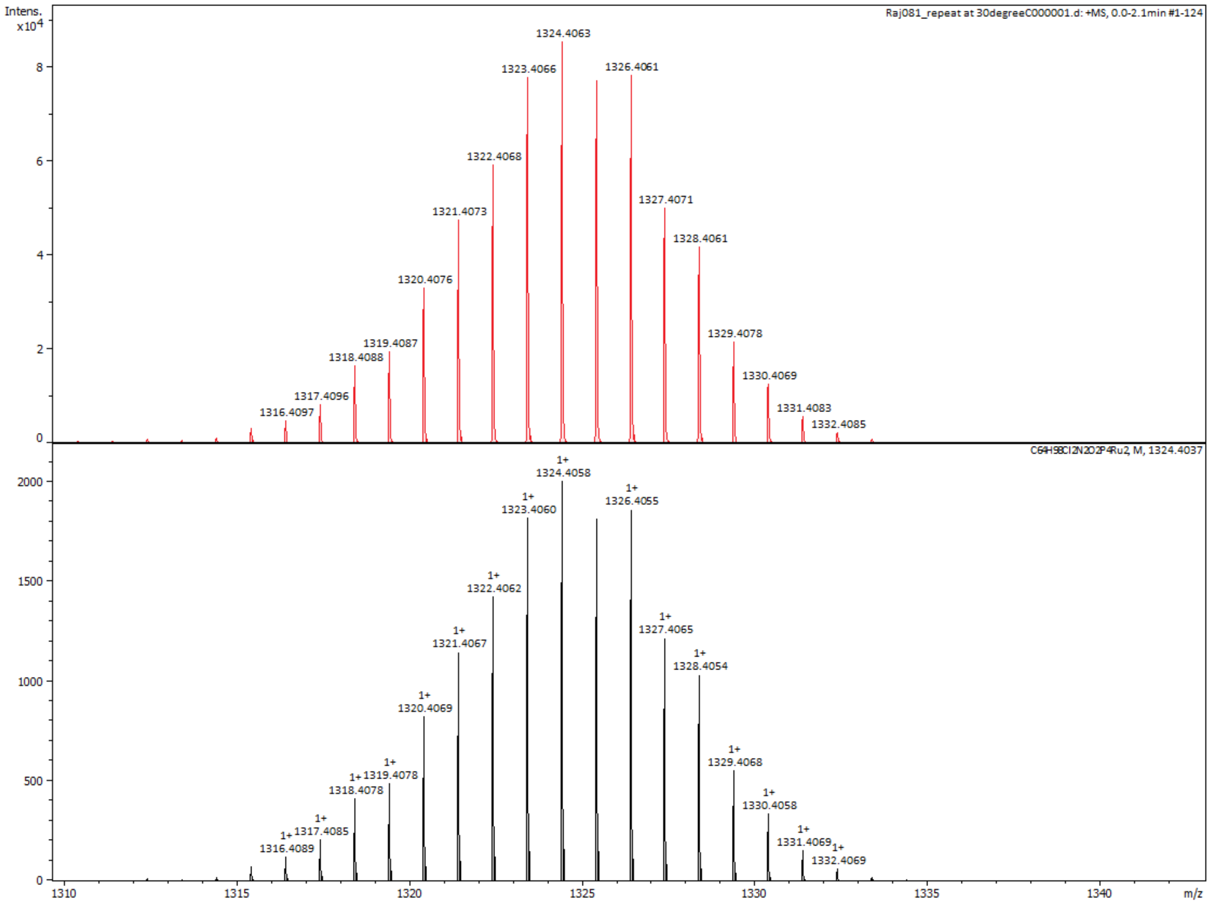


**Figure S29.** Isotopic pattern of the molecular ion $[\mathbf{M}]^+$ peak of **$Ru_2$-7**. Experimentally observed spectrum (red) is displayed on top and simulated spectrum (black) is at the bottom.

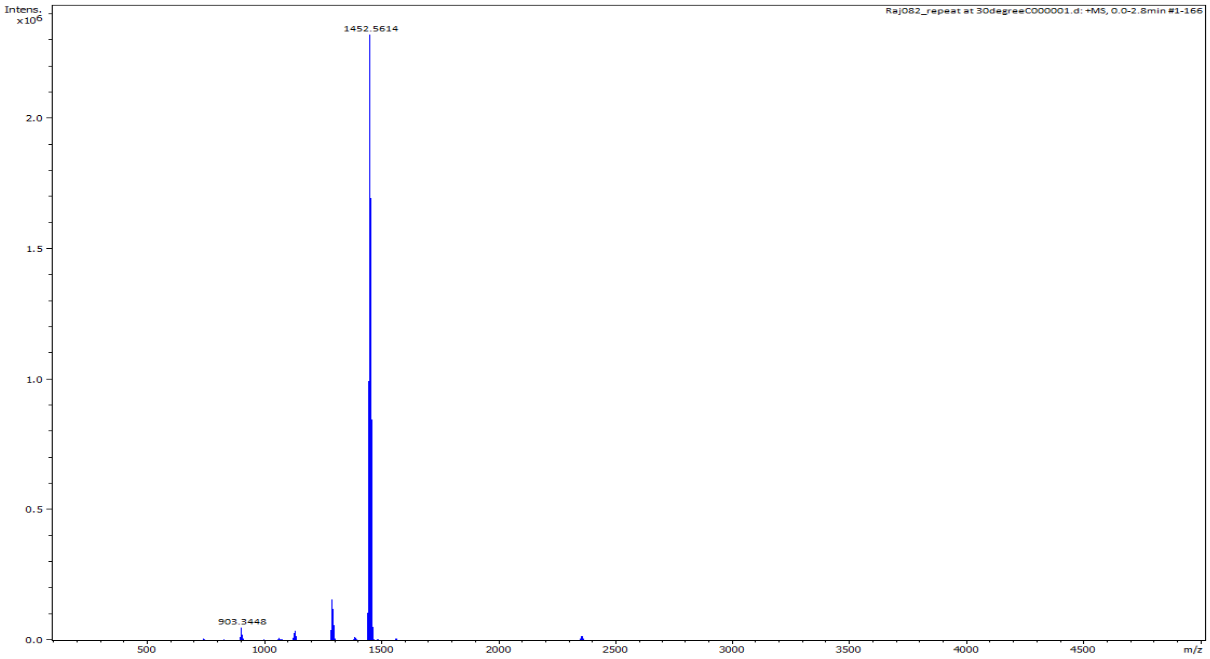


**Figure S30.** High-resolution electrospray ionisation mass spectrum (HR ESI-MS) of **$Ru_2$-8** (+ve ion mode).

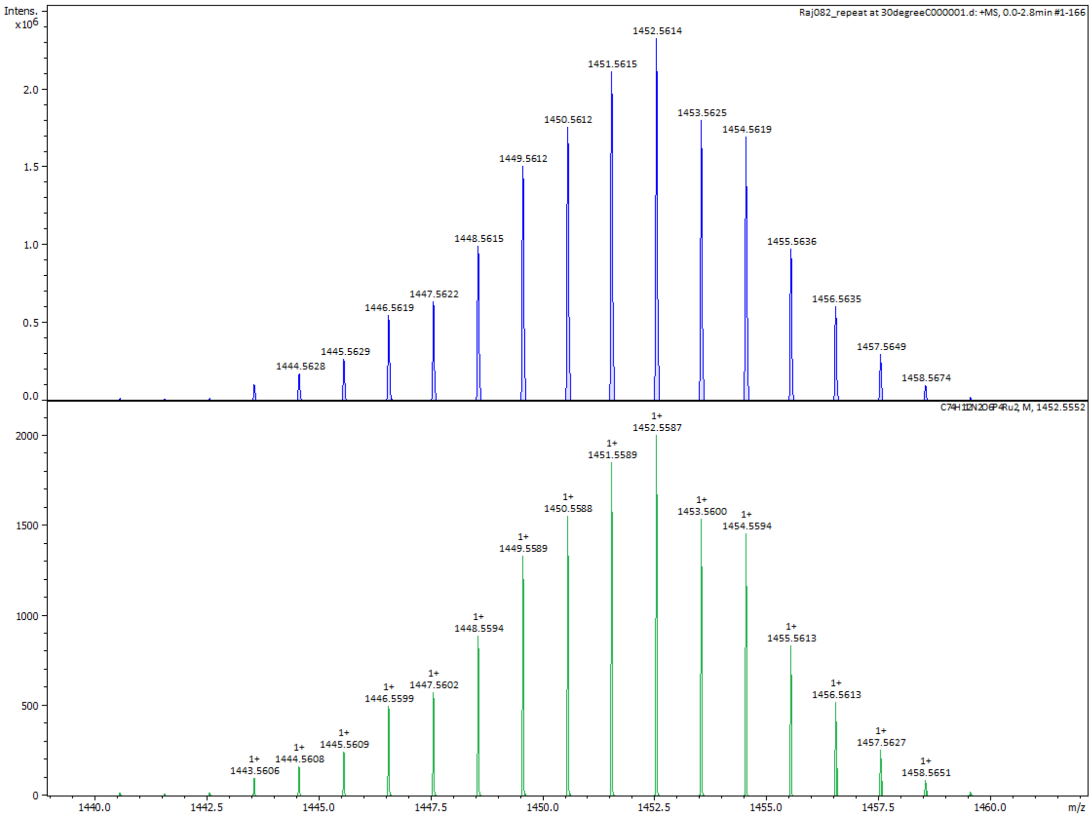


**Figure S31.** Isotopic pattern of the molecular ion [**M**]$^+$ peak of **$Ru_2$-8**. Experimentally observed spectrum (blue) is displayed on top and simulated spectrum (green) is at the bottom.

**X-Ray Crystallography**

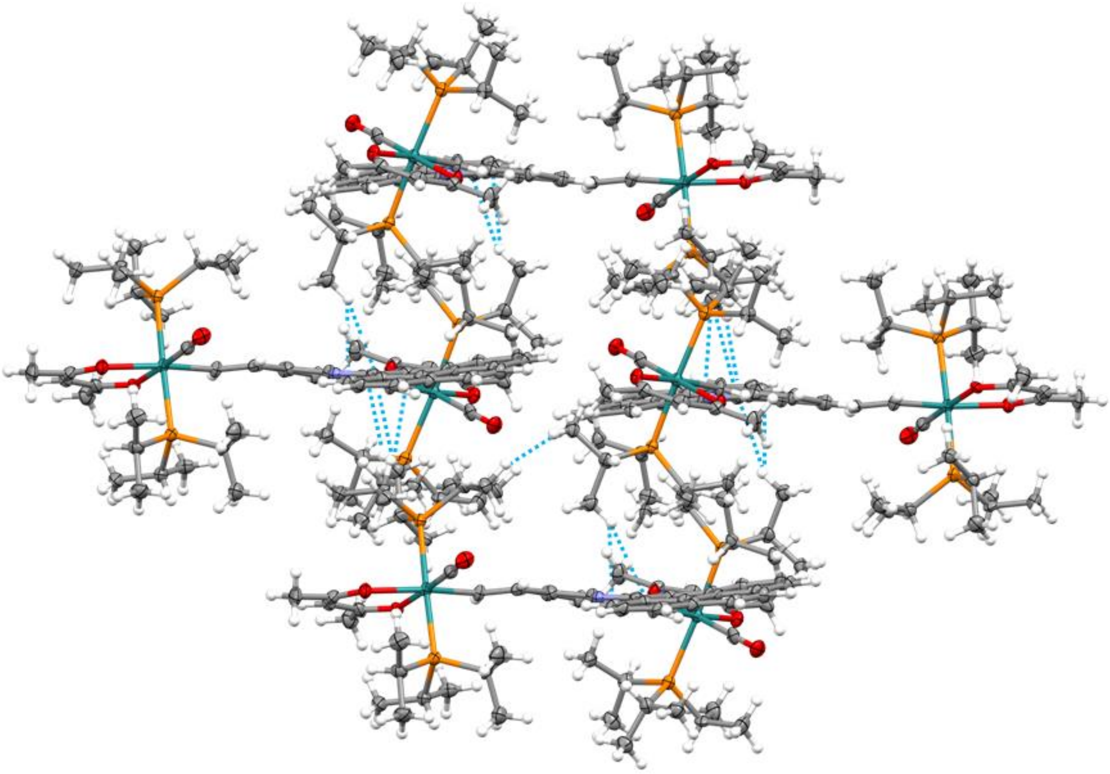

**Figure S32**. Packing of individual molecules of **$Ru_2$-8** in the solid state with short intermolecular CH···π interactions between the extended polyaromatic hydrocarbon (PAH) moieties and the bulky triisopropylphosphine ligands indicated by blue dotted lines.

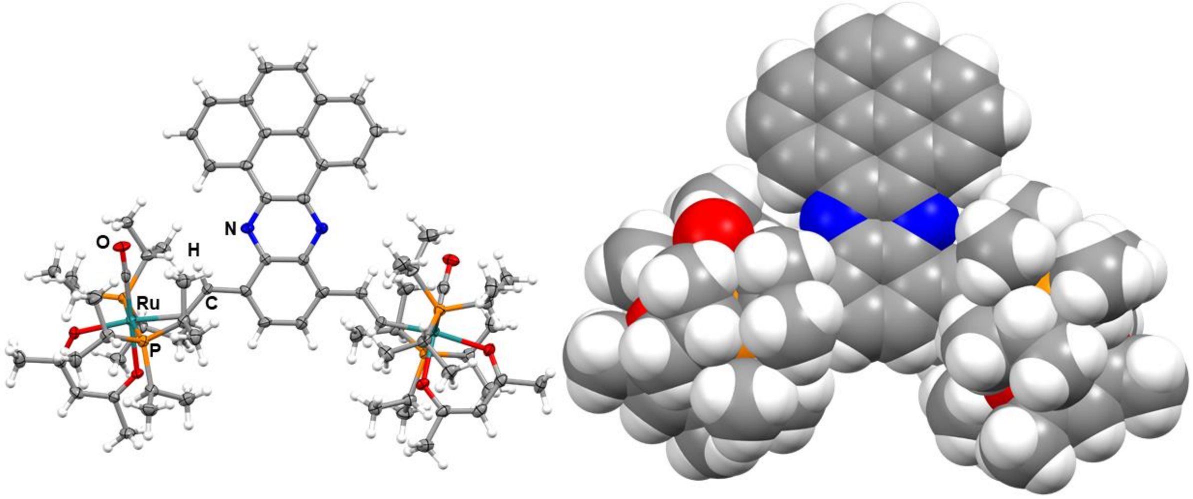


**Figure S33**. Molecular structure of **$Ru_2$-8** in **$Ru_2$-8** · $4CH_2Cl_2$. Solvent molecules are omitted for clarity reasons. Thermal ellipsoids are displayed at a 50% probability level. The space-filling model is also depicted at the right side, which confirms the presence of the open electron-rich pyrene platform available for π-stacking.

**Table S1. Crystal data and structure refinement for $Ru_2$-8.**

| | |
|---|---|
| CCDC No. | 2126940 |
| Identification code | **$Ru_2$-8** |
| Empirical formula | $C_{78}H_{120}Cl_8N_2O_6P_4Ru_2$ |
| Formula weight | 1791.37 |
| Temperature/K | 100.0 |
| Crystal system | triclinic |
| Space group | *P*-1 |
| a/Å | 11.916(8) |
| b/Å | 17.612(15) |
| c/Å | 22.062(17) |
| α/° | 74.73(6) |
| β/° | 80.32(6) |
| γ/° | 77.17(6) |
| Volume/Å$^3$ | 4326(6) |
| Z | 2 |
| $\rho_{calc}$g/cm$^3$ | 1.375 |
| μ/mm$^{-1}$ | 0.719 |
| F(000) | 1868.0 |
| Crystal size/mm$^3$ | 0.25 × 0.167 × 0.1 |
| Radiation | MoKα (λ = 0.71073) |
| 2Θ range for data collection/° | 3.82 to 55.218 |
| Index ranges | $-12 \leq h \leq 15$, $-22 \leq k \leq 22$, $-28 \leq l \leq 28$ |
| Reflections collected | 39161 |
| Independent reflections | 19794 [$R_{int}$ = 0.0680, $R_{sigma}$ = 0.0967] |
| Data/restraints/parameters | 19794/13/939 |
| Goodness-of-fit on F$^2$ | 1.066 |
| Final R indexes [I>=2σ (I)] | $R_1$ = 0.0676, w$R_2$ = 0.1619 |
| Final R indexes [all data] | $R_1$ = 0.1301, w$R_2$ = 0.2163 |
| Largest diff. peak/hole / e Å$^{-3}$ | 1.86/-1.93 |

**Electrochemistry**

Compound **1**:

**Figure S34.** Cyclic voltammogram of **1** at 100 mV/s with 0.1 M $TBAPF_6$ supporting electrolyte in THF at 295(±3) K.

Complex **$Ru_2$-3**:

**Figure S35.** Cyclic (left) and square-wave voltammograms (right) of **$Ru_2$-3** at 100 mV/s with 0.1 M $TBAPF_6$ supporting electrolyte in THF at 295(±3) K.

**Figure S36.** Simulation of cyclic voltammogram of **$Ru_2$-3** for its two consecutive oxidations (100 mV/s with 0.1 M $TBAPF_6$ supporting electrolyte in THF at 295(±3) K). The red line is the experimental CV at 800 mV/s and black circle line is the simulated one.

**Figure S37.** Cyclic voltammogram of **$Ru_2$-3** at 100 mV/s with 0.1 M $TBAPF_6$ supporting electrolyte in dichloromethane at 295(±3) K. The inset voltammogram of **$Ru_2$-3** elaborates reduction wave only.

Complex **$Ru_2$-4**:

**Figure S38.** Cyclic (left) and square-wave voltammograms (right) of **$Ru_2$-4** at 100 mV/s with 0.1 M $TBAPF_6$ supporting electrolyte in THF at 295(±3) K.

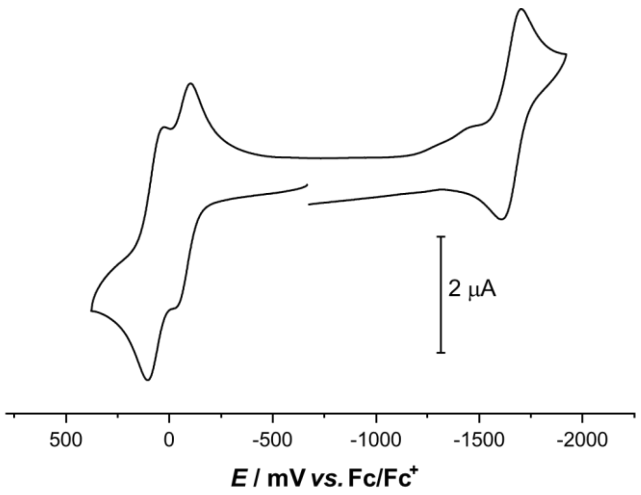


**Figure S39.** Cyclic voltammogram of **$Ru_2$-4** at 100 mV/s with 0.1 M $TBAPF_6$ supporting electrolyte in dichloromethane at 295(±3) K.

Compound **5**:

**Figure S40.** Cyclic voltammogram of **5** at 100 mV/s with 0.1 M $TBAPF_6$ supporting electrolyte in THF at 295(±3) K.

Complex **$Ru_2$-7:**

**Figure S41.** Cyclic (left) and square-wave voltammograms (right) of **$Ru_2$-7** at 100 mV/s with 0.1 M $TBAPF_6$ supporting electrolyte in THF at 295(±3) K.

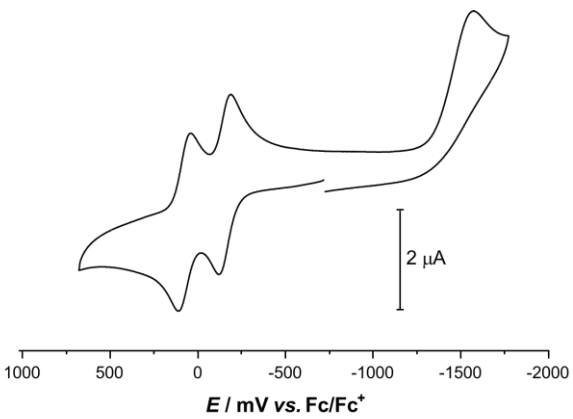


**Figure S42.** Cyclic voltammogram of **$Ru_2$-7** at 100 mV/s with 0.1 M $TBAPF_6$ supporting electrolyte in dichloromethane at 295(±3) K.

Complex **Ru$_2$-8:**

**Figure S43.** Cyclic (left) and square-wave voltammograms (right) of **Ru$_2$-8** at 100 mV/s with 0.1 M TBAPF$_6$ supporting electrolyte in THF at 295(±3) K.

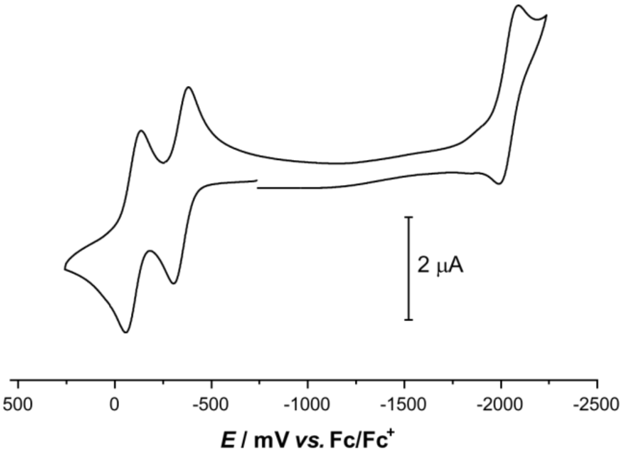


**Figure S44.** Cyclic voltammogram of **Ru$_2$-8** at 100 mV/s with 0.1 M TBAPF$_6$ supporting electrolyte in dichloromethane at 295(±3) K.

Complex **Ru$_2$-10:**

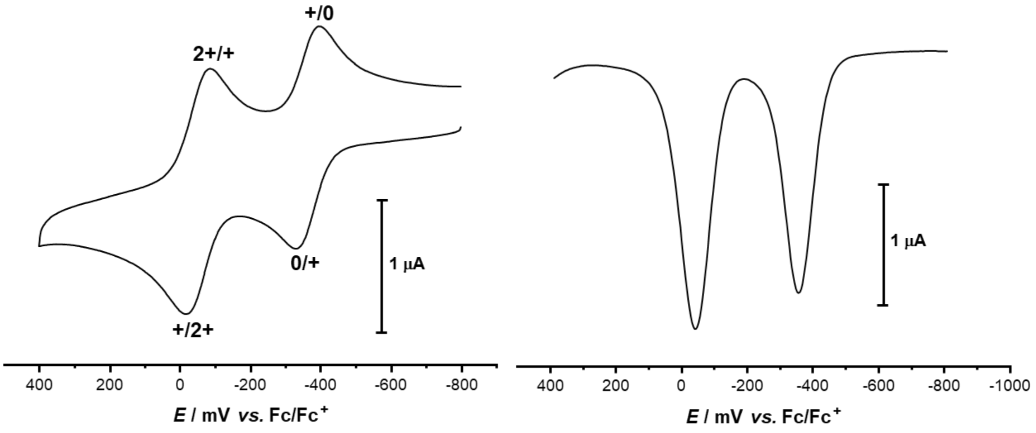


**Figure S45.** Cyclic (left) and square-wave voltammograms (right) of **Ru$_2$-10** at 100 mV/s with 0.1 M TBAPF$_6$ supporting electrolyte in $CH_2Cl_2$ at 295(±3) K.

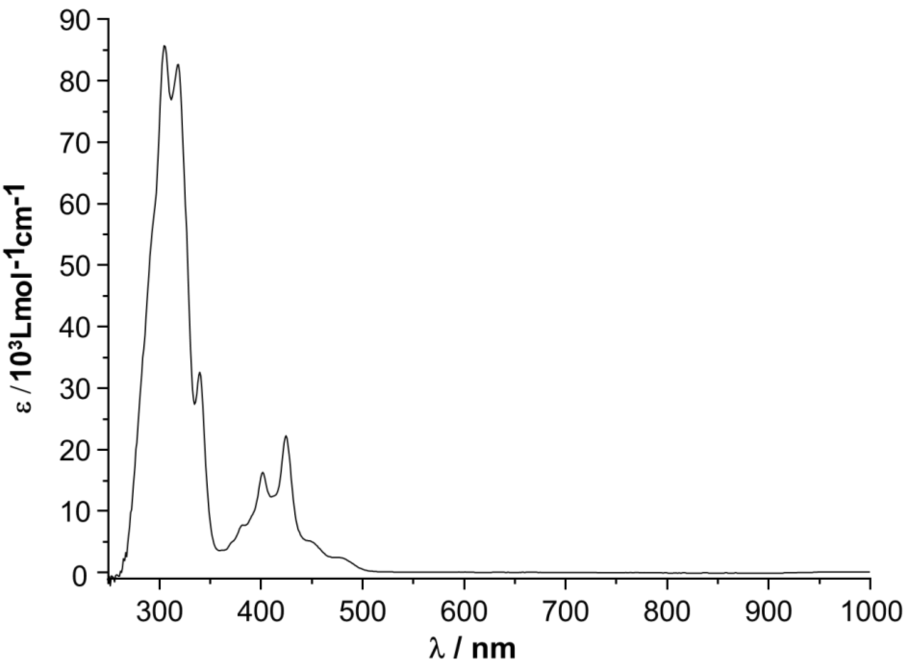


**Figure S46.** UV-vis spectrum of TMS-protected alkyne **1** in dichloromethane at 295(±3) K.

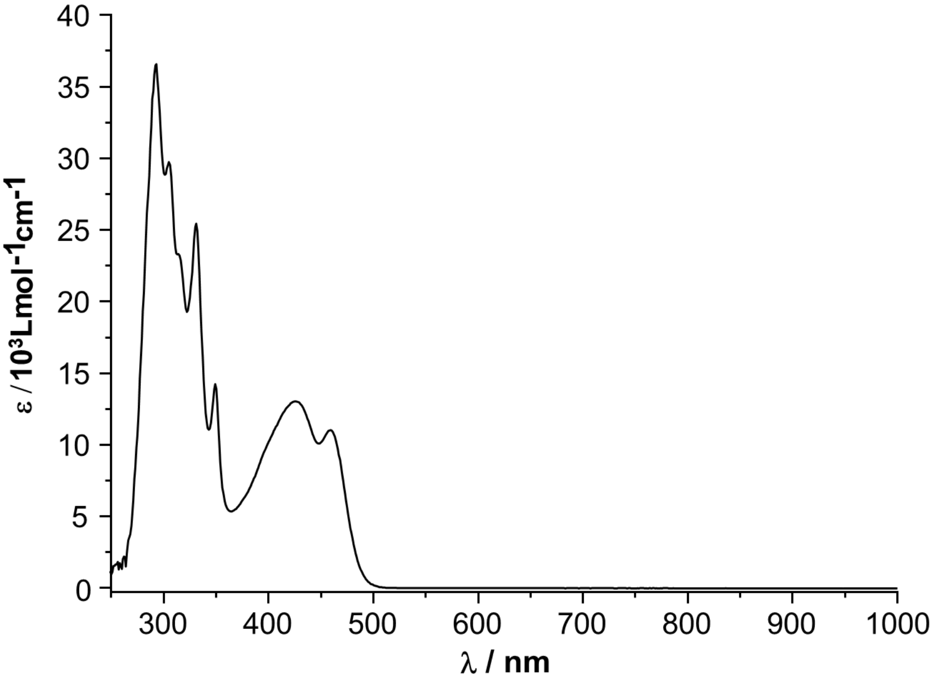


**Figure S47.** UV-vis spectrum of TMS-protected alkyne **5** in dichloromethane at 295(±3) K.

**Spectroelectrochemistry**

Complex **$Ru_2$-3**:

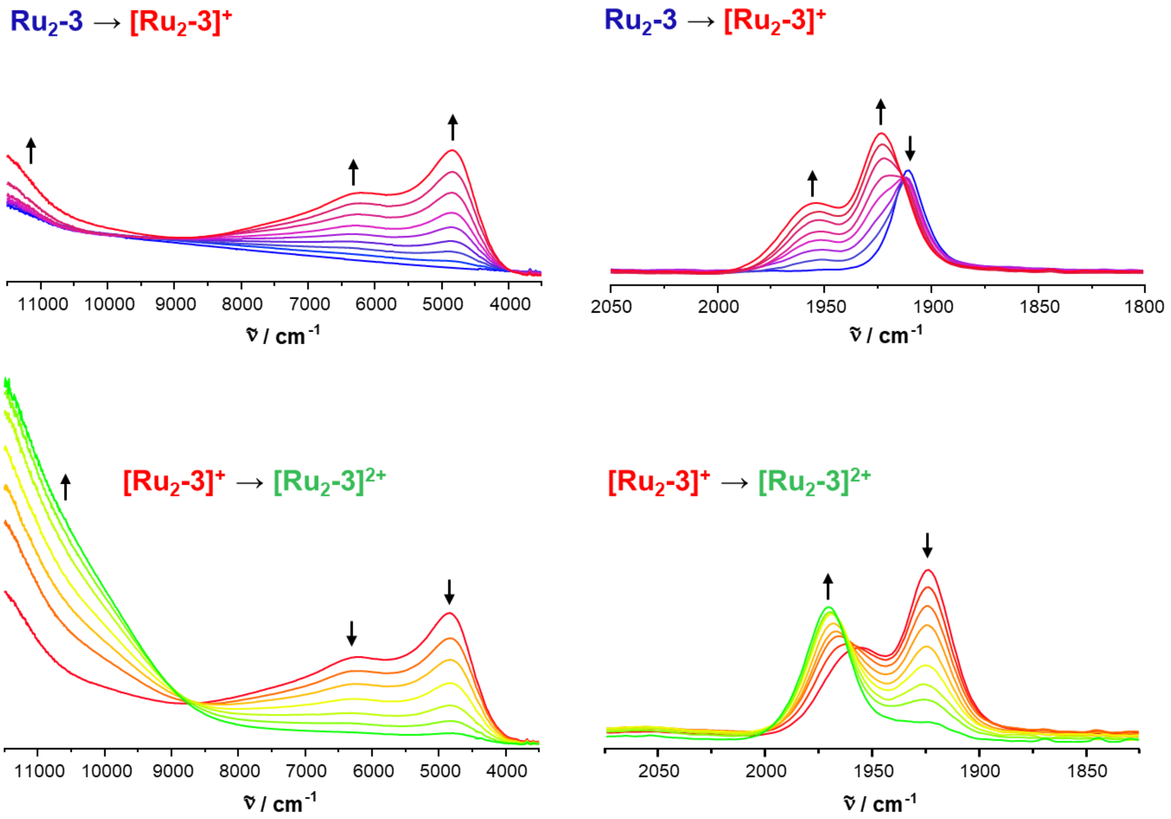


**Figure S48.** Changes in the NIR (left) and mid-IR [Ru(CO)] (right) spectra of **$Ru_2$-3** upon the first oxidation from **$Ru_2$-3** to [**$Ru_2$-3**]$^+$ (top) and the second oxidation from [**$Ru_2$-3**]$^+$ to [**$Ru_2$-3**]$^{2+}$ (bottom) measured in $CH_2Cl_2$, 0.1 M $TBAPF_6$ (298 K).

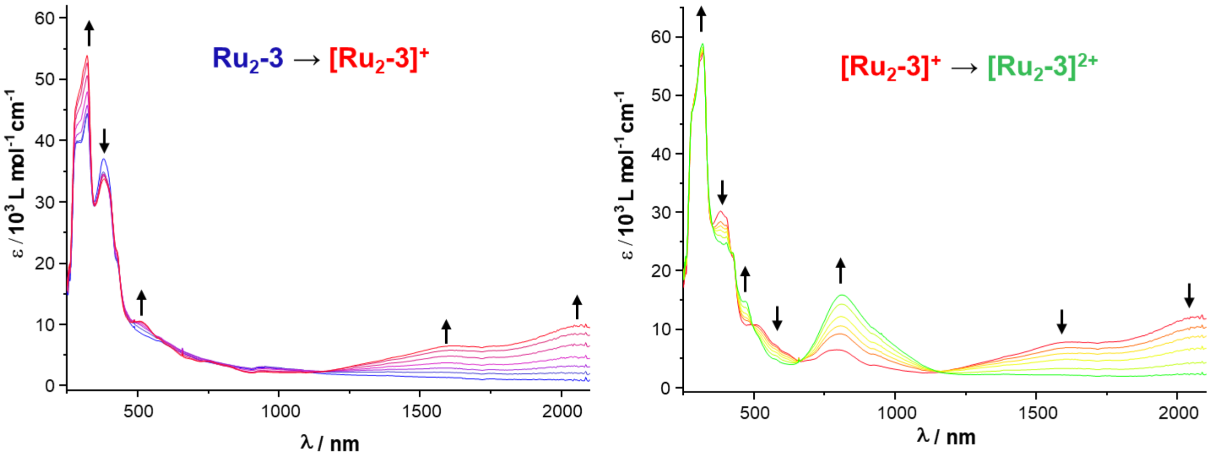


**Figure S49.** Changes in the UV/vis/NIR spectra of **$Ru_2$-3** upon the first oxidation from **$Ru_2$-3** to [**$Ru_2$-3**]$^+$ (left) and the second oxidation from [**$Ru_2$-3**]$^+$ to [**$Ru_2$-3**]$^{2+}$ (right) measured in $CH_2Cl_2$, 0.1 M $TBAPF_6$ (298 K).

Complex **$Ru_2$-4**:

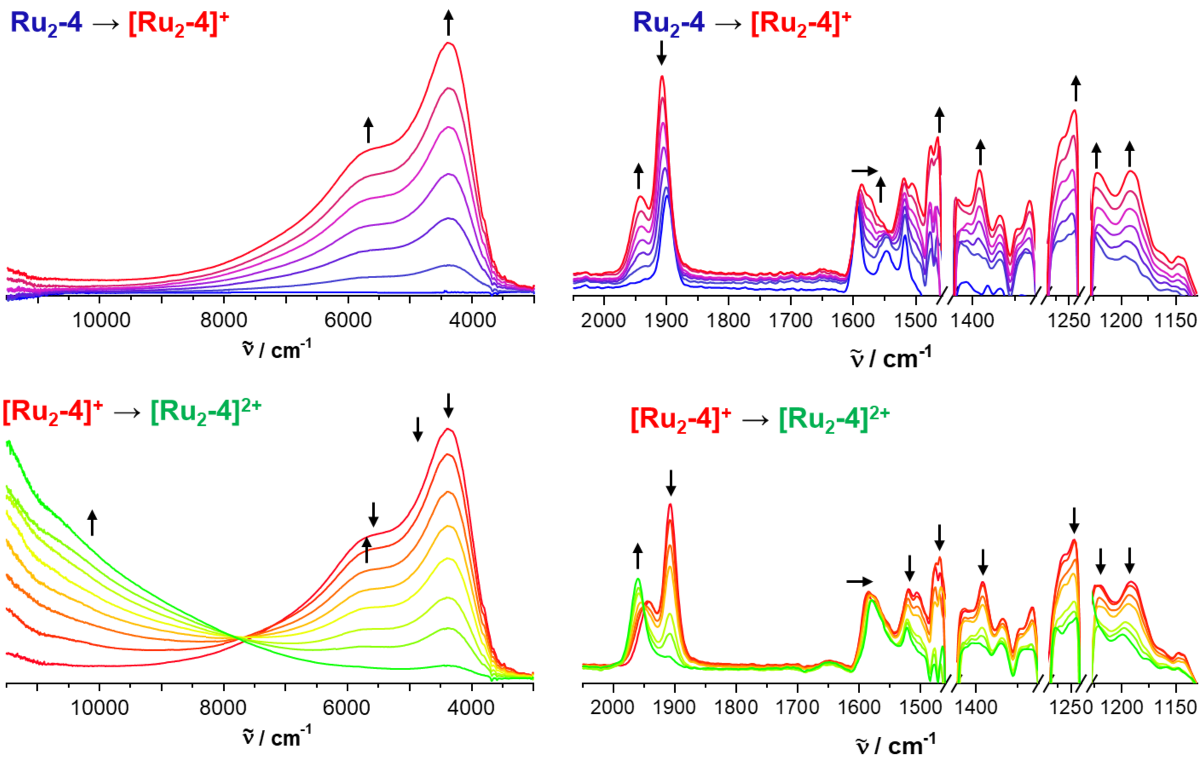


**Figure S50.** Changes in the NIR (left) and mid-IR [Ru(CO)] and arene backbone (right) spectra of **$Ru_2$-4** upon the first oxidation from **$Ru_2$-4** to [**$Ru_2$-4**]$^+$ (top) and the second oxidation from [**$Ru_2$-4**]$^+$ to [**$Ru_2$-4**]$^{2+}$ (bottom) measured in $CH_2Cl_2$, 0.1 M $TBAPF_6$ (298 K).

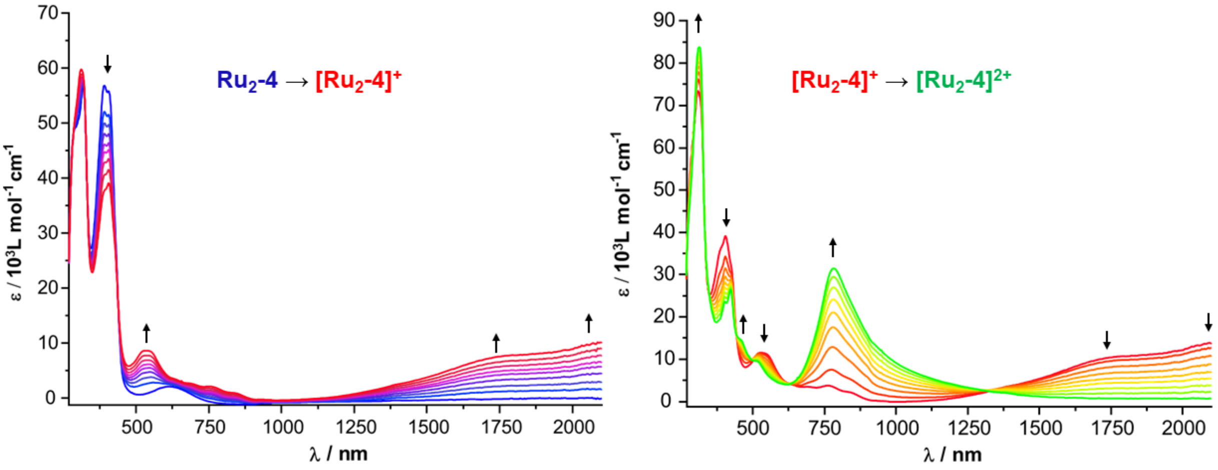


**Figure S51.** Changes in the UV/vis/NIR spectrum of **$Ru_2$-4** upon the first oxidation from **$Ru_2$-4** to [**$Ru_2$-4**]$^+$ (left) and the second oxidation from [**$Ru_2$-4**]$^+$ to [**$Ru_2$-4**]$^{2+}$ (right) measured in $CH_2Cl_2$, 0.1 M $TBAPF_6$ (298 K).

Complex **Ru$_2$-7**:

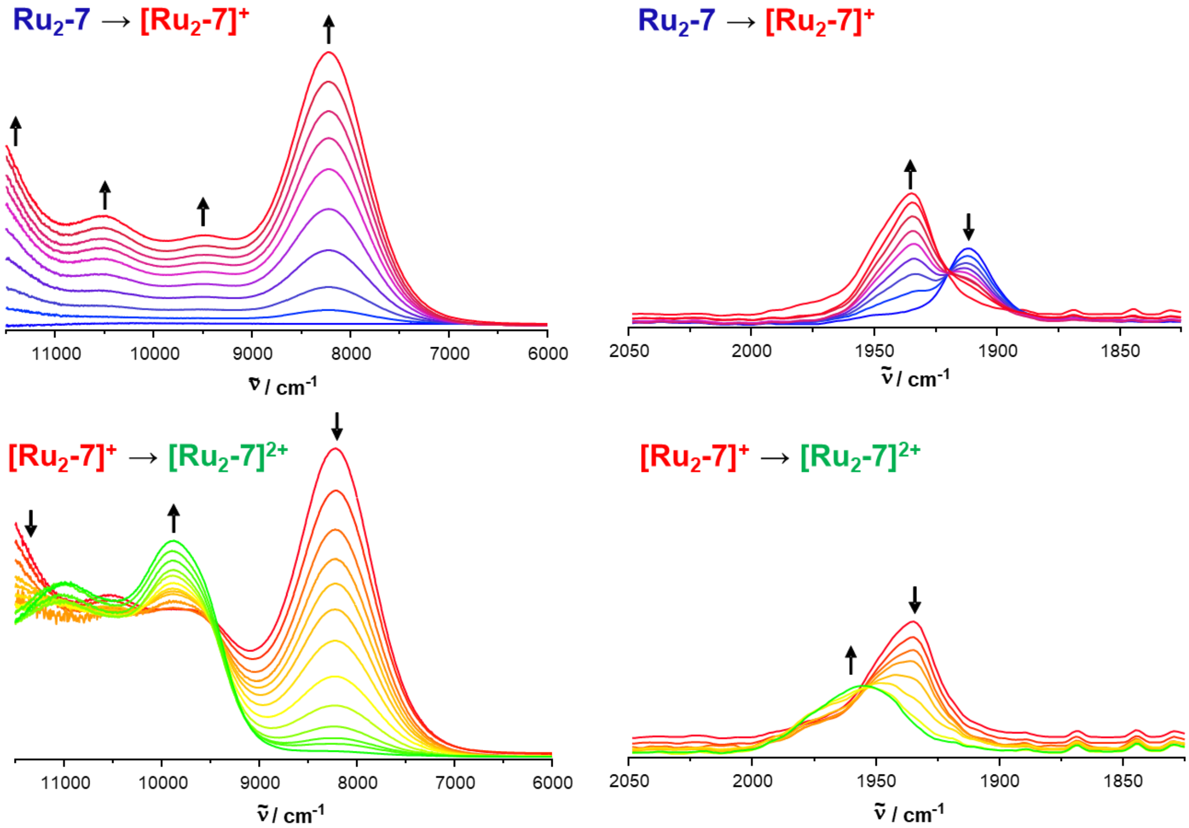


**Figure S52.** Changes in the NIR (left) and mid-IR [Ru(CO)] (right) spectra of **Ru$_2$-7** upon the first oxidation from **Ru$_2$-7** to [**Ru$_2$-7**]$^+$ (top) and the second oxidation from [**Ru$_2$-7**]$^+$ to [**Ru$_2$-7**]$^{2+}$ (bottom) measured in $CH_2Cl_2$, 0.1 M $TBAPF_6$ (298 K).

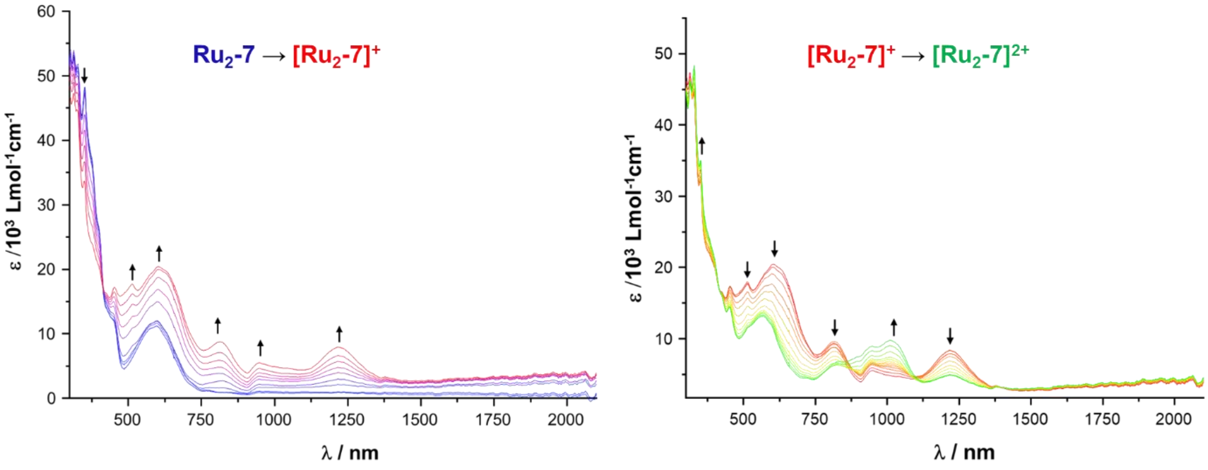


**Figure S53.** Changes in the UV/vis/NIR spectrum of **Ru$_2$-7** upon the first oxidation from **Ru$_2$-7** to [**Ru$_2$-7**]$^+$ (left) and the second oxidation from [**Ru$_2$-7**]$^+$ to [**Ru$_2$-7**]$^{2+}$ (right) measured in $CH_2Cl_2$, 0.1 M $TBAPF_6$ (298 K).

Complex **Ru$_2$-8**:

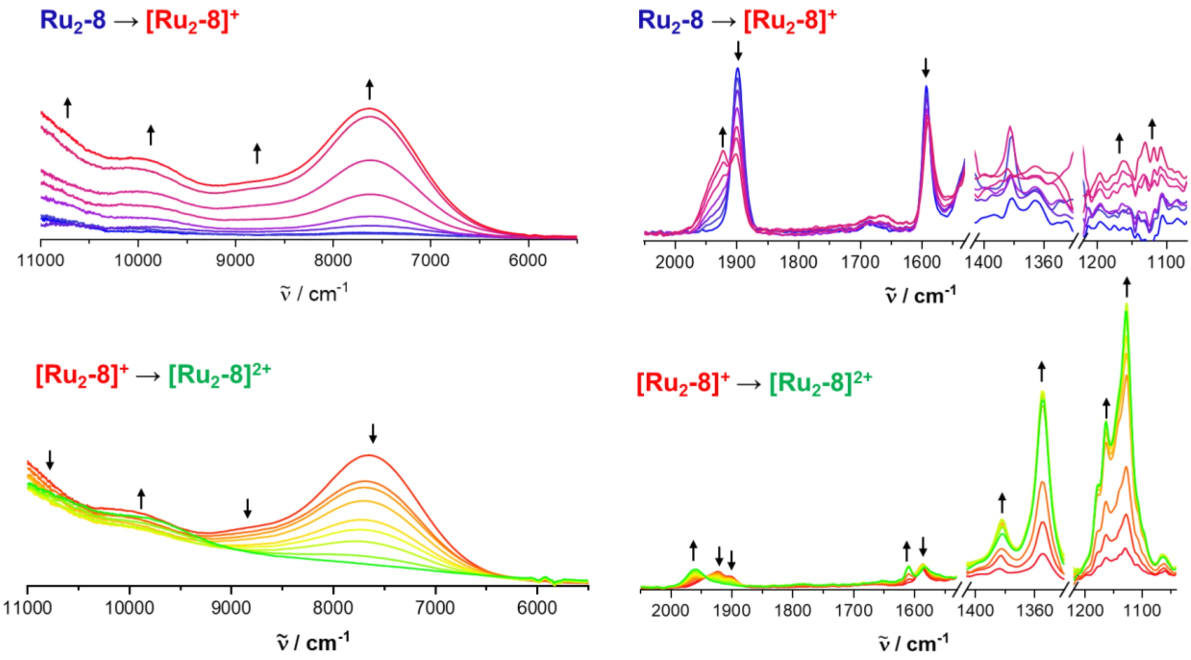


**Figure S54.** Changes in the NIR (left) and mid-IR [Ru(CO)] and arene backbone (right) spectra of **Ru$_2$-8** upon the first oxidation from **Ru$_2$-8** to [**Ru$_2$-8**]$^+$ (top) and the second oxidation from [**Ru$_2$-8**]$^+$ to [**Ru$_2$-8**]$^{2+}$ (bottom) measured in $CH_2Cl_2$, 0.1 M $TBAPF_6$ (298 K).

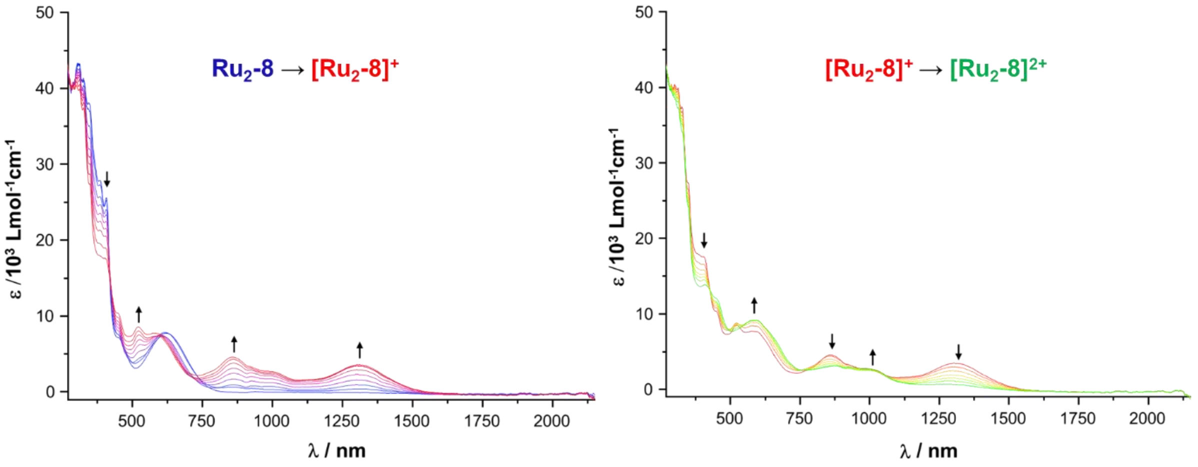


**Figure S55.** Changes in the UV/vis/NIR spectrum of **Ru$_2$-8** upon the first oxidation from **Ru$_2$-8** to [**Ru$_2$-8**]$^+$ (left) and second oxidation from [**Ru$_2$-8**]$^+$ to [**Ru$_2$-8**]$^{2+}$ (right) measured in $CH_2Cl_2$, 0.1 M $TBAPF_6$ (298 K).

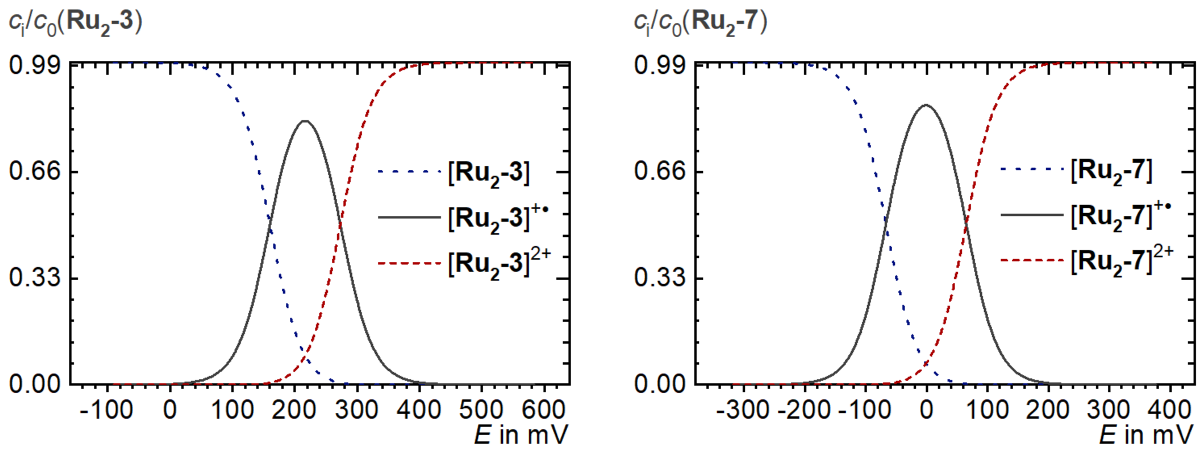


**Figure S56.** Relative concentration profiles for the neutral (dashed blue), monooxidized (black line) and dioxidized (dashed red) forms of **Ru$_2$-3** (left) and **Ru$_2$-7** (right) in dependence of the applied oxidation potential in a spectroelectrochemical experiment. At the maximum concentration of the monooxidized species the relative ratio is 0.09/0.82/0.09 for **Ru$_2$-3$^{0/+•/2+}$**, and 0.07/0.86/0.07 for **Ru$_2$-7$^{0/+•/2+}$**.

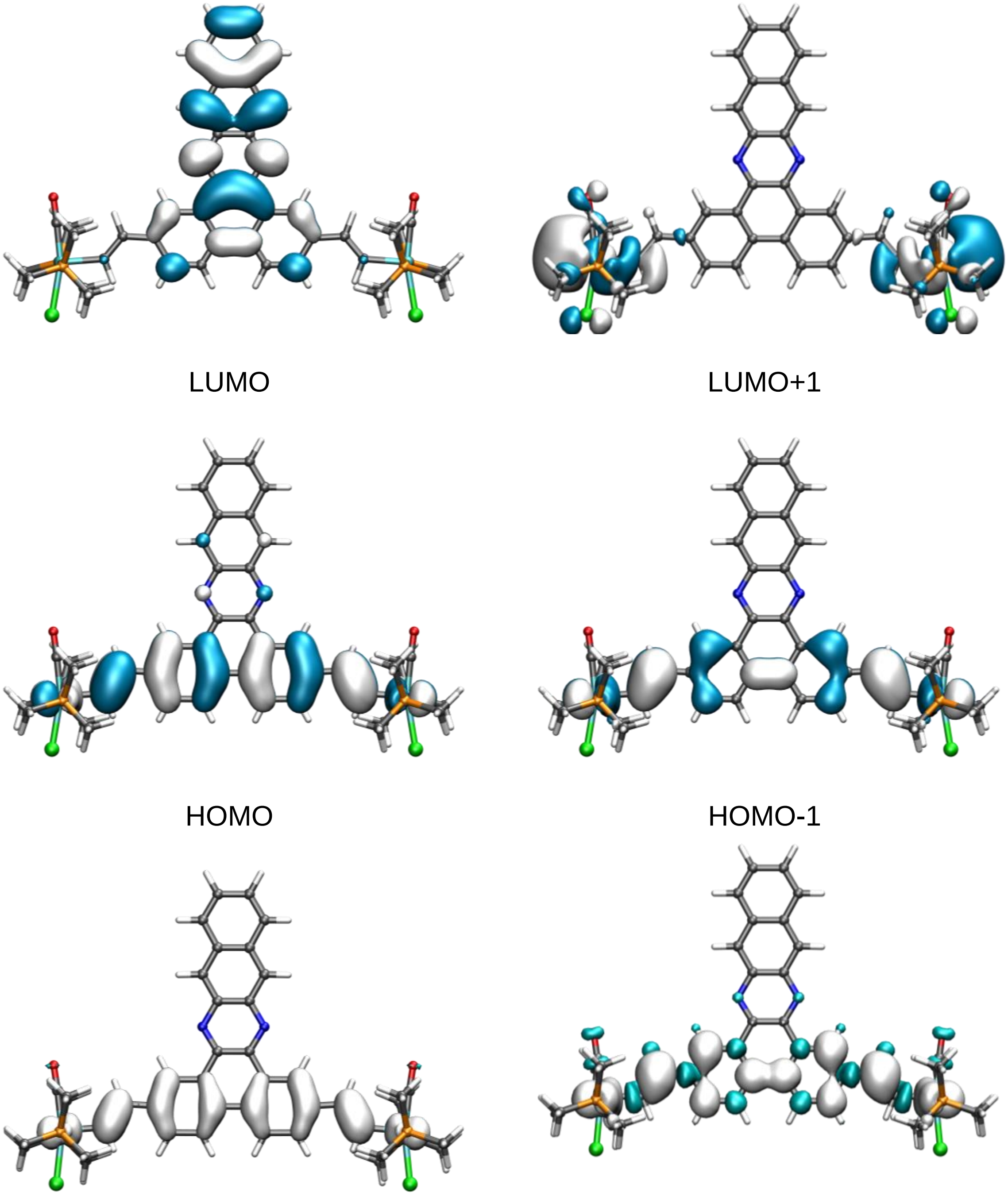


**Figure S57.** DFT/ PBE1PBE/6-31G(d)PCM($CH_2Cl$-$CH_2Cl$) calculated contour diagrams of complex **Ru$_2$-3** (HOMO, HOMO-1, LUMO and LUMO+1) (top and middle panel) and spin densities of **[Ru$_2$-3]$^+$** (bottom left) and the triplet state of **[Ru$_2$-3]$^{2+}$** (bottom right).

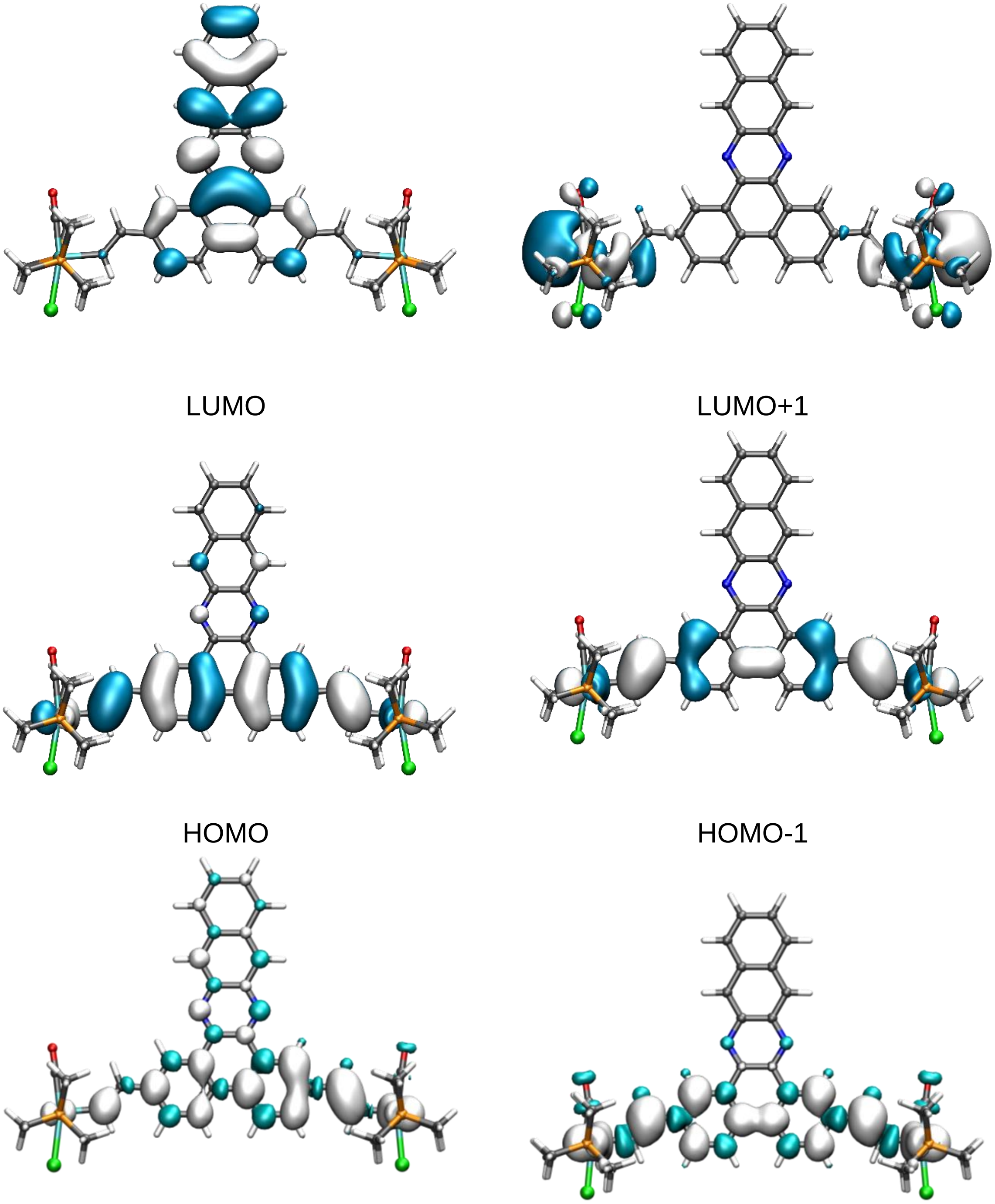


**Figure S58.** DFT/BLYP35/6-31G(d)PCM($CH_2Cl$-$CH_2Cl$) calculated contour diagrams of complex **$Ru_2$-3** (HOMO, HOMO-1, LUMO and LUMO+1) (top and middle panel) and spin densities of **$[Ru_2\text{-}3]^+$** (bottom left) and the triplet state of **$[Ru_2\text{-}3]^{2+}$** (bottom right).

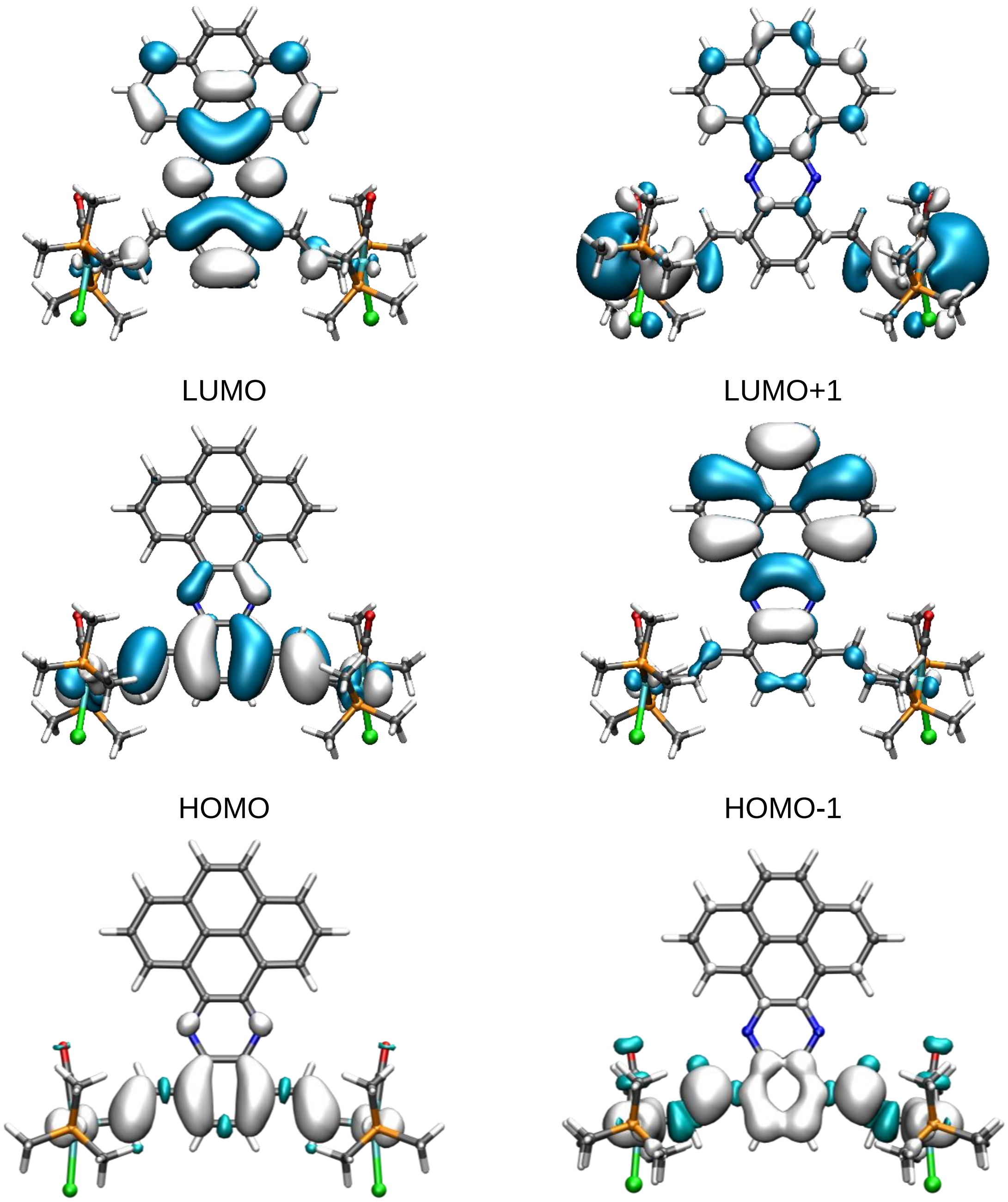


**Figure S59.** DFT/BLYP35/6-31G(d)PCM($CH_2Cl$-$CH_2Cl$) calculated contour diagrams of complex **Ru₂-7** (HOMO, HOMO-1, LUMO and LUMO+1) (top and middle panel) and spin densities of **[Ru₂-7]**$^{+}$ (bottom left) and the triplet state of **[Ru₂-7]**$^{2+}$ (bottom right).

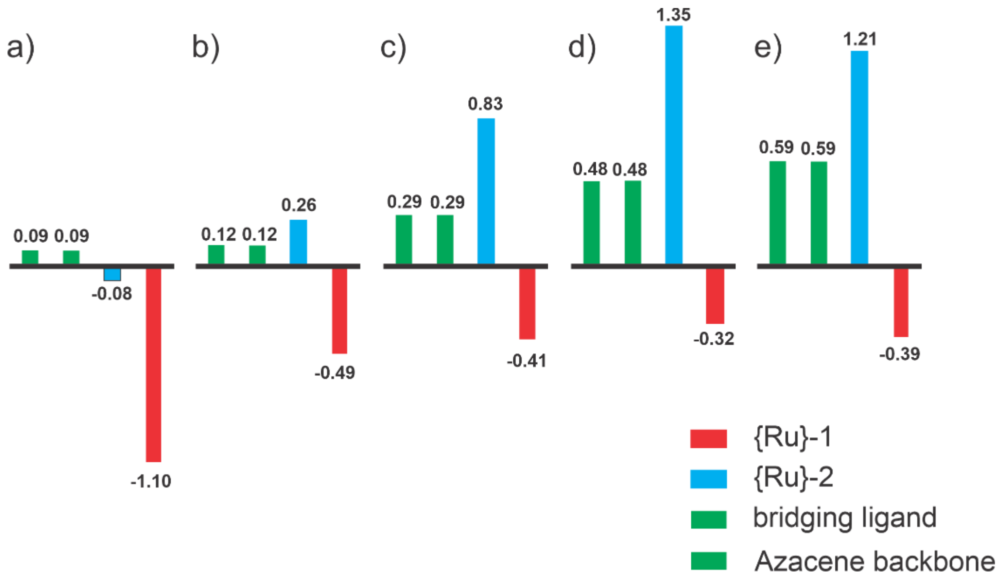


**Figure S60.** pbe1pbe-Calculated charge densities on the constituents of complex **Ru$_2$-3** in its **a**) monoanionic, **b**) neutral, **c**) monocationic, **d**) dicationic (singlet) and **e**) dicationic (triplet) states according to NBO-analysis. {Ru} = Ru(-CH=CH-)(CO)(Cl)(P$^i$Pr$_3$)$_2$.

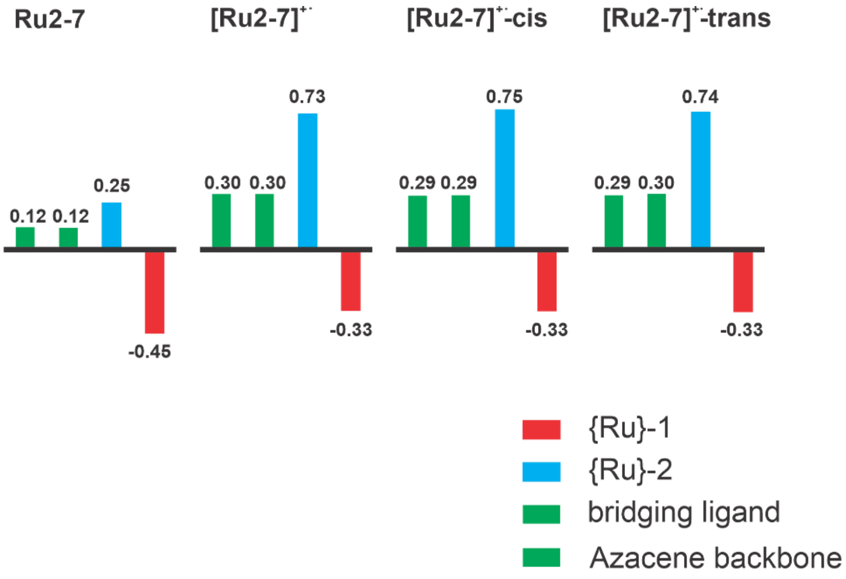


**Figure S61.** BLYP35-calculated charge densities on the constituents of complex **Ru$_2$-7** in its **a**) monoanionic, **b**) neutral, **c**) monocationic, **d**) dicationic (singlet) and **e**) dicationic (triplet) states according to NBO-analysis. {Ru} = Ru(-CH=CH-)(CO)(Cl)(P$^i$Pr$_3$)$_2$.

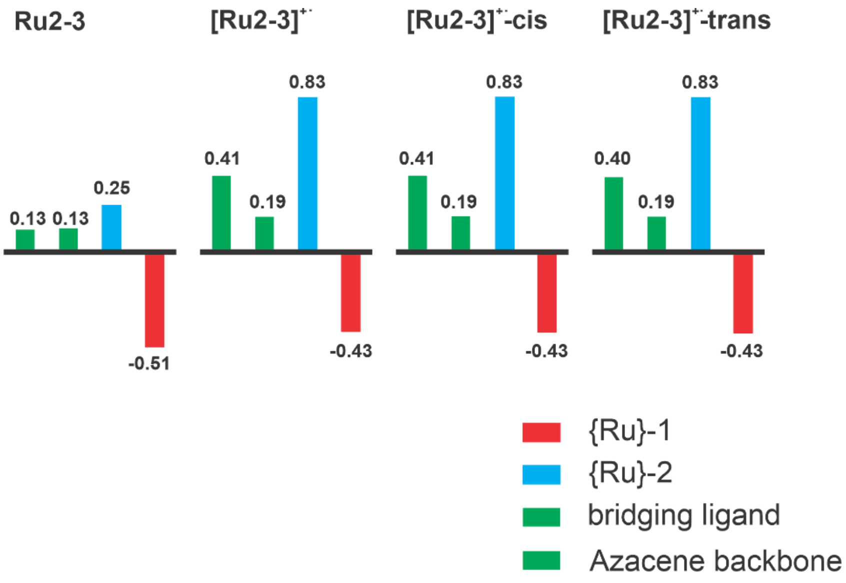


**Figure S62.** BLYP35-calculated charge densities on the constituents of complex **$Ru_2$-3** in its **a**) monoanionic, **b**) neutral, **c**) monocationic, **d**) dicationic (singlet) and **e**) dicationic (triplet) states according to NBO-analysis. {Ru} = Ru(-CH=CH-)(CO)(Cl)($P^iPr_3$)$_2$.

**Table S2.** NBO-Calculated (pbe1pbe) densities on the Ru moieties, bridging ligand and the azacene backbone of complexes **$Ru_2$-3** and **$Ru_2$-7** in their neutral, monocationic and dicationic states.

| NBO Charge distributions | | | | | | |
|---|---|---|---|---|---|---|
| | **$Ru_2$-3** | **$[Ru_2\text{-}3]^+$** | **$[Ru_2\text{-}3]^+$ (*cis*)** | **$[Ru_2\text{-}3]^+$ (*trans*)** | **$[Ru_2\text{-}3]^{2+}$ (singlet)** | **$[Ru_2\text{-}3]^{2+}$ (triplet)** |
| **$Ru(CO)(Cl)(P^iPr_3)_2$** | 0.12 | 0.29 | 0.29 | 0.28 | 0.48 | 0.59 |
| **$Ru(CO)(Cl)(P^iPr_3)_2$** | 0.12 | 0.29 | 0.29 | 0.30 | 0.48 | 0.59 |
| Bridging ligand | 0.26 | 0.83 | 0.83 | 0.83 | 1.35 | 1.21 |
| Azacene backbone | -0.49 | -0.41 | -0.41 | -0.41 | -0.32 | -0.39 |
| | | | | | | |
| Total charge | 0 | +1 | +1 | +1 | +2 | +2 |

| Differences of NBO charges between the charged and neutral states | | | | | | |
|---|---|---|---|---|---|---|
| | **$Ru_2$-3** | **$[Ru_2\text{-}3]^+$** | **$[Ru_2\text{-}3]^+$ (*cis*)** | **$[Ru_2\text{-}3]^+$ (*trans*)** | **$[Ru_2\text{-}3]^{2+}$ (singlet)** | **$[Ru_2\text{-}3]^{2+}$ (triplet)** |
| **$Ru(CO)(Cl)(P^iPr_3)_2$** | 0 | 0.17 | 0.17 | 0.16 | 0.36 | 0.47 |
| **$Ru(CO)(Cl)(P^iPr_3)_2$** | 0 | 0.17 | 0.17 | 0.18 | 0.36 | 0.47 |
| Bridging ligand | 0 | 0.57 | 0.57 | 0.57 | 1.09 | 0.95 |
| Azacene backbone | 0 | 0.08 | 0.08 | 0.08 | 0.18 | 0.11 |
| | | | | | | |
| Total charge | 0 | +1 | +1 | +1 | +2 | +2 |

**Table S2 continued**

| NBO Charges | | | | | | |
|---|---|---|---|---|---|---|
| | **$Ru_2$-7** | **[$Ru_2$-7]$^+$** | **[$Ru_2$-7]$^+$ (*cis*)** | **[$Ru_2$-7]$^+$ (*trans*)** | **[$Ru_2$-7]$^{2+}$ (singlet)** | **[$Ru_2$-7]$^{2+}$ (triplet)** |
| **Ru(CO)(Cl)($P^iPr_3$)$_2$** | 0.11 | 0.31 | 0.30 | 0.30 | 0.54 | 0.62 |
| **Ru(CO)(Cl)($P^iPr_3$)$_2$** | 0.11 | 0.31 | 0.30 | 0.30 | 0.54 | 0.62 |
| Bridging ligand | 0.24 | 0.72 | 0.74 | 0.73 | 1.15 | 0.98 |
| Azacene backbone | -0.46 | -0.34 | -0.34 | -0.33 | -0.24 | -0.22 |
| | | | | | | |
| Total charge | 0 | +1 | +1 | +1 | +2 | +2 |

| Differences of spin densities between the charged and neutral states | | | | | | |
|---|---|---|---|---|---|---|
| | **$Ru_2$-7** | **[$Ru_2$-7]$^+$** | **[$Ru_2$-7]$^+$ (*cis*)** | **[$Ru_2$-7]$^+$ (*trans*)** | **[$Ru_2$-7]$^{2+}$ (singlet)** | **[$Ru_2$-7]$^{2+}$ (triplet)** |
| **Ru(CO)(Cl)($P^iPr_3$)$_2$** | 0 | 0.20 | 0.19 | 0.19 | 0.43 | 0.51 |
| **Ru(CO)(Cl)($P^iPr_3$)$_2$** | 0 | 0.20 | 0.19 | 0.19 | 0.43 | 0.51 |
| Bridging ligand | 0 | 0.48 | 0.50 | 0.49 | 0.91 | 0.74 |
| Azacene backbone | 0 | 0.12 | 0.12 | 0.13 | 0.22 | 0.24 |
| | | | | | | |
| Total charge | 0 | +1 | +1 | +1 | +2 | +2 |

**Table S3.** NBO-Calculated (BLYP35) densities on the Ru moieties, bridging ligand and the azacene backbone of complexes **$Ru_2$-3** and **$Ru_2$-7** in their neutral, monocationic and dicationic states.

**NBO Charge distributions**

| | **$Ru_2$-7** | **[$Ru_2$-7]$^+$** | **[$Ru_2$-7]$^+$ (*cis*)** | **[$Ru_2$-7]$^+$ (*trans*)** |
|---|---|---|---|---|
| **Ru(CO)(Cl)($P^iPr_3$)$_2$** | 0.12 | 0.30 | 0.29 | 0.29 |
| **Ru(CO)(Cl)($P^iPr_3$)$_2$** | 0.12 | 0.30 | 0.29 | 0.30 |
| Bridging ligand | 0.21 | 0.73 | 0.75 | 0.74 |
| Azacene backbone | -0.45 | -0.33 | -0.33 | -0.33 |
| | | | | |
| Total charge | 0 | +1 | +1 | +1 |

**Differences of NBO charges between the charged and neutral states**

| | **$Ru_2$-7** | **[$Ru_2$-7]$^+$** | **[$Ru_2$-7]$^+$ (*cis*)** | **[$Ru_2$-7]$^+$ (*trans*)** |
|---|---|---|---|---|
| **Ru(CO)(Cl)($P^iPr_3$)$_2$** | 0 | 0.18 | 0.17 | 0.17 |
| **Ru(CO)(Cl)($P^iPr_3$)$_2$** | 0 | 0.18 | 0.17 | 0.17 |
| Bridging ligand | 0 | 0.53 | 0.54 | 0.54 |
| Azacene backbone | 0 | 0.12 | 0.12 | 0.12 |
| | | | | |
| Total charge | 0 | +1 | +1 | +1 |

**Table S3 continued**

| NBO Charges | | | | |
|---|---|---|---|---|
| | **$Ru_2$-3** | **$[Ru_2\text{-}3]^+$** | **$[Ru_2\text{-}3]^+$ (*cis*)** | **$[Ru_2\text{-}3]^+$ (*trans*)** |
| **Ru(CO)(Cl)($P^iPr_3$)$_2$** | 0.13 | 0.41 | 0.41 | 0.40 |
| **Ru(CO)(Cl)($P^iPr_3$)$_2$** | 0.13 | 0.19 | 0.19 | 0.19 |
| Bridging ligand | 0.25 | 0.83 | 0.83 | 0.83 |
| Azacene backbone | -0.51 | -0.43 | -0.43 | -0.43 |
| | | | | |
| Total charge | 0 | +1 | +1 | +1 |

| Differences of spin densities between the charged and neutral states | | | | |
|---|---|---|---|---|
| | **$Ru_2$-3** | **$[Ru_2\text{-}3]^+$** | **$[Ru_2\text{-}3]^+$ (*cis*)** | **$[Ru_2\text{-}3]^+$ (*trans*)** |
| **Ru(CO)(Cl)($P^iPr_3$)$_2$** | 0 | 0.28 | 0.28 | 0.27 |
| **Ru(CO)(Cl)($P^iPr_3$)$_2$** | 0 | 0.06 | 0.06 | 0.06 |
| Bridging ligand | 0 | 0.58 | 0.58 | 0.58 |
| Azacene backbone | 0 | 0.08 | 0.08 | 0.08 |
| | | | | |
| Total charge | 0 | +1 | +1 | +1 |

**Table S4.** pbe1pbe (left column) and BLYP35 (right column) computed data for the Ru(CO) bands of $PMe_3$ models of complexes **$[Ru_2\text{-}3]^{n+}$** and **$[Ru_2\text{-}7]^{n+}$** (n = 0, 1, 2) as their different conformers (n = 1) or in their diffrerent spin states (n = 2). CO data are provided in $cm^{-1}$ and scaled with a factor of 0.951.

| **pbe1pbe** | | | | **BLYP35** | | | |
|---|---|---|---|---|---|---|---|
| | ~<br>(CO) | Δ ~<br>(CO) | intensity | | ~<br>(CO) | Δ ~<br>(CO) | intensity |
| **$Ru_2$-3** | 1912 | - | 522 | **$Ru_2$-3** | 1911 | | 517 |
| | 1913 | - | 1946 | | 1912 | | 2234 |
| **$[Ru_2\text{-}3]^{\bullet+}$-*cis*** | 1928 | 16 | 13269 | **$[Ru_2\text{-}3]^{\bullet+}$ (*cis*)** | 1920 | 9 | 3880 |
| | 1939 | 26 | 2316 | | 1949 | 37 | 6389 |
| **$[Ru_2\text{-}3]^{\bullet+}$-*trans*** | 1928 | 16 | 14826 | **$[Ru_2\text{-}3]^{\bullet+}$ (*trans*)** | 1920 | 9 | 4317 |
| | 1940 | 27 | 14 | | 1949 | 37 | 5293 |
| **$[Ru_2\text{-}3]^{\bullet+}$** | 1928 | 16 | 13553 | **$[Ru_2\text{-}3]^{\bullet+}$** | 1921 | 10 | 3600 |
| | 1940 | 27 | 1610 | | 1950 | 39 | 5643 |
| **$[Ru_2\text{-}3]^{2+}$ (S)** | 1961 | 49 | 5819 | | | | |
| | 1966 | 53 | 1344 | | | | |
| **$[Ru_2\text{-}3]^{2+}$ (OSS)** | 1961 | 49 | 5816 | | | | |
| | 1966 | 53 | 1344 | | | | |
| **$[Ru_2\text{-}3]^{2+}$ (T)** | 1973 | 61 | 1688 | | | | |
| | 1975 | 62 | 1569 | | | | |
| | | | | | | | |
| | ~<br>(CO) | Δ ~<br>(CO) | intensity | | ~<br>(CO) | Δ ~<br>(CO) | intensity |
| **$Ru_2$-7** | 1913 | | 538 | **$Ru_2$-7** | 1909 | | 862 |
| | 1914 | | 1969 | | 1910 | | 1858 |
| **$[Ru_2\text{-}7]^{\bullet+}$ (*cis*)** | 1938 | 25 | 3029 | **$[Ru_2\text{-}7]^{\bullet+}$ (*cis*)** | 1929 | 20 | 2982 |
| | 1945 | 31 | 2695 | | 1937 | 27 | 2907 |
| **$[Ru_2\text{-}7]^{\bullet+}$ (*trans*)** | 1941 | 28 | 4638 | **$[Ru_2\text{-}7]^{\bullet+}$ (*trans*)** | 1932 | 23 | 4780 |
| | 1948 | 34 | 40 | | 1939 | 29 | 150 |
| **$[Ru_2\text{-}7]^{\bullet+}$** | 1942 | 29 | 2567 | **$[Ru_2\text{-}7]^{\bullet+}$** | 1935 | 26 | 2916 |
| | 1949 | 35 | 1835 | | 1941 | 31 | 1797 |
| **$[Ru_2\text{-}7]^{2+}$ (S)** | 1970 | 57 | 1736 | | | | |
| | 1974 | 60 | 1529 | | | | |
| **$[Ru_2\text{-}7]^{2+}$ (OSS)** | 1970 | 57 | 1823 | | | | |
| | 1975 | 61 | 1508 | | | | |
| **$[Ru_2\text{-}7]^{2+}$ (T)** | 1971 | 58 | 1578 | | | | |
| | 1973 | 59 | 510 | | | | |

## UV-vis-NIR Spectra from TD-DFT calculations

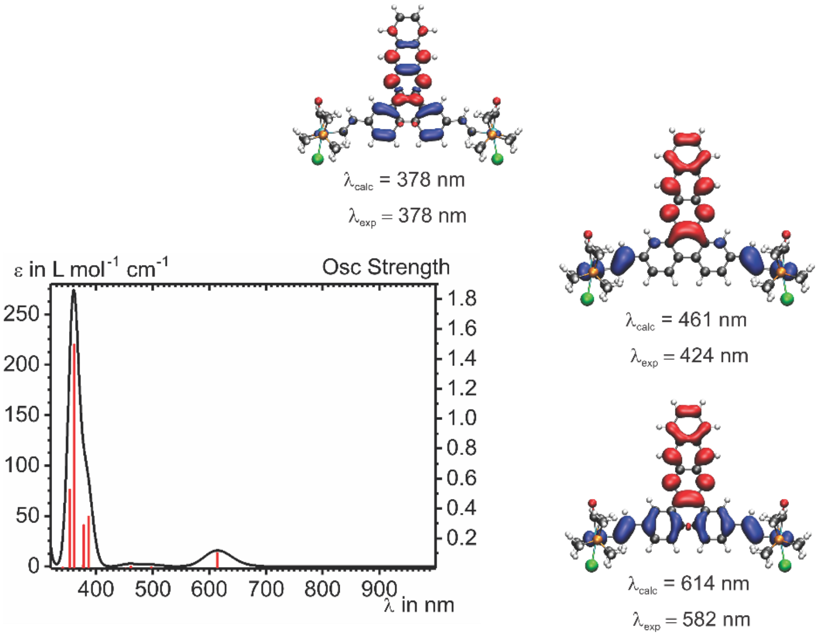


**Figure S63.** Calculated (pbe1pbe) UV-vis-NIR spectrum of **$Ru_2$-3** obtained from the TD-DFT calculations. The red colour marks an increase, the blue colour a decrease of electron density during the corresponding electronic transition.

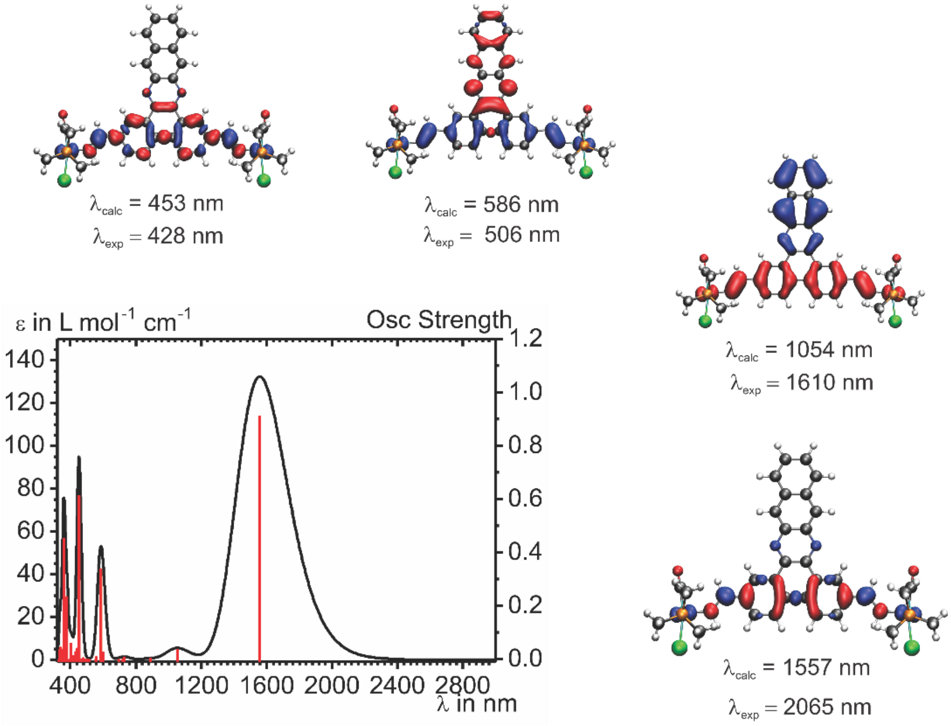


**Figure S64.** Calculated (pbe1pbe) UV-vis-NIR spectrum of the *Ru trans-trans* conformer of **$[Ru_2\text{-}3]^+$** obtained from TD-DFT calculations. The red colour marks an increase, the blue colour a decrease of electron density during the corresponding electronic transition.

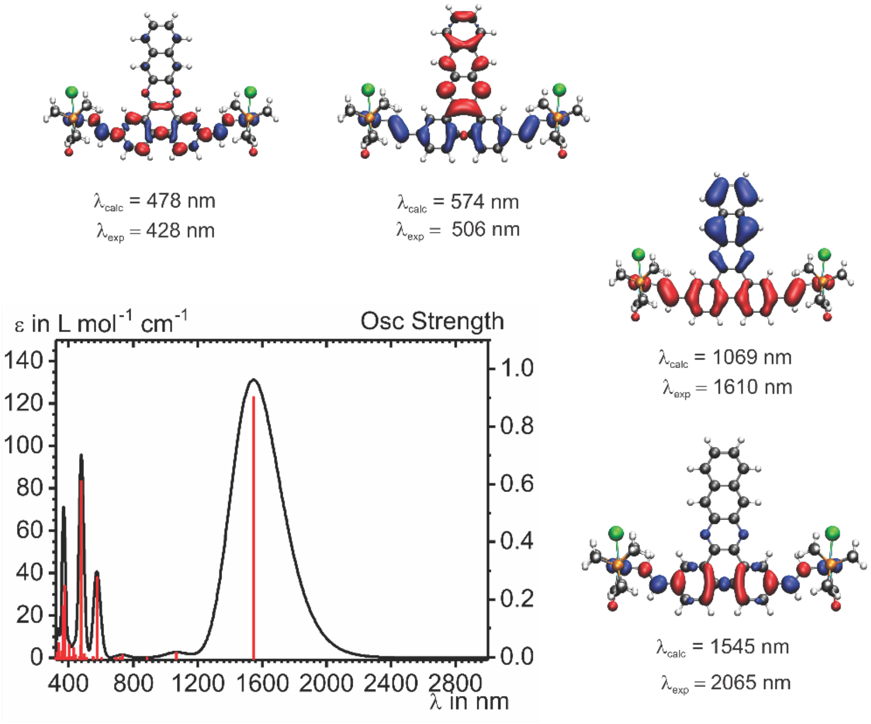


**Figure S65.** Calculated (pbe1pbe) UV-vis-NIR spectrum of the *Ru cis-cis* conformer of **[$Ru_2$-3]$^+$** obtained from TD-DFT calculations. The red colour marks an increase, the blue colour a decrease of electron density during the corresponding electronic transition.

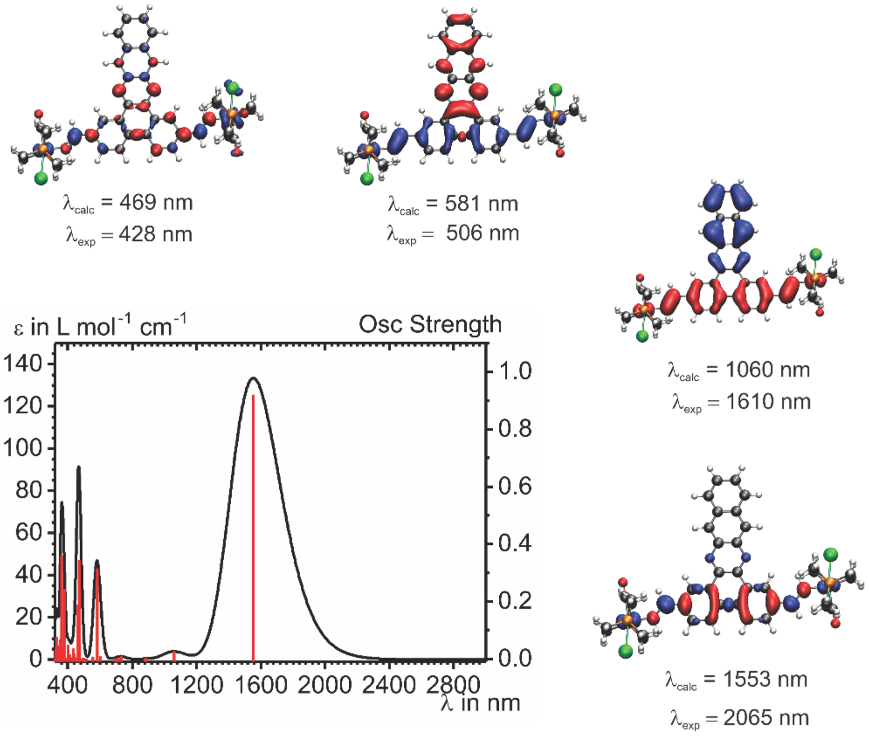

**Figure S66.** Calculated (pbe1pbe) UV-vis-NIR spectrum of the *Ru cis/trans* conformer of **[Ru$_2$-3]$^+$** obtained from TD-DFT calculations. The red colour marks an increase, the blue colour a decrease of electron density during the corresponding electronic transition.

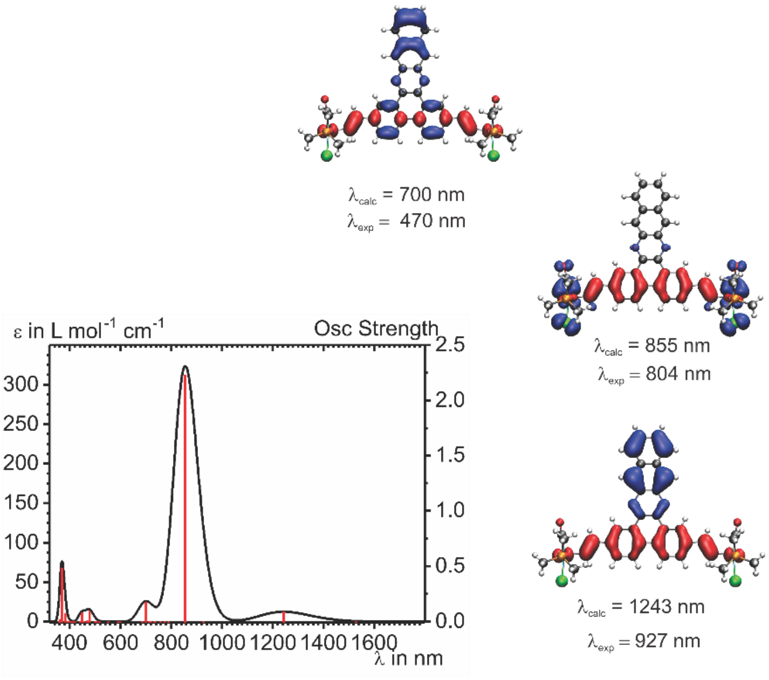


**Figure S67.** Calculated (pbe1pbe) UV-vis-NIR spectrum of **[Ru$_2$-3]$^{2+}$** in its singlet state obtained from the TD-DFT calculations. The red colour marks an increase, the blue colour a decrease of electron density during the corresponding electronic transition.

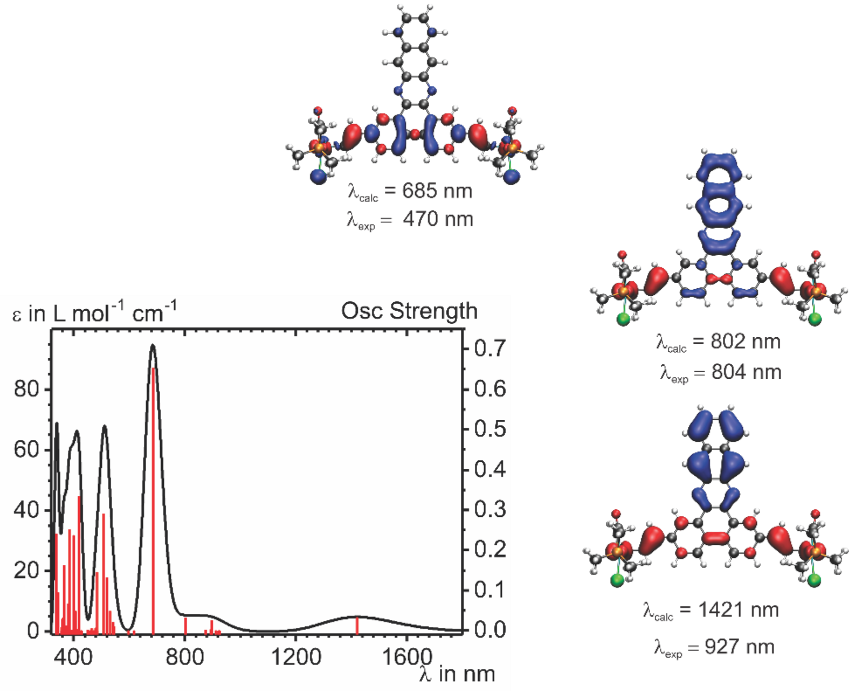

**Figure S68.** Calculated (pbe1pbe) UV-vis-NIR spectrum of **[Ru2-3]**$^{2+}$ in its triplet state obtained from the TD-DFT calculations. The red colour marks an increase, the blue colour a decrease of electron density during the corresponding electronic transition.

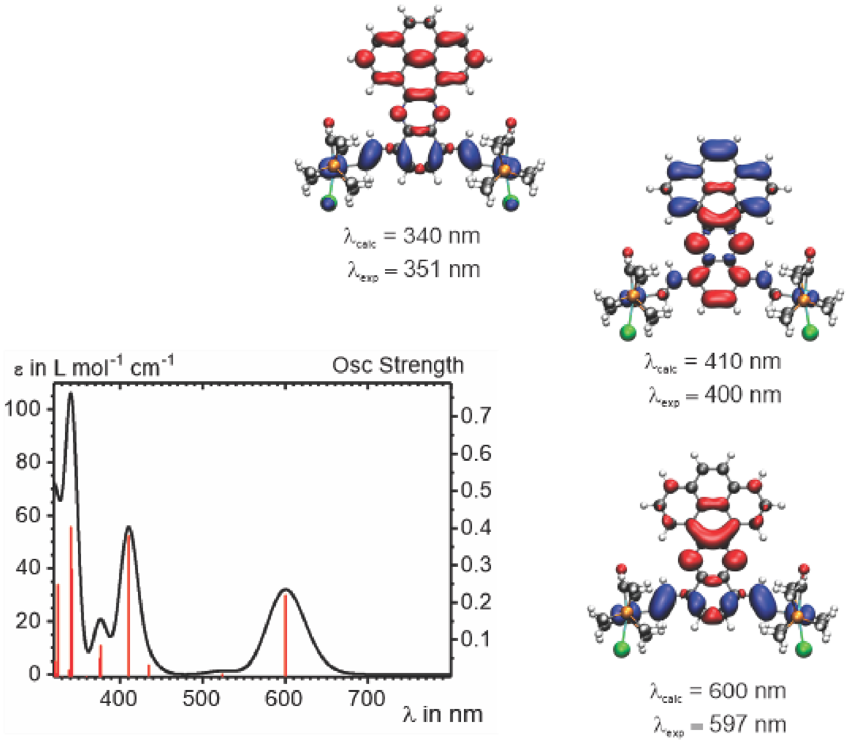


**Figure S69.** Calculated (pbe1pbe) UV-vis-NIR spectrum of **Ru2-7** obtained from the TD-DFT calculations. The red colour marks an increase, the blue colour a decrease of electron density during the corresponding electronic transition.

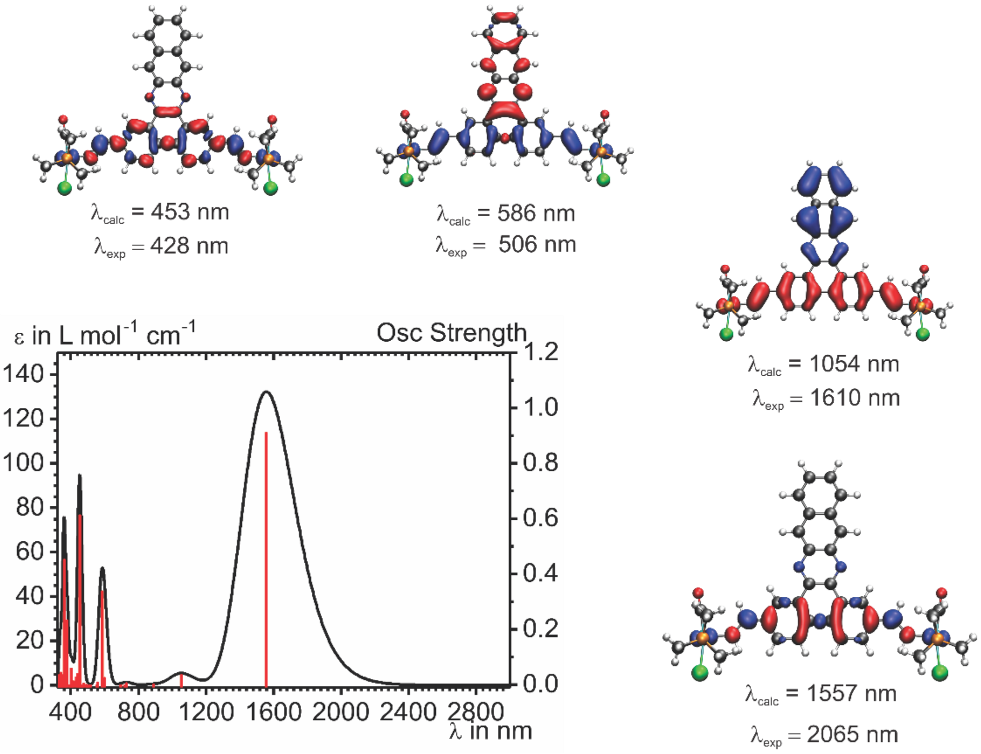


**Figure S70.** Calculated (pbe1pbe) UV-vis-NIR spectrum of **[Ru$_2$-7]$^+$** obtained from the TD-DFT calculations. The red colour marks an increase, the blue colour a decrease of electron density during the corresponding electronic transition.

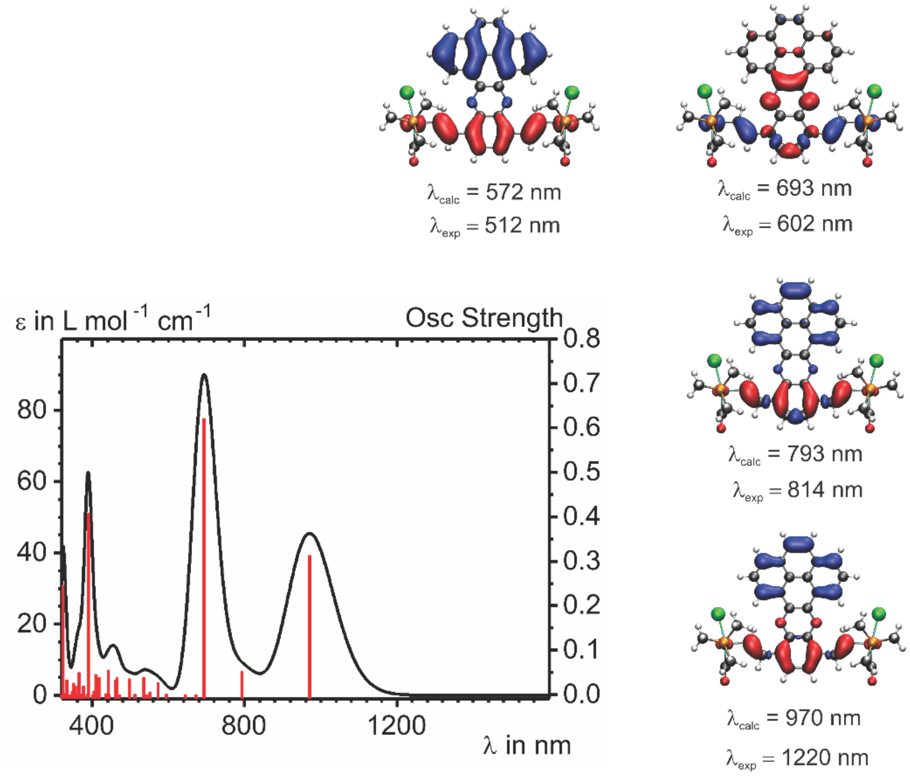


**Figure S71.** Calculated (pbe1pbe) UV-vis-NIR spectrum of ***cis*-[Ru$_2$-7]$^+$** obtained from the TD-DFT calculations. The red colour marks an increase, the blue colour a decrease of electron density during the corresponding electronic transition.

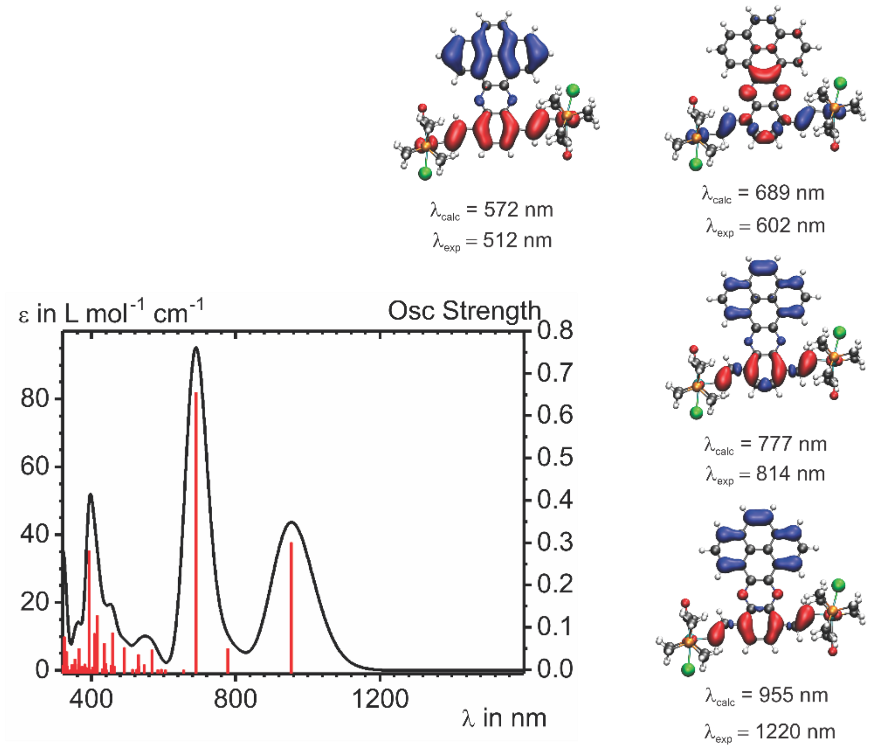


**Figure S72.** Calculated (pbe1pbe) UV-vis-NIR spectrum of ***trans*-[Ru$_2$-7]$^+$** obtained from the TD-DFT calculations. The red colour marks an increase, the blue colour a decrease of electron density during the corresponding electronic transition.

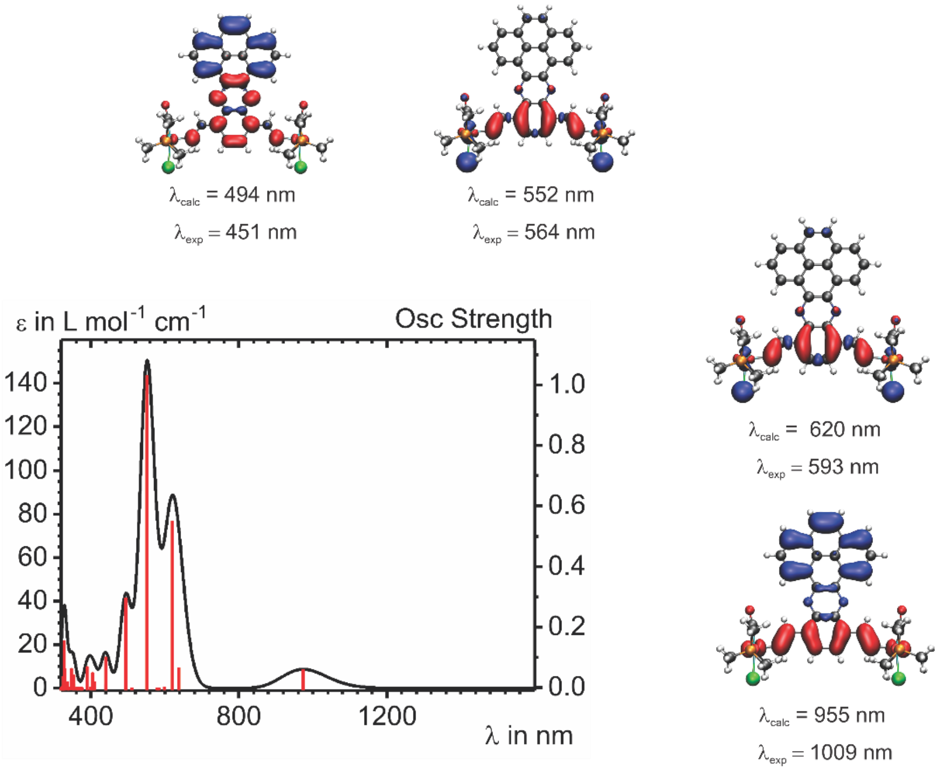


**Figure S73.** Calculated (pbe1pbe) UV-vis-NIR spectrum of **[Ru$_2$-7]$^{2+}$** in its singlet state obtained from the TD-DFT calculations. The red colour marks an increase, the blue colour a decrease of electron density during the corresponding electronic transition.

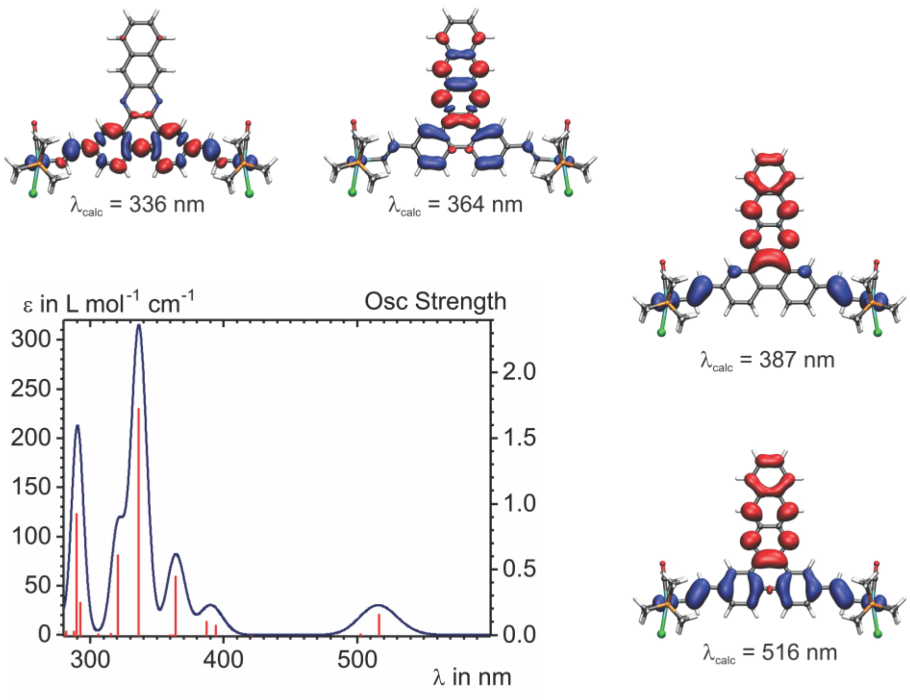


**Figure S74.** Calculated (BLYP35) UV-vis-NIR spectrum of **$Ru_2$-3** obtained from the TD-DFT calculations. The red colour marks an increase, the blue colour a decrease of electron density during the corresponding electronic transition.

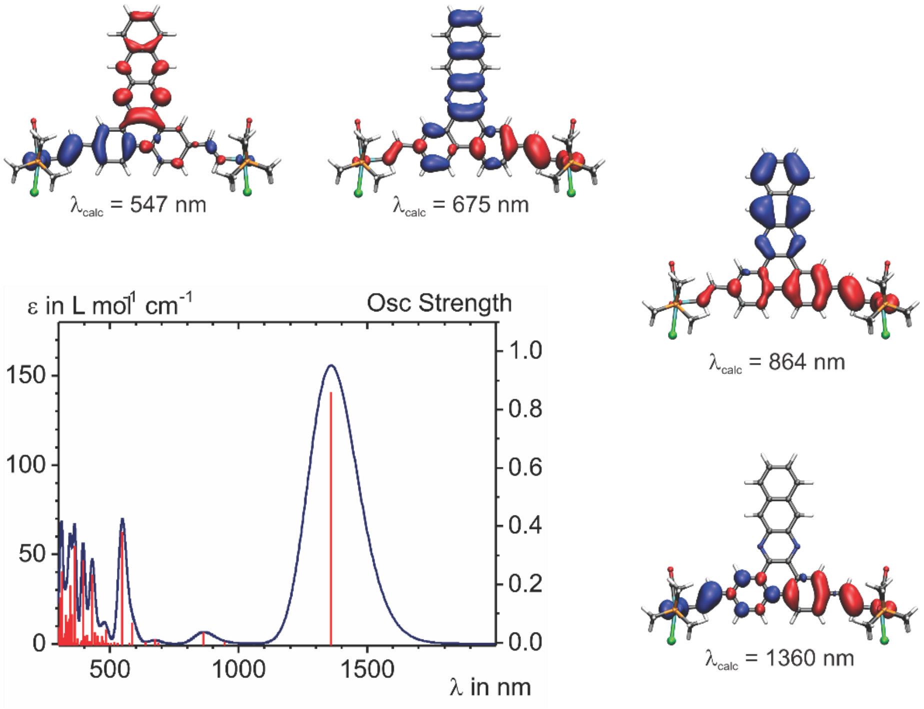


**Figure S75.** Calculated (BLYP35) UV-vis-NIR spectrum of the *Ru trans* conformer of **$[Ru_2\text{-}3]^+$** obtained from TD-DFT calculations. The red colour marks an increase, the blue colour a decrease of electron density during the corresponding electronic transition.

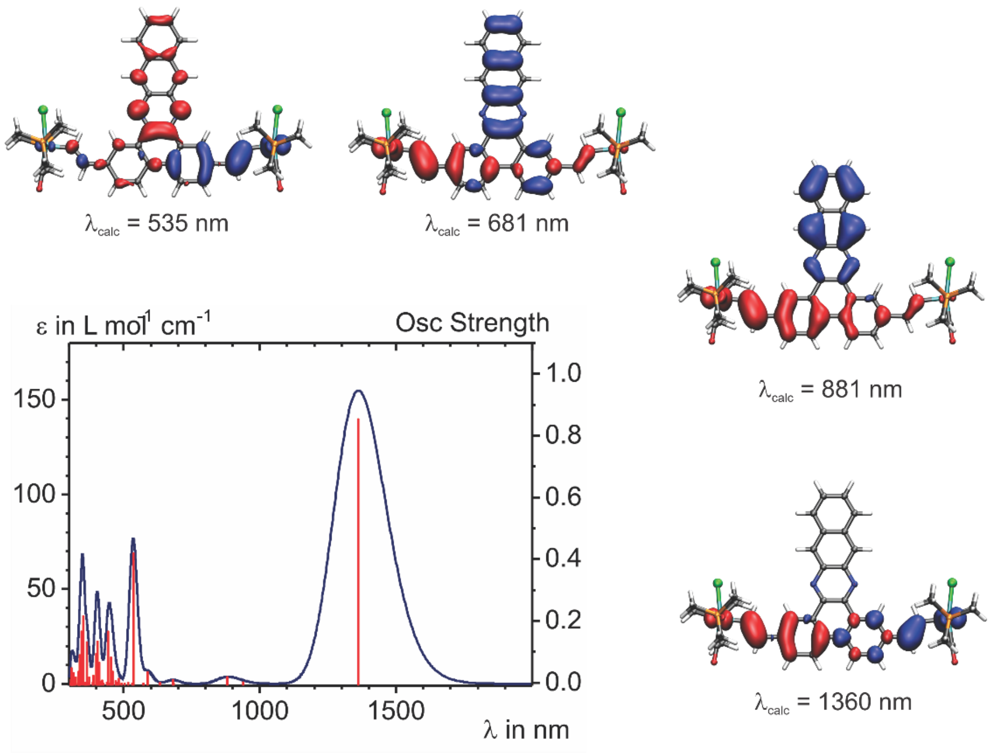


**Figure S76.** Calculated (BLYP35) UV-vis-NIR spectrum of the *Ru cis* conformer of **$[Ru_2\text{-}3]^+$** obtained from TD-DFT calculations. The red colour marks an increase, the blue colour a decrease of electron density during the corresponding electronic transition.

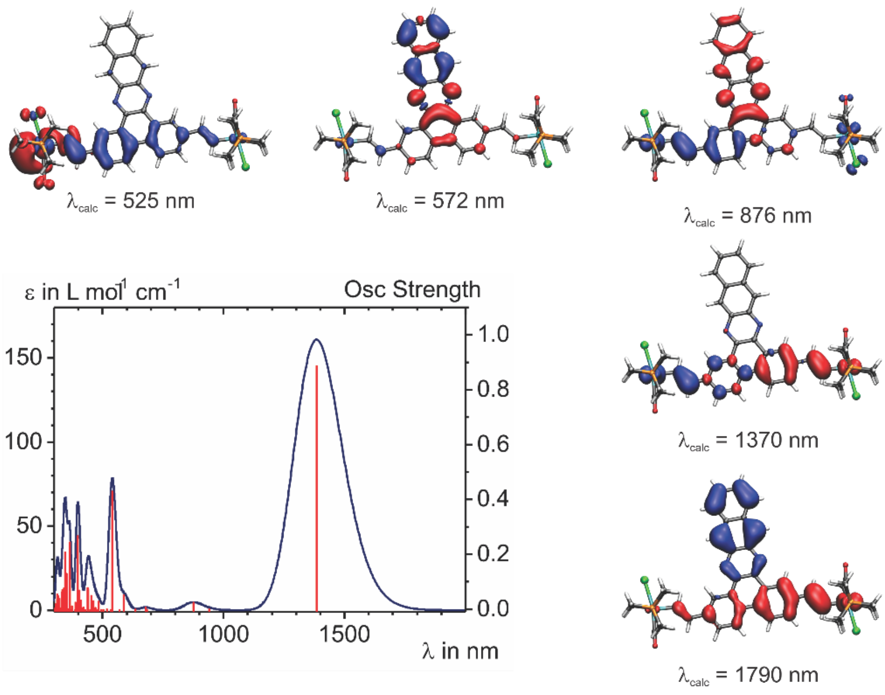


**Figure S77.** Calculated (BLYP35) UV-vis-NIR spectrum of the *Ru cis/trans* conformer of **$[Ru_2\text{-}3]^+$** obtained from TD-DFT calculations. The red colour marks an increase, the blue colour a decrease of electron density during the corresponding electronic transition.

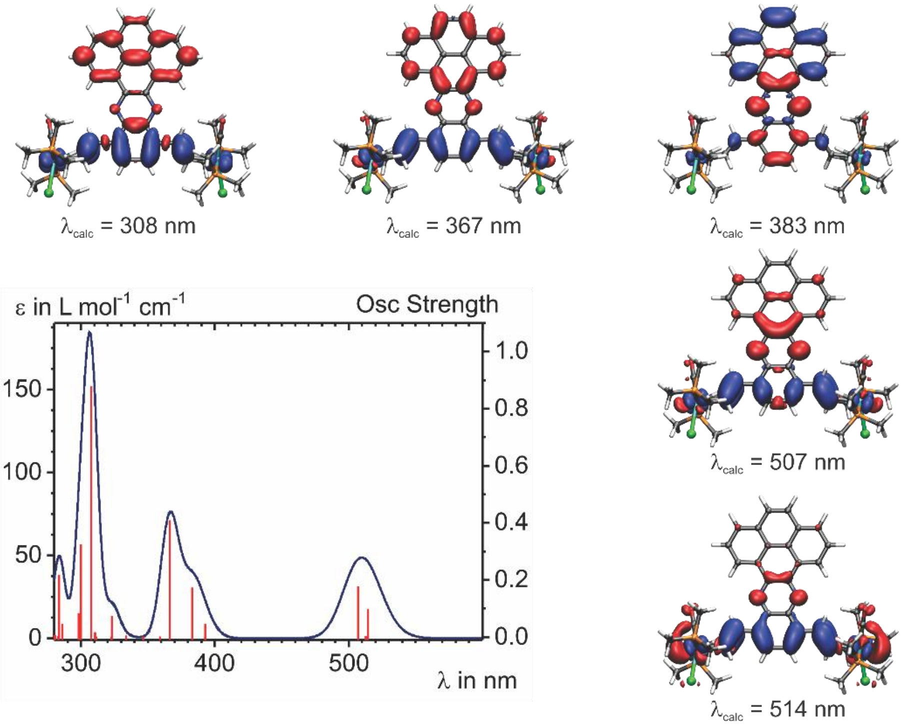


**Figure S78.** Calculated (BLYP35) UV-vis-NIR spectrum of **$Ru_2$-7** obtained from the TD-DFT calculations. The red colour marks an increase, the blue colour a decrease of electron density during the corresponding electronic transition.

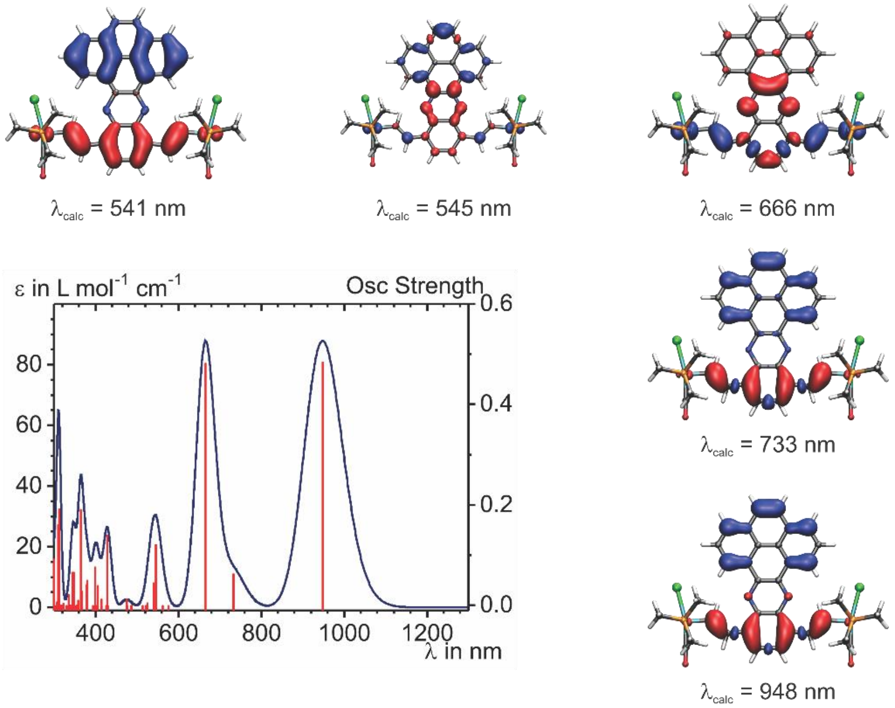


**Figure S79.** Calculated (BLYP35) UV-vis-NIR spectrum of the *Ru cis/trans* conformer of **$[Ru_2\text{-}7]^+$** obtained from the TD-DFT calculations. The red colour marks an increase, the blue colour a decrease of electron density during the corresponding electronic transition.

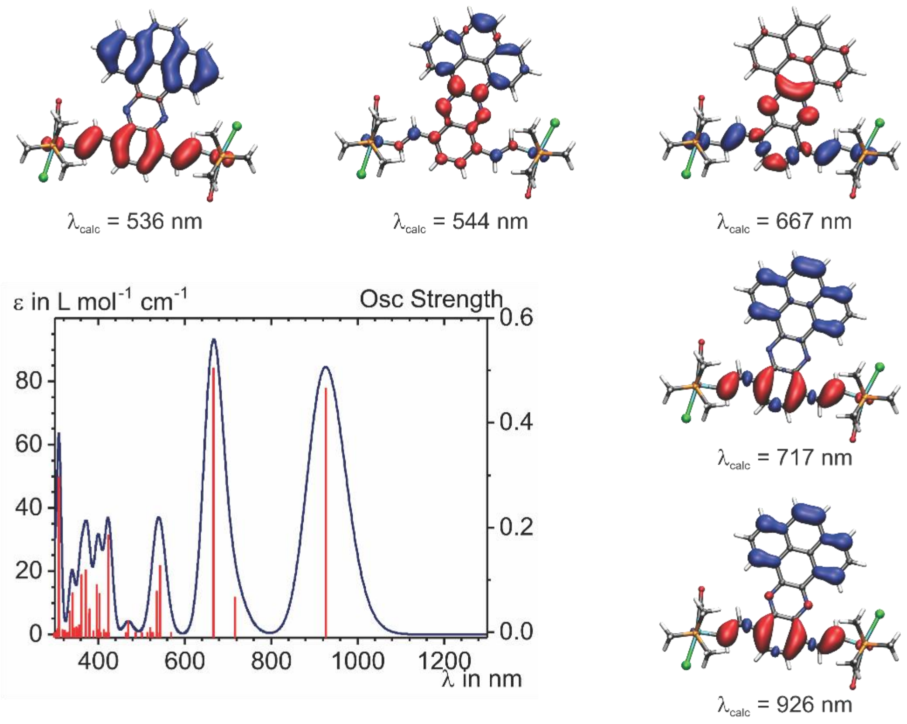


**Figure S80.** Calculated (BLYP35) UV-vis-NIR spectrum of the *Ru cis* conformer of **[Ru$_2$-7]$^+$** obtained from the TD-DFT calculations. The red colour marks an increase, the blue colour a decrease of electron density during the corresponding electronic transition.

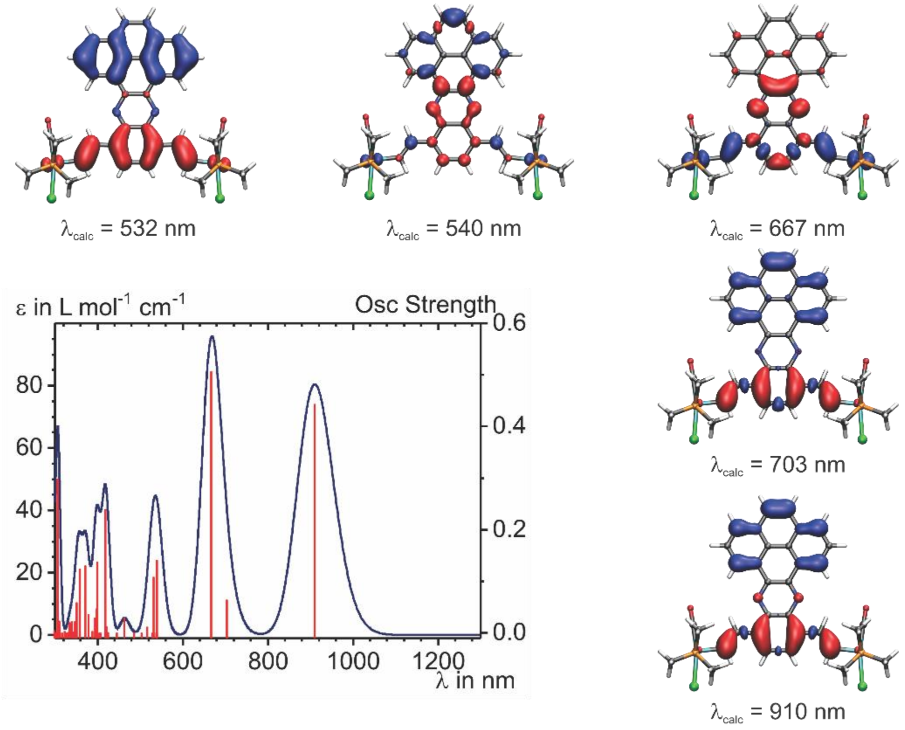


**Figure S81.** Calculated (BLYP35) UV-vis-NIR spectrum of the *Ru trans* conformer of **[Ru$_2$-7]$^+$** obtained from the TD-DFT calculations. The red colour marks an increase, the blue colour a decrease of electron density during the corresponding electronic transition.

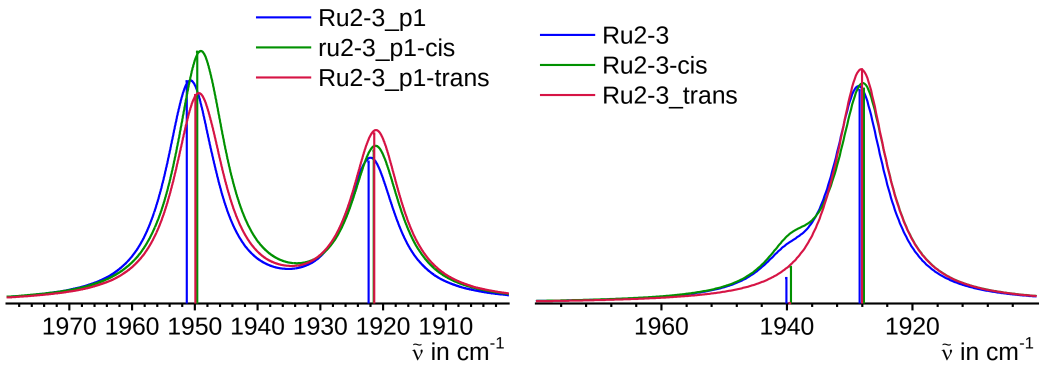


**Figure S82.** Calculated IR spectra in the region of the Ru(CO) stretching vibrations of the three rotamers of **[$Ru_2$-3]$^+$** (left: DFT/BLYP35/6-31G*/PCM($CH_2Cl$-$CH_2Cl$); right: pbe1pbe/6-31G*/PCM($CH_2Cl$-$CH_2Cl$)).

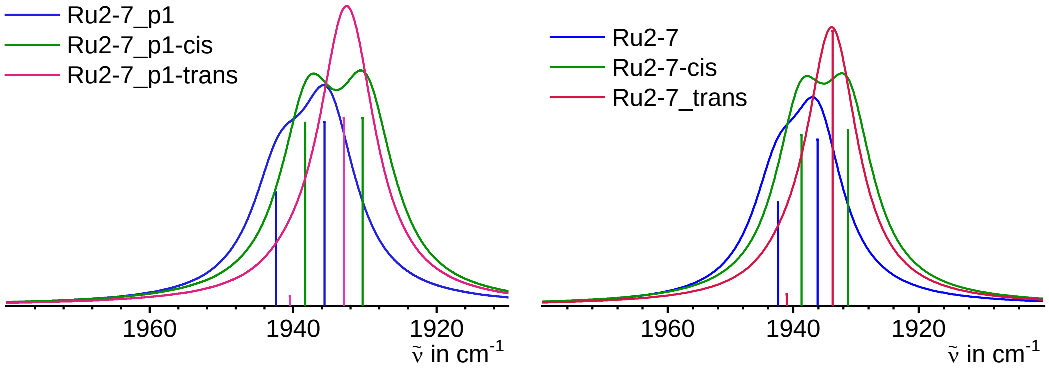


**Figure S83.** Calculated IR spectra in the region of the Ru(CO) stretching vibrations of the three rotamers of **[$Ru_2$-7]$^+$** (left: DFT/BLYP35/6-31G*/PCM($CH_2Cl$-$CH_2Cl$); right: pbe1pbe/6-31G*/PCM($CH_2Cl$-$CH_2Cl$)).

**EPR Spectroscopy**

Complex **$Ru_2$-3**:

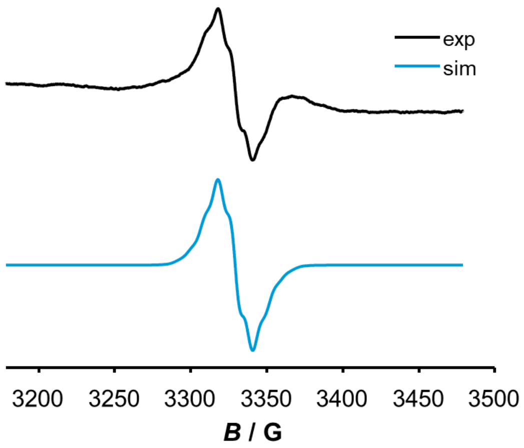


**Figure S84.** EPR Spectra of **[$Ru_2$-3]$^+$** measured at room temperature; experimentally observed (black line, top) and simulated (blue line, bottom).

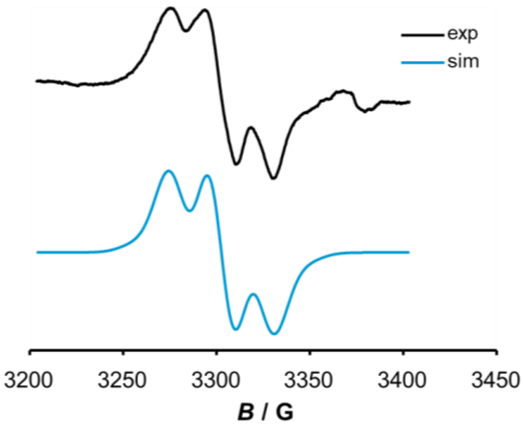


**Figure S85.** EPR Spectra of **[$Ru_2$-3]$^{2+}$** measured at room temperature (left), experimentally observed (black line, top) and simulated (blue line, bottom). EPR spectra of **[$Ru_2$-3]$^{2+}$** measured at different temperatures (right).

Complex **Ru$_2$-4**:

**Figure S86.** EPR Spectra of **[Ru$_2$-4]$^+$** measured at room temperature; experimentally observed (black line, top) and simulated (blue line, bottom).

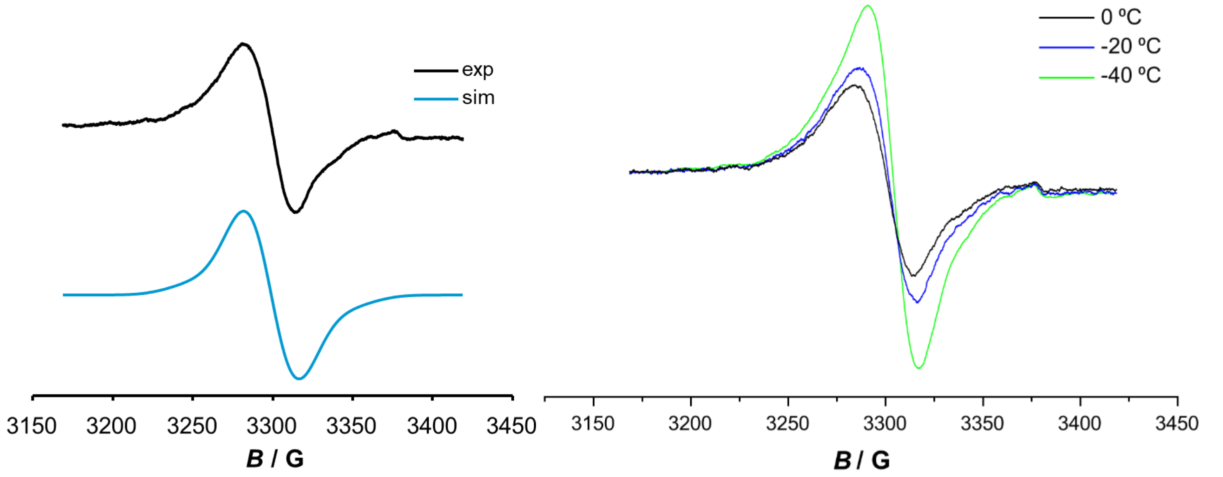


**Figure S87.** EPR Spectra of **[Ru$_2$-4]$^{2+}$** measured at room temperature (left), experimentally observed (black line, top) and simulated (blue line, bottom). EPR spectra of **[Ru$_2$-4]$^{2+}$** measured at different temperatures (right).

Complex **Ru$_2$-7**:

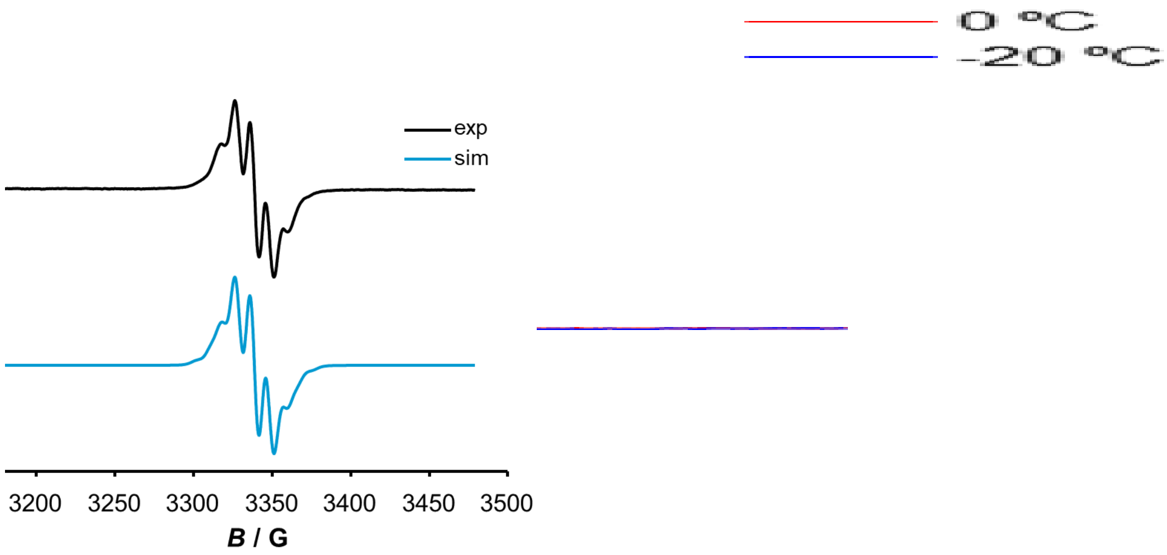


**Figure S88.** EPR Spectra of **[Ru$_2$-7]$^+$** measured at room temperature (left), experimentally observed (black line, top) and simulated (blue line, bottom). EPR spectra of **[Ru$_2$-7]$^+$** measured at = 0 °C and -20 °C (right).

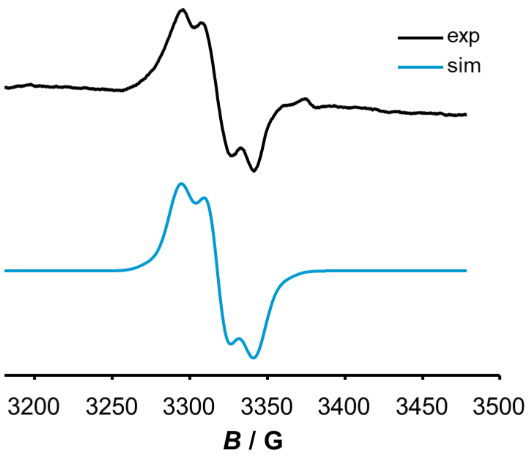


**Figure S89.** EPR Spectra of **[Ru$_2$-7]$^{2+}$** measured at room temperature (left), experimentally observed (black line, top) and simulated (blue line, bottom). EPR spectra of **[Ru$_2$-7]$^{2+}$** measured at difrerent temperatures (right).

Complex **$Ru_2$-8**:

**Figure S90.** EPR Spectra of **$[Ru_2\text{-}8]^+$** measured at room temperature; experimentally observed (black line, top) and simulated (blue line, bottom).

**Figure S91.** EPR Spectra of **$[Ru_2\text{-}8]^{2+}$** measured at room temperature (left), experimentally observed (black line, top) and simulated (blue line, bottom). EPR spectra of **$[Ru_2\text{-}8]^{2+}$** measured at different temperatures (right).

Complex **Ru$_2$-10**:

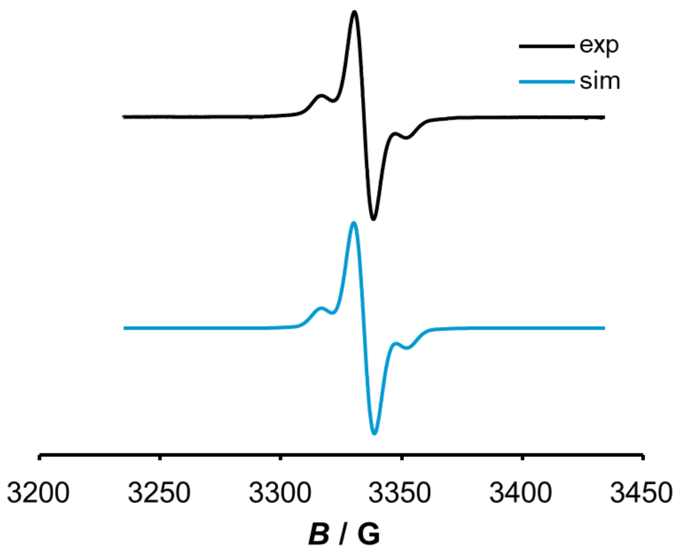


**Figure S92.** EPR Spectra of **[Ru$_2$-10]$^+$** measured at room temperature; experimentally observed (black line, top) and simulated (blue line, bottom).

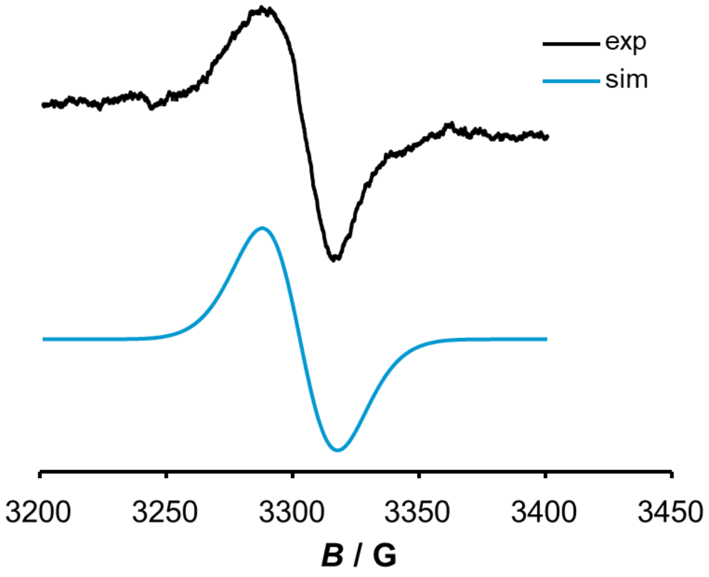


**Figure S93.** EPR Spectra of **[Ru$_2$-10]$^{2+}$** measured at room temperature (left), experimentally observed (black line, top) and simulated (blue line, bottom).

## Spectroscopic characterization of charge-transfer salts

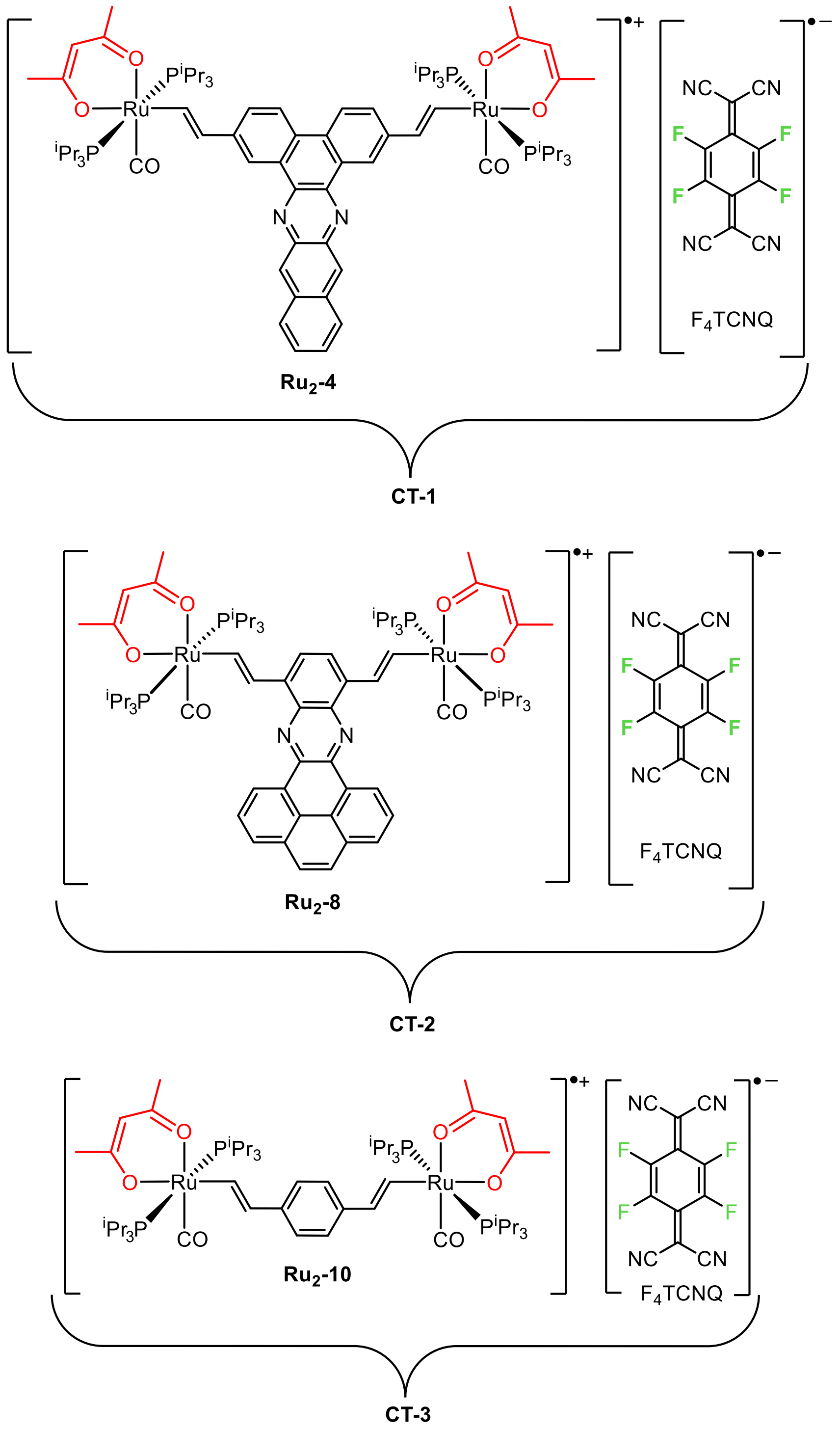


**Figure S94.** The charge-transfer salts **CT-1**, **CT-2** and **CT-3** synthesized in this study.

## IR Characterization of the CT salt CT-1 using $Ru_2$-4 and $F_4$TCNQ in a 1:1 ratio

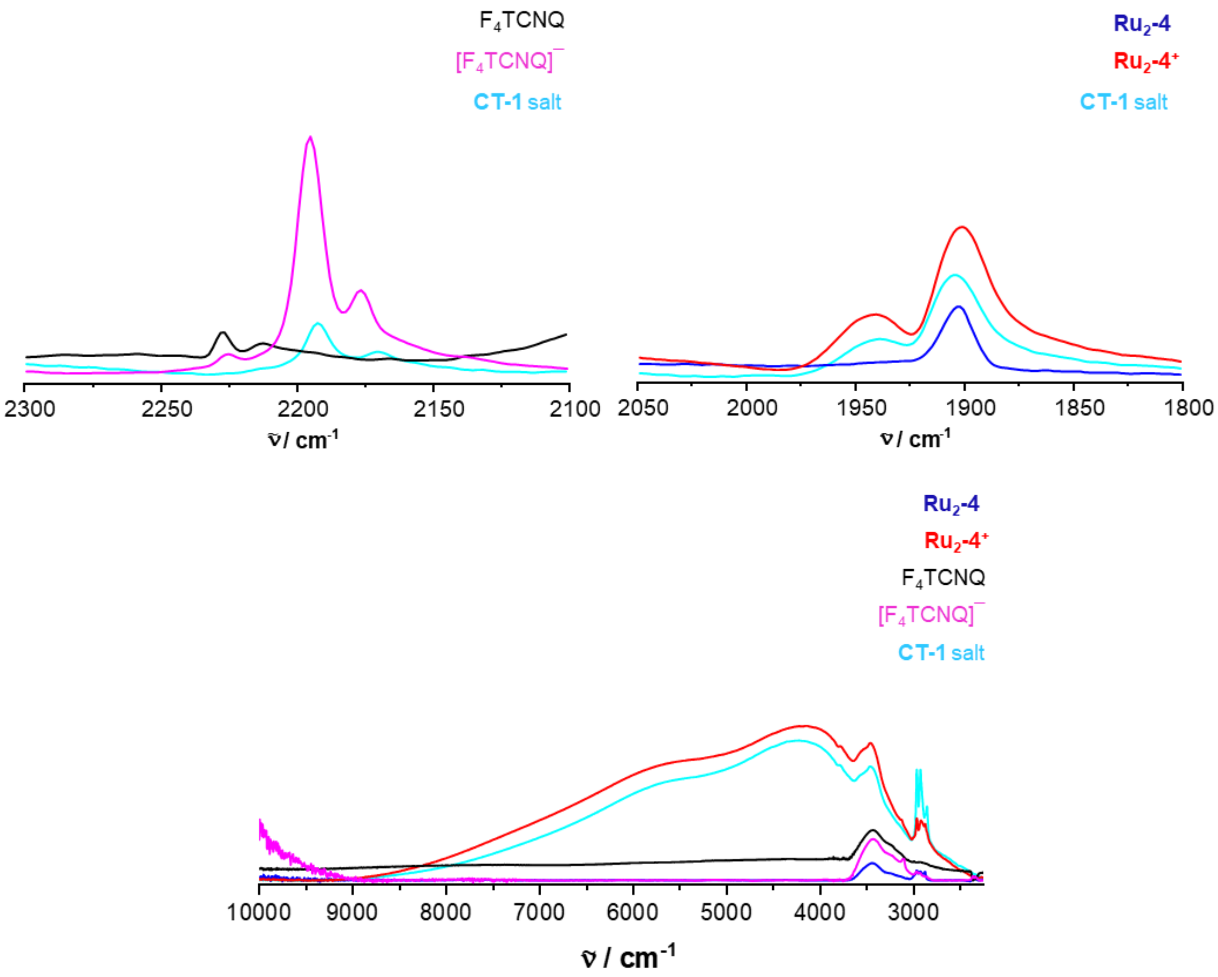


**Figure S95.** Solid-state IR spectra of **CT-1** salt (**1:1** mixture of **$Ru_2$-4** and $F_4$TCNQ) with the spectra of the neutral complex **$Ru_2$-4**, neutral $F_4$TCNQ, the mono-oxidized complex and of $F_4TCNQ^{\bullet -}$ for comparison purposes. Change of CO stretching vibration (top right), CN stretching vibration (top left) and the changes in NIR region are displayed.

## UV/Vis/NIR Characterization of the CT salt CT-2 using $Ru_2$-8 and $F_4$TCNQ in a 1:1 ratio

**Figure S96.** UV/vis/NIR spectrum of **CT-2** salt (**1:1** mixture of **$Ru_2$-8** and $F_4$TCNQ) with the spectra of the neutral complex, neutral $F_4$TCNQ, the mono-oxidized complex and of $F_4TCNQ^{\bullet-}$ for comparison purposes.

## IR Characterization of the CT salt CT-2 using $Ru_2$-8 and $F_4$TCNQ in a 1:1 ratio

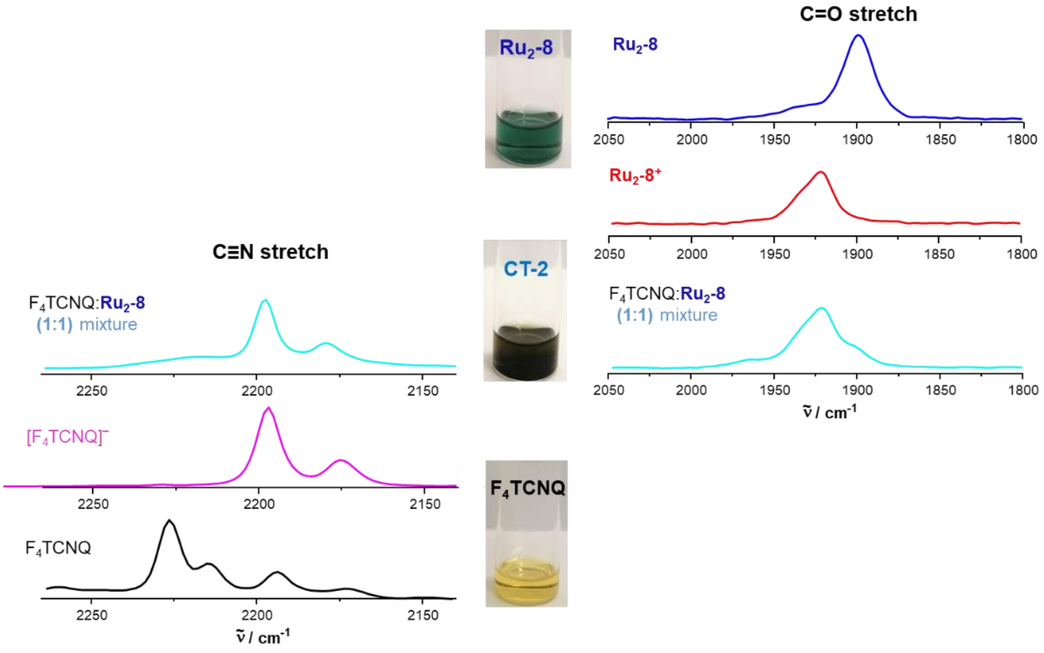


**Figure S97.** Monitoring the formation of salt **CT-2** (**1:1** mixture of **$Ru_2$-8** and $F_4$TCNQ) through IR spectroscopy. The blue shift of the CO stretch of ruthenium complex due to its oxidation and the red-shift of CN stretch of the $F_4$TCNQ due to its reduction are clearly observed. Photographic

representations of solutions of the corresponding neutral and oxidized complexes and of $F_4TCNQ$ also shown.

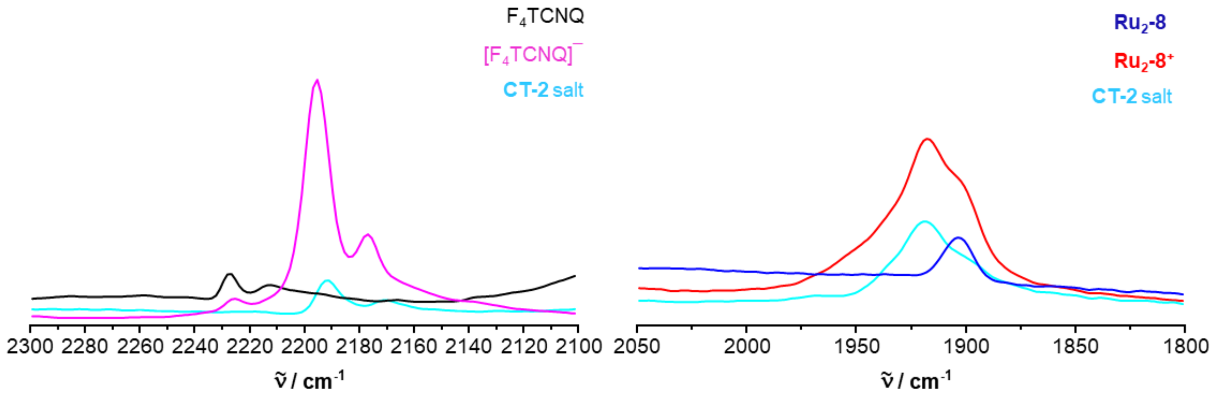


**Figure S98.** Solid-state IR spectra of **CT-2** salt (**1:1** mixture of **$Ru_2$-8** and $F_4TCNQ$) with the spectra of the neutral complex **$Ru_2$-8**, neutral $F_4TCNQ$, the mono-oxidized complex and of $F_4TCNQ^{\bullet-}$ for comparison purposes. Change of CO stretching vibration (right) and CN stretching vibration (left) are displayed.

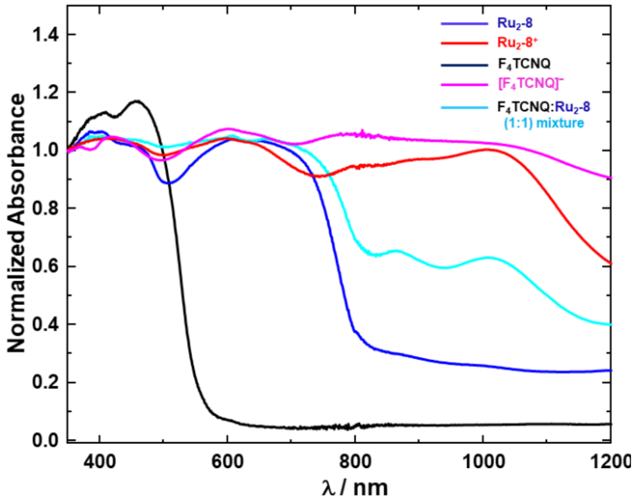


**Figure S99.** Solid-state UV/Vis/NIR spectrum of **CT-2** with its neutral components **$Ru_2$-8** and the organic electron-acceptor $F_4TCNQ$. The spectra of the monooxidized **$Ru_2$-8$^+$** as well as that of the reduced anion $F_4TCNQ^{\bullet-}$ have also been plotted for comparison purposes.

**EPR Characterization of CT salts**

**EPR spectrum of CT-1**

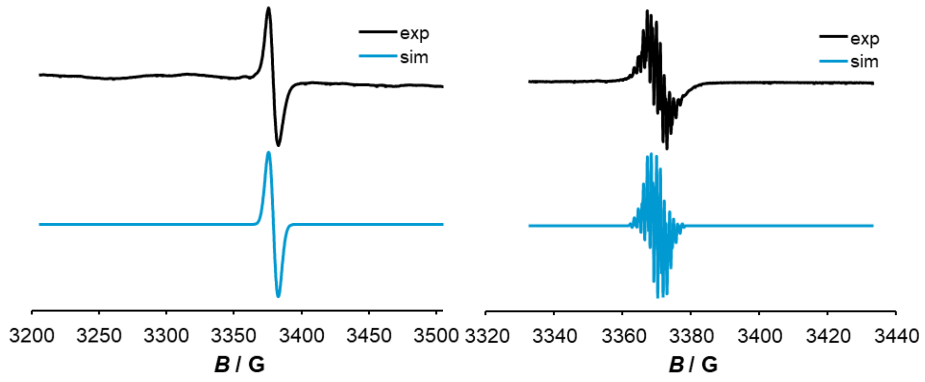


**Figure S100.** EPR spectrum of charge-transfer salt **CT-1** (**$Ru_2$-4·$F_4$TCNQ**) measured in $CH_2Cl_2$ solution. The spectrum shows mainly the signal of the $F_4TCNQ^{•-}$ radical anion. At low modulation, the hyperfine splitting to the fluorine and nitrogen atoms are resolved (right).

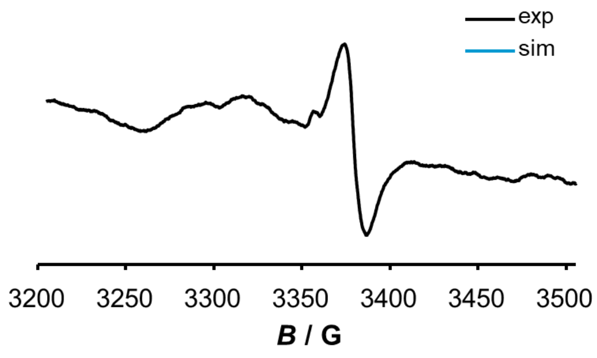


**Figure S101.** EPR spectrum of a powdered sample of charge-transfer salt **CT-1** (**$Ru_2$-4·$F_4$TCNQ**).

## Characterization of CT-3 salt

## IR Characterization of the CT-3 salt using $Ru_2$-10 and $F_4$TCNQ in a 1:1 ratio

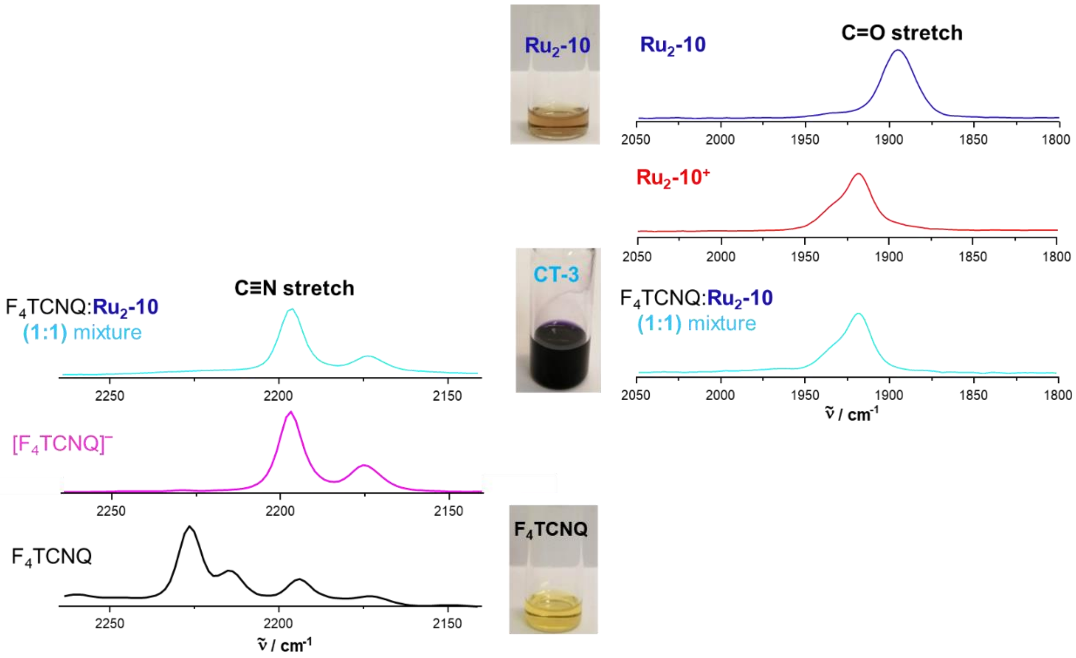


**Figure S102.** Monitoring the formation of charge transfer salt using **1:1** mixture of **$Ru_2$-10** and $F_4$TCNQ through the IR spectroscopy. The blue shift of the CO stretch of ruthenium complex due to its first oxidation is clearly observed.

## UV-vis-NIR and EPR Characterization of CT-3 salt using $Ru_2$-10 and $F_4$TCNQ in 1:1 ratio

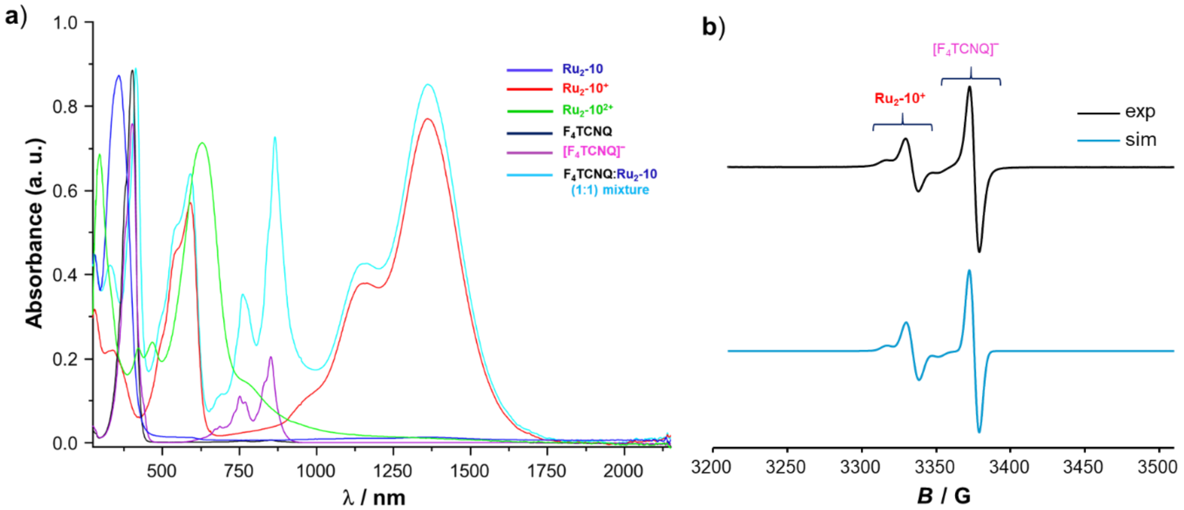


**Figure S103. a**) The UV/vis/NIR spectrum of charge transfer salt **CT-3** (**1:1** mixture of **$Ru_2$-10** and $F_4$TCNQ) is plotted with spectra of the neutral complex, neutral $F_4$TCNQ, the mono- and dioxidized complex and of the $F_4TCNQ^{\bullet-}$ anion for comparison purposes. **b**) EPR spectrum of salt **CT-3**. Two separate signals for the radical cation and radical anion are clearly observed. The experimental spectrum is shown at the top and simulated one is at the bottom.

**Monitoring the decomposition of CT-3 salt**

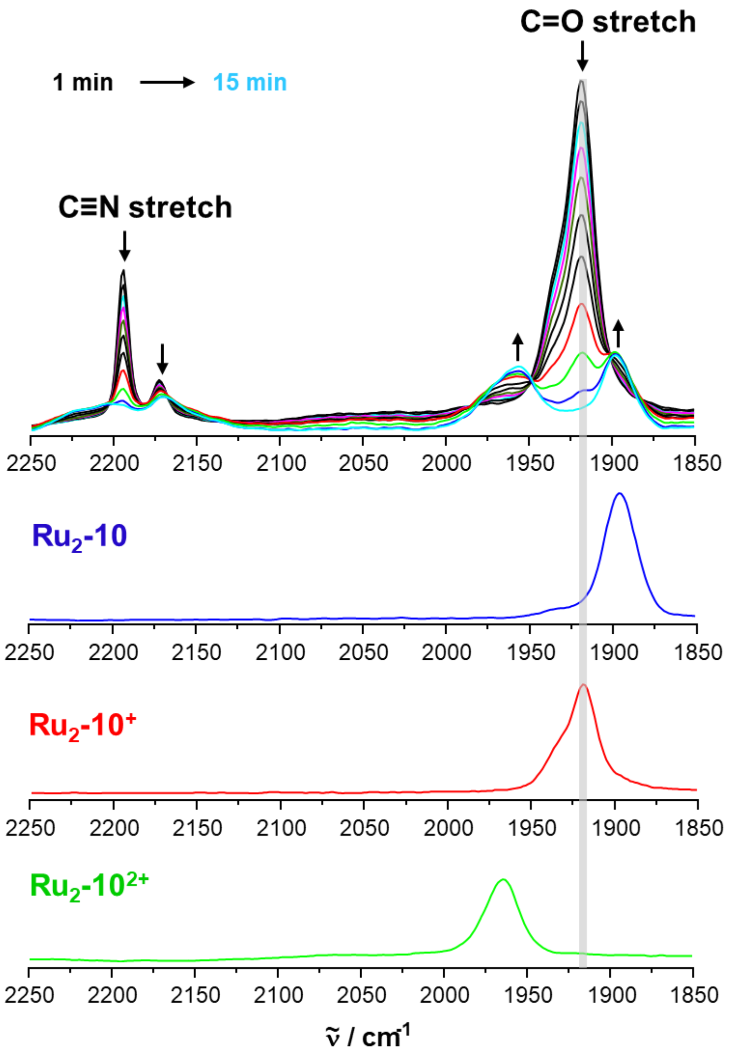


**Figure S104.** Monitoring the stability of **CT-3** salt by IR-spectroscopy. The CT salt decomposed within 15 minutes.

**Conductivity Measurements with charge-transfer salts:**

**Figure S105.** Conductivity measurements on charge-transfer salt **CT-1** through the transfer length method (TLM) using **a**) 70 μm and **b**) 10 μm gaps. A maximum gate voltage ($V_g$) of 20 V was applied.

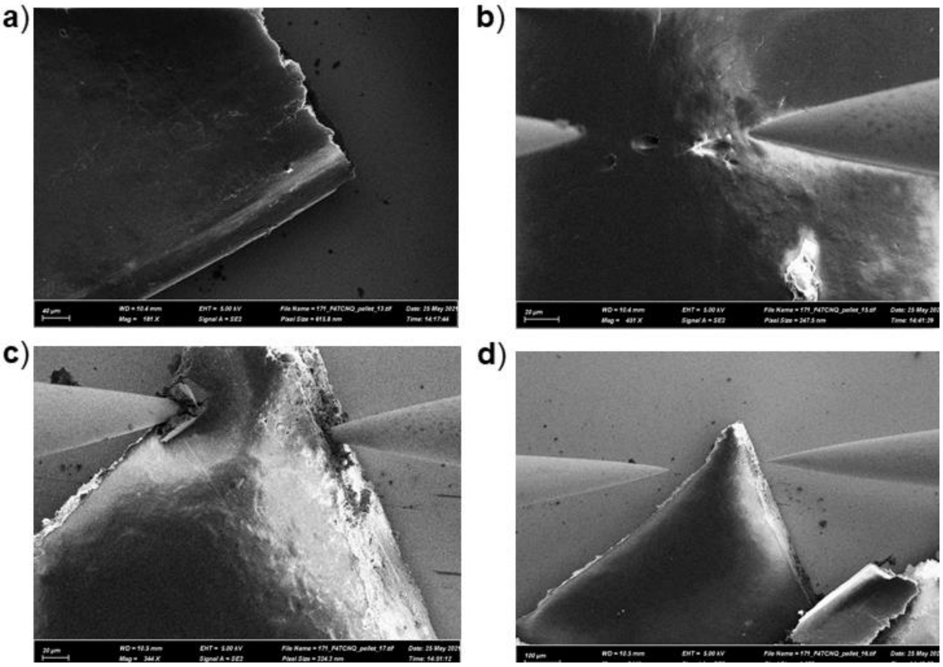


**Figure S106.** Conductivity measurement with the **CT-1** salt. **a**) The pellet was prepared using an IR pellet maker. **b**)-**d**) Nano probes with two platinum cathode and anode electrodes were used to reach the closest distance on the pellet to minimize the resistance.

**Figure S107. a,b**) SEM pictures of freshly synthesized charge-transfer salt **CT-1** after solvent evaporation.

**IR Characterization of the CT salt CT-2' using $Ru_2$-8 and $F_4$TCNQ in a 1:2 ratio**

**Figure S108.** Monitoring the formation of charge transfer salt using **1:2** mixture of **$Ru_2$-8** and $F_4$TCNQ through the IR spectroscopy. The further blue shift of the CO stretch of ruthenium complex due to its second oxidation is clearly observed.

**UV-vis-NIR Characterization of CT salt using $Ru_2$-8 and $F_4TCNQ$ in 1:2 ratio**

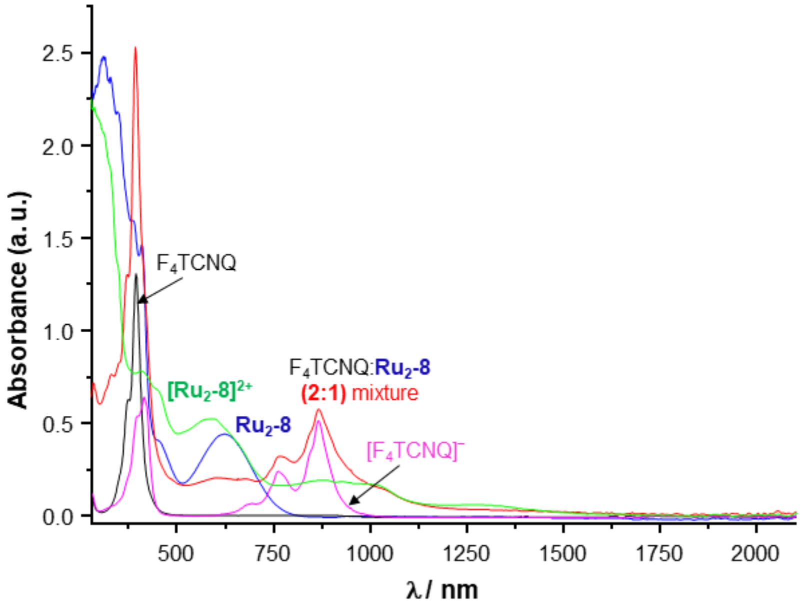


**Figure S109.** The UV/vis/NIR spectrum of charge transfer salt **CT-2'** (**1:2** mixture of **$Ru_2$-8** and $F_4TCNQ$) is plotted with spectra of the neutral complex, neutral $F_4TCNQ$, the dioxidized complex and of the $F_4TCNQ^{\bullet -}$ anion for comparison purposes.

## References


[1] J. Wu, G. Lai, Z. Li, Y. Lu, T. Leng, Y. Shen, C. Wang, *Dyes Pigments* **2016**, *124*, 268-276.

[2] A. L. Appleton, S. Miao, S. M. Brombosz, N. J. Berger, S. Barlow, S. R. Marder, B. M. Lawrence, K. I. Hardcastle, U. H. F. Bunz, *Org. Lett.* **2009**, *11*, 5222-5225.

[3] J. Merz, M. Dietz, Y. Vonhausen, F. Wober, A. Friedrich, D. Sieh, I. Krummenacher, H. Braunschweig, M. Moos, M. Holzapfel, C. Lambert, T. B. Marder, *Chem. Eur. J.* **2020**, *26*, 438-453.

[4] J. Maurer, B. Sarkar, B. Schwederski, W. Kaim, R. F. Winter, S. Záliš, *Organometallics* **2006**, *25*, 3701–3712.

[5] M. Krejčik, M. Daněk, F. Hartl, *J. Electroanal. Chem. Interfac. Electrochem.* **1991**, *317*, 179-187.

[6] M. Rudolph, S. Feldberg, DigiSim3, Version 3.03, Bioanalytical Systems, Inc., **1994**.

[7] S. Stoll, A. Schweiger, *J. Magn. Reson.* **2006**, *178*, 42-55.

[8] M. J. Frisch, G. W. Trucks, H. B. Schlegel, G. E. Scuseria, M. A. Robb, J. R. Cheeseman, G. Scalmani, V. Barone, G. A. Petersson, H. Nakatsuji, X. Li, M. Caricato, A. Marenich, J. Bloino, B. G. Janesko, R. Gomperts, B. Mennucci, H. P. Hratchian, J. V. Ortiz, A. F. Izmaylov, J. L. Sonnenberg, D. Williams-Young, F. Ding, F. Lipparini, F. Egidi, J. Goings, B. Peng, A. Petrone, T. Henderson, D. Ranasinghe, V. G. Zakrzewski, J. Gao, N. Rega, G. Zheng, W. Liang, M. Hada, M. Ehara, K. Toyota, R. Fukuda, J. Hasegawa, M. Ishida, T. Nakajima, Y. Honda, O. Kitao, H. Nakai, T. Vreven, K. Throssell, J. J. A. Montgomery, J. E. Peralta, F. Ogliaro, M. Bearpark, J. J. Heyd, E. Brothers, K. N. Kudin, V. N. Staroverov, T. Keith, R. Kobayashi, J. Normand, K. Raghavachari, A. Rendell, J. C. Burant, S. S. Iyengar, J. Tomasi, M. Cossi, J. M. Millam, M. Klene, C. Adamo, R. Cammi, J. W. Ochterski, R. L. Martin, K. Morokuma, O. Farkas, J. B. Foresman, D. J. Fox, *Gaussian 09, revision D.01; Gaussian, Inc.: Wallingford, CT, 2009*.

[9] a) B. Mennucci, J. Tomasi, *J. Chem. Phys.* **2010**, *132*, 154104; b) M. Cossi, N. Rega, G. Scalmani, V. Barone, *J. Comput. Chem.* **2003**, *24*, 669– 681.

[10] a) M. Dolg, H. Stoll, H. Preuss, *J. Chem. Phys.* **2010**, *132*, 154104; b) W. Küchle, M. Dolg, H. Stoll, H. Preuss, *J. Chem. Phys.* **1994**, *100*, 7535– 7532.

[11] D. Andrae, U. Haeussermann, M. Dolg, H. Stoll, H. Preuss, *Theor. Chim. Acta* **1990**, *77*, 123–141.

[12] P. H. Hariharan, J. A. Pople, *Theor. Chim. Acta* **1973**, *28*, 213– 222.

[13] a) J. P. Perdew, K. Burke, M. Ernzerhof, *Phys. Rev. Lett.* **1996**, *77*, 3865– 3868; b) C. Adamo, V. Barone, *J. Chem. Phys.* **2006**, *124*, 6158-6170.

[14] M. Renz, M. Kess, M. Diedenhofen, A. Klamt, M. Kaupp, *J. Chem. Theory Comput.* **2012**, *8*, 4189-4203.

[15] N. M. O'Boyle, A. L. Tenderholt, K. M. Langner, *J. Comput. Chem.* **2008**, *29*, 839-845.

[16] M. D. Hanwell, D. E. Curtis, D. C. Lonie, T. Vandermeersch, E. Zurek, G. R. Hutchison, *J. Cheminf.* **2012**, *4*, 17.

[17] O. Tange, *USENIX Magazine* **2011**, *36*, 42-47.

[18] W. Humphrey, A. Dalke, K. Schulten, *J. Mol. Graphics* **1996**, *14*, 33– 38.

[19] O. V. Dolomanov, L. J. Bourhis, R. J. Gildea, J. A. K. Howard, H. Puschmann, *J. Appl. Cryst.* **2009**, *42*, 339-341.

[20] G. M. Sheldrick, *Acta Cryst. C* **2015**, *71*, 3-8.

[21] L. J. Farrugia, *J. Appl. Cryst.* **2012**, *45*, 849-854.

[22] S. A. Odom, M. M. Caruso, A. D. Finke, A. M. Prokup, J. A. Ritchey, J. H. Leonard, S. R. White, N. R. Sottos, J. S. Moore, *Adv. Funct. Mat.* **2010**, *20*, 1721-1727.

[23] Y. S. Guan, Y. Hu, H. Zhang, G. Wu, H. Yan, S. Ren, *Chem. Commun.* **2019**, *55*, 7179-7182.

[24] Z. Wang, X. Song, L. Ma, Y. Feng, C. Gu, X. Zhang, P. Lu, Y. Ma, *New J. Chem.* **2013**, *37*.

[25] J. J. Bryant, Y. Zhang, B. D. Lindner, E. A. Davey, A. L. Appleton, X. Qian, U. H. Bunz, *J. Org. Chem.* **2012**, *77*, 7479-7486.

[26] J. Lu, R. Ojha, A. M. Bond, L. L. Martin, *Cryst. Growth Des.* **2019**, *19*, 2712-2722.

**Atomic coordinates of $[Ru_2\text{-}3]^-$ from its DFT structure**

Ru 7.90389 -1.39930 0.01388
P 7.81712 -1.53959 2.37067
O 8.22785 1.55577 0.08646
C 5.92235 -1.26487 -0.02958
H 5.53210 -2.29197 -0.05096
P 7.92514 -1.43466 -2.34787
C 5.04906 -0.23852 -0.03460
H 5.43300 0.78287 -0.01275
C 8.06991 0.39947 0.05725
C 6.64066 -2.79328 2.99652
H 5.62897 -2.52643 2.67634
H 6.89731 -3.76676 2.56971
H 6.67262 -2.84736 4.08991
C 9.40100 -2.00676 3.16579
H 9.73329 -2.96536 2.75607
H 10.16195 -1.25135 2.94610
H 9.28607 -2.09498 4.25161
C 7.31546 -0.01996 3.25795
H 6.34337 0.30910 2.87752
H 7.24019 -0.19955 4.33544
H 8.04466 0.77547 3.07655
C 9.54403 -1.86643 -3.09072
H 9.85998 -2.84147 -2.70791
H 9.47832 -1.90789 -4.18339
H 10.29254 -1.12083 -2.80476
C 6.77854 -2.65606 -3.08351
H 6.85738 -2.65705 -4.17585
H 7.01725 -3.64965 -2.69454
H 5.75385 -2.40385 -2.79428
C 7.46249 0.12470 -3.18613
H 7.43395 -0.00541 -4.27293
H 6.47477 0.43776 -2.83339
H 8.18312 0.90962 -2.93735
C 3.58363 -0.32699 -0.05883
C 2.87202 -1.55024 -0.08958
C 1.49381 -1.56734 -0.09831
H 3.40993 -2.49473 -0.10401
C 1.42760 0.85085 -0.05394
C 0.72585 -0.38281 -0.07753
H 0.99560 -2.53206 -0.11887
Cl 8.35071 -3.80755 -0.02630
C 2.83432 0.84511 -0.04538
H 3.33491 1.80939 -0.02374
C -0.72580 -0.38290 -0.07403
C -1.49377 -1.56737 -0.09800
C -1.42753 0.85054 -0.04055
C -2.87195 -1.55041 -0.08459
H -0.99560 -2.53190 -0.12670

C -2.83420 0.84464 -0.02647
H -3.40986 -2.49483 -0.10309
H -3.33475 1.80878 0.00133
C -0.70919 2.11433 -0.02088
C 0.70918 2.11462 -0.03427
C -0.72627 4.41970 0.02049
C 0.72609 4.42016 -0.00272
C -1.40162 5.64121 0.05382
C 1.40112 5.64232 0.00066
H -2.48975 5.62384 0.07454
H 2.48928 5.62603 -0.01898
N 1.42273 3.25543 -0.02659
N -1.42279 3.25475 0.00884
C -3.58353 -0.32738 -0.04541
C -5.04896 -0.23894 -0.02101
C -5.92241 -1.26514 -0.02677
H -5.43279 0.78229 0.00861
Ru -7.90408 -1.39958 0.01034
H -5.53226 -2.29208 -0.05635
P -7.91834 -1.42347 -2.35176
P -7.82343 -1.55102 2.36648
C -8.07022 0.39897 0.06177
Cl -8.35058 -3.80767 -0.04265
C -6.77114 -2.64288 -3.08977
C -9.53550 -1.84941 -3.10177
C -7.45116 0.13926 -3.18117
C -9.40878 -2.02333 3.15558
C -6.64722 -2.80656 2.98901
C -7.32510 -0.03511 3.26193
O -8.22821 1.55514 0.09590
H -5.74689 -2.39310 -2.79683
H -7.01196 -3.63787 -2.70572
H -6.84711 -2.63899 -4.18230
H -9.85410 -2.82590 -2.72490
H -10.28389 -1.10419 -2.81450
H -9.46638 -1.88551 -4.19442
H -6.46428 0.44960 -2.82371
H -7.41915 0.01413 -4.26847
H -8.17178 0.92381 -2.93118
H -9.73937 -2.98007 2.74020
H -9.29633 -2.11697 4.24120
H -10.16976 -1.26736 2.93793
H -6.68112 -2.86554 4.08209
H -6.90226 -3.77835 2.55741
H -5.63521 -2.53743 2.67177
H -7.25237 -0.21949 4.33877
H -6.35230 0.29628 2.88535
H -8.05440 0.76063 3.08228
C 0.71899 6.87687 0.02972
C -0.71988 6.87628 0.05937
C -1.39748 8.12199 0.09161

C -0.70648 9.31568 0.09364
C 0.70491 9.31630 0.06292
C 1.39624 8.12320 0.03175
H -2.48597 8.11882 0.11439
H -1.24785 10.25842 0.11816
H 1.24602 10.25952 0.06381
H 2.48471 8.12097 0.00830

**Atomic coordinates of neutral $Ru_2$-3 from its DFT structure**

Ru 7.89489 -1.40636 0.01348
P 7.79603 -1.59773 2.36843
O 8.22884 1.54666 0.15148
C 5.92186 -1.26026 -0.04703
H 5.52116 -2.28224 -0.09503
P 7.96626 -1.39646 -2.34960
C 5.05630 -0.22554 -0.03488
H 5.44590 0.79233 0.01093
C 8.07009 0.39203 0.09622
C 6.60756 -2.85328 2.96616
H 5.59769 -2.56860 2.65546
H 6.85302 -3.81900 2.51603
H 6.64170 -2.93338 4.05787
C 9.37690 -2.09466 3.14948
H 9.70033 -3.04730 2.71951
H 10.14362 -1.34110 2.94424
H 9.26245 -2.20438 4.23331
C 7.30747 -0.09248 3.28687
H 6.33831 0.25353 2.91395
H 7.23043 -0.29480 4.36015
H 8.04421 0.69977 3.12313
C 9.60145 -1.82971 -3.05290
H 9.89635 -2.81585 -2.68207
H 9.56648 -1.84726 -4.14757
H 10.34844 -1.09806 -2.72960
C 6.82871 -2.59254 -3.13799
H 6.94132 -2.57683 -4.22719
H 7.04526 -3.59463 -2.75816
H 5.79800 -2.33432 -2.87667
C 7.54203 0.18338 -3.16976
H 7.53901 0.07191 -4.25898
H 6.54969 0.50338 -2.83663
H 8.26627 0.95455 -2.89034
C 3.59339 -0.30655 -0.06803
C 2.86954 -1.51784 -0.11507
C 1.48944 -1.53135 -0.13002
H 3.39917 -2.46634 -0.13682
C 1.44677 0.87308 -0.05993
C 0.73093 -0.34466 -0.09941

H 0.99067 -2.49417 -0.16341
Cl 8.29683 -3.81746 -0.07249
C 2.84634 0.87151 -0.04495
H 3.35290 1.83193 -0.01093
C -0.73093 -0.34466 -0.09942
C -1.48944 -1.53135 -0.13009
C -1.44677 0.87308 -0.05992
C -2.86954 -1.51783 -0.11516
H -0.99067 -2.49417 -0.16355
C -2.84634 0.87151 -0.04493
H -3.39918 -2.46634 -0.13696
H -3.35290 1.83193 -0.01089
C -0.72415 2.14402 -0.03184
C 0.72415 2.14402 -0.03186
C -0.71849 4.43545 0.01897
C 0.71848 4.43545 0.01897
C -1.40945 5.65008 0.04515
C 1.40945 5.65008 0.04511
H -2.49614 5.62873 0.04459
H 2.49614 5.62874 0.04453
N 1.40883 3.26673 -0.00659
N -1.40883 3.26673 -0.00656
C -3.59339 -0.30655 -0.06806
C -5.05630 -0.22553 -0.03489
C -5.92186 -1.26025 -0.04704
H -5.44590 0.79233 0.01095
Ru -7.89489 -1.40637 0.01351
H -5.52116 -2.28224 -0.09508
P -7.96633 -1.39621 -2.34957
P -7.79596 -1.59799 2.36843
C -8.07009 0.39201 0.09645
Cl -8.29685 -3.81745 -0.07271
C -6.82880 -2.59220 -3.13813
C -9.60154 -1.82937 -3.05286
C -7.54213 0.18373 -3.16957
C -9.37681 -2.09498 3.14948
C -6.60749 -2.85362 2.96599
C -7.30734 -0.09284 3.28702
O -8.22882 1.54665 0.15181
H -5.79809 -2.33401 -2.87681
H -7.04535 -3.59433 -2.75841
H -6.94145 -2.57636 -4.22733
H -9.89644 -2.81556 -2.68213
H -10.34853 -1.09776 -2.72946
H -9.56661 -1.84681 -4.14753
H -6.54978 0.50369 -2.83644
H -7.53915 0.07238 -4.25880
H -8.26636 0.95486 -2.89004
H -9.70027 -3.04757 2.71942
H -9.26233 -2.20482 4.23329
H -10.14352 -1.34139 2.94435

H -6.64160 -2.93385 4.05769
H -6.85297 -3.81929 2.51576
H -5.59762 -2.56892 2.65529
H -7.23027 -0.29529 4.36028
H -6.33819 0.25319 2.91411
H -8.04408 0.69943 3.12340
C 0.72218 6.86533 0.07125
C -0.72217 6.86534 0.07128
C -1.40663 8.11804 0.09816
C -0.71219 9.29506 0.12339
C 0.71218 9.29506 0.12337
C 1.40664 8.11805 0.09811
H -2.49377 8.11491 0.09814
H -1.24475 10.24175 0.14363
H 1.24474 10.24175 0.14357
H 2.49378 8.11492 0.09807

**Atomic coordinates of $[Ru_2\text{-}3]^+$ from its DFT structure**

Ru 7.80459 -1.40929 -0.00238
P 7.90880 -1.55572 2.37155
O 8.18128 1.54936 0.03416
C 5.88096 -1.26182 0.00868
H 5.45960 -2.27488 0.00358
P 7.88190 -1.49030 -2.38049
C 5.01167 -0.20019 0.01717
H 5.41523 0.81166 0.01960
C 8.00840 0.40047 0.02029
C 6.71692 -2.73308 3.09909
H 5.69805 -2.38563 2.90265
H 6.85226 -3.71176 2.63130
H 6.86941 -2.81475 4.18030
C 9.53839 -2.13974 2.96134
H 9.74859 -3.11607 2.51537
H 10.31865 -1.43705 2.65349
H 9.54410 -2.22773 4.05305
C 7.61672 -0.00883 3.30063
H 6.63251 0.39178 3.03881
H 7.65394 -0.19304 4.37913
H 8.37583 0.73411 3.03869
C 9.50431 -2.05525 -3.00726
H 9.72154 -3.04393 -2.59307
H 9.49580 -2.11053 -4.10112
H 10.28749 -1.36048 -2.68898
C 6.68071 -2.64652 -3.12626
H 6.81860 -2.69603 -4.21131
H 6.82187 -3.63858 -2.68951
H 5.66469 -2.30512 -2.90596
C 7.57911 0.08148 -3.26312
H 7.60713 -0.07340 -4.34652

H 6.59684 0.47352 -2.98186
H 8.33990 0.81776 -2.98766
C 3.57775 -0.28458 0.01819
C 2.84964 -1.50612 0.02588
C 1.47951 -1.51370 0.02081
H 3.37703 -2.45449 0.03752
C 1.44192 0.91307 0.00647
C 0.71903 -0.31483 0.00647
H 0.97616 -2.47332 0.03129
Cl 8.06490 -3.82557 -0.03653
C 2.82854 0.90771 0.01183
H 3.34240 1.86434 0.00996
C -0.71899 -0.31482 -0.00624
C -1.47948 -1.51368 -0.02060
C -1.44187 0.91308 -0.00615
C -2.84961 -1.50609 -0.02561
H -0.97614 -2.47331 -0.03106
C -2.82849 0.90773 -0.01144
H -3.37701 -2.45446 -0.03715
H -3.34234 1.86437 -0.00944
C -0.72103 2.18745 -0.00140
C 0.72108 2.18744 0.00180
C -0.71928 4.47545 -0.00065
C 0.71935 4.47544 0.00124
C -1.41073 5.68961 -0.00134
C 1.41081 5.68959 0.00205
H -2.49714 5.66832 -0.00274
H 2.49722 5.66830 0.00345
N 1.40908 3.30669 0.00266
N -1.40902 3.30670 -0.00217
C -3.57771 -0.28455 -0.01786
C -5.01164 -0.20014 -0.01675
C -5.88093 -1.26176 -0.00880
H -5.41517 0.81172 -0.01867
Ru -7.80457 -1.40924 0.00219
H -5.45961 -2.27483 -0.00426
P -7.90880 -1.55424 -2.37182
P -7.88196 -1.49174 2.38025
C -8.00837 0.40053 -0.01951
Cl -8.06499 -3.82553 0.03491
C -6.71716 -2.73124 -3.10032
C -9.53852 -2.13764 -2.96186
C -7.61661 -0.00681 -3.29996
C -9.50443 -2.05715 3.00647
C -6.68086 -2.64854 3.12526
C -7.57915 0.07940 3.26400
O -8.18116 1.54944 -0.03280
H -5.69821 -2.38417 -2.90361
H -6.85266 -3.71032 -2.63340
H -6.86969 -2.81190 -4.18160
H -9.74912 -3.11394 -2.51601

H -10.31857 -1.43470 -2.65406
H -9.54411 -2.22552 -4.05358
H -6.63224 0.39341 -3.03815
H -7.65419 -0.19032 -4.37857
H -8.37548 0.73614 -3.03732
H -9.72139 -3.04572 2.59187
H -9.49620 -2.11284 4.10031
H -10.28766 -1.36242 2.68823
H -6.81881 -2.69885 4.21027
H -6.82208 -3.64027 2.68777
H -5.66481 -2.30704 2.90526
H -7.60696 -0.07628 4.34729
H -6.59695 0.47171 2.98287
H -8.34004 0.81585 2.98922
C 0.72247 6.90441 0.00123
C -0.72238 6.90441 -0.00042
C -1.40740 8.15668 -0.00114
C -0.71207 9.33342 -0.00029
C 0.71218 9.33341 0.00133
C 1.40750 8.15667 0.00207
H -2.49432 8.15394 -0.00238
H -1.24406 10.28045 -0.00086
H 1.24418 10.28044 0.00198
H 2.49442 8.15393 0.00332

**Atomic coordinates of *cis*-[$Ru_2$-3]$^+$ from its DFT structure**

Ru -7.77379 -0.70637 0.00088
P -7.85522 -0.67092 2.37993
O -8.23453 -3.65159 -0.09611
C -5.85565 -0.90707 -0.01057
H -5.40588 0.09323 0.02322
P -7.86488 -0.50977 -2.36964
C -5.01414 -1.99029 -0.04899
H -5.44445 -2.99077 -0.07882
C -8.02835 -2.50856 -0.05880
C -6.63843 0.45119 3.15181
H -5.62703 0.09835 2.92843
H -6.76183 1.45352 2.73357
H -6.77979 0.48231 4.23712
C -9.47043 -0.09372 3.01408
H -9.67013 0.90748 2.62169
H -10.26385 -0.76821 2.67806
H -9.46447 -0.06318 4.10890
C -7.57950 -2.26500 3.23068
H -6.60186 -2.66531 2.94494
H -7.61001 -2.13239 4.31695
H -8.34989 -2.98387 2.93667
C -9.48092 0.11371 -2.95655
H -9.67708 1.08456 -2.49256

H -9.47828 0.22344 -4.04629
H -10.27495 -0.58150 -2.66735
C -6.64875 0.66101 -3.06675
H -6.79530 0.77013 -4.14636
H -6.76802 1.63112 -2.57722
H -5.63713 0.29111 -2.87397
C -7.59513 -2.04416 -3.32600
H -7.62337 -1.84048 -4.40125
H -6.61966 -2.46695 -3.06640
H -8.36879 -2.77789 -3.08122
C -3.57816 -1.94145 -0.05049
C -2.84963 -3.16060 -0.07883
C -1.47842 -3.17750 -0.07534
H -3.39735 -4.09892 -0.10318
C -1.44397 -0.75151 -0.02598
C -0.71938 -1.98041 -0.04392
H -0.97788 -4.13832 -0.10066
Cl -7.98072 1.71343 0.08281
C -2.82914 -0.74803 -0.02765
H -3.32199 0.21794 -0.01232
C 0.71921 -1.98054 -0.03123
C 1.47824 -3.17802 -0.02953
C 1.44377 -0.75156 -0.01802
C 2.84944 -3.16120 -0.02339
H 0.97770 -4.13918 -0.02974
C 2.82894 -0.74813 -0.01242
H 3.39716 -4.09983 -0.02243
H 3.32179 0.21794 -0.00450
C 0.72084 0.52317 -0.00908
C -0.72105 0.52315 -0.00868
C 0.71909 2.81189 0.01215
C -0.71938 2.81184 0.01445
C 1.41071 4.02568 0.02189
C -1.41105 4.02558 0.02697
H 2.49724 4.00475 0.01962
H -2.49758 4.00458 0.02834
N -1.40821 1.64245 0.00353
N 1.40796 1.64252 0.00075
C 3.57796 -1.94175 -0.01700
C 5.01399 -1.99062 -0.01490
C 5.85561 -0.90691 -0.00315
H 5.44420 -2.99155 -0.02382
Ru 7.77390 -0.70617 -0.00102
H 5.40600 0.09397 0.00855
P 7.85822 -0.55259 -2.37524
P 7.86164 -0.62809 2.37661
C 8.02821 -2.50910 -0.02924
Cl 7.98245 1.71455 0.03682
C 6.64378 0.60874 -3.09090
C 9.47450 0.05484 -2.97809
C 7.58105 -2.10269 -3.30358

C 9.47930 -0.04202 2.99623
C 6.64884 0.50965 3.13168
C 7.58525 -2.20631 3.25633
O 8.23441 -3.65261 -0.04686
H 5.63153 0.24592 -2.88834
H 6.76747 1.58781 -2.62075
H 6.78760 0.69624 -4.17285
H 9.67475 1.03397 -2.53365
H 10.26723 -0.63694 -2.67735
H 9.46942 0.14320 -4.06977
H 6.60487 -2.51760 -3.03411
H 7.60705 -1.91732 -4.38221
H 8.35298 -2.83461 -3.04817
H 9.67971 0.95133 2.58471
H 9.47600 0.00918 4.09029
H 10.27084 -0.72399 2.67101
H 6.79289 0.55917 4.21597
H 6.77340 1.50432 2.69588
H 5.63618 0.15513 2.91683
H 7.61751 -2.05449 4.34004
H 6.60654 -2.61009 2.97921
H 8.35405 -2.93156 2.97404
C -0.72258 5.24042 0.03700
C 0.72219 5.24048 0.03424
C 1.40726 6.49262 0.04431
C 0.71186 7.66930 0.05624
C -0.71235 7.66925 0.05899
C -1.40769 6.49252 0.04972
H 2.49417 6.48980 0.04231
H 1.24388 8.61630 0.06375
H -1.24441 8.61620 0.06858
H -2.49460 6.48961 0.05190

**Atomic coordinates of *trans*-[$Ru_2$-3]$^+$ from its DFT structure**

Ru -8.07881 -0.73509 -0.00007
P -8.18773 -0.82860 -2.37540
O -7.89119 2.24158 0.00003
C -6.16055 -0.95614 0.00005
H -5.93966 -2.03102 -0.00001
P -8.18793 -0.82880 2.37524
C -5.10638 -0.07915 0.00018
H -5.31116 0.99070 0.00020
C -7.93670 1.08034 0.00000
C -7.25535 -2.21530 -3.11293
H -6.18945 -2.08659 -2.90138
H -7.59350 -3.15213 -2.66251
H -7.40729 -2.24847 -4.19682
C -9.89814 -1.05527 -2.98189

H -10.30885 -1.97394 -2.55334
H -10.51837 -0.21130 -2.66503
H -9.91507 -1.12207 -4.07480
C -7.57581 0.63627 -3.28144
H -6.53166 0.82074 -3.01030
H -7.64368 0.47758 -4.36263
H -8.16618 1.51685 -3.01147
C -9.89838 -1.05559 2.98160
H -10.30903 -1.97423 2.55292
H -9.91536 -1.12251 4.07451
H -10.51862 -0.21161 2.66481
C -7.25556 -2.21553 3.11274
H -7.40758 -2.24878 4.19661
H -7.59363 -3.15233 2.66223
H -6.18965 -2.08676 2.90129
C -7.57611 0.63602 3.28141
H -7.64400 0.47727 4.36259
H -6.53196 0.82055 3.01032
H -8.16649 1.51660 3.01147
C -3.71335 -0.43203 0.00027
C -3.22739 -1.76826 0.00046
C -1.88285 -2.03304 0.00049
H -3.92338 -2.60099 0.00060
C -1.39047 0.34309 0.00022
C -0.91077 -0.99842 0.00034
H -1.56909 -3.07035 0.00065
Cl -8.80166 -3.05613 -0.00020
C -2.75360 0.59806 0.00019
H -3.07873 1.63418 0.00009
C 0.50196 -1.26918 0.00032
C 1.02334 -2.58785 0.00036
C 1.44403 -0.19747 0.00024
C 2.37328 -2.82859 0.00034
H 0.35161 -3.43810 0.00039
C 2.80507 -0.45388 0.00022
H 2.73512 -3.85351 0.00037
H 3.47060 0.40230 0.00017
C 0.97286 1.19011 0.00018
C -0.44368 1.46004 0.00015
C 1.39964 3.43859 0.00007
C -0.01360 3.70757 0.00004
C 2.30610 4.50169 0.00003
C -0.46582 5.02955 -0.00002
H 3.36967 4.27828 0.00005
H -1.53700 5.21195 -0.00004
N -0.90972 2.68837 0.00009
N 1.85756 2.16097 0.00014
C 3.31732 -1.76679 0.00026
C 4.71831 -2.08236 0.00023
C 5.74846 -1.17455 0.00005
H 4.95456 -3.14572 0.00033

Ru 7.66824 -1.33900 -0.00007
H 5.49511 -0.10684 -0.00007
P 7.78251 -1.24008 -2.37609
P 7.78286 -1.23944 2.37591
C 7.58055 -3.15850 0.00017
Cl 8.32540 1.00036 -0.00044
C 6.81086 0.11877 -3.11410
C 9.48803 -0.96041 -2.97321
C 7.22045 -2.72236 -3.28598
C 9.48847 -0.95969 2.97273
C 6.81136 0.11963 3.11370
C 7.22089 -2.72148 3.28626
O 7.57051 -4.32037 0.00040
H 5.74838 -0.04318 -2.90827
H 7.11745 1.06436 -2.65949
H 6.96757 0.15958 -4.19704
H 9.86797 -0.02987 -2.54201
H 10.13220 -1.78530 -2.65402
H 9.50749 -0.89213 -4.06600
H 6.18090 -2.93887 -3.02106
H 7.28950 -2.56048 -4.36661
H 7.83625 -3.58460 -3.01369
H 9.86835 -0.02919 2.54139
H 9.50810 -0.89131 4.06551
H 10.13260 -1.78461 2.65351
H 6.96824 0.16075 4.19660
H 7.11790 1.06509 2.65876
H 5.74885 -0.04235 2.90808
H 7.29021 -2.55935 4.36684
H 6.18127 -2.93798 3.02163
H 7.83658 -3.58382 3.01403
C 0.43757 6.09419 -0.00006
C 1.85688 5.82386 -0.00004
C 2.76400 6.92584 -0.00008
C 2.30105 8.21188 -0.00014
C 0.90194 8.47836 -0.00017
C -0.00122 7.45246 -0.00013
H 3.83116 6.71969 -0.00006
H 3.00094 9.04261 -0.00017
H 0.55641 9.50819 -0.00022
H -1.06956 7.65285 -0.00015

**Atomic coordinates of $[Ru_2\text{-}3]^{2+}$ singlet from its DFT structure**

Ru 7.73078 -1.40749 -0.00298
P 7.97320 -1.52222 2.37388
O 8.10781 1.56028 -0.01403
C 5.85157 -1.28340 0.00636
H 5.42284 -2.29209 0.01475
P 7.95315 -1.53947 -2.38117
C 4.96943 -0.19822 0.00402

H 5.38187 0.80861 -0.00754
C 7.93478 0.41604 -0.00980
C 6.82604 -2.67934 3.19222
H 5.79993 -2.31843 3.07520
H 6.91371 -3.66258 2.72351
H 7.06391 -2.75382 4.25836
C 9.64419 -2.11355 2.81184
H 9.80631 -3.09528 2.35837
H 10.39799 -1.41717 2.43234
H 9.74329 -2.19192 3.89964
C 7.77090 0.04652 3.28511
H 6.76725 0.44587 3.11075
H 7.90988 -0.11796 4.35847
H 8.50549 0.77923 2.93882
C 9.61779 -2.14002 -2.83070
H 9.78055 -3.11893 -2.37142
H 9.70571 -2.22711 -3.91880
H 10.37795 -1.44372 -2.46409
C 6.79502 -2.69902 -3.18034
H 7.02219 -2.78126 -4.24827
H 6.88503 -3.67926 -2.70578
H 5.77106 -2.33477 -3.05532
C 7.74736 0.02342 -3.30163
H 7.87299 -0.14949 -4.37530
H 6.74768 0.42881 -3.11841
H 8.48946 0.75504 -2.96932
C 3.56512 -0.28833 0.01295
C 2.83169 -1.52133 0.03395
C 1.47197 -1.52461 0.03204
H 3.35849 -2.46875 0.05334
C 1.43784 0.92757 0.00397
C 0.70586 -0.31316 0.00848
H 0.96503 -2.48101 0.05494
Cl 7.92222 -3.81663 0.00441
C 2.81089 0.91815 0.00352
H 3.33164 1.87066 -0.00472
C -0.70584 -0.31320 -0.00840
C -1.47190 -1.52468 -0.03186
C -1.43787 0.92751 -0.00402
C -2.83162 -1.52146 -0.03388
H -0.96492 -2.48106 -0.05458
C -2.81092 0.91802 -0.00370
H -3.35838 -2.46891 -0.05319
H -3.33171 1.87051 0.00445
C -0.71850 2.20341 0.00073
C 0.71840 2.20345 -0.00076
C -0.72033 4.48782 0.00188
C 0.72013 4.48785 -0.00184
C -1.41216 5.70120 0.00398
C 1.41190 5.70127 -0.00390
H -2.49831 5.68003 0.00682

H 2.49806 5.68015 -0.00675
N 1.40921 3.31971 -0.00260
N -1.40935 3.31965 0.00260
C -3.56510 -0.28849 -0.01310
C -4.96941 -0.19841 -0.00428
C -5.85155 -1.28360 -0.00651
H -5.38186 0.80841 0.00715
Ru -7.73077 -1.40756 0.00298
H -5.42283 -2.29229 -0.01486
P -7.97332 -1.52218 -2.37386
P -7.95301 -1.53963 2.38117
C -7.93459 0.41600 0.00984
Cl -7.92187 -3.81673 -0.00447
C -6.82618 -2.67929 -3.19226
C -9.64429 -2.11353 -2.81189
C -7.77106 0.04660 -3.28502
C -9.61767 -2.14007 2.83077
C -6.79494 -2.69926 3.18031
C -7.74707 0.02320 3.30170
O -8.10750 1.56026 0.01393
H -5.80008 -2.31834 -3.07538
H -6.91378 -3.66251 -2.72349
H -7.06417 -2.75385 -4.25838
H -9.80642 -3.09526 -2.35843
H -10.39815 -1.41716 -2.43248
H -9.74328 -2.19192 -3.89970
H -6.76740 0.44593 -3.11068
H -7.91009 -0.11784 -4.35837
H -8.50563 0.77928 -2.93865
H -9.78073 -3.11878 2.37117
H -9.70535 -2.22757 3.91887
H -10.37778 -1.44348 2.46461
H -7.02215 -2.78152 4.24823
H -6.88497 -3.67949 2.70572
H -5.77096 -2.33506 3.05534
H -7.87283 -0.14975 4.37535
H -6.74731 0.42843 3.11860
H -8.48902 0.75496 2.96937
C 0.72244 6.91588 -0.00206
C -0.72276 6.91584 0.00217
C -1.40830 8.16673 0.00422
C -0.71199 9.34352 0.00219
C 0.71155 9.34355 -0.00204
C 1.40792 8.16680 -0.00409
H -2.49499 8.16455 0.00741
H -1.24367 10.29053 0.00379
H 1.24318 10.29059 -0.00361
H 2.49461 8.16467 -0.00729

**Atomic coordinates of $[Ru_2\text{-}3]^{2+}$ triplet from its DFT structure**

Ru 7.78011 -1.38658 -0.00994
P 8.12961 -1.51883 2.35874
O 8.10669 1.59005 -0.00159
C 5.89058 -1.30033 0.03313
H 5.48178 -2.31638 0.04431
P 8.00685 -1.49561 -2.39574
C 5.00770 -0.22853 0.03901
H 5.40991 0.78281 0.03164
C 7.95106 0.44488 -0.00465
C 7.02529 -2.69663 3.20384
H 5.99267 -2.34379 3.12753
H 7.10527 -3.67382 2.72157
H 7.30274 -2.77900 4.25978
C 9.82676 -2.09214 2.70643
H 9.97420 -3.07441 2.24951
H 10.55156 -1.38973 2.28396
H 9.98456 -2.16309 3.78775
C 7.94819 0.04210 3.28489
H 6.93362 0.43009 3.15423
H 8.13365 -0.12751 4.35042
H 8.65980 0.78431 2.91206
C 9.67887 -2.07689 -2.83953
H 9.84764 -3.05877 -2.38908
H 9.77246 -2.15225 -3.92801
H 10.43061 -1.37698 -2.46262
C 6.85535 -2.66067 -3.19410
H 7.07733 -2.72999 -4.26406
H 6.95942 -3.64386 -2.72883
H 5.82873 -2.30798 -3.05923
C 7.78444 0.07479 -3.29711
H 7.91067 -0.08631 -4.37258
H 6.78055 0.46673 -3.10846
H 8.51939 0.81051 -2.95825
C 3.57109 -0.31749 0.04212
C 2.85485 -1.54050 0.05611
C 1.47979 -1.54636 0.04292
H 3.38210 -2.48829 0.07797
C 1.43859 0.87673 0.01345
C 0.73076 -0.34895 0.01357
H 0.97561 -2.50503 0.05926
Cl 8.00716 -3.78744 -0.02854
C 2.83315 0.87504 0.02591
H 3.34465 1.83271 0.02139
C -0.73075 -0.34896 -0.01362
C -1.47976 -1.54638 -0.04281
C -1.43858 0.87671 -0.01366
C -2.85482 -1.54054 -0.05603
H -0.97557 -2.50505 -0.05900
C -2.83314 0.87500 -0.02616
H -3.38205 -2.48834 -0.07774

H -3.34465 1.83268 -0.02177
C -0.71980 2.15075 -0.00371
C 0.71979 2.15076 0.00342
C -0.72003 4.43703 -0.00260
C 0.72000 4.43704 0.00225
C -1.41139 5.65162 -0.00466
C 1.41134 5.65164 0.00430
H -2.49767 5.63046 -0.00826
H 2.49762 5.63049 0.00789
N 1.40966 3.26909 0.00562
N -1.40968 3.26908 -0.00594
C -3.57107 -0.31753 -0.04222
C -5.00768 -0.22857 -0.03912
C -5.89057 -1.30037 -0.03311
H -5.40990 0.78277 -0.03186
Ru -7.78010 -1.38658 0.01002
H -5.48177 -2.31642 -0.04415
P -8.12970 -1.51957 -2.35863
P -8.00675 -1.49487 2.39584
C -7.95099 0.44488 0.00426
Cl -8.00724 -3.78743 0.02937
C -7.02558 -2.69781 -3.20336
C -9.82693 -2.09274 -2.70615
C -7.94801 0.04104 -3.28530
C -9.67865 -2.07632 2.83991
C -6.85502 -2.65945 3.19458
C -7.78463 0.07585 3.29673
O -8.10658 1.59005 0.00075
H -5.99289 -2.34514 -3.12715
H -7.10574 -3.67485 -2.72082
H -7.30303 -2.78041 -4.25928
H -9.97448 -3.07483 -2.24889
H -10.55165 -1.39009 -2.28393
H -9.98472 -2.16407 -3.78744
H -6.93342 0.42895 -3.15464
H -8.13336 -0.12890 -4.35080
H -8.65959 0.78344 -2.91280
H -9.84724 -3.05840 2.38982
H -9.77218 -2.15131 3.92841
H -10.43053 -1.37670 2.46277
H -7.07718 -2.72863 4.26452
H -6.95869 -3.64277 2.72949
H -5.82849 -2.30646 3.05984
H -7.91075 -0.08497 4.37224
H -6.78085 0.46797 3.10787
H -8.51979 0.81130 2.95770
C 0.72277 6.86550 0.00204
C -0.72283 6.86549 -0.00242
C -1.40805 8.11799 -0.00455
C -0.71242 9.29406 -0.00241
C 0.71233 9.29406 0.00202

C 1.40797 8.11800 0.00417
H -2.49488 8.11556 -0.00791
H -1.24393 10.24128 -0.00407
H 1.24383 10.24129 0.00367
H 2.49480 8.11559 0.00752

**Atomic coordinates of $[Ru_2\text{-}7]^-$ from its DFT structure**

Ru -5.73261 -1.66819 0.03692
P -5.72445 -2.18616 -2.26386
O -6.08949 1.23332 -0.50185
C -3.74293 -1.50741 -0.01658
H -3.33955 -2.51692 0.14943
P -5.62617 -1.30578 2.36656
C -2.89865 -0.46828 -0.17358
H -3.30628 0.53148 -0.31046
C -5.91914 0.09633 -0.29201
C -4.56498 -3.52820 -2.71615
H -3.54559 -3.21616 -2.46963
H -4.80777 -4.42036 -2.13221
H -4.62679 -3.75647 -3.78554
C -7.32816 -2.76824 -2.93687
H -7.65491 -3.64434 -2.36856
H -8.08141 -1.98177 -2.82619
H -7.24046 -3.03392 -3.99605
C -5.24850 -0.82964 -3.39648
H -4.27319 -0.43969 -3.08874
H -5.19006 -1.17947 -4.43247
H -5.97979 -0.01811 -3.33229
C -7.19693 -1.57112 3.27526
H -7.54589 -2.59171 3.09117
H -7.06100 -1.42254 4.35209
H -7.95689 -0.87285 2.91072
C -4.44764 -2.40638 3.23225
H -4.46388 -2.22480 4.31222
H -4.71388 -3.44702 3.02735
H -3.44029 -2.22063 2.84780
C -5.09570 0.36361 2.89556
H -4.98173 0.41681 3.98332
H -4.13881 0.59561 2.41739
H -5.83169 1.10715 2.57478
Ru 5.73786 -1.65529 0.02791
P 5.62333 -1.86993 2.37402
O 6.06986 1.29366 0.20383
C 3.74742 -1.50211 -0.02130
H 3.35473 -2.52732 -0.08454
P 5.74986 -1.60514 -2.33092
C 2.89582 -0.45714 0.00145
H 3.29699 0.55298 0.05776

C 5.90910 0.13826 0.13369
C 4.44231 -3.14660 2.94342
H 3.43577 -2.87344 2.61275
H 4.71032 -4.10544 2.49106
H 4.45564 -3.23426 4.03508
C 7.19386 -2.35691 3.18582
H 7.53888 -3.30000 2.75117
H 7.95550 -1.59176 3.00547
H 7.05957 -2.48164 4.26591
C 5.10067 -0.38071 3.29998
H 4.13854 -0.04010 2.90480
H 4.99957 -0.59429 4.36916
H 5.83338 0.42012 3.16112
C 7.36148 -2.00384 -3.11008
H 7.68716 -2.99014 -2.76605
H 7.28294 -2.00740 -4.20277
H 8.11061 -1.26538 -2.80750
C 4.60143 -2.80401 -3.10100
H 4.66969 -2.76639 -4.19346
H 4.84710 -3.80972 -2.74884
H 3.57931 -2.56531 -2.79206
C 5.27484 -0.01937 -3.11091
H 5.23183 -0.11231 -4.20112
H 4.29225 0.27964 -2.73278
H 5.99823 0.75741 -2.84494
C -1.43553 -0.49920 -0.14379
C -0.69099 -1.69101 -0.13533
C -0.72373 0.73657 -0.11481
C 0.69356 -1.68867 -0.08914
H -1.21380 -2.64457 -0.17442
C 0.71630 0.73901 -0.06101
C 1.43343 -0.49443 -0.04871
H 1.21985 -2.64089 -0.08604
Cl -6.17875 -4.03978 0.49522
Cl 6.19606 -4.06553 -0.09253
N 1.40658 1.91030 -0.01807
C 0.69707 3.05520 -0.03674
C -0.71490 3.05289 -0.10448
C 1.41517 4.31583 0.01719
C -1.43752 4.31138 -0.14687
N -1.41982 1.90519 -0.13359
C 0.69828 5.54710 -0.01895
C 2.81343 4.35403 0.10646
C -2.83607 4.34584 -0.23436
C -0.72471 5.54492 -0.10388
C 1.40691 6.78172 0.02988
C 3.49744 5.56544 0.15654
C -3.52396 5.55531 -0.27744
C -1.43727 6.77752 -0.14614
C 0.66106 8.00672 -0.01295
C 2.80676 6.77224 0.11705

H 4.58271 5.56658 0.22636
C -2.83713 6.76411 -0.23264
H -4.60927 5.55330 -0.34660
C -0.69533 8.00470 -0.09777
H 1.20968 8.94560 0.02359
H 3.34312 7.71791 0.15407
H -3.37650 7.70824 -0.26498
H -1.24701 8.94194 -0.13015
H -3.37155 3.40218 -0.27086
H 3.35142 3.41157 0.13820

**Atomic coordinates of $Ru_2$-7 from its DFT structure**

Ru -5.72478 -1.66392 -0.00987
P -5.65993 -1.91002 -2.36008
O -6.09640 1.28115 -0.21313
C -3.75316 -1.48446 0.01306
H -3.33421 -2.49705 0.09798
P -5.74760 -1.59613 2.35334
C -2.91589 -0.42747 -0.05093
H -3.32666 0.57665 -0.12053
C -5.92500 0.12945 -0.13340
C -4.45859 -3.15948 -2.94500
H -3.44986 -2.85012 -2.65486
H -4.68098 -4.11810 -2.46849
H -4.50595 -3.26662 -4.03389
C -7.24209 -2.44987 -3.10993
H -7.54680 -3.39588 -2.65258
H -8.01752 -1.70257 -2.91472
H -7.13863 -2.58539 -4.19199
C -5.20781 -0.41812 -3.31807
H -4.24076 -0.04794 -2.96332
H -5.13941 -0.64251 -4.38753
H -5.95592 0.36519 -3.16295
C -7.35536 -2.04652 3.10754
H -7.63697 -3.04820 2.76947
H -7.29352 -2.03479 4.20109
H -8.12626 -1.34010 2.78433
C -4.56598 -2.74854 3.14231
H -4.65505 -2.71154 4.23320
H -4.76770 -3.76328 2.78856
H -3.54752 -2.47280 2.85231
C -5.33830 0.01169 3.12543
H -5.30750 -0.07329 4.21662
H -4.36125 0.34407 2.76073
H -6.08504 0.76082 2.84521
Ru 5.72467 -1.66387 0.01021
P 5.65889 -1.90919 2.36047
O 6.09619 1.28127 0.21261

C 3.75306 -1.48448 -0.01359
H 3.33420 -2.49714 -0.09810
P 5.74860 -1.59684 -2.35301
C 2.91573 -0.42748 0.04959
H 3.32647 0.57667 0.11889
C 5.92477 0.12955 0.13319
C 4.45768 -3.15880 2.94532
H 3.44897 -2.84987 2.65467
H 4.68055 -4.11753 2.46925
H 4.50464 -3.26554 4.03427
C 7.24089 -2.44837 3.11113
H 7.54600 -3.39445 2.65417
H 8.01621 -1.70094 2.91599
H 7.13702 -2.58358 4.19319
C 5.20590 -0.41709 3.31772
H 4.23893 -0.04731 2.96232
H 5.13701 -0.64110 4.38722
H 5.95387 0.36638 3.16268
C 7.35706 -2.04652 -3.10613
H 7.63896 -3.04802 -2.76776
H 7.29596 -2.03492 -4.19973
H 8.12740 -1.33968 -2.78247
C 4.56808 -2.75017 -3.14229
H 4.65780 -2.71348 -4.23313
H 4.77011 -3.76469 -2.78809
H 3.54930 -2.47486 -2.85301
C 5.33877 0.01049 -3.12585
H 5.30829 -0.07496 -4.21701
H 4.36147 0.34255 -2.76153
H 6.08508 0.76007 -2.84573
C -1.45556 -0.47117 -0.02612
C -0.70062 -1.63717 -0.01484
C -0.71819 0.76110 -0.01462
C 0.70047 -1.63718 0.01276
H -1.20722 -2.59873 -0.02854
C 0.71805 0.76109 0.01325
C 1.45541 -0.47120 0.02451
H 1.20705 -2.59876 0.02606
Cl -6.08688 -4.07932 0.14542
Cl 6.08773 -4.07919 -0.14398
N 1.39068 1.92700 0.02959
C 0.71513 3.06260 0.01737
C -0.71524 3.06261 -0.01816
C 1.44669 4.32699 0.04094
C -1.44678 4.32702 -0.04143
N -1.39080 1.92702 -0.03067
C 0.71730 5.54332 0.02087
C 2.83921 4.35680 0.08354
C -2.83929 4.35687 -0.08390
C -0.71736 5.54334 -0.02114
C 1.42034 6.77905 0.04253

C 3.52334 5.57275 0.10554
C -3.52341 5.57284 -0.10560
C -1.42037 6.77908 -0.04258
C 0.67876 8.00584 0.02059
C 2.82488 6.76926 0.08485
H 4.60920 5.57826 0.13902
C -2.82492 6.76933 -0.08479
H -4.60926 5.57838 -0.13901
C -0.67877 8.00586 -0.02042
H 1.23067 8.94238 0.03729
H 3.35796 7.71674 0.10161
H -3.35798 7.71683 -0.10135
H -1.23066 8.94241 -0.03705
H -3.38169 3.41695 -0.10066
H 3.38158 3.41687 0.10020

**Atomic coordinates of $[Ru_2\text{-}7]^+$ from its DFT structure**

Ru -5.63047 -1.66149 -0.00558
P -5.71605 -1.86683 -2.37757
O -6.04165 1.29242 -0.11603
C -3.71751 -1.48844 0.00394
H -3.27781 -2.49322 0.03419
P -5.75615 -1.68689 2.37261
C -2.87257 -0.39929 -0.01471
H -3.29600 0.60098 -0.03415
C -5.85731 0.14635 -0.07358
C -4.50631 -3.04708 -3.06925
H -3.49276 -2.67908 -2.88306
H -4.62791 -4.01307 -2.57223
H -4.65695 -3.16358 -4.14751
C -7.33631 -2.48556 -2.95712
H -7.53515 -3.45394 -2.48917
H -8.12664 -1.78593 -2.66848
H -7.33687 -2.59912 -4.04646
C -5.44043 -0.33766 -3.34018
H -4.46329 0.08312 -3.08365
H -5.47051 -0.54619 -4.41443
H -6.21117 0.40010 -3.09835
C -7.38903 -2.25604 2.96637
H -7.58303 -3.25702 2.57067
H -7.41104 -2.28484 4.06097
H -8.17038 -1.57837 2.60910
C -4.56182 -2.81101 3.17536
H -4.72467 -2.83300 4.25791
H -4.68374 -3.81553 2.76199
H -3.54409 -2.46563 2.96955
C -5.49477 -0.09056 3.22323
H -5.55167 -0.21965 4.30885

H -4.50940 0.30604 2.95981
H -6.25560 0.62994 2.90912
Ru 5.63051 -1.66135 0.00537
P 5.71823 -1.86570 2.37735
O 6.04178 1.29260 0.11421
C 3.71755 -1.48831 -0.00257
H 3.27786 -2.49311 -0.03220
P 5.75407 -1.68779 -2.37294
C 2.87259 -0.39918 0.01634
H 3.29601 0.60111 0.03515
C 5.85742 0.14651 0.07242
C 4.50939 -3.04587 3.07073
H 3.49558 -2.67821 2.88528
H 4.63077 -4.01209 2.57412
H 4.66106 -3.16177 4.14892
C 7.33918 -2.48395 2.95543
H 7.53770 -3.45243 2.48754
H 8.12909 -1.78427 2.66576
H 7.34090 -2.59723 4.04480
C 5.44325 -0.33617 3.33957
H 4.46581 0.08432 3.08372
H 5.47432 -0.54423 4.41389
H 6.21365 0.40162 3.09674
C 7.38642 -2.25706 -2.96802
H 7.58070 -3.25803 -2.57244
H 7.40750 -2.28588 -4.06264
H 8.16811 -1.57942 -2.61143
C 4.55905 -2.81235 -3.17403
H 4.72126 -2.83533 -4.25665
H 4.68100 -3.81654 -2.75985
H 3.54151 -2.46657 -2.96793
C 5.49180 -0.09187 -3.22406
H 5.54772 -0.22149 -4.30967
H 4.50666 0.30482 -2.95994
H 6.25289 0.62881 -2.91100
C -1.45157 -0.44453 -0.00638
C -0.68923 -1.63302 -0.00143
C -0.71171 0.80243 -0.00450
C 0.68928 -1.63298 0.00486
H -1.19990 -2.59068 -0.00469
C 0.71169 0.80247 0.00617
C 1.45159 -0.44447 0.00867
H 1.19999 -2.59062 0.00900
Cl -5.84541 -4.07939 0.08310
Cl 5.84565 -4.07927 -0.08253
N 1.38956 1.95755 0.01266
C 0.71541 3.09829 0.00757
C -0.71552 3.09826 -0.00705
C 1.44784 4.35791 0.01673
C -1.44803 4.35783 -0.01677
N -1.38963 1.95748 -0.01161

C 0.71673 5.57386 0.00823
C 2.84143 4.38567 0.03370
C -2.84163 4.38550 -0.03378
C -0.71698 5.57383 -0.00878
C 1.41977 6.80933 0.01658
C 3.52476 5.60142 0.04212
C -3.52502 5.60121 -0.04271
C -1.42009 6.80925 -0.01763
C 0.67877 8.03647 0.00747
C 2.82468 6.79760 0.03355
H 4.61076 5.60820 0.05548
C -2.82500 6.79744 -0.03461
H -4.61102 5.60794 -0.05608
C -0.67915 8.03644 -0.00900
H 1.23091 8.97271 0.01396
H 3.35873 7.74445 0.04007
H -3.35910 7.74426 -0.04151
H -1.23133 8.97265 -0.01586
H -3.38566 3.44660 -0.04036
H 3.38552 3.44680 0.04066

**Atomic coordinates of *cis*-[$Ru_2$-7]$^+$ from its DFT structure**

Ru -5.77545 -1.14560 0.00468
P -5.90001 -1.04584 2.38068
O -5.84043 -4.12654 -0.00430
C -3.85907 -1.06858 0.01021
H -3.53381 -0.02652 0.02659
P -5.88232 -1.03461 -2.37158
C -2.87390 -2.03531 -0.00451
H -3.19141 -3.07783 -0.01390
C -5.78337 -2.96565 -0.00067
C -4.89060 0.28416 3.12018
H -3.83297 0.09127 2.91631
H -5.16803 1.23815 2.66435
H -5.04919 0.33010 4.20263
C -7.59811 -0.71503 2.97209
H -7.94864 0.22587 2.53849
H -8.26565 -1.52082 2.65212
H -7.61817 -0.64431 4.06477
C -5.38526 -2.54462 3.29127
H -4.35201 -2.79267 3.02927
H -5.45258 -2.38115 4.37176
H -6.02630 -3.38750 3.01676
C -7.57585 -0.70035 -2.97394
H -7.92914 0.23882 -2.53885
H -7.58801 -0.62497 -4.06643
H -8.24600 -1.50724 -2.66217

C -4.86602 0.29737 -3.09810
H -5.01583 0.34738 -4.18165
H -5.14591 1.25008 -2.64113
H -3.81028 0.10253 -2.88645
C -5.36191 -2.52989 -3.28468
H -5.42147 -2.36187 -4.36491
H -4.33063 -2.77952 -3.01642
H -6.00538 -3.37351 -3.01829
Ru 5.77548 -1.14564 -0.00456
P 5.90004 -1.04589 -2.38054
O 5.84038 -4.12658 0.00454
C 3.85911 -1.06854 -0.01021
H 3.53383 -0.02648 -0.02635
P 5.88226 -1.03456 2.37172
C 2.87393 -2.03528 0.00411
H 3.19145 -3.07780 0.01330
C 5.78336 -2.96570 0.00086
C 4.89063 0.28413 -3.12004
H 3.83299 0.09122 -2.91625
H 5.16803 1.23810 -2.66416
H 5.04930 0.33011 -4.20248
C 7.59809 -0.71511 -2.97210
H 7.94856 0.22593 -2.53877
H 8.26573 -1.52076 -2.65197
H 7.61808 -0.64468 -4.06480
C 5.38521 -2.54470 -3.29106
H 4.35205 -2.79285 -3.02883
H 5.45227 -2.38122 -4.37156
H 6.02641 -3.38751 -3.01670
C 7.57577 -0.70020 2.97407
H 7.92898 0.23900 2.53899
H 7.58795 -0.62485 4.06656
H 8.24596 -1.50704 2.66227
C 4.86588 0.29746 3.09806
H 5.01580 0.34773 4.18158
H 5.14559 1.25011 2.64083
H 3.81014 0.10242 2.88656
C 5.36185 -2.52976 3.28493
H 5.42140 -2.36166 4.36515
H 4.33057 -2.77941 3.01668
H 6.00531 -3.37342 3.01861
C -1.45812 -1.87599 -0.00466
C -0.68830 -3.05887 -0.00253
C -0.71633 -0.62614 -0.00600
C 0.68835 -3.05887 0.00179
H -1.21237 -4.01091 -0.00355
C 0.71636 -0.62613 0.00566
C 1.45816 -1.87598 0.00418
H 1.21242 -4.01090 0.00265
Cl -6.37098 1.21383 0.00715
Cl 6.37096 1.21380 -0.00703

N 1.38279 0.53510 0.01954
C 0.71356 1.68071 0.01376
C -0.71353 1.68071 -0.01399
C 1.44794 2.93986 0.03484
C -1.44791 2.93986 -0.03500
N -1.38276 0.53510 -0.01986
C 0.71656 4.15634 0.01778
C 2.84122 2.97345 0.07057
C -2.84119 2.97344 -0.07069
C -0.71654 4.15634 -0.01791
C 1.41884 5.39280 0.03581
C 3.52381 4.18909 0.08828
C -3.52379 4.18909 -0.08834
C -1.41881 5.39280 -0.03588
C 0.67863 6.62002 0.01726
C 2.82321 5.38429 0.07099
H 4.60968 4.19014 0.11523
C -2.82319 5.38429 -0.07103
H -4.60965 4.19013 -0.11524
C -0.67861 6.62002 -0.01731
H 1.23125 7.55587 0.03127
H 3.35516 6.33225 0.08462
H -3.35514 6.33225 -0.08461
H -1.23124 7.55587 -0.03128
H -3.40114 2.04547 -0.08540
H 3.40116 2.04547 0.08525

**Atomic coordinates of *trans*-[Ru$_2$-7]$^+$ from its DFT structure**

Ru 5.44478 -1.83573 0.03852
P 5.56362 -2.07531 -2.32738
O 4.78179 -4.71797 0.41861
C 3.60530 -1.29489 -0.00393
H 3.54385 -0.21194 -0.13157
P 5.60364 -1.45616 2.38529
C 2.41381 -1.98557 0.09858
H 2.46459 -3.07019 0.19302
C 5.00924 -3.58790 0.27020
C 4.91590 -0.64216 -3.25497
H 3.84674 -0.53150 -3.04974
H 5.43273 0.26147 -2.92163
H 5.06648 -0.78099 -4.33062
C 7.28520 -2.26343 -2.91384
H 7.86956 -1.39869 -2.58684
H 7.72529 -3.16896 -2.48510
H 7.31485 -2.33224 -4.00638
C 4.67970 -3.50273 -3.04955
H 3.61886 -3.44563 -2.78700
H 4.77979 -3.50739 -4.13981
H 5.08746 -4.43537 -2.64884

C 7.33315 -1.47502 2.97784
H 7.90228 -0.71299 2.43781
H 7.37398 -1.26825 4.05258
H 7.78183 -2.45377 2.78312
C 4.95723 0.16562 2.92017
H 5.11936 0.30736 3.99368
H 5.46644 0.95512 2.36147
H 3.88572 0.21824 2.70473
C 4.73906 -2.65124 3.46449
H 4.84598 -2.36813 4.51668
H 3.67615 -2.67381 3.20478
H 5.15412 -3.65300 3.32002
Ru -6.08393 -0.89068 0.00202
P -6.16154 -1.00517 -2.37532
O -5.74465 2.07412 -0.02692
C -4.18806 -1.19864 0.04293
H -4.01224 -2.28135 0.07096
P -6.27234 -0.94859 2.37573
C -3.09887 -0.35428 0.03516
H -3.25881 0.71950 0.00165
C -5.85276 0.91762 -0.01481
C -5.26068 -2.42528 -3.08694
H -4.19351 -2.32303 -2.86750
H -5.62852 -3.34699 -2.62866
H -5.40411 -2.46729 -4.17166
C -7.87168 -1.19048 -2.99532
H -8.31001 -2.09499 -2.56411
H -8.47251 -0.32827 -2.69060
H -7.87971 -1.26492 -4.08783
C -5.49902 0.43404 -3.28643
H -4.45197 0.59061 -3.00890
H -5.56312 0.26858 -4.36682
H -6.06570 1.33353 -3.02835
C -8.00967 -1.12310 2.91919
H -8.42854 -2.03539 2.48511
H -8.06735 -1.17755 4.01141
H -8.59537 -0.26642 2.57191
C -5.40388 -2.35053 3.16011
H -5.59939 -2.36961 4.23726
H -5.74723 -3.28298 2.70455
H -4.32754 -2.24961 2.99041
C -5.65631 0.51145 3.28648
H -5.76903 0.36662 4.36590
H -4.59841 0.66709 3.05362
H -6.21370 1.40346 2.98561
C 1.08421 -1.47601 0.08736
C 0.03797 -2.42685 0.13668
C 0.68492 -0.08133 0.04101
C -1.29894 -2.09484 0.12001
H 0.31174 -3.47767 0.18266
C -0.70102 0.26247 0.02906

C -1.73491 -0.75657 0.06094
H -2.02967 -2.89641 0.15345
Cl 6.59081 0.29066 -0.24938
Cl -6.89089 -3.18241 0.01049
N -1.08136 1.54465 -0.00882
C -0.15933 2.49525 -0.03223
C 1.22911 2.15721 -0.01682
C -0.57885 3.88975 -0.07015
C 2.23677 3.21065 -0.03875
N 1.61153 0.88689 0.02008
C 0.41576 4.90056 -0.09266
C -1.92775 4.24105 -0.08343
C 3.59986 2.91821 -0.02412
C 1.80937 4.56423 -0.07739
C 0.02019 6.26547 -0.12994
C -2.30870 5.58210 -0.11958
C 4.54735 3.94103 -0.04752
C 2.78077 5.60231 -0.10043
C 1.02666 7.28571 -0.15313
C -1.34869 6.58153 -0.14291
H -3.36323 5.84185 -0.12943
C 4.14487 5.26637 -0.08475
H 5.60409 3.68984 -0.03708
C 2.34677 6.96800 -0.13924
H 0.70806 8.32455 -0.18205
H -1.64662 7.62658 -0.17125
H 4.88360 6.06380 -0.10230
H 3.10311 7.74834 -0.15737
H 3.92623 1.88478 0.00563
H -2.67587 3.45530 -0.06470

**Atomic coordinates of $[Ru_2\text{-}7]^{2+}$ singlet from its DFT structure**

Ru -5.55604 -1.65608 -0.02339
P -5.77970 -1.74555 -2.40622
O -5.96158 1.31177 0.01507
C -3.69672 -1.51555 0.02398
H -3.25020 -2.51617 0.02813
P -5.90033 -1.82004 2.33934
C -2.83464 -0.39678 0.04092
H -3.26646 0.59953 0.03933
C -5.77898 0.17043 -0.00001
C -4.62261 -2.88555 -3.23193
H -3.60025 -2.51475 -3.11413
H -4.70054 -3.87335 -2.77148
H -4.86210 -2.95296 -4.29817
C -7.44860 -2.34108 -2.84332
H -7.60323 -3.32986 -2.40292
H -8.20520 -1.65368 -2.45315

H -7.54975 -2.40523 -3.93188
C -5.58559 -0.16225 -3.29161
H -4.58731 0.24410 -3.10355
H -5.71459 -0.31399 -4.36808
H -6.33077 0.55793 -2.94186
C -7.59363 -2.41653 2.66780
H -7.72540 -3.39799 2.20432
H -7.75966 -2.49593 3.74736
H -8.32395 -1.72060 2.24389
C -4.79168 -2.99216 3.18656
H -5.09357 -3.10419 4.23299
H -4.83752 -3.96015 2.68183
H -3.76569 -2.61477 3.14571
C -5.74830 -0.26719 3.28457
H -5.93742 -0.45331 4.34669
H -4.73979 0.13993 3.16519
H -6.47015 0.46765 2.91695
Ru 5.55674 -1.65804 0.01566
P 5.79216 -1.76274 2.39702
O 5.96240 1.30997 -0.00626
C 3.69701 -1.51781 -0.01542
H 3.25030 -2.51836 -0.02191
P 5.88225 -1.80602 -2.35092
C 2.83507 -0.39878 -0.02096
H 3.26729 0.59727 -0.03368
C 5.77938 0.16861 0.00243
C 4.63825 -2.90811 3.21986
H 3.61566 -2.53535 3.11055
H 4.71271 -3.89224 2.75104
H 4.88330 -2.98441 4.28424
C 7.46247 -2.36099 2.82457
H 7.61572 -3.34688 2.37726
H 8.21771 -1.67100 2.43642
H 7.56715 -2.43222 3.91236
C 5.60156 -0.18520 3.29353
H 4.60215 0.22199 3.11338
H 5.73620 -0.34379 4.36831
H 6.34454 0.53761 2.94455
C 7.57332 -2.39815 -2.69812
H 7.71063 -3.38168 -2.24064
H 7.72974 -2.47211 -3.77948
H 8.30632 -1.70322 -2.27718
C 4.76765 -2.97371 -3.19645
H 5.05833 -3.07527 -4.24711
H 4.82176 -3.94608 -2.70108
H 3.74105 -2.59982 -3.14089
C 5.71925 -0.24703 -3.28430
H 5.89811 -0.42551 -4.34950
H 4.71147 0.15792 -3.15216
H 6.44371 0.48634 -2.91891
C -1.45043 -0.44569 0.04269

C -0.67882 -1.65977 0.04762
C -0.70560 0.81492 0.03180
C 0.67890 -1.66021 0.02876
H -1.19409 -2.61361 0.06825
C 0.70691 0.81437 0.00164
C 1.45095 -0.44675 -0.00046
H 1.19400 -2.61433 0.03601
Cl -5.70650 -4.06369 -0.06604
Cl 5.70935 -4.06570 0.04138
N 1.38848 1.95886 -0.02397
C 0.71647 3.10583 -0.01383
C -0.71376 3.10640 0.03029
C 1.44944 4.35975 -0.04675
C -1.44596 4.36092 0.05669
N -1.38652 1.95995 0.04723
C 0.71773 5.57635 -0.02420
C 2.84310 4.38575 -0.09933
C -2.83959 4.38799 0.10956
C -0.71348 5.57693 0.02864
C 1.42057 6.81138 -0.05357
C 3.52486 5.60100 -0.12904
C -3.52058 5.60379 0.13412
C -1.41554 6.81251 0.05298
C 0.68137 8.03923 -0.02854
C 2.82431 6.79746 -0.10615
H 4.60994 5.60872 -0.16999
C -2.81928 6.79970 0.10593
H -4.60564 5.61239 0.17544
C -0.67556 8.03977 0.02293
H 1.23363 8.97491 -0.05125
H 3.35892 7.74355 -0.12898
H -3.35327 7.74622 0.12498
H -1.22724 8.97589 0.04195
H -3.38551 3.45055 0.13226
H 3.38850 3.44791 -0.11734

**Atomic coordinates of $[Ru_2\text{-}7]^{2+}$ triplet from its DFT structure**

Ru -5.62629 -1.63360 -0.01465
P -5.86379 -1.70663 -2.40179
O -5.97590 1.34148 0.03293
C -3.73193 -1.53109 0.04992
H -3.31452 -2.54349 0.04715
P -6.01485 -1.79960 2.34621
C -2.87860 -0.44270 0.08311
H -3.28978 0.56252 0.08562
C -5.81119 0.19786 0.01484
C -4.70768 -2.85199 -3.22137
H -3.68314 -2.49432 -3.08441

H -4.80409 -3.84333 -2.77231
H -4.93322 -2.90458 -4.29158
C -7.53554 -2.28806 -2.84414
H -7.69724 -3.27812 -2.40931
H -8.28765 -1.59753 -2.45100
H -7.63535 -2.34526 -3.93317
C -5.65040 -0.11961 -3.27479
H -4.64829 0.27328 -3.07887
H -5.77609 -0.26275 -4.35285
H -6.38901 0.60626 -2.92316
C -7.71324 -2.38836 2.65720
H -7.84694 -3.36566 2.18572
H -7.88711 -2.47409 3.73501
H -8.43599 -1.68492 2.23310
C -4.91416 -2.98211 3.18902
H -5.20905 -3.08427 4.23854
H -4.97773 -3.95162 2.68924
H -3.88382 -2.61870 3.13607
C -5.85651 -0.24944 3.29352
H -6.05536 -0.43500 4.35398
H -4.84268 0.14633 3.18184
H -6.56803 0.49295 2.92100
Ru 5.62632 -1.63348 -0.01472
P 6.01528 -1.79994 2.34606
O 5.97597 1.34159 0.03342
C 3.73198 -1.53092 0.04981
H 3.31455 -2.54332 0.04696
P 5.86365 -1.70606 -2.40187
C 2.87862 -0.44255 0.08307
H 3.28977 0.56268 0.08568
C 5.81129 0.19797 0.01516
C 4.91468 -2.98252 3.18889
H 3.88439 -2.61889 3.13646
H 4.97788 -3.95188 2.68878
H 5.20996 -3.08506 4.23826
C 7.71368 -2.38886 2.65662
H 7.84715 -3.36619 2.18513
H 8.43641 -1.68553 2.23231
H 7.88782 -2.47458 3.73440
C 5.85722 -0.24989 3.29362
H 4.84344 0.14605 3.18205
H 6.05611 -0.43561 4.35404
H 6.56883 0.49244 2.92114
C 7.53541 -2.28739 -2.84435
H 7.69709 -3.27758 -2.40984
H 7.63526 -2.34422 -3.93339
H 8.28752 -1.59699 -2.45095
C 4.70753 -2.85128 -3.22161
H 4.93305 -2.90371 -4.29184
H 4.80394 -3.84269 -2.77272
H 3.68299 -2.49364 -3.08458

C 5.65023 -0.11888 -3.27455
H 5.77596 -0.26182 -4.35263
H 4.64809 0.27393 -3.07860
H 6.38878 0.60696 -2.92276
C -1.43575 -0.49357 0.10072
C -0.69722 -1.67857 0.12757
C -0.71423 0.75111 0.08423
C 0.69730 -1.67853 0.12757
H -1.20829 -2.63524 0.14851
C 0.71418 0.75115 0.08424
C 1.43577 -0.49349 0.10070
H 1.20843 -2.63517 0.14856
Cl -5.82480 -4.03454 -0.06978
Cl 5.82473 -4.03442 -0.07030
N 1.39458 1.90667 0.06518
C 0.71996 3.04411 0.04828
C -0.72013 3.04406 0.04823
C 1.44778 4.30344 0.03015
C -1.44803 4.30335 0.02999
N -1.39469 1.90659 0.06514
C 0.71614 5.51871 0.01606
C 2.84247 4.33144 0.02726
C -2.84273 4.33126 0.02692
C -0.71647 5.51867 0.01597
C 1.41821 6.75347 0.00101
C 3.52635 5.54680 0.01201
C -3.52667 5.54658 0.01155
C -1.41862 6.75338 0.00083
C 0.67945 7.98004 -0.01266
C 2.82505 6.74157 -0.00041
H 4.61214 5.55405 0.01014
C -2.82545 6.74140 -0.00078
H -4.61246 5.55376 0.00954
C -0.67993 7.98000 -0.01276
H 1.23115 8.91620 -0.02334
H 3.35854 7.68847 -0.01179
H -3.35900 7.68827 -0.01225
H -1.23168 8.91613 -0.02350
H -3.38701 3.39289 0.03652
H 3.38680 3.39310 0.03692

**Atomic coordinates of neutral $Ru_2$-3 from its DFT/BLYP35 calculated structure**

| | | | |
|---|---|---|---|
| Ru | 7.92624 | -1.39999 | 0.01060 |
| P | 7.87699 | -1.54975 | 2.40514 |
| O | 8.24429 | 1.56824 | 0.08570 |
| C | 5.92692 | -1.26233 | -0.02961 |
| H | 5.52595 | -2.27866 | -0.05398 |
| P | 7.98954 | -1.44078 | -2.38764 |
| C | 5.06147 | -0.23360 | -0.02754 |

| | | | |
|---|---|---|---|
| H | 5.44424 | 0.78254 | -0.00355 |
| C | 8.09668 | 0.41835 | 0.05583 |
| C | 6.70104 | -2.79368 | 3.06872 |
| H | 5.68648 | -2.52402 | 2.77499 |
| H | 6.93698 | -3.77002 | 2.64815 |
| H | 6.75913 | -2.83964 | 4.15739 |
| C | 9.48395 | -2.03343 | 3.15625 |
| H | 9.79706 | -2.99173 | 2.74240 |
| H | 10.24247 | -1.28730 | 2.91621 |
| H | 9.39986 | -2.11867 | 4.24102 |
| C | 7.42019 | -0.02213 | 3.31760 |
| H | 6.43962 | 0.31788 | 2.98346 |
| H | 7.38807 | -0.20409 | 4.39277 |
| H | 8.14605 | 0.76515 | 3.11252 |
| C | 9.63289 | -1.88604 | -3.08158 |
| H | 9.93199 | -2.85954 | -2.69367 |
| H | 9.60046 | -1.92575 | -4.17174 |
| H | 10.37477 | -1.14772 | -2.77478 |
| C | 6.85303 | -2.65613 | -3.16344 |
| H | 6.96695 | -2.65392 | -4.24861 |
| H | 7.07054 | -3.64987 | -2.77498 |
| H | 5.82385 | -2.40114 | -2.91046 |
| C | 7.57055 | 0.12553 | -3.25147 |
| H | 7.58264 | -0.01003 | -4.33387 |
| H | 6.57716 | 0.45295 | -2.94373 |
| H | 8.28772 | 0.90174 | -2.98322 |
| C | 3.59231 | -0.31345 | -0.04842 |
| C | 2.86857 | -1.52342 | -0.07812 |
| C | 1.48952 | -1.53514 | -0.08738 |
| H | 3.39479 | -2.46841 | -0.09230 |
| C | 1.44865 | 0.86858 | -0.04318 |
| C | 0.73323 | -0.34755 | -0.06799 |
| H | 0.99187 | -2.49324 | -0.10854 |
| Cl | 8.33137 | -3.84957 | -0.03485 |
| C | 2.84851 | 0.86417 | -0.03390 |
| H | 3.35672 | 1.81784 | -0.01259 |
| C | -0.73323 | -0.34755 | -0.06799 |
| C | -1.48952 | -1.53514 | -0.08738 |
| C | -1.44865 | 0.86858 | -0.04318 |
| C | -2.86857 | -1.52342 | -0.07812 |
| H | -0.99187 | -2.49324 | -0.10853 |
| C | -2.84851 | 0.86417 | -0.03391 |
| H | -3.39479 | -2.46841 | -0.09230 |
| H | -3.35672 | 1.81784 | -0.01260 |
| C | -0.72500 | 2.14295 | -0.02525 |
| C | 0.72500 | 2.14295 | -0.02525 |
| C | -0.71722 | 4.43732 | 0.00745 |
| C | 0.71722 | 4.43732 | 0.00745 |
| C | -1.40800 | 5.65117 | 0.02439 |
| C | 1.40800 | 5.65117 | 0.02439 |
| H | -2.48996 | 5.63020 | 0.02404 |
| H | 2.48996 | 5.63020 | 0.02405 |
| N | 1.40491 | 3.26485 | -0.00909 |
| N | -1.40491 | 3.26485 | -0.00910 |
| C | -3.59231 | -0.31345 | -0.04842 |
| C | -5.06147 | -0.23360 | -0.02754 |

| | | | |
|---|---|---|---|
| C | -5.92692 | -1.26233 | -0.02961 |
| H | -5.44424 | 0.78254 | -0.00355 |
| Ru | -7.92624 | -1.39999 | 0.01061 |
| H | -5.52595 | -2.27866 | -0.05398 |
| P | -7.98956 | -1.44079 | -2.38763 |
| P | -7.87697 | -1.54975 | 2.40515 |
| C | -8.09668 | 0.41835 | 0.05585 |
| Cl | -8.33137 | -3.84957 | -0.03483 |
| C | -6.85306 | -2.65614 | -3.16344 |
| C | -9.63292 | -1.88603 | -3.08156 |
| C | -7.57057 | 0.12552 | -3.25147 |
| C | -9.48393 | -2.03344 | 3.15627 |
| C | -6.70101 | -2.79366 | 3.06873 |
| C | -7.42018 | -0.02212 | 3.31760 |
| O | -8.24429 | 1.56825 | 0.08562 |
| H | -5.82388 | -2.40116 | -2.91046 |
| H | -7.07057 | -3.64988 | -2.77497 |
| H | -6.96699 | -2.65393 | -4.24861 |
| H | -9.93202 | -2.85953 | -2.69365 |
| H | -10.37478 | -1.14771 | -2.77476 |
| H | -9.60049 | -1.92575 | -4.17172 |
| H | -6.57717 | 0.45294 | -2.94374 |
| H | -7.58266 | -0.01004 | -4.33387 |
| H | -8.28772 | 0.90174 | -2.98321 |
| H | -9.79703 | -2.99174 | 2.74242 |
| H | -9.39983 | -2.11867 | 4.24104 |
| H | -10.24245 | -1.28732 | 2.91623 |
| H | -6.75909 | -2.83962 | 4.15739 |
| H | -6.93694 | -3.77001 | 2.64816 |
| H | -5.68646 | -2.52400 | 2.77499 |
| H | -7.38805 | -0.20408 | 4.39277 |
| H | -6.43962 | 0.31790 | 2.98345 |
| H | -8.14605 | 0.76516 | 3.11252 |
| C | 0.72187 | 6.86563 | 0.04135 |
| C | -0.72187 | 6.86563 | 0.04134 |
| C | -1.40620 | 8.12107 | 0.05885 |
| C | -0.71328 | 9.29523 | 0.07524 |
| C | 0.71328 | 9.29523 | 0.07524 |
| C | 1.40620 | 8.12107 | 0.05886 |
| H | -2.48887 | 8.11832 | 0.05884 |
| H | -1.24264 | 10.23887 | 0.08837 |
| H | 1.24264 | 10.23887 | 0.08838 |
| H | 2.48887 | 8.11832 | 0.05886 |

**Atomic coordinates of [$Ru_2$-3]$^+$ from its DFT/BLYP35 calculated structure**

| | | | |
|---|---|---|---|
| Ru | 7.79510 | -1.40029 | -0.00709 |
| P | 8.00953 | -1.51120 | 2.40191 |
| O | 8.15865 | 1.58061 | -0.02151 |
| C | 5.87734 | -1.27881 | 0.01626 |
| H | 5.45387 | -2.28323 | 0.02787 |
| P | 7.94097 | -1.53398 | -2.42120 |
| C | 4.99718 | -0.21177 | 0.01672 |
| H | 5.39867 | 0.79482 | 0.00559 |
| C | 7.99422 | 0.44083 | -0.01635 |
| C | 6.85759 | -2.67689 | 3.21975 |
| H | 5.83271 | -2.33183 | 3.08250 |
| H | 6.96133 | -3.66150 | 2.76749 |
| H | 7.07533 | -2.73734 | 4.28696 |
| C | 9.68131 | -2.08933 | 2.88833 |
| H | 9.86417 | -3.06867 | 2.44799 |
| H | 10.43579 | -1.39255 | 2.52201 |
| H | 9.75890 | -2.16009 | 3.97431 |
| C | 7.78239 | 0.05760 | 3.32437 |
| H | 6.78267 | 0.45100 | 3.13910 |
| H | 7.90595 | -0.10854 | 4.39525 |
| H | 8.51351 | 0.79557 | 2.99449 |
| C | 9.59147 | -2.13145 | -2.95331 |
| H | 9.77869 | -3.10872 | -2.51024 |
| H | 9.63458 | -2.21199 | -4.04054 |
| H | 10.36327 | -1.43892 | -2.61639 |
| C | 6.75656 | -2.69752 | -3.19482 |
| H | 6.94178 | -2.76741 | -4.26762 |
| H | 6.86767 | -3.67952 | -2.73862 |
| H | 5.73855 | -2.34484 | -3.02925 |
| C | 7.69930 | 0.02869 | -3.35023 |
| H | 7.78579 | -0.14827 | -4.42301 |
| H | 6.71031 | 0.43420 | -3.13568 |
| H | 8.44848 | 0.76175 | -3.05144 |
| C | 3.57163 | -0.29738 | 0.02582 |
| C | 2.84239 | -1.52056 | 0.04316 |
| C | 1.47545 | -1.52271 | 0.04288 |
| H | 3.36554 | -2.46535 | 0.05722 |
| C | 1.43997 | 0.90694 | 0.01636 |
| C | 0.71763 | -0.32021 | 0.02439 |
| H | 0.97001 | -2.47511 | 0.05959 |
| Cl | 8.01676 | -3.84496 | 0.00036 |
| C | 2.82462 | 0.89903 | 0.01606 |
| H | 3.34116 | 1.84785 | 0.00714 |
| C | -0.72621 | -0.31915 | 0.01440 |
| C | -1.48478 | -1.51494 | 0.00535 |
| C | -1.44753 | 0.90536 | 0.01120 |
| C | -2.85684 | -1.50548 | -0.00001 |
| H | -0.98615 | -2.47170 | -0.00087 |
| C | -2.83781 | 0.89736 | 0.00684 |
| H | -3.38232 | -2.44970 | -0.00740 |
| H | -3.35111 | 1.84801 | 0.00597 |
| C | -0.72614 | 2.18259 | 0.01030 |
| C | 0.71763 | 2.18407 | 0.00919 |
| C | -0.71967 | 4.47408 | 0.00335 |

| | | | |
|---|---|---|---|
| C | 0.71651 | 4.47412 | 0.00082 |
| C | -1.41024 | 5.68799 | 0.00064 |
| C | 1.40836 | 5.68698 | -0.00467 |
| H | -2.49191 | 5.66744 | 0.00272 |
| H | 2.49005 | 5.66571 | -0.00643 |
| N | 1.40256 | 3.30113 | 0.00417 |
| N | -1.40769 | 3.30265 | 0.00783 |
| C | -3.58363 | -0.28941 | 0.00302 |
| C | 0.72172 | 6.90108 | -0.00768 |
| C | -0.72261 | 6.90145 | -0.00486 |
| C | -1.40708 | 8.15664 | -0.00796 |
| C | -0.71306 | 9.33012 | -0.01348 |
| C | 0.71347 | 9.32971 | -0.01629 |
| C | 1.40687 | 8.15581 | -0.01349 |
| H | -2.48953 | 8.15441 | -0.00588 |
| H | -1.24160 | 10.27411 | -0.01580 |
| H | 1.24252 | 10.27341 | -0.02069 |
| H | 2.48934 | 8.15313 | -0.01564 |
| C | -5.03644 | -0.20917 | 0.00101 |
| C | -5.89777 | -1.25426 | 0.00066 |
| H | -5.42834 | 0.80273 | 0.00106 |
| Ru | -7.87326 | -1.41088 | 0.00051 |
| H | -5.47942 | -2.26279 | 0.00113 |
| P | -7.91978 | -1.51423 | -2.40213 |
| P | -7.91920 | -1.51460 | 2.40320 |
| C | -8.07247 | 0.41132 | 0.00062 |
| Cl | -8.16535 | -3.86908 | 0.00040 |
| C | -6.71293 | -2.68144 | -3.14283 |
| C | -9.53694 | -2.07187 | -3.07311 |
| C | -7.59562 | 0.05246 | -3.30385 |
| C | -9.53609 | -2.07238 | 3.07473 |
| C | -6.71208 | -2.68188 | 3.14338 |
| C | -7.59473 | 0.05197 | 3.30503 |
| O | -8.23272 | 1.55776 | 0.00065 |
| H | -5.70039 | -2.35368 | -2.90664 |
| H | -6.86652 | -3.67280 | -2.71953 |
| H | -6.83251 | -2.72317 | -4.22647 |
| H | -9.77466 | -3.05360 | -2.66454 |
| H | -10.31990 | -1.37282 | -2.77685 |
| H | -9.50604 | -2.13078 | -4.16241 |
| H | -6.61960 | 0.44323 | -3.01492 |
| H | -7.60940 | -0.11044 | -4.38236 |
| H | -8.35370 | 0.79295 | -3.04770 |
| H | -9.77386 | -3.05413 | 2.66624 |
| H | -9.50478 | -2.13131 | 4.16402 |
| H | -10.31922 | -1.37341 | 2.77878 |
| H | -6.83149 | -2.72390 | 4.22703 |
| H | -6.86562 | -3.67314 | 2.71985 |
| H | -5.69960 | -2.35394 | 2.90713 |
| H | -7.60774 | -0.11119 | 4.38352 |
| H | -6.61897 | 0.44299 | 3.01553 |
| H | -8.35313 | 0.79236 | 3.04956 |

**Atomic coordinates of cis-$[Ru_2\text{-}3]^+$ from its DFT/BLYP35 calculated structure**

| | | | |
|---|---|---|---|
| Ru | -7.76802 | -0.72315 | -0.01044 |
| P | -7.97966 | -0.44966 | 2.38591 |
| O | -8.20768 | -3.68837 | 0.15690 |
| C | -5.85376 | -0.89189 | 0.02830 |
| H | -5.40453 | 0.10044 | -0.01770 |
| P | -7.91069 | -0.74337 | -2.42737 |
| C | -4.99999 | -1.97811 | 0.09152 |
| H | -5.42715 | -2.97344 | 0.13336 |
| C | -8.01296 | -2.55480 | 0.09418 |
| C | -6.82358 | 0.76679 | 3.12000 |
| H | -5.79940 | 0.41213 | 3.00350 |
| H | -6.92652 | 1.71787 | 2.60092 |
| H | -7.03830 | 0.90183 | 4.18105 |
| C | -9.64871 | 0.16492 | 2.83517 |
| H | -9.82808 | 1.11490 | 2.33327 |
| H | -10.40646 | -0.55065 | 2.51485 |
| H | -9.72488 | 0.30508 | 3.91442 |
| C | -7.75476 | -1.95245 | 3.41317 |
| H | -6.75617 | -2.35978 | 3.25379 |
| H | -7.87632 | -1.71371 | 4.47051 |
| H | -8.48791 | -2.70965 | 3.13542 |
| C | -9.56534 | -0.19047 | -2.99422 |
| H | -9.75721 | 0.81376 | -2.61844 |
| H | -9.61179 | -0.18485 | -4.08432 |
| H | -10.33222 | -0.86286 | -2.60841 |
| C | -6.73100 | 0.37278 | -3.27451 |
| H | -6.91935 | 0.37613 | -4.34898 |
| H | -6.84237 | 1.38068 | -2.87902 |
| H | -5.71161 | 0.03307 | -3.09077 |
| C | -7.66500 | -2.36034 | -3.25692 |
| H | -7.75333 | -2.24954 | -4.33837 |
| H | -6.67459 | -2.74901 | -3.01932 |
| H | -8.41188 | -3.07562 | -2.91280 |
| C | -3.57229 | -1.92767 | 0.09783 |
| C | -2.84305 | -3.14847 | 0.13767 |
| C | -1.47528 | -3.16117 | 0.13081 |
| H | -3.38761 | -4.08269 | 0.17148 |
| C | -1.44174 | -0.73248 | 0.06243 |
| C | -0.71826 | -1.96118 | 0.08588 |
| H | -0.97316 | -4.11494 | 0.16322 |
| Cl | -7.95416 | 1.71958 | -0.16212 |
| C | -2.82509 | -0.73057 | 0.06558 |
| H | -3.31993 | 0.22760 | 0.04410 |
| C | 0.72579 | -1.96115 | 0.06393 |
| C | 1.48197 | -3.15589 | 0.05983 |
| C | 1.44968 | -0.73576 | 0.04092 |
| C | 2.85539 | -3.13905 | 0.04088 |
| H | 0.98525 | -4.11364 | 0.06718 |
| C | 2.83777 | -0.73677 | 0.02387 |
| H | 3.39741 | -4.07594 | 0.03743 |
| H | 3.33451 | 0.22066 | 0.00714 |
| C | 0.72699 | 0.54229 | 0.03158 |
| C | -0.71654 | 0.54449 | 0.03696 |
| C | 0.72169 | 2.83428 | 0.00243 |

| | | | |
|---|---|---|---|
| C | -0.71433 | 2.83511 | 0.00556 |
| C | 1.41311 | 4.04734 | -0.01413 |
| C | -1.40577 | 4.04791 | -0.00859 |
| H | 2.49486 | 4.02651 | -0.01582 |
| H | -2.48757 | 4.02757 | -0.00581 |
| N | -1.40014 | 1.66190 | 0.02339 |
| N | 1.40833 | 1.66190 | 0.01520 |
| C | 3.58303 | -1.92572 | 0.02517 |
| C | -0.71829 | 5.26160 | -0.02550 |
| C | 0.72594 | 5.26115 | -0.02814 |
| C | 1.41119 | 6.51573 | -0.04519 |
| C | 0.71777 | 7.68955 | -0.05863 |
| C | -0.70871 | 7.68996 | -0.05609 |
| C | -1.40282 | 6.51653 | -0.04013 |
| H | 2.49363 | 6.51271 | -0.04720 |
| H | 1.24688 | 8.63314 | -0.07141 |
| H | -1.23727 | 8.63388 | -0.06702 |
| H | -2.48529 | 6.51433 | -0.03831 |
| C | 5.03727 | -1.97652 | 0.00863 |
| C | 5.87671 | -0.91340 | 0.00564 |
| H | 5.45103 | -2.97978 | 0.00051 |
| Ru | 7.84709 | -0.71310 | -0.01180 |
| H | 5.43510 | 0.08515 | 0.01662 |
| P | 7.86745 | -0.58297 | -2.41346 |
| P | 7.91198 | -0.63303 | 2.39144 |
| C | 8.08605 | -2.53014 | -0.03356 |
| Cl | 8.09903 | 1.74869 | 0.01165 |
| C | 6.63896 | 0.57769 | -3.12824 |
| C | 9.47070 | 0.00065 | -3.09566 |
| C | 7.55180 | -2.14441 | -3.32716 |
| C | 9.52837 | -0.06609 | 3.05621 |
| C | 6.69884 | 0.51451 | 3.15201 |
| C | 7.61012 | -2.21214 | 3.27905 |
| O | 8.27202 | -3.67272 | -0.04781 |
| H | 5.63282 | 0.23487 | -2.88618 |
| H | 6.78429 | 1.56557 | -2.69413 |
| H | 6.74742 | 0.63428 | -4.21244 |
| H | 9.70054 | 0.98151 | -2.68056 |
| H | 10.26533 | -0.69124 | -2.81408 |
| H | 9.42775 | 0.06926 | -4.18397 |
| H | 6.58408 | -2.54965 | -3.03033 |
| H | 7.55050 | -1.97090 | -4.40407 |
| H | 8.32180 | -2.87806 | -3.08739 |
| H | 9.75346 | 0.92207 | 2.65605 |
| H | 9.50457 | -0.01848 | 4.14624 |
| H | 10.31603 | -0.75432 | 2.74740 |
| H | 6.82611 | 0.54715 | 4.23509 |
| H | 6.83903 | 1.51132 | 2.73699 |
| H | 5.68788 | 0.17906 | 2.91993 |
| H | 7.63075 | -2.06051 | 4.35911 |
| H | 6.63554 | -2.60886 | 2.99335 |
| H | 8.37317 | -2.94258 | 3.00922 |

**Atomic coordinates of trans-[$Ru_2$-3]$^+$ from its DFT/BLYP35 calculated structure**

| | | | |
|---|---|---|---|
| Ru | 8.06521 | -0.75057 | -0.01692 |
| P | 8.32178 | -0.77579 | 2.38960 |
| O | 7.87497 | 2.24497 | -0.08374 |
| C | 6.15667 | -0.98489 | 0.03244 |
| H | 5.92651 | -2.05023 | 0.05908 |
| P | 8.19675 | -0.89809 | -2.43016 |
| C | 5.09502 | -0.09910 | 0.03288 |
| H | 5.30436 | 0.96390 | 0.00955 |
| C | 7.92166 | 1.09440 | -0.05784 |
| C | 7.42582 | -2.13080 | 3.23541 |
| H | 6.35234 | -1.98930 | 3.10953 |
| H | 7.71023 | -3.08243 | 2.79020 |
| H | 7.66462 | -2.13634 | 4.29968 |
| C | 10.07715 | -1.00610 | 2.87032 |
| H | 10.44326 | -1.94426 | 2.45522 |
| H | 10.68038 | -0.18793 | 2.47567 |
| H | 10.17350 | -1.02761 | 3.95688 |
| C | 7.79797 | 0.73179 | 3.29314 |
| H | 6.74102 | 0.92323 | 3.10670 |
| H | 7.95249 | 0.60489 | 4.36548 |
| H | 8.37334 | 1.59246 | 2.95201 |
| C | 9.92441 | -1.16586 | -2.98511 |
| H | 10.30766 | -2.08176 | -2.53717 |
| H | 9.96687 | -1.24759 | -4.07219 |
| H | 10.54902 | -0.33073 | -2.66719 |
| C | 7.25096 | -2.28463 | -3.16389 |
| H | 7.43409 | -2.34198 | -4.23780 |
| H | 7.55253 | -3.21688 | -2.68984 |
| H | 6.18607 | -2.12867 | -2.99054 |
| C | 7.63638 | 0.56797 | -3.37891 |
| H | 7.74066 | 0.39055 | -4.45009 |
| H | 6.58978 | 0.77321 | -3.15307 |
| H | 8.22922 | 1.44080 | -3.10541 |
| C | 3.70930 | -0.44437 | 0.05366 |
| C | 3.21600 | -1.78013 | 0.08148 |
| C | 1.87267 | -2.03227 | 0.08843 |
| H | 3.90305 | -2.61338 | 0.09609 |
| C | 1.39312 | 0.34970 | 0.04756 |
| C | 0.90731 | -0.98881 | 0.06883 |
| H | 1.55041 | -3.06116 | 0.10819 |
| Cl | 8.74379 | -3.10957 | 0.02327 |
| C | 2.75568 | 0.59500 | 0.04032 |
| H | 3.08992 | 1.62219 | 0.02175 |
| C | -0.51189 | -1.25280 | 0.06704 |
| C | -1.03751 | -2.56550 | 0.08289 |
| C | -1.44752 | -0.17977 | 0.04519 |
| C | -2.39080 | -2.79963 | 0.07518 |
| H | -0.37453 | -3.41638 | 0.10045 |
| C | -2.81200 | -0.43423 | 0.03772 |
| H | -2.75278 | -3.81973 | 0.08681 |
| H | -3.47552 | 0.41609 | 0.02026 |
| C | -0.96983 | 1.20850 | 0.02977 |
| C | 0.44938 | 1.47344 | 0.03136 |
| C | -1.38160 | 3.46306 | 0.00124 |

| | | | |
|---|---|---|---|
| C | 0.03045 | 3.72494 | 0.00348 |
| C | -2.28196 | 4.53036 | -0.01445 |
| C | 0.48971 | 5.04343 | -0.00964 |
| H | -3.34198 | 4.31357 | -0.01602 |
| H | 1.55710 | 5.21989 | -0.00751 |
| N | 0.91878 | 2.69665 | 0.01845 |
| N | -1.84372 | 2.18540 | 0.01471 |
| C | -3.32773 | -1.73939 | 0.05097 |
| C | -0.40676 | 6.11193 | -0.02494 |
| C | -1.82684 | 5.84884 | -0.02756 |
| C | -2.72869 | 6.95801 | -0.04342 |
| C | -2.26029 | 8.23835 | -0.05564 |
| C | -0.85762 | 8.49815 | -0.05292 |
| C | 0.03821 | 7.47047 | -0.03809 |
| H | -3.79251 | 6.75826 | -0.04541 |
| H | -2.95220 | 9.06998 | -0.06753 |
| H | -0.50943 | 9.52246 | -0.06270 |
| H | 1.10309 | 7.66503 | -0.03593 |
| C | -4.74740 | -2.05575 | 0.03991 |
| C | -5.76961 | -1.16664 | 0.01539 |
| H | -4.96951 | -3.11783 | 0.05166 |
| Ru | -7.74166 | -1.34099 | -0.00624 |
| H | -5.52214 | -0.10309 | 0.00449 |
| P | -7.83719 | -1.22712 | 2.39362 |
| P | -7.78320 | -1.26640 | -2.40986 |
| C | -7.63520 | -3.17073 | 0.00951 |
| Cl | -8.46064 | 1.02675 | -0.03337 |
| C | -6.89300 | 0.16046 | 3.13498 |
| C | -9.54390 | -0.99218 | 3.03191 |
| C | -7.22553 | -2.69204 | 3.31660 |
| C | -9.47606 | -1.05100 | -3.09090 |
| C | -6.82849 | 0.11327 | -3.15254 |
| C | -7.14333 | -2.74289 | -3.29449 |
| O | -7.60536 | -4.32801 | 0.01896 |
| H | -5.83282 | 0.03504 | 2.91410 |
| H | -7.23145 | 1.09821 | 2.69737 |
| H | -7.03252 | 0.18855 | 4.21674 |
| H | -9.96054 | -0.07785 | 2.61038 |
| H | -10.16932 | -1.83215 | 2.72746 |
| H | -9.54723 | -0.92284 | 4.12087 |
| H | -6.18822 | -2.88613 | 3.04233 |
| H | -7.28486 | -2.52582 | 4.39315 |
| H | -7.82048 | -3.56886 | 3.06006 |
| H | -9.90726 | -0.13248 | -2.69377 |
| H | -9.45512 | -0.99881 | -4.18065 |
| H | -10.10379 | -1.88963 | -2.78736 |
| H | -6.94243 | 0.12187 | -4.23763 |
| H | -7.18140 | 1.05673 | -2.73942 |
| H | -5.77323 | -0.00298 | -2.90466 |
| H | -7.17672 | -2.59373 | -4.37463 |
| H | -6.11235 | -2.92810 | -2.99173 |
| H | -7.74099 | -3.61817 | -3.03912 |

### Atomic coordinates of $Ru_2$-7 from its DFT/BLYP35 calculated structure

| | | | |
|---|---|---|---|
| Ru | -5.73784 | -1.67039 | -0.04568 |
| P | -5.58235 | -2.61982 | -2.24377 |
| O | -6.10079 | 1.08626 | -1.13436 |
| C | -3.74496 | -1.45836 | 0.02167 |
| H | -3.31934 | -2.40219 | 0.37321 |
| P | -5.89918 | -0.89522 | 2.21862 |
| C | -2.91061 | -0.43754 | -0.24449 |
| H | -3.31324 | 0.51987 | -0.54700 |
| C | -5.93625 | 0.01858 | -0.71164 |
| C | -4.39058 | -4.00808 | -2.39682 |
| H | -3.38581 | -3.64817 | -2.17466 |
| H | -4.64919 | -4.78301 | -1.67685 |
| H | -4.40717 | -4.42396 | -3.40547 |
| C | -7.15903 | -3.33837 | -2.85998 |
| H | -7.49819 | -4.10651 | -2.16520 |
| H | -7.92141 | -2.56029 | -2.91560 |
| H | -7.02855 | -3.77944 | -3.84972 |
| C | -5.07258 | -1.48671 | -3.59700 |
| H | -4.10793 | -1.04248 | -3.35050 |
| H | -4.98914 | -2.02121 | -4.54439 |
| H | -5.80292 | -0.68448 | -3.70557 |
| C | -7.56796 | -1.09863 | 2.96401 |
| H | -7.85112 | -2.15029 | 2.92458 |
| H | -7.57889 | -0.76099 | 4.00179 |
| H | -8.29816 | -0.52180 | 2.39510 |
| C | -4.79228 | -1.75938 | 3.40116 |
| H | -4.94599 | -1.39067 | 4.41661 |
| H | -4.99463 | -2.82873 | 3.36505 |
| H | -3.75486 | -1.59143 | 3.11165 |
| C | -5.51205 | 0.87696 | 2.50931 |
| H | -5.56152 | 1.11957 | 3.57184 |
| H | -4.50895 | 1.09111 | 2.13983 |
| H | -6.21983 | 1.50586 | 1.96858 |
| Ru | 5.73784 | -1.67040 | 0.04563 |
| P | 5.58203 | -2.62022 | 2.24352 |
| O | 6.10096 | 1.08594 | 1.13496 |
| C | 3.74498 | -1.45826 | -0.02180 |
| H | 3.31936 | -2.40198 | -0.37364 |
| P | 5.89932 | -0.89480 | -2.21853 |
| C | 2.91062 | -0.43753 | 0.24464 |
| H | 3.31324 | 0.51980 | 0.54742 |
| C | 5.93626 | 0.01842 | 0.71191 |
| C | 4.39050 | -4.00877 | 2.39595 |
| H | 3.38572 | -3.64904 | 2.17353 |
| H | 4.64950 | -4.78352 | 1.67593 |
| H | 4.40685 | -4.42482 | 3.40453 |
| C | 7.15864 | -3.33855 | 2.86013 |
| H | 7.49818 | -4.10651 | 2.16533 |
| H | 7.92085 | -2.56033 | 2.91611 |
| H | 7.02793 | -3.77981 | 3.84975 |
| C | 5.07155 | -1.48744 | 3.59674 |
| H | 4.10690 | -1.04337 | 3.34995 |
| H | 4.98788 | -2.02210 | 4.54402 |
| H | 5.80168 | -0.68507 | 3.70571 |

| | | | |
|---|---|---|---|
| C | 7.56818 | -1.09772 | -2.96389 |
| H | 7.85163 | -2.14930 | -2.92450 |
| H | 7.57903 | -0.76002 | -4.00164 |
| H | 8.29820 | -0.52071 | -2.39494 |
| C | 4.79267 | -1.75902 | -3.40127 |
| H | 4.94626 | -1.39000 | -4.41663 |
| H | 4.99537 | -2.82831 | -3.36543 |
| H | 3.75521 | -1.59146 | -3.11172 |
| C | 5.51183 | 0.87735 | -2.50891 |
| H | 5.56131 | 1.12017 | -3.57139 |
| H | 4.50866 | 1.09122 | -2.13944 |
| H | 6.21945 | 1.50629 | -1.96802 |
| C | -1.44676 | -0.46711 | -0.12425 |
| C | -0.69919 | -1.63016 | -0.06718 |
| C | -0.71416 | 0.76653 | -0.06293 |
| C | 0.69922 | -1.63015 | 0.06726 |
| H | -1.20243 | -2.58609 | -0.12868 |
| C | 0.71416 | 0.76653 | 0.06305 |
| C | 1.44677 | -0.46709 | 0.12435 |
| H | 1.20247 | -2.58608 | 0.12876 |
| Cl | -6.11071 | -3.97072 | 0.81627 |
| Cl | 6.11147 | -3.97048 | -0.81666 |
| N | 1.38311 | 1.93428 | 0.12305 |
| C | 0.71253 | 3.06921 | 0.06608 |
| C | -0.71255 | 3.06921 | -0.06593 |
| C | 1.44182 | 4.33701 | 0.13975 |
| C | -1.44185 | 4.33700 | -0.13961 |
| N | -1.38311 | 1.93427 | -0.12292 |
| C | 0.71542 | 5.55285 | 0.07008 |
| C | 2.82658 | 4.36877 | 0.27767 |
| C | -2.82661 | 4.36874 | -0.27754 |
| C | -0.71547 | 5.55284 | -0.06993 |
| C | 1.41414 | 6.78793 | 0.13972 |
| C | 3.50681 | 5.58463 | 0.34660 |
| C | -3.50685 | 5.58460 | -0.34647 |
| C | -1.41419 | 6.78792 | -0.13958 |
| C | 0.67395 | 8.01627 | 0.06687 |
| C | 2.81160 | 6.77934 | 0.27806 |
| H | 4.58342 | 5.59040 | 0.45462 |
| C | -2.81165 | 6.77932 | -0.27792 |
| H | -4.58346 | 5.59037 | -0.45450 |
| C | -0.67401 | 8.01626 | -0.06672 |
| H | 1.22135 | 8.94882 | 0.12116 |
| H | 3.33993 | 7.72295 | 0.33129 |
| H | -3.33999 | 7.72292 | -0.33115 |
| H | -1.22142 | 8.94881 | -0.12101 |
| H | -3.36685 | 3.43524 | -0.33204 |
| H | 3.36683 | 3.43527 | 0.33215 |

**Atomic coordinates of $[Ru_2\text{-}7]^+$ from its DFT/BLYP35 calculated structure**

| | | | |
|---|---|---|---|
| Ru | -5.64566 | -1.66941 | 0.00003 |
| P | -5.76185 | -1.78700 | -2.40881 |
| O | -6.07804 | 1.29673 | 0.00002 |
| C | -3.71226 | -1.48148 | -0.00003 |
| H | -3.26222 | -2.47505 | -0.00003 |
| P | -5.76171 | -1.78699 | 2.40888 |
| C | -2.87432 | -0.38962 | -0.00005 |
| H | -3.30154 | 0.60276 | -0.00004 |
| C | -5.89055 | 0.15769 | 0.00003 |
| C | -4.55585 | -2.92985 | -3.18346 |
| H | -3.54341 | -2.57014 | -2.99898 |
| H | -4.66287 | -3.91849 | -2.74049 |
| H | -4.72429 | -2.98949 | -4.25971 |
| C | -7.39434 | -2.39508 | -2.98737 |
| H | -7.58294 | -3.37839 | -2.55798 |
| H | -8.18026 | -1.71364 | -2.66013 |
| H | -7.41400 | -2.46536 | -4.07605 |
| C | -5.52013 | -0.21665 | -3.32730 |
| H | -4.54059 | 0.19922 | -3.08982 |
| H | -5.58322 | -0.38673 | -4.40288 |
| H | -6.28293 | 0.50638 | -3.03830 |
| C | -7.39413 | -2.39514 | 2.98753 |
| H | -7.58271 | -3.37847 | 2.55817 |
| H | -7.41373 | -2.46541 | 4.07622 |
| H | -8.18010 | -1.71375 | 2.66033 |
| C | -4.55560 | -2.92977 | 3.18346 |
| H | -4.72399 | -2.98941 | 4.25973 |
| H | -4.66261 | -3.91843 | 2.74051 |
| H | -3.54319 | -2.57001 | 2.99892 |
| C | -5.52001 | -0.21662 | 3.32734 |
| H | -5.58302 | -0.38670 | 4.40292 |
| H | -4.54050 | 0.19929 | 3.08979 |
| H | -6.28286 | 0.50637 | 3.03838 |
| Ru | 5.64589 | -1.66941 | 0.00001 |
| P | 5.76139 | -1.78672 | 2.40889 |
| O | 6.07841 | 1.29666 | -0.00018 |
| C | 3.71243 | -1.48131 | -0.00003 |
| H | 3.26241 | -2.47492 | -0.00001 |
| P | 5.76148 | -1.78708 | -2.40884 |
| C | 2.87445 | -0.38953 | -0.00006 |
| H | 3.30164 | 0.60287 | -0.00006 |
| C | 5.89075 | 0.15763 | -0.00012 |
| C | 4.55536 | -2.92997 | 3.18292 |
| H | 3.54288 | -2.57071 | 2.99780 |
| H | 4.66306 | -3.91862 | 2.74013 |
| H | 4.72318 | -2.98945 | 4.25928 |
| C | 7.39368 | -2.39401 | 2.98881 |
| H | 7.58308 | -3.37730 | 2.55973 |
| H | 8.17957 | -1.71227 | 2.66213 |
| H | 7.41245 | -2.46410 | 4.07753 |
| C | 5.51842 | -0.21632 | 3.32698 |
| H | 4.53894 | 0.19920 | 3.08861 |
| H | 5.58068 | -0.38624 | 4.40262 |
| H | 6.28123 | 0.50690 | 3.03849 |

| | | | |
|---|---|---|---|
| C | 7.39384 | -2.39432 | -2.98861 |
| H | 7.58331 | -3.37752 | -2.55937 |
| H | 7.41265 | -2.46458 | -4.07732 |
| H | 8.17966 | -1.71247 | -2.66201 |
| C | 4.55558 | -2.93056 | -3.18274 |
| H | 4.72344 | -2.99019 | -4.25909 |
| H | 4.66335 | -3.91913 | -2.73979 |
| H | 3.54305 | -2.57137 | -2.99770 |
| C | 5.51841 | -0.21685 | -3.32719 |
| H | 5.58073 | -0.38693 | -4.40281 |
| H | 4.53888 | 0.19863 | -3.08893 |
| H | 6.28114 | 0.50649 | -3.03878 |
| C | -1.45162 | -0.42718 | -0.00006 |
| C | -0.68794 | -1.61519 | -0.00006 |
| C | -0.71059 | 0.82099 | -0.00007 |
| C | 0.68810 | -1.61516 | -0.00006 |
| H | -1.19530 | -2.56875 | -0.00006 |
| C | 0.71066 | 0.82101 | -0.00007 |
| C | 1.45172 | -0.42714 | -0.00007 |
| H | 1.19550 | -2.56870 | -0.00006 |
| Cl | -5.82500 | -4.12657 | 0.00003 |
| Cl | 5.82730 | -4.12641 | 0.00019 |
| N | 1.38572 | 1.97818 | -0.00006 |
| C | 0.71566 | 3.11900 | -0.00006 |
| C | -0.71565 | 3.11898 | -0.00006 |
| C | 1.44954 | 4.38144 | -0.00005 |
| C | -1.44956 | 4.38140 | -0.00005 |
| N | -1.38569 | 1.97814 | -0.00006 |
| C | 0.71812 | 5.59696 | -0.00004 |
| C | 2.84182 | 4.41140 | -0.00004 |
| C | -2.84185 | 4.41134 | -0.00005 |
| C | -0.71818 | 5.59694 | -0.00004 |
| C | 1.42004 | 6.83182 | -0.00003 |
| C | 3.52421 | 5.62713 | -0.00003 |
| C | -3.52427 | 5.62704 | -0.00004 |
| C | -1.42013 | 6.83179 | -0.00003 |
| C | 0.67716 | 8.06051 | -0.00002 |
| C | 2.82416 | 6.82146 | -0.00003 |
| H | 4.60593 | 5.63410 | -0.00003 |
| C | -2.82424 | 6.82139 | -0.00003 |
| H | -4.60598 | 5.63399 | -0.00004 |
| C | -0.67727 | 8.06049 | -0.00003 |
| H | 1.22724 | 8.99276 | -0.00002 |
| H | 3.35590 | 7.76443 | -0.00002 |
| H | -3.35600 | 7.76435 | -0.00003 |
| H | -1.22738 | 8.99272 | -0.00002 |
| H | -3.38724 | 3.47939 | -0.00005 |
| H | 3.38722 | 3.47946 | -0.00005 |

**Atomic coordinates of cis-$[Ru_2\text{-}7]^+$ from its DFT/BLYP35 calculated structure**

| | | | |
|---|---|---|---|
| Ru | -5.80744 | -1.16552 | -0.00008 |
| P | -5.93785 | -1.06462 | 2.40739 |
| O | -5.81918 | -4.16057 | -0.00017 |
| C | -3.87036 | -1.05354 | -0.00010 |
| H | -3.55855 | -0.01492 | -0.00025 |
| P | -5.93803 | -1.06442 | -2.40755 |
| C | -2.87415 | -2.00550 | 0.00013 |
| H | -3.17768 | -3.04663 | 0.00027 |
| C | -5.78624 | -3.00596 | -0.00014 |
| C | -4.94332 | 0.27304 | 3.17000 |
| H | -3.88556 | 0.09205 | 2.97808 |
| H | -5.22170 | 1.22615 | 2.72388 |
| H | -5.11200 | 0.30843 | 4.24724 |
| C | -7.64867 | -0.75015 | 2.99309 |
| H | -8.00747 | 0.18521 | 2.56518 |
| H | -8.30443 | -1.55833 | 2.66783 |
| H | -7.67673 | -0.68540 | 4.08191 |
| C | -5.42179 | -2.56499 | 3.32961 |
| H | -4.38614 | -2.80495 | 3.08749 |
| H | -5.50749 | -2.40334 | 4.40502 |
| H | -6.04876 | -3.41094 | 3.04767 |
| C | -7.64885 | -0.74984 | -2.99323 |
| H | -8.00760 | 0.18552 | -2.56530 |
| H | -7.67687 | -0.68505 | -4.08206 |
| H | -8.30466 | -1.55800 | -2.66803 |
| C | -4.94350 | 0.27330 | -3.17006 |
| H | -5.11232 | 0.30890 | -4.24727 |
| H | -5.22176 | 1.22634 | -2.72372 |
| H | -3.88573 | 0.09220 | -2.97831 |
| C | -5.42201 | -2.56473 | -3.32990 |
| H | -5.50757 | -2.40295 | -4.40530 |
| H | -4.38641 | -2.80480 | -3.08770 |
| H | -6.04908 | -3.41066 | -3.04812 |
| Ru | 5.80744 | -1.16552 | -0.00007 |
| P | 5.93802 | -1.06437 | -2.40754 |
| O | 5.81920 | -4.16057 | -0.00025 |
| C | 3.87036 | -1.05354 | -0.00009 |
| H | 3.55855 | -0.01492 | -0.00022 |
| P | 5.93785 | -1.06467 | 2.40740 |
| C | 2.87415 | -2.00550 | 0.00013 |
| H | 3.17768 | -3.04663 | 0.00025 |
| C | 5.78623 | -3.00596 | -0.00016 |
| C | 4.94346 | 0.27335 | -3.17002 |
| H | 3.88570 | 0.09223 | -2.97825 |
| H | 5.22171 | 1.22639 | -2.72366 |
| H | 5.11226 | 0.30897 | -4.24723 |
| C | 7.64883 | -0.74975 | -2.99323 |
| H | 8.00757 | 0.18562 | -2.56529 |
| H | 8.30466 | -1.55790 | -2.66805 |
| H | 7.67684 | -0.68493 | -4.08205 |
| C | 5.42203 | -2.56467 | -3.32992 |
| H | 4.38643 | -2.80476 | -3.08772 |
| H | 5.50759 | -2.40287 | -4.40532 |
| H | 6.04911 | -3.41059 | -3.04815 |

| | | | |
|---|---|---|---|
| C | 7.64868 | -0.75019 | 2.99309 |
| H | 8.00746 | 0.18519 | 2.56521 |
| H | 7.67674 | -0.68547 | 4.08192 |
| H | 8.30445 | -1.55836 | 2.66781 |
| C | 4.94331 | 0.27295 | 3.17005 |
| H | 5.11200 | 0.30832 | 4.24729 |
| H | 5.22167 | 1.22608 | 2.72395 |
| H | 3.88556 | 0.09195 | 2.97813 |
| C | 5.42182 | -2.56508 | 3.32959 |
| H | 5.50752 | -2.40345 | 4.40500 |
| H | 4.38617 | -2.80504 | 3.08747 |
| H | 6.04879 | -3.41101 | 3.04762 |
| C | -1.45804 | -1.83883 | 0.00017 |
| C | -0.68707 | -3.02120 | 0.00025 |
| C | -0.71519 | -0.58721 | 0.00015 |
| C | 0.68707 | -3.02120 | 0.00025 |
| H | -1.20768 | -3.96952 | 0.00029 |
| C | 0.71519 | -0.58721 | 0.00014 |
| C | 1.45804 | -1.83883 | 0.00016 |
| H | 1.20767 | -3.96952 | 0.00029 |
| Cl | -6.41835 | 1.22456 | 0.00004 |
| Cl | 6.41835 | 1.22456 | 0.00010 |
| N | 1.37968 | 0.57526 | 0.00014 |
| C | 0.71379 | 1.72065 | 0.00015 |
| C | -0.71379 | 1.72065 | 0.00017 |
| C | 1.44977 | 2.98269 | 0.00013 |
| C | -1.44976 | 2.98269 | 0.00018 |
| N | -1.37967 | 0.57526 | 0.00017 |
| C | 0.71800 | 4.19869 | 0.00009 |
| C | 2.84221 | 3.01762 | 0.00014 |
| C | -2.84221 | 3.01763 | 0.00025 |
| C | -0.71799 | 4.19869 | 0.00012 |
| C | 1.41937 | 5.43448 | 0.00004 |
| C | 3.52386 | 4.23344 | 0.00009 |
| C | -3.52386 | 4.23344 | 0.00023 |
| C | -1.41937 | 5.43448 | 0.00010 |
| C | 0.67706 | 6.66324 | 0.00001 |
| C | 2.82324 | 5.42688 | 0.00004 |
| H | 4.60551 | 4.23544 | 0.00010 |
| C | -2.82323 | 5.42688 | 0.00015 |
| H | -4.60551 | 4.23545 | 0.00028 |
| C | -0.67705 | 6.66324 | 0.00003 |
| H | 1.22771 | 7.59512 | -0.00004 |
| H | 3.35334 | 6.37078 | 0.00000 |
| H | -3.35333 | 6.37079 | 0.00014 |
| H | -1.22770 | 7.59513 | 0.00001 |
| H | -3.40155 | 2.09554 | 0.00032 |
| H | 3.40156 | 2.09554 | 0.00019 |

**Atomic coordinates of trans-[$Ru_2$-7]$^+$ from its DFT/BLYP35 calculated structure**

| | | | |
|---|---|---|---|
| Ru | 5.47220 | -1.84512 | 0.00019 |
| P | 5.62962 | -1.78448 | -2.40753 |
| O | 4.75204 | -4.75262 | 0.00173 |
| C | 3.62109 | -1.26672 | -0.00005 |
| H | 3.56996 | -0.18314 | -0.00056 |
| P | 5.62957 | -1.78189 | 2.40790 |
| C | 2.42353 | -1.95023 | 0.00033 |
| H | 2.46501 | -3.03399 | 0.00074 |
| C | 5.00352 | -3.62543 | 0.00109 |
| C | 4.98492 | -0.25202 | -3.17920 |
| H | 3.91391 | -0.17412 | -2.99171 |
| H | 5.48133 | 0.60899 | -2.73502 |
| H | 5.16085 | -0.26276 | -4.25587 |
| C | 7.36864 | -1.88703 | -2.98635 |
| H | 7.93807 | -1.06248 | -2.55912 |
| H | 7.81312 | -2.82700 | -2.65756 |
| H | 7.41442 | -1.83319 | -4.07518 |
| C | 4.77788 | -3.12492 | -3.32697 |
| H | 3.71262 | -3.11014 | -3.09496 |
| H | 4.90923 | -2.99616 | -4.40211 |
| H | 5.18330 | -4.09363 | -3.03451 |
| C | 7.36857 | -1.88302 | 2.98703 |
| H | 7.93762 | -1.05860 | 2.55901 |
| H | 7.41420 | -1.82805 | 4.07581 |
| H | 7.81356 | -2.82308 | 2.65922 |
| C | 4.98416 | -0.24879 | 3.17777 |
| H | 5.16005 | -0.25816 | 4.25446 |
| H | 5.48023 | 0.61190 | 2.73259 |
| H | 3.91313 | -0.17156 | 2.99014 |
| C | 4.77828 | -3.12167 | 3.32873 |
| H | 4.90958 | -2.99180 | 4.40374 |
| H | 3.71302 | -3.10750 | 3.09669 |
| H | 5.18403 | -4.09053 | 3.03726 |
| Ru | -6.10168 | -0.91375 | 0.00002 |
| P | -6.23947 | -1.00435 | -2.40810 |
| O | -5.80115 | 2.06872 | -0.00010 |
| C | -4.17925 | -1.20245 | 0.00013 |
| H | -3.98506 | -2.27586 | 0.00026 |
| P | -6.23974 | -1.00412 | 2.40815 |
| C | -3.10050 | -0.34909 | 0.00005 |
| H | -3.27116 | 0.71751 | -0.00008 |
| C | -5.89574 | 0.91810 | -0.00006 |
| C | -5.32564 | -2.39665 | -3.17445 |
| H | -4.25808 | -2.27390 | -2.99065 |
| H | -5.65392 | -3.33208 | -2.72460 |
| H | -5.50214 | -2.42470 | -4.25064 |
| C | -7.96719 | -1.22004 | -2.98973 |
| H | -8.37825 | -2.13328 | -2.56108 |
| H | -8.57520 | -0.37564 | -2.66363 |
| H | -8.00149 | -1.28262 | -4.07842 |
| C | -5.64057 | 0.46402 | -3.33184 |
| H | -4.59259 | 0.64555 | -3.09199 |
| H | -5.73751 | 0.30689 | -4.40700 |
| H | -6.21812 | 1.34441 | -3.04942 |

| | | | |
|---|---|---|---|
| C | -7.96751 | -1.21985 | 2.98965 |
| H | -8.37852 | -2.13311 | 2.56098 |
| H | -8.00189 | -1.28242 | 4.07834 |
| H | -8.57551 | -0.37547 | 2.66349 |
| C | -5.32591 | -2.39630 | 3.17470 |
| H | -5.50255 | -2.42430 | 4.25088 |
| H | -5.65404 | -3.33179 | 2.72488 |
| H | -4.25834 | -2.27347 | 2.99104 |
| C | -5.64101 | 0.46436 | 3.33181 |
| H | -5.73793 | 0.30728 | 4.40698 |
| H | -4.59305 | 0.64599 | 3.09195 |
| H | -6.21865 | 1.34468 | 3.04934 |
| C | 1.09327 | -1.43996 | 0.00022 |
| C | 0.05150 | -2.39767 | 0.00041 |
| C | 0.68451 | -0.04496 | 0.00002 |
| C | -1.28505 | -2.07425 | 0.00033 |
| H | 0.32903 | -3.44338 | 0.00059 |
| C | -0.70154 | 0.28904 | 0.00001 |
| C | -1.73044 | -0.73779 | 0.00012 |
| H | -2.00672 | -2.87780 | 0.00044 |
| Cl | 6.63444 | 0.33003 | -0.00090 |
| Cl | -6.86944 | -3.25560 | 0.00007 |
| N | -1.08737 | 1.57035 | -0.00012 |
| C | -0.17561 | 2.52809 | -0.00019 |
| C | 1.21530 | 2.19904 | -0.00020 |
| C | -0.60591 | 3.92302 | -0.00028 |
| C | 2.21734 | 3.26243 | -0.00030 |
| N | 1.60284 | 0.93159 | -0.00010 |
| C | 0.38149 | 4.94057 | -0.00038 |
| C | -1.95557 | 4.26638 | -0.00026 |
| C | 3.58154 | 2.98127 | -0.00030 |
| C | 1.77985 | 4.61317 | -0.00040 |
| C | -0.02289 | 6.30228 | -0.00047 |
| C | -2.34515 | 5.60491 | -0.00034 |
| C | 4.52054 | 4.01161 | -0.00042 |
| C | 2.74248 | 5.65803 | -0.00051 |
| C | 0.97848 | 7.33094 | -0.00056 |
| C | -1.39284 | 6.60975 | -0.00044 |
| H | -3.39722 | 5.85653 | -0.00033 |
| C | 4.10827 | 5.33275 | -0.00053 |
| H | 5.57485 | 3.76993 | -0.00044 |
| C | 2.29732 | 7.02309 | -0.00059 |
| H | 0.65375 | 8.36356 | -0.00062 |
| H | -1.69692 | 7.64870 | -0.00051 |
| H | 4.83835 | 6.13219 | -0.00062 |
| H | 3.04514 | 7.80558 | -0.00066 |
| H | 3.91611 | 1.95571 | -0.00020 |
| H | -2.69762 | 3.48186 | -0.00017 |
