# Supplementary material for: Electron‐Rich Diruthenium Complexes with π‐Extended Alkenyl Ligands and Their F4TCNQ Charge‐Transfer Salts
Source: Chemistry. 2022 Mar 18;28(23):e202104403. doi: 10.1002/chem.202104403 (PMC9310581; doi:10.1002/chem.202104403)
Supplement: Supplementary file 2 — Supporting Information [file CHEM-28-0-s002.pptx]

## Slide 1
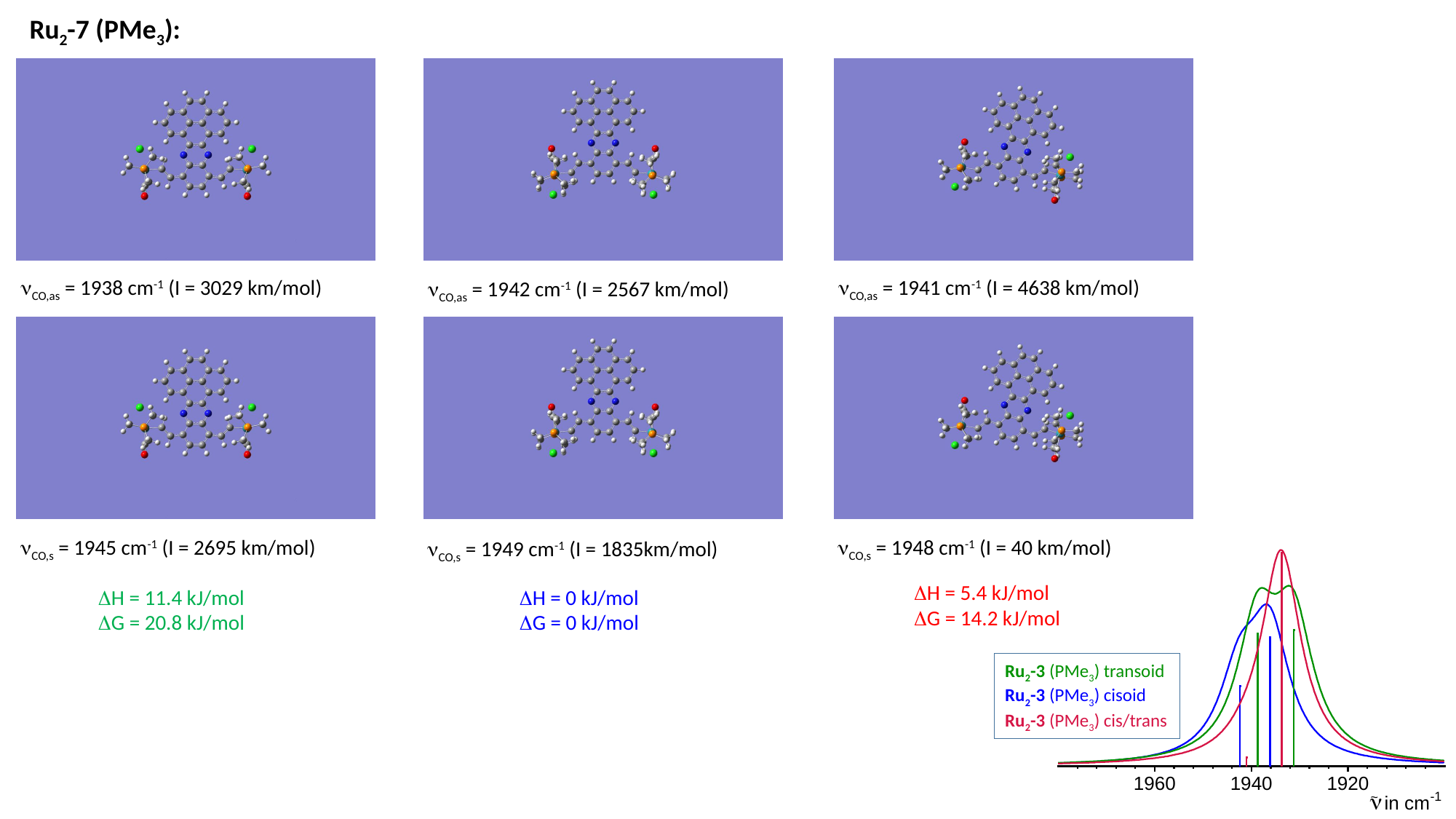

Ru2-7 (PMe3):
nCO,as = 1938 cm-1 (I = 3029 km/mol)
nCO,as = 1941 cm-1 (I = 4638 km/mol)
nCO,as = 1942 cm-1 (I = 2567 km/mol)
nCO,s = 1945 cm-1 (I = 2695 km/mol)
nCO,s = 1948 cm-1 (I = 40 km/mol)
nCO,s = 1949 cm-1 (I = 1835km/mol)
DH = 5.4 kJ/mol
DG = 14.2 kJ/mol
DH = 11.4 kJ/mol
DG = 20.8 kJ/mol
DH = 0 kJ/mol
DG = 0 kJ/mol
Ru2-3 (PMe3) transoid
Ru2-3 (PMe3) cisoid
Ru2-3 (PMe3) cis/trans
